# De Novo Transcriptome Analysis of the Venom of *Latrodectus geometricus* with the Discovery of an Insect-Selective Na Channel Modulator

**DOI:** 10.3390/molecules27010047

**Published:** 2021-12-22

**Authors:** Pornsawan Khamtorn, Steve Peigneur, Fernanda Gobbi Amorim, Loïc Quinton, Jan Tytgat, Sakda Daduang

**Affiliations:** 1 Program in Research and Development in Pharmaceuticals, Faculty of Pharmaceutical Sciences, Khon Kaen University, Khon Kaen 40002, Thailand; pornsawan.k@kkumail.com; 2 Toxicology and Pharmacology, Campus Gasthuisberg, University of Leuven (KU Leuven), 3000 Leuven, Belgium; steve.peigneur@kuleuven.be (S.P.); jan.tytgat@kuleuven.be (J.T.); 3 Laboratory of Mass Spectrometry, MolSys Research Unit, Department of Chemistry, University of Liège, 4000 Liège, Belgium; fernandagamorim@gmail.com (F.G.A.); loic.quinton@uliege.be (L.Q.); 4 Center for Research and Development of Herbal Health Products (CDR-HHP), Faculty of Pharmaceutical Sciences, Khon Kaen University, Khon Kaen 40002, Thailand; 5 Protein and Proteomics Research Center for Commercial and Industrial Purposes (ProCCI), Khon Kaen University, Khon Kaen 40002, Thailand; 6 Division of Pharmacognosy and Toxicology, Faculty of Pharmaceutical Sciences, Khon Kaen University, Khon Kaen 40002, Thailand

**Keywords:** transcriptome, *Latrodectus geometricus*, venom, toxin, spider, ion channel, insect sodium channels

## Abstract

The brown widow spider, *Latrodectus geometricus*, is a predator of a variety of agricultural insects and is also hazardous for humans. Its venom is a true pharmacopeia representing neurotoxic peptides targeting the ion channels and/or receptors of both vertebrates and invertebrates. The lack of transcriptomic information, however, limits our knowledge of the diversity of components present in its venom. The purpose of this study was two-fold: (1) carry out a transcriptomic analysis of the venom, and (2) investigate the bioactivity of the venom using an electrophysiological bioassay. From 32,505 assembled transcripts, 8 toxin families were classified, and the ankyrin repeats (ANK), agatoxin, centipede toxin, ctenitoxin, lycotoxin, scorpion toxin-like, and SCP families were reported in the *L. geometricus* venom gland. The diversity of *L. geometricus* venom was also uncovered by the transcriptomics approach with the presence of defensins, chitinases, translationally controlled tumor proteins (TCTPs), leucine-rich proteins, serine proteases, and other important venom components. The venom was also chromatographically purified, and the activity contained in the fractions was investigated using an electrophysiological bioassay with the use of a voltage clamp on ion channels in order to find if the neurotoxic effects of the spider venom could be linked to a particular molecular target. The findings show that U24-ctenitoxin-Pn1a involves the inhibition of the insect sodium (Na_v_) channels, BgNa_v_ and DmNa_v_. This study provides an overview of the molecular diversity of *L. geometricus* venom, which can be used as a reference for the venom of other spider species. The venom composition profile also increases our knowledge for the development of novel insecticides targeting voltage-gated sodium channels.

## 1. Introduction

Over 49,000 species of spiders belonging to the order Araneae are reported in the World Spider Catalog [1]. Spiders represent a large group of venomous animals. Their venom is effective in paralyzing, killing, and digesting prey. Spiders are mostly insect hunters [2]. Furthermore, spider bites can be dangerous to humans because their venom includes complex combinations of bioactive compounds that can cause local and systemic symptoms. Several toxins have been studied, in particular peptides, targeting ion channels important in specific neurological disorders [3,4].

The spider genus *Latrodectus* is found in the pantropical and subtropical regions of the world [5,6]. Their venoms contain latrotoxins, which are the main neurotoxins in the venom. The venom of *Latrodectus geometricus*, commonly known as the brown widow spider, has powerful toxicity that affects both mammals and insects. It can be found in agricultural areas, garages, and even houses. There is no surprise that cases of *L. geometricus* spider envenomation have been reported [7,8,9]. Therefore, *Latrodectus* is considered one of the most dangerous spider genera to humans and animals.

Previous research on the venom composition and antivenom production against *L. geometricus* venom has resulted in a better understanding of this spider [2,10,11,12,13,14,15,16,17]. Besides the neurotoxins, serine proteases, metalloproteases, hyaluronidases, chitinases, venom allergen antigen 5-like proteins, and antibacterial activity have been reported in *L. geometricus* venom [18].

In recent years, the transcriptomics approach has been utilized to identify the peptides, proteins, enzymes, and other components found in spider venom glands [19,20,21,22,23,24,25,26,27,28,29]. Even though transcriptome analyses have been used to study the gene expression level and function of venom components in various spiders, there is currently a lack of transcriptome information regarding *L. geometricus* venom.

Besides the transcripts revealed by transcriptomics, spider venom peptides are also investigated as a potential source for therapeutic leads or bioinsecticides [30,31] because spider venoms commonly target the nervous system, especially the ion channels of their prey or predators. Spider venoms have been investigated and have been shown to be a possible source of insecticidal peptides that cause insect paralysis or mortality by modulating ion channels, receptors, and enzymes. One-third of the spider venom-derived ion channel modulators listed in ArachnoServer (www.uniprot.org, accessed on 6 July 2021) target voltage-gated sodium (Na_v_) channels [32]. Ctenitoxins are found in several spider species, including *Phoneutria nigriventer* [33] and *Latrodectus tredecimguttatus* [34]. Their functions have been reported as the protease inhibitor and the Na_v_ channel modulator, particularly insect Na_v_ channels [33,35].

In this study, we investigated the venom gland of *L. geometricus* using high-throughput next-generation sequencing (NGS). This technology allowed detailed information of the venom components to be obtained. We also used electrophysiological techniques to screen the venom component against several ion channels of both humans and insects. These findings will contribute to a better understanding of spider venoms. Moreover, we report for the first time the presence of insect selective Na_v_ channel modulators in the venom of *Latrodectus* spiders.

## 2. Results

### 2.1. Illumina Transcriptome Sequencing, De Novo Assembly, and Annotation

**Figure 1:**
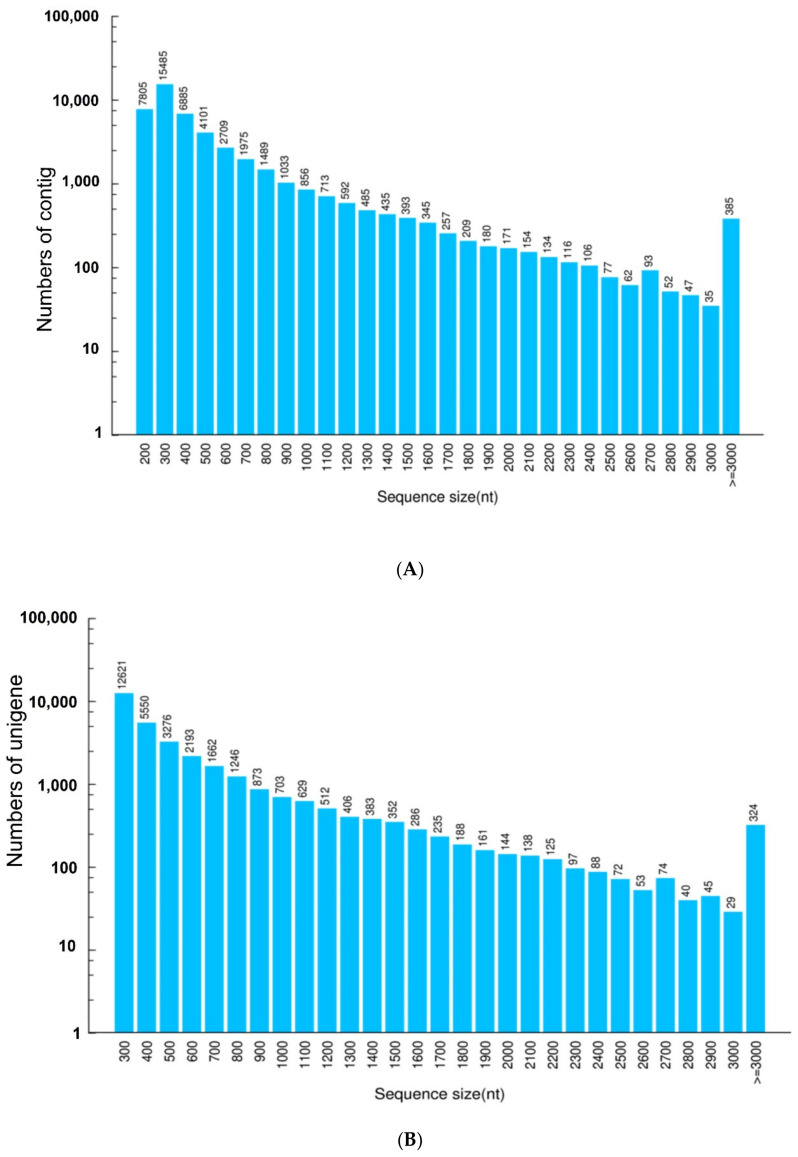
Size distributions of (**A**) contig and (**B**) unigene in the transcriptome of the *Latrodectus geometricus* venom gland. The sequence size is represented on the x-axis, while the numbers of contig and unigene are represented on the y-axis.

To obtain an overview of the proteins expressed in *L. geometricus* venom glands, illumina sequencing was performed. A cDNA library was constructed from mRNA extracted from twenty adult female *L. geometricus* spiders. A total of 85,709,012 raw reads were obtained after high-throughput paired-end sequencing. Afterwards, the dataset quality was checked using the FastQ program, and the adapters were removed. The remaining clean reads totaled 67,659,540 high-quality reads, which were assembled using the Trinity software (version V2.0.6). The details of the output sequencing data are shown in Table 1. Using paired-end joining and gap filling, contigs were further assembled into 32,505 unigenes with a length of >200 bp, of which 324 unigenes were ≥3000 bp. The total read length was 18,752,035 nt, the mean length was 576 bp, and the median unigene size (N50) was 770 bp. In addition, the percentage of GC was 34%. Figure 1 shows the size distributions of contigs and unigenes, and the data are summarized in Table 2.

**Figure 2:**
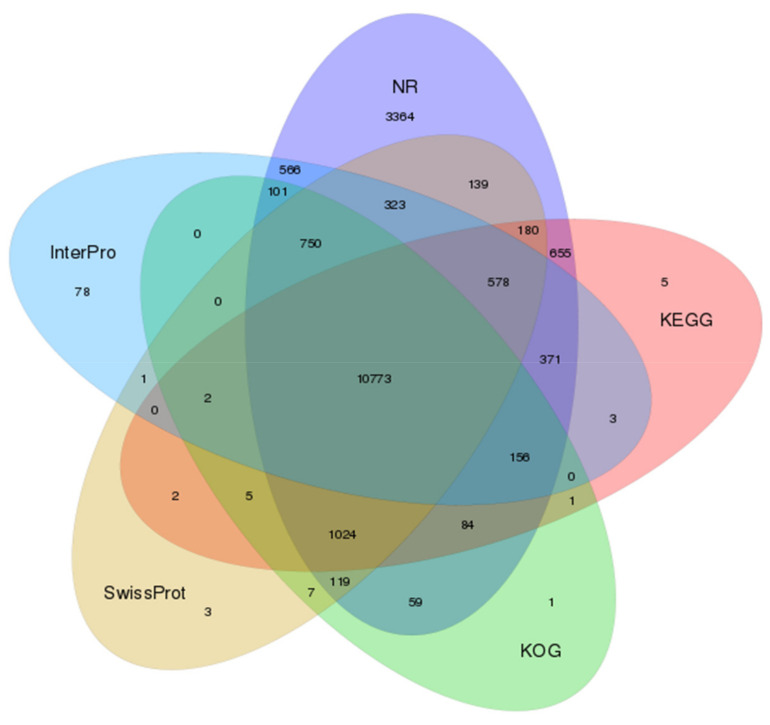
Venn diagram of unigenes annotated with InterPro, NR, KEGG, KOG, and SwissProt databases.

**Figure 3:**
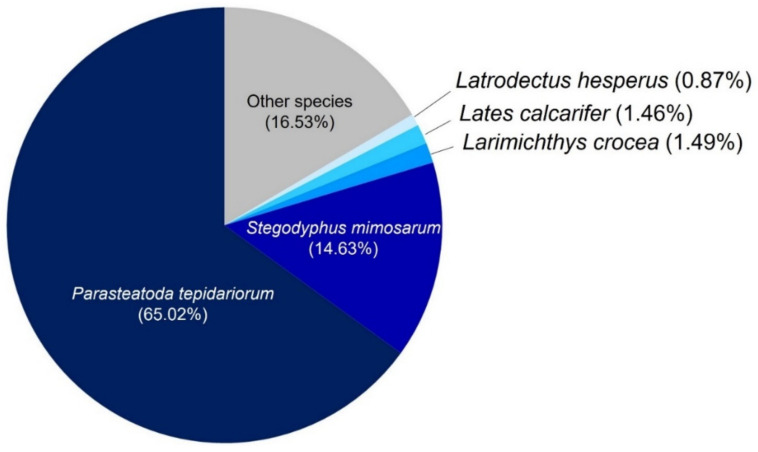
Species distribution showing the proportions of different species associated with the unigene annotations according to the results of the NR annotations.

Due to the absence of a reference genome for *L. geometricus*, the assembled unigenes were blast searched in five databases: (1) InterPro, (2) Non-redundant (NR), (3) Kyoto Encyclopedia of Genes and Genomes (KEGG), (4) EuKaryotic Orthologous Groups (KOG), and (5) SwissProt public protein databases. The Venn diagram presents the number of genes that were uniquely expressed within each group, with the overlapping regions showing the number of genes that were expressed in five databases, as shown in Figure 2. A total of 10,773 unigenes were annotated with all databases. These included: 13,702 unigenes annotated in the InterPro database, 19,242 unigenes annotated in the NR database, 13,839 unigenes annotated in the KEGG database, 13,082 unigenes annotated in the KOG database, and 13,906 unigenes annotated in the SwissProt database. The annotations of these databases are summarized in Supplementary Materials, Table S1. These findings provide a foundation for further investigation into the protein functions of the annotated unigenes. Furthermore, the species with the great number of *L. geometricus* unigenes were *Parasteatoda tepidariorum* (65.02%), *Stegodyphus mimosarum* (14.63%), *Larimichthys crocea* (1.49%), *Lates calcarifer* (1.46%), *Latrodectus hesperus* (0.87%), and *L. tredecimguttatus* (0.07%), as shown in Figure 3. This result indicated that our sequences were matched with spider species more than other animal species.

### 2.2. Gene Ontology (GO) Functional Annotation

**Figure 4:**
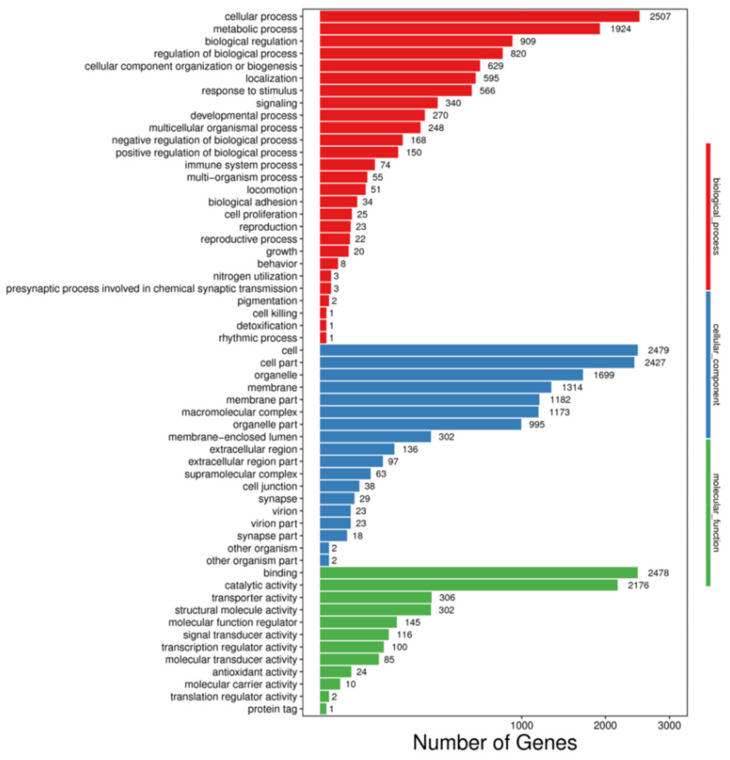
Gene ontology classifications of assembled unigenes *Latrodectus geometricus* venom gland. The figure shows a summary classification in three categories: biological process, cellular component, and molecular function.

Approximately 27,196 genes were classified into three categories based on the GO functional annotation: biological process, cellular component, and molecular function. A number of classified genes was associated with cellular process (2507 genes, 9.22%) and metabolic process (1924 genes, 7.07%) of the biological process. The cellular component was mainly associated with the cell (2479 genes, 9.12%) and cell part (2427 genes, 8.92%). The molecular function was binding (2478 genes, 9.11%) and catalytic activity (2176 genes, 8.00%) (Figure 4). Biological processes such as cellular processes, metabolic processes, and biological regulation were plentiful, showing the venom gland is highly metabolically active with protein synthesis. Additionally, transcripts with molecular functions relevant to protein processing and synthesis, such as RNA and DNA binding and catalytic activity, were plentiful in venom gland cells, indicating that venom gland activity is related to venom production.

**Figure 5:**
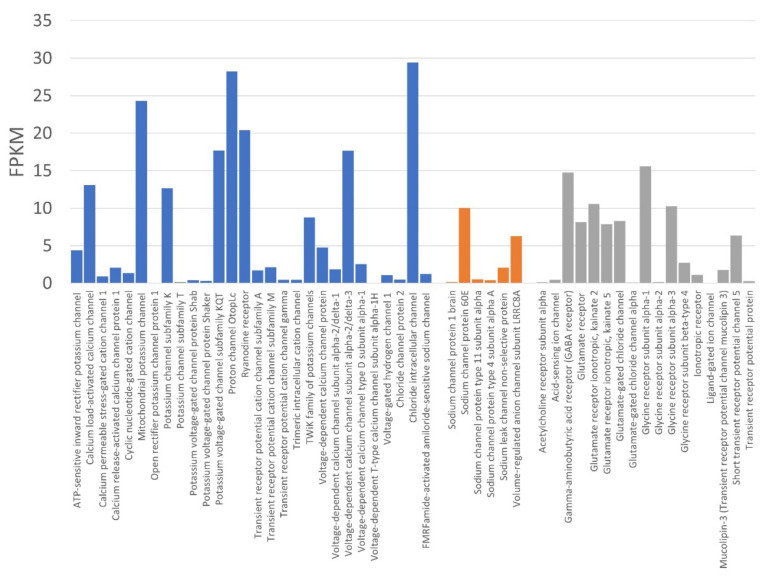
The expressed ion channels of the *Latrodectus geometricus* venom gland.

One of the fascinating aspects of venom gland cells is how venomous animals manage to withstand their own toxins. Certainly, the main targets of spider venom for paralysis or prey capture are voltage-gated sodium, potassium, and calcium channels. Therefore, we investigated the expression of these ion channels in the venom gland and discovered that voltage-gated potassium channels protein *Shab*, *Shaker*, and subfamily KQT (Fragments per Kilobase of transcript, per Million fragments sequenced (FPKM) = 0.41, 0.32, and 17.71, respectively) and voltage-gated calcium channels (average of the FPKM = 5.38) were expressed at low expression levels (Figure 5); in addition, other voltage-gated sodium, potassium, and calcium channels and α-latrotoxin (LTX) receptors were not detected in the venom gland of *L. geometricus* (Supplementary Materials, Table S2). The results indicated that the venom gland with its tissue-specific absence of toxin targets in the venom gland cells makes these immune to their toxin, according to [34].

The FPKM method was used to estimate the gene expression in the venom gland transcriptome. The 20 most expressed unigenes are shown in Supplementary Materials, Table S3. α-Latrotoxin (α-LTX), associated low molecular weight protein (LMWP) 2, U4-lycotoxin-Ls1a, ω-ctenitoxin-Cs1a, and U-scoloptoxin (01)-cw1a were found to be expressed. In addition, proteins for venom reinforcement such as nepprilysin-2, cysteine-rich secretory proteins (CRISPs)/Allergen/PR-1, and troponin C, were also found. Unsurprisingly, some mitochondrial genes (e.g., cytochrome oxidase, ATP synthase, and pre-mRNA splicing factor) and muscle proteins (actin, muscle protein, myophilin, and myosin) were highly expressed in the venom gland.

### 2.3. Toxinome of Latrodectus geometricus

**Figure 6:**
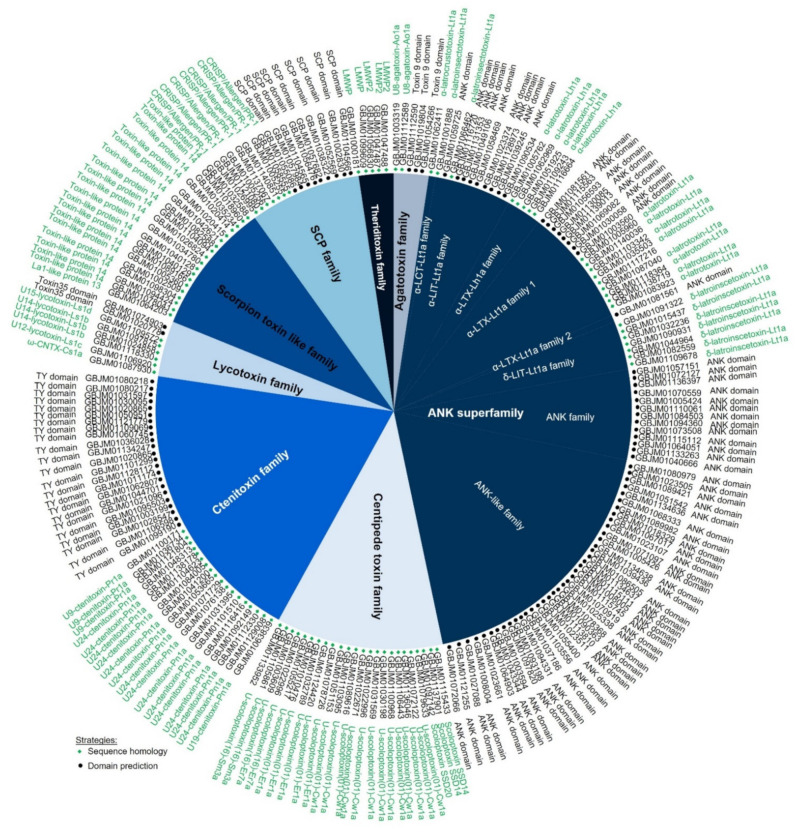
Toxinome of *L. geometricus* venom gland. All 212 unigenes were clustered using sequence homology and domain prediction.

**Figure 7:**
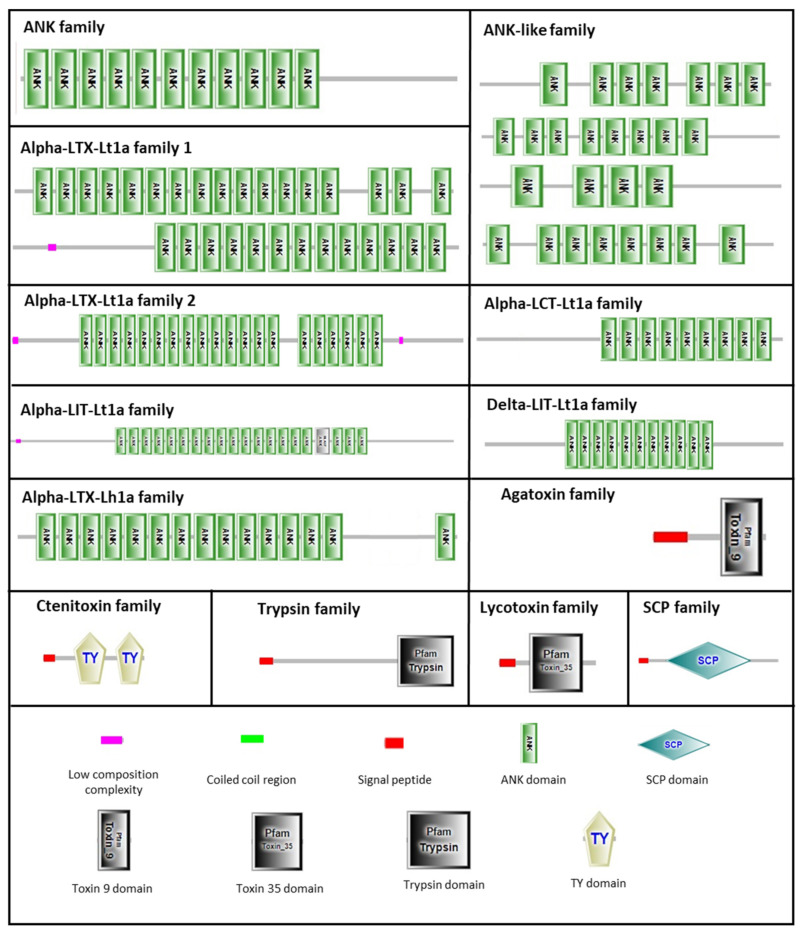
Domain architecture of *Latrodectus geometricus* toxins was predicted by the simple modular architecture research tool (SMART) and the protein family database (Pfam) servers (http://www.smart.embl-heidelberg.de, accessed on 4 April 2021 and http://pfam.janelia.org, accessed date 4 April 2021) [42,43]. All toxins were grouped based on the specific family classification. This scheme presents only domain matching among all unigenes. The abbreviations of domain names are as follows: ANK (SMART ID: SM00248); TY (SMART ID: SM00211); toxin 35 (Pfam ID: PF10530); toxin 9 (Pfam ID: PF02819); SCP (SMART ID: SM00198); trypsin (Pfam ID: PF00089).

We used two strategies to identify the potential toxins: sequence homology searching and domain prediction. The 212 unigenes were classified into eight families; ANK superfamily and six other families, including agatoxin, centipede toxin, ctenitoxin, lycotoxin, scorpion toxin-like, and SCP families, except for the theriditoxin family, were all reported in *L. geometricus*. All the unigenes of clustered toxinome using two strategies are shown in Figure 6. Figure 7 presents the domain architectures of some unigenes that indicate the different domains and functions. The toxin classifications are summarized in Supplementary Materials, Table S4. The alignment sequences are shown in Supplementary Materials, Figure S1.

#### 2.3.1. Agatoxin Family

There are five members in the agatoxin family, with two members being homologous with U8-agatoxin-Ao1a (UniProt ID: Q5Y4U4) from the funnel-web spider (*Agelena orientalis*). This toxin contains five disulfide bonds in its structure, and its function is associated with ion channel inhibitor activity. In addition, toxin 9 domain (Pfam ID: PF0281) is a spider toxin also included in this family, with three members (Figure 6 and Supplementary Materials, Table S4). These toxins share a common structural motif composed of a three-stranded antiparallel beta-sheet, which can stabilize by internal disulfide bonds known as cystine knots [36], and they also function as ion channel blockers [37].

#### 2.3.2. ANK Superfamily

The ANK superfamily was characterized by the presence of several ANK domain repeats (ankyrin repeat, SMART ID: SM00248) and contained eight families: α-latrocrustotoxin-Lt1a (α-LCT-Lt1a), α-latrotoxin-Lh1a (α-LTX-Lh1a), α-latrotoxin-Lt1a (α-LTX-Lt1a) family 1, α-LTX-Lt1a family 2, α-latroinsectotoxin-Lt1a (α-LIT-Lt1a), δ-latroinsectotoxin-Lt1a (δ-LIT-Lt1a), ANK family, and ANK-like family (Supplementary Materials, Table S4). The ANK domain is approximately 33 amino acid residues long, and the structure contains a helix–loop–helix structure. The number and distribution of ANK domains differ in this superfamily (Figure 7); for instance, the α-LTX-Lt1a family 2 contains 20 ANK domains located in the central part, and the ANK pattern is split into two parts (14 + 6). For the α-LIT-Lt1a family, consecutive ANK domains are located on the central part of toxins, whereas diverse patterns of ANK domains are found in the α-LTX-Lt1a family 1 and ANK-like families. The function of the ANK domain involves protein–protein interactions and may direct their binding to receptors, which are neurexin and latrophilin receptors located in presynaptic membrane cells. These receptors are directly bound by the α-LTX-Lt1a and α-LIT-Lt1a families to induce neurotransmitter vesicle exocytosis via both calcium (Ca^2+^)-dependent and -independent mechanisms, resulting in vesicle depletion, causing the massive neurotransmitter release [38,39]. It is well described that proteins belonging to the α-LTX-Lt1a family are the main neurotoxins responsible for the symptoms occurring upon vertebrate envenomation [2,40]. α-LTX-Lt1a family proteins specifically bind neuronal cell receptors on the presynaptic membrane. The α-LIT-Lt1a and δ-LIT-Lt1a families, which lack the C-terminal region of the ANK domain, were reported to induce neurotransmitter release only in insects [41]. Only one sequence shared homology with the α-LCT-Lt1a family, which targets only crustaceans, and five members were matched with the α-LTX-Lh1a family, which has the same function as the α-LTX-Lt1a family but is active in different species. Additionally, different amino acid residues and consecutive ANK domains within these families suggest that the gene may generate diverse functions [34].

#### 2.3.3. Centipede Toxin like Family

The centipede toxin-like family includes twenty-four members with homology to scoloptoxin SSD14, U-scoloptoxin (01)-Cw1a, U-scoloptoxin (01)-Er1a, U-scoloptoxin (16)-Er7a, and U-scoloptoxin (16)-Sm3a (Supplementary Materials, Table S4). The function of these toxins involved the inhibition of voltage-gated sodium (Na_v_), potassium (K_v_), and calcium (Ca_v_) channels [44].

#### 2.3.4. Ctenitoxin Family

The ctenitoxin family contains two consecutive thyroglobulin type I repeats (TY) domains (SMART ID: SM0021) and a predicted signal peptide in the sequence (Figure 7). The same TY domain architecture was also found in other spider venoms, such as *Cyriopagopus schmidti* (SWISSPORT ID: B5M6G6), *Phoneutria nigriventer* (SWISSPORT ID: P84032), and *Latrodectus tredecimguttatus* [34]. The function of the TY domain has been proposed to be a cysteine protease inhibitor [45], indicating that the members in this family may function as cysteine protease inhibitors. Other toxins were homologous of known ctenitoxins (U9-CNTX-Pr1a, U19-CNTX-Pn1a, and U24-CNTX-Pn1a) from *P. reidyi* and *P. nigriventer* (Supplementary Materials, Table S4). However, several studies have been reported that ctenitoxins completely inhibited the insect Na_v_ channel (BgNa_v_) and the arachnid channel (VdNa_v_), resulting in sustained non-inactivating currents [33,35].

#### 2.3.5. Lycotoxin Family

Eight members were identified to be members of the lycotoxin family. Six of these eight were homologous, with ω-ctenitoxin-Cs1a (ω-CNTX-Cs1a), U12-lycotoxin-Ls1a (U12-LCTX-Ls1a), U14-lycotoxin-Ls1b (U14-LCTX-Ls1b), and U15-lycotoxin-Ls1d (U15-LCTX-Ls1d) from the *Cupiennius salei* and *Lycosa singoriensis* venoms (Supplementary Materials, Table S4). Other members shared a characteristic inhibitor cystine knot (ICK)-like motif (toxin-35 domains), which is common for spider toxins acting on ion channels [46,47]. The protein containing the toxin 35 domain in *L. tredecimguttatus* venom is similar to the CSTX-1 family, which are voltage-gated calcium channel inhibitors [34]. Therefore, these toxins may operate as a blocker of neuronal signal transduction by interacting with ion channels. In addition, there was one sequence homologous with ω-CNTX-Cs1a from *C. salei*. This toxin contains four disulfide bonds in its structure, and it was defined as a knottin protein that targets the neuronal system of arthropods. Moreover, ω-CNTX-Cs1a is a potent insecticidal peptide with the median lethal dose (LD_50_) values in the picomolar range when injected in drosophila [48]. This toxin acts as an inhibitor of voltage-activated calcium channels [47] and might be an interesting candidate for the development of insecticides [49,50].

#### 2.3.6. Scorpion Toxin like Family

This family contains nineteen members that shared homology with La1-like protein 13 and toxin-like protein 14 from scorpion *Urodacus yaschenkoi* venom [51], and they were expressed at a low level (Supplementary Materials, Table S4). The function of this family is unknown.

#### 2.3.7. SCP Family

The SCP family includes sixteen members and was identified because it contains an SCP domain. The SCP domain (SMART ID: SM00198) (Figure 7), also known as the cysteine-rich secretory proteins, antigen 5, and pathogenesis-related 1 proteins (CAP) domain, is a cysteine-rich domain generally found in secretory proteins. This domain is involved in several processes, including the regulation of the extracellular matrix and branching morphogenesis and ion channel regulation in fertility, as well as in cell–cell adhesion during fertilization [52]. Proteins containing an SCP domain are, for example, the cysteine-rich secretory proteins (CRISPs), allergen 5, and plant pathogenesis-related protein 1 (PR-1). CRISPs have been highlighted as the most common member in several arthropod venoms, including fire ants, scorpions, and spiders [52]. The ability of proteins containing this domain to block ryanodine receptors, which are large ion channels responsible for the release of Ca^2+^ from the sarco/endoplasmic reticulum, has been described [53,54]. Moreover, the CRISPs are involved in the stimulation of smooth muscle contraction [55], inflammation [56], and inhibition of angiogenesis [57]. This indicates that these molecules might also have a potential for the development of new drugs.

#### 2.3.8. Theriditoxin Family

This family includes four members that share homology with the α-latrotoxin associated low molecular weight protein (LMWP)1 and LMWP2, which are known as latrodectins with high FPKM (60,435). These latrodectins are predicted to enhance the neurotoxic activity of latrotoxins and also increase the insecticidal activity of α-LTX or other venom components [58,59,60]. Furthermore, according to the UniProt database, latrodectins are exclusively found in the spider genus *Latrodectus* [34] and *Steatoda nobilis*, which is the sister group to *Latrodectus* [61].

### 2.4. Other Venom Components

**Figure 8:**
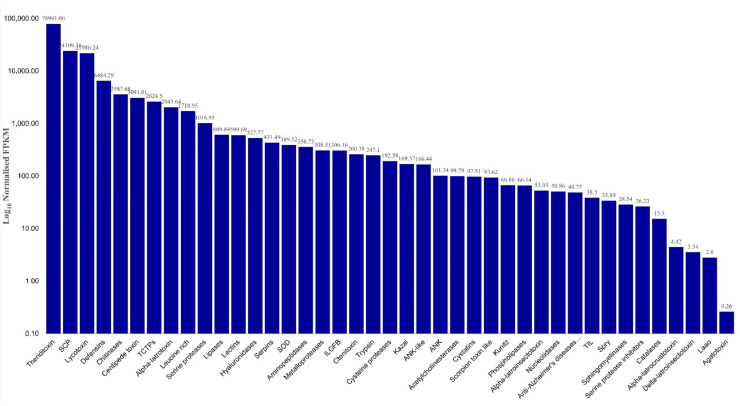
The relative abundance expressed in FPKM of the venom component of the *Latrodectus geometricus* venom gland. Unigene sequences were classified into known toxin subfamilies according to the UniProt database. Bars represent the sum of FPKM for each transcript belonging to the described groups.

In addition to the superfamily classifications from the SMART and Pfam databases, Figure 8 shows the relative abundance in FPKM of other venom components in the *L. geometricus* venom gland.

The venom contains several components, and the highest-expression level (FPKM = 6484.29) presented defensins. Defensins are known antimicrobial peptides (AMPs) that are widely found in organisms such as animals, plants, and fungi [62]. AMPs function in venom is associated with the immune system of animals by interaction with the cell membrane of pathogens, leading to structural and functional cellular disruption [63], and they are also known for protecting themselves from infesting infectious organisms [18]. Oh-defensin, isolated from the spider *Ornithoctonus hainana*, has shown broad-spectrum antimicrobial activity and hemolysis activity [64]. This information suggests that defensins in spider venom have a role in antibacterial activity.

Chitinases were the second most represented component, with a high expression level (FPKM = 3587.68). These enzymes may play an important role in feeding habits because chitins are the main component of arthropod exoskeletons. Unsurprisingly, they were found in many species of spider venoms such as *L. hesperus* and *P. nigriventer* [65,66].

Translationally controlled tumor proteins (TCTPs) were first described in human carcinoma; in addition, the proteins are also related to histamine-releasing factors and other physiological activities in cells, such as cell proliferation, tumor reversion, and cell death [67]. TCTPs have also been described in spiders and other venomous animals. For example, a recombinant TCTP from the spider *Loxosceles intermedia* induced paw edema and increased vascular permeability in mice [68]. Indeed, TCTPs have been assumed to induce the local inflammatory reaction often observed upon envenomation [20].

Leucine-rich repeat (LRR) proteins are the fourth most represented in *L. geometricus* venom, and it has also been reported in other spider species such as *C. salei* [46], *L. hesperus*, and *L. tredecimguttatus* [65]. This protein may have a role in the immune system of spiders, and it is believed to be the protection of the spider venom gland against pathogen infections [63].

Serine proteases were found in the *L. geometricus* venom gland, and they were expressed in high levels (FPKM = 1016.95). Even though they have been studied tremendously in snake venom [68], they are also found in several arachnid venoms that have been studied by transcriptomics, proteomics, and experimental approaches [69,70,71]. Several roles have been suggested for serine proteases in spider venom, including acting in toxin maturation, victim digestion, hemostasis impairment [72], and direct tissue damage.

Furthermore, protease inhibitors in the *L. geometricus* venom gland were also found, such as serpins, kazal-type inhibitors, cystatins, Kunitz-type inhibitors, trypsin inhibitors such as cysteine-rich domain (TIL)-type inhibitors, and serine protease inhibitors. Moreover, the transcriptomics analysis revealed other potential components in small portions, including lipases, phospholipases, superoxide dismutases (SOD), aminopeptidases, metalloproteases, insulin-like growth factor-binding proteins (IGFBP), cysteine proteases, hyaluronidases, acetylcholinesterases (ACE), nucleotidases, anti-Alzheimer’s diseases peptides, SPRY domain-containing proteins, sphingomyelinases, catalases, and L-amino-acid oxidases (LAAO) in the present study.

### 2.5. Screening Activity of Crude Venom on Voltage-Gated Ion Channels

**Figure 9:**
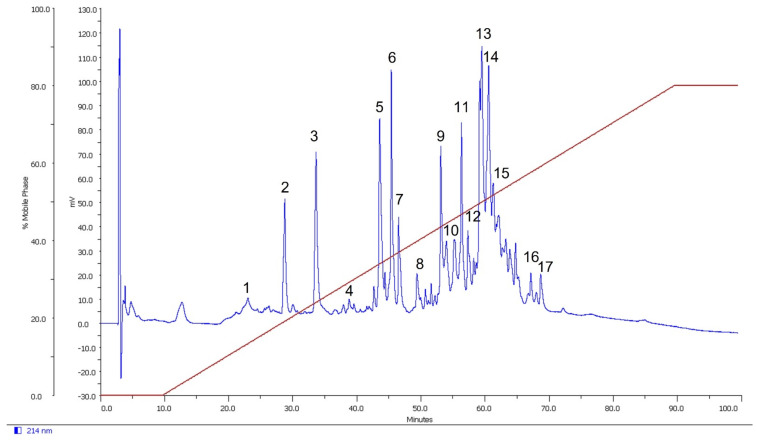
Reverse-phase HPLC separation on Vydac C18 of *Latrodectus geometricus* venom. The chromatogram presents the separated fractions from the crude venom separated at a flow rate of 1 mL/min. The red line indicates the gradient of acetonitrile in 0.085% (*v*/*v*) aqueous trifluoroacetic acid.

**Figure 10:**
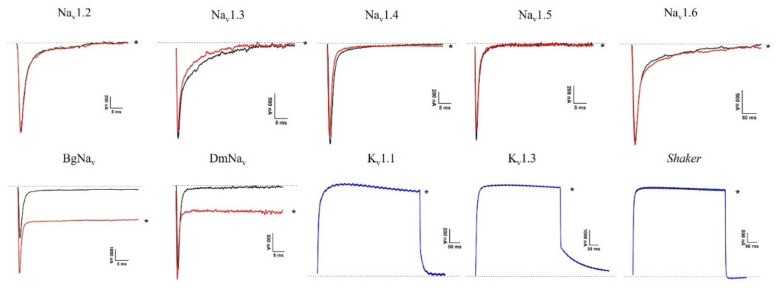
Electrophysiological profiles of *L. geometricus* venom fraction 14 on Na_v_ and K_v_ channels. Panels show superimposed current traces of 0.4 µg/µL fraction 14. Black indicates a current trace in control conditions; red or blue indicates a current trace in toxin situation. The dotted line indicates zero current level. The asterisk marks steady-state current trace after application of toxin.

**Figure 11:**
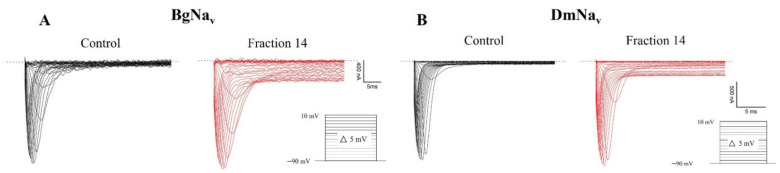
The effects on the insect BgNa_v_ and DmNa_v_ channels induced by fraction 14 on currents of the cloned insect BgNa_v_ channels (**A**) and DmNa_v_ channels (**B**), expressed in *Xenopus* oocytes. Current traces were elicited by 5 mV step depolarizations in control (black) and the presence of 0.4 µg/μL fraction 14 (red). The dotted line indicates zero current level.

This study aimed to investigate the ability of spider venom to affect ion channels expressed in *Xenopus laevis* oocytes. The crude *L. geometricus* venom was separated using the reverse-phase high-performance liquid chromatography (RP-HPLC) column C18 with a linear gradient of 0–80% acetonitrile (I). RP-HPLC separation resulted in 17 fractions of venom, as shown in Figure 9. All the fractions were screened for activity on mammalian (voltage-gated sodium (Na_v_) channels: Na_v_1.2–1.6 and voltage-gated potassium (K_v_) channels: K_v_1.1, K_v_1.3, channels) and insect (insect Na_v_ channels from *Blattella germanica* (BgNa_v_), *Drosophila melanogaster* (DmNa_v_), and insect K_v_ channel from *Shaker* IR). There was no effect on mammalian channels after the application of the fractions (data not shown). On the other hand, fraction 14 slowed down the inactivation of the insect channels BgNa_v_ and DmNa_v_ (Figure 10). On currents elicited by depolarizations to −5 mV, addition of 0.4 μg/μL fraction 14 resulted in two effects: (i) The peak of transient sodium currents, BgNa_v_ channel (49.32 ± 4.5%), and DmNa_v_ channel (2.5 ± 0.4%) (*n* = 3) increased. (ii) The time-to-peak after fraction 14 addition decreased from 1.07 ± 0.12 to 0.95 ± 0.10 ms and 2.57 ± 0.25 to 1.59 ± 0.34 ms (*n* = 3) of BgNa_v_ and DmNa_v_ channels, respectively. (Figure 11). In fact, fraction 14 inhibited the sodium current through insect Na_v_ channels while another fraction did not show activity. Additionally, no activity was observed on *Shaker* IR channels.

### 2.6. Shotgun Proteomics of Latrodectus geometricus

In order to obtain a complete overview of the fraction 14 composition, shotgun proteomics methodology was performed. We used two algorithms for fraction 14 analysis. Firstly, after the database match on the PEAKS algorithm, we obtained 33 sequences which were grouped into 14 proteins. They were majority cellular components; however, we obtained four unique peptides related to U24-ctenitoxin-Pn1a from the *Parasteatoda tepidariorum* spider, which represents 11% of the sequence coverage. For the SPIDER algorithm, we obtained 102 sequences, which were grouped into 30 proteins. The result from this algorithm showed that most of the proteins were cellular components. Four unique peptides related to U24-ctenitoxin-Pn1a (close to 14% of sequence coverage). The coverages for each analysis and the best unique peptide spectrum matches are available in Supplementary Materials, File S2 and S3.

## 3. Discussion

The transcriptome of the *Latrodectus geometricus* venom gland was analyzed using high-throughput sequencing in this study. The quantitative and functional investigations revealed a function associated with tissue-specific gene expression. To begin with, highly expressed transcripts were significantly abundant in protein synthesis and metabolic pathways, both of which are required for toxin translation, transportation, and secretion. In addition, numerous extracellular and secreted proteins, particularly toxins, were abundant. Finally, most ion channels expected to be the target of spider toxins were not expressed or were expressed at low levels in the venom gland cells, indicating that they may be protected from the toxins present in the venom gland. There are three proposed strategies for preventing toxin assault in venom gland cells: (1) low or non-expression level of toxin targets, (2) inhibition of toxin maturation, and (3) mutation of toxin targets [73,74]. Our results support the first strategy that the ion channels, which are the toxin target, are low or not expressed in the venom gland cells.

In a previous study of transcriptome of the black widow spider, *L. tredecimguttatus*, venom gland, the authors used three strategies from clustering the toxin family, including sequence homology, domain prediction, and cysteine pattern [34], and eight families were clustered from the black widow spider venom gland. In comparison with the previous study [34], we used sequence homology and domain prediction strategies for clustering the toxins. We report the first time to find two more families, including the agatoxin and centipede toxin families in the spider *Latrodectus* venom gland. Furthermore, the quantification of FPKM also uncovered other venom components in the venom gland (Figure 8), and the defensins, TCTPs, and leucine-rich are only found in the *L. geometricus* spider (Table 3). Transcriptome analyses of other species have contributed to the understanding of the molecular basis involved in the venom component, and our finding corresponds with previous transcriptomics studies in the spider genus *Latrodectus* [34,67] and genus *Steatoda* [61], as shown in Table 3. We speculate that the functions of diverse components in the venom gland are coordinated, such as neurotoxins acting on the nervous system by inducing a neurotransmitter release, inhibiting ion channels, and paralyzing or killing prey. In addition, assistant toxins may enhance the toxicity of neurotoxins by promoting the binding of neurotoxins or by protecting them from protein degradation. Moreover, proteases may cleave precursor toxins into mature toxins. Protease inhibitors protect toxins and other venom components from breakdown by insect protease activity.

The chromatogram in this study presented the familiarity in our previous proteome study in *L. geometricus* venom [18]. The active components (fraction14) elute rather later in the gradient of the reverse phase, meaning it has a significant degree of hydrophobicity. By shotgun proteomics of fraction 14, we identified a peptide similar to U24-ctenitoxin-Pn1a from the *Parasteatoda tepidariorum* spider. This toxin group was already described as an insect toxin that can bind to Na^+^ ion channels [33]. The electrophysiological study of fraction 14 was performed on the insect and mammalian Na_v_ and K_v_ channels expressed in *X. laevis* oocytes, revealing new information on how the *L. geometricus* spider venom functions. This fractionated toxin causes two effects on the currents of the insect BgNa_v_ and DmNa_v_ channels, including (1) an increase of peak sodium current, and (2) slowing down of the inactivation of action potential and the shapes of current traces were different those controls, meaning that the fraction 14 selectively inhibited insect BgNa_v_ and DmNa_v_ channels. On the other hand, another insect *Shaker* IR channel and mammalian channels were not affected by fraction14, which was as expected because the natural prey of *L. geometricus* does not include mammals and therefore targets sodium channels more than other ion channels, as mentioned above [32]. It is possible that it can be considered as a lead for bioinsecticide development targeting Na_v_ channels, because the absence of the effect from human ion channels suggests that it will be safe for human health.

Therefore, we summarized that U24-ctenitoxin-Pn1a, which is found in the *L. geometricus* venom gland, involved the inhibition of BgNa_v_ and DmNa_v_ channels. However, the electrophysiological characterization and mode of action of these toxins need to be further investigated.

## 4. Materials and Methods

### 4.1. Specimens

Adult female brown widow spiders (*Latrodectus geometricus*) were collected from wild areas of Khon Kaen province (Khon Kaen, Thailand), and the species were identified by an entomologist. Living spiders were housed following the approval of the Institutional Animal Care and Use Committee of Khon Kaen University (IACUC-KKU-74/61). Spiders were acclimated in airflow for 7 days and fed with cricket (*Gryllus* sp.) twice per week, and they were subjected to fasting before venom collection. *Xenopus laevis* female frogs were purchased from the Centre de Ressources Biologiques Xénopes, France. The use of frogs was in accordance with the license number LA1210239 of the Laboratory of Toxicology & Pharmacology, University of Leuven, Belgium. All animal care and experimental procedures agreed with the guidelines of the “European convention for the protection of vertebrate animals used for experimental and other scientific purposes” (Strasbourg, France, 18 March 1986).

### 4.2. Transcriptomic Analysis

4.2.1. cDNA Library Construction and DNA Sequencing

Paired venom glands from twenty adult female spiders, *L. geometricus*, were used to produce a cDNA library. After immediate immobilization by cold-shocking at −80 °C, venom glands were collected and washed to remove the contaminants in 0.1% diethyl pyrocarbonate (DEPC) water placed on ice. Total RNA was extracted following the TRizol reagent-based method (Sigma). The quality of the RNA extract was checked by gel electrophoresis on a 0.5 M Tris-acetate-EDTA (TAE) agarose gel, and by spectrophotometry using a Nanodrop spectrometer (BioQ). RNA was quantified by size-exclusion chromatography with an Agilent Technologies 2100 Bioanalyzer and using an Agilent RNA 600 Nano Kit. cDNA library construction and sequencing were performed by the Beijing Genomics Institute (BGI, Hong Kong) according to the manufacturer’s instructions (Illumina, San Diego, CA, USA). Briefly, a complementary DNA (cDNA) library was constructed with total RNA extraction from *L. geometricus* spider venom. The mRNAs were isolated from total RNA with oligo (dT) method and were fragmented. First-strand cDNA and second-strand cDNA were synthesized. cDNA fragments were purified and resolved with EB buffer for end reparation and single nucleotide A (adenine) addition. Subsequently, the cDNA fragment was connected with adapters. These cDNA fragments with suitable sizes were selected for the polymerase chain reaction (PCR) amplification. An Agilent 2100 Bioanaylzer and an ABI StepOnePlus Real-Time PCR System were used in the quantification and qualification of those libraries. High-throughput sequencing was conducted using the Illumina HiSeqTM 2000 platform to generate 100-bp paired-end reads.

#### 4.2.2. Raw Data, De Novo Assembly, and Functional Annotation

A reference de novo transcriptome assembly was performed from *L. geometricus* venom reads. Beginning with the raw sequences, they were filtered to remove the low-quality reads. The filtering steps were as follows: (1) removal of reads containing the adaptor sequence; (2) removal of reads containing over 5% of unknown nucleotides “N”; and (3) removal of low-quality reads (those including more than 20% of bases with a quality value lower than 10). The remaining clean reads were used for further analysis. The quality control of reads was accessed by running the FastQ program [75]. Transcriptome de novo assembly was carried out with short paired-end reads using the Trinity software version v2.0.6 with parameters: --min_contig_length 150 --CPU 8 --min_kmer_cov 3 --min_glue 3 --bfly_opts ‘-V 5 --edgethr = 0.1 –stderr’ [76]. After Trinity assembly, the sequences were defined as unigenes. Unigenes, which are here defined as non-redundant assembled sequences obtained from assembly and/or clustering [77], can either form clusters in which the similarity among overlapping sequences is superior to 94%, or singletons that are unique unigenes. As the length of sequences assembled is a recognized criterion for assembly success in terms of contiguity, we calculated the distribution of both contigs and unigenes. To evaluate the depth of coverage, all usable reads were realigned to the unigenes using a SOAP aligner with the default setting [78].

Due to the lack of a reference genome for the *L. geometricus* venom gland, the assembled unigenes were blast searched with five databases: (1) InterPro, (2) Non-redundant (NR), (3) Kyoto Encyclopedia of Genes and Genomes (KEGG), (4) EuKaryotic Orthologous Groups (KOG), and (5) SwissProt public protein databases, with cut-off E-value < 10^−5^ [34]. The sequences were further annotated by Gene Ontology (GO) to understand how genes encode biological functions at the molecular, cellular, and tissue system levels. The unigene expression was evaluated using the “Fragments per kilobase of the transcript, per million fragments sequenced” (FPKM) method. The FPKM value is calculated following the specific formula FPKM = (10^6^ C)/((N × L)/10^3^), where C is the number of fragments shown as uniquely aligned to the concerned unigene, N is the total number of fragments that uniquely align any unigene, and L is the base number in the coding DNA sequence of the concerned unigene. The FPKM method integrates the influence of different gene lengths and sequencing levels on the calculation of gene expression.

#### 4.2.3. Toxin-like Protein Searching

In order to create the precision and accuracy the toxin cluster, we used the sequences from accession number PRJNA376772, which is the multiple organs of the spider genus *Latrodecus* and *Steatoda* sequences obtained from the NCBI-bioproject database as reference sequences for clustering the toxin. The Transdecoder database with default parameter “—at least 40 amino acid residues” was used for translation into peptide sequences. Two strategies were used to identify toxin-like proteins, sequence homology searching, and domain structure [34]. A BlastP search against the Uniprot and Toxprot databases with cut-off E-value < 10^−5^ identified 212 toxin-like proteins homologous with known toxins. In addition, the HMMer database (version v3.3.2) was used for toxin domain structure identification with cut-off E-value < 10^−5^, and the hmm profile (Supplementary Materials, File S1) was constructed based on the Pfam database. The BlastP and HMMer data were combined, and the duplicates data were manually removed. A simple modular architecture research tool (SMART) database (http://smart.embl-heidelberg.de/, accessed on 4 April 2021) was used for comparison of domain structure with all protein that known toxins.

### 4.3. Electrophysiology

#### 4.3.1. Spider Venom Purification

Crude venom from *L. geometricus* was collected using the cold shock method, as described previously [18]. Crude venom was purified with RP-HPLC on an analytical column (4.6 mm × 25 cm) of Vydac C18 (218MS, Technicol Ltd., Stockport, UK) using extended linear gradients of acetonitrile in 0.085% (*v*/*v*) aqueous trifluoroacetic acid at a flow rate of 1 mL/min, and the elution absorbance was monitored at 214 nm. The fractions were collected and lyophilized before use.

#### 4.3.2. Heterologous Expression in *Xenopus laevis* Oocytes

For expression of the mammalian channels (Na_v_1.2–1.6, K_v_1.1, and Kv1.3), the human Na_v_ channels are expressed in the central nervous system (CNS), peripheral nervous system (PNS), cardiac muscle, and skeletal muscle in human cells. The K_v_1.1 and K_v_1.3 subtypes are also expressed in the neuron at CNS in humans; the insect channels (BgNa_v_, DmNa_v_, and *Shaker* IR), and the auxiliary subunits (rβ, hβ, and TipE), were transcribed using the T7 or SP6 mMESSAGE-mMACHINE transcription kit (Invitrogen, Carlsbad, CA, USA). The harvesting of stage V and VI oocytes from anesthetized *X. laevis* has been described in [79]. The oocytes were injected with 30–50 nL of each cRNA channel at a concentration of 1 ng/nL using a microinjector (Drummond Scientific, Broomall, PA, USA). Subsequently, oocytes were incubated in ND96 solution (in mM: NaCl 96, MgCl_2_ 1, CaCl_2_ 1.8, HEPES 5), adjusted to pH 7.5, and supplemented with 50 mg/mL of gentamycin sulfate and 90 mg/L theophylline, at 16 °C for 1–6 days until the expression of its ion channels.

#### 4.3.3. Electrophysiological Recordings

Two-electrode voltage clamp (TEVC) recordings were carried out at room temperature (18–22 °C) using a GeneClamp 500 amplifier (Molecular Devices, San Jose, CA, USA) controlled by a pClamp data acquisition system (Molecular Devices, San Jose, CA, USA). The oocyte currents were recorded after 1–6 days of injection. The bath solution contained ND96 solution without supplements. Electrode capillaries were filled with 3 M KCl, and the electrode resistance was kept between 0.8–1.5 MΩ. The elicited currents were filtered at 2 kHz and sampled at 20 kHz using a four-pole, low-pass Bessel filter. The oocytes were recorded using TEVC with voltage-gated ion channels as Na_v_1.2–1.6, BgNa_v_, DmNa_v_ channels, K_v_1.1, K_v_1.3, and *Shaker* IR channels after application of fractioned venom. The experiment was performed in triplicate. The effect of venoms on the voltage-dependence of Na_v_ channel activation (*I_Na_*) was determined using depolarizing test pulses from −90 to 70 mV, in 5 mV increments. Whereas the effect of the venom on the voltage-dependence of K_v_ channel activation (*I_k_*) was determined using depolarizing test pulses from –50 to 70 mV, in 5 mV increments. The current-voltage relationships were determined according to [80]. The Clampfit version 11.0.3 (Molecular devices, San Jose, CA, USA) and OriginPro 9.0 software (Originlab, Northampton, MA, USA) were used for the analysis of all data. The data are expressed as mean ± standard error (S.E.M.) of independent experiments (*n* = 3).

#### 4.3.4. Shotgun Proteomics

Approximately 5 µg of fractions were lyophilized and dissolved into 4 µL of 50 mM NH_4_HCO_3_ pH 7.8. The sample was reduced with 2 µL of 500 mM dithiothreitol (DTT) for 40 min at 56 °C under shaking at 650 rpm. The reduced samples were alkylated for 30 min at room temperature in the dark with 3 µL of 500 mM iodoacetamide. A second step of reduction was performed with 2 µL of 500 mM dithiothreitol (DTT) for 10 min at room temperature in the dark. The samples were then digested enzymatically using trypsin in two consecutive steps: the first one at a ratio of 1:50 (trypsin:protein), incubated overnight at 37 °C, shaking at 650 rpm, the second with a ratio of 1:100 with incubation at 37 °C for 3h. Stop reactions were conducted by acidifying, with 10% TFA as the medium (final concentration). Finally, the digested samples were dried on a speed vacuum. Before the mass spectrometry analysis, the samples were suspended in 20 µL of 0.1% TFA for desalting on ZipTip pipette tips with C18 resin. The elution was performed by 20 µL of 0.1% TFA/ACN (50/50).

The LC-MS/MS analyses were performed on an Acquity M-Class UPLC (Waters) hyphenated to a Q Exactive (Thermo Scientific), in nanoelectrospray positive ion mode. The trap column was a Symmetry C18 5 μm (180 μm × 20 mm) and analytical column was a HSS T3 C18 1.8 μm (75 μm × 250 mm) (Waters, Corp., Milford, CT, USA). The samples were loaded at 20 μL/min on the trap column in 98% solvent A for 3 min and subsequently separated on the analytical column at a flow rate of 600 nL/min with the following linear gradient: initial conditions 98% A; 5 min 93% A; 30 min 60% A; 33 min 15% A, which was maintained for 5 min before the column was reconditioned in initial conditions. Solvent A was 0.1% formic acid in water and solvent B was 0.1% formic acid in acetonitrile. The total run time was 60 min. The mass spectrometer method is a TopN-MSMS method where N was set to 12, meaning that the spectrometer acquires one Full MS spectrum, selects the 12 most intense peaks in this spectrum (singly charged and unassigned charge precursors excluded), and makes a Full MS2 spectrum of each of these 12 compounds. The parameters for MS spectrum acquisition are: Mass range from 400 to 1600 *m*/*z*; Resolution of 70,000; automatic gain control (AGC) target of 3 × 10^6^ or Maximum injection time of 200 ms. The parameters for MS2 spectrum acquisition are: Isolation Window of 2.0 *m*/*z*; Normalized Collision energy (NCE) of 27; Resolution of 17,500; AGC target of 1 × 10^5^ or Maximum injection time of 50 ms. The main parameters for Q Exactive tune were: spray voltage of 2.3 kV, capillary temperature of 270 °C and S-Lens RF level of 50.0.

Protein identification by automated de novo sequencing was performed using the software Peaks Studio X+, with a database created by the deposits related to the “Theridiidae” family in the UniProt repository, downloaded in October 2021 (18,128 sequences) [80]. Carbamidomethylation was set as fixed modification, and oxidation (M) was set as variable modification, with maximum missed cleavages at 3. Parent mass and fragment mass error tolerance were set at 5 ppm and 0.015 Da, respectively. A false discovery rate (FDR) of 1% and unique peptide ≥1 were used to filter out inaccurate proteins for the PEAKS and SPIDER search algorithms and “De novo only” analysis with a −10lgP > 20 for the database match with high in confidence.

## 5. Conclusions

Although the venom of *Latrodectus* has been studied previously, this is the first study that provides a broad view of the *L. geometricus* venom composition. The molecular intricacy of the venom was uncovered through next-generation sequencing in this study. The findings revealed that toxin and non-toxin activities work in tandem, implying that these toxins play an essential role in prey envenomation. U24-ctenitoxin-Pn1a is able to modulate the insect ion channels, suggesting that this molecule may be used as a source of insecticidal peptides. However, additional research into the mode of action of this compound is required. This study also provided an overview of the molecular diversity of the *L. geometricus* venom, which can be used as a reference for other spider species, as well as provide knowledge for the development of sodium channel targeting insecticides.

## Figures and Tables

**Table 1:** Description of the output sequencing data.

	*Latrodectus geometricus* Venom Gland Sample
Total Raw Reads	85,709,012
Total Clean Reads	67,659,540
Total Clean Nucleotide (nt)	6,765,954,000
Q20 (%) ^1^	95.15

^1^ Q20 percentage is the proportion of nucleotides with a quality value larger than 20 in reads.

**Table 2:** Summary statistics of assembly for *L. geometricus* venom gland transcriptome.

Assembly	Number	Total Length (nt)	Mean Length (nt)	N50 ^1^ (nt)	GC% ^2^
Contig	47,379	23,845,768	503	681	34.34
Unigene	32,505	18,752,035	576	770	34.67

^1^ N50 is the median unigene size. ^2.^ GC percentage is the proportion of guanidine and cytosine nucleotides among total nucleotides.

**Table 3:** Main venom components identified in the spider venoms.

Venom Components	Functions	*Latrodectus hesperus* [67]	*Latrodectus tredecimguttatus* [34]	*Latrodectus geometricus*	*Steatoda nobilis* [61]
α-Latrotoxin	Targeting vertebrates, form Ca^2+^ channel on presynaptic neurons, massive neurotransmitter release	√	√	√	√
α, δ-Latroinsectotoxins	Targeting insects, form Ca^2+^ channel on presynaptic neurons, massive neurotransmitter release	√	√	√	√
α-Latrocrustotoxin	Targeting crustaceans, form Ca^2+^ channel on presynaptic neurons, massive neurotransmitter release	√	√	√	√
Ctenitoxin	Protease inhibitors and ion channel blocker	N/A	√	√	√
Agatotoxin	Ion channel blockers	N/A	N/A	√	N/A
Centipede toxin	Voltage-gated ion channel inhibitor	N/A	N/A	√	N/A
Lycotoxin	Calcium channel inhibitor/ potential insecticides	N/A	√	√	N/A
Scorpion toxin like	Unknown function	N/A	√	√	N/A
Cysteine Rich Secretory Protein (CRISPs)	Calcium channel inhibitor	√	N/A	√	√
Metalloprotease	Tissue damage/enhancing latrotoxins spreading	√	N/A	√	√
Serine protease	Tissue damage/enhancing latrotoxins spreading	√	N/A	√	√
Hyaluronidase	Spreading factor/enhancing latrotoxins spreading	√	N/A	√	√
Chitinase	Arthropod exoskeletons digestion	√	N/A	√	√
Inhibitor cystine knot (ICK)	Ion channel function alternation	√	N/A	√	√
Lipase	Breaking down fat/enhancing latrotoxins spreading	√	N/A	√	√
Phospholipase	Phospholipids degradation/enhancing latrotoxins spreading	N/A	N/A	√	√
Defensins	Antibacterial activity	N/A	N/A	√	N/A
Translationally Controlled Tumor Proteins (TCTPs)	Inducing the local inflammatory reaction	N/A	N/A	√	N/A
Leucine-rich	Pathogen infection protection	N/A	N/A	√	N/A

Check mark (√) indicates the proteins found in each spider species.

## Data Availability

Data is available from the authors.

## Supplementary Materials

Figure S1: The annotated sequence alignment of the toxin cluster from *Latrodectus geometricus* venom gland, File S1: hmm profile of the genus *Latrodectus*, Files S2 and S3: Shotgun proteomics of *Latrodectus geometricus*, Table S1: The statistic of annotation databases of *Latrodectus geometricus* venom gland, Table S2: FPKM statistics of ion channel in *Latrodectus geometricus* venom gland, Table S3: Blastn/x of 20 most expressed unigenes of *Latrodectus geometricus* venom gland, Table S4: Clustering classification of *Latrodectus geometricus* venom gland.

## Sample Availability

Samples of the compounds are not available from the authors.
