# Supplementary material for: De Novo Transcriptome Analysis of the Venom of Latrodectus geometricus with the Discovery of an Insect-Selective Na Channel Modulator
Source: Molecules. 2021 Dec 22;27(1):47. doi: 10.3390/molecules27010047 (PMC8746590; doi:10.3390/molecules27010047)
Supplement: Supplementary file 1 [file molecules-27-00047-s001.zip › Supplementary materials .pdf]

Supplementary materials Figure S1 The annotated sequence alignment of the toxin cluster from *Latrodectus geometricus* venom gland.

```
                                                             Toxin 9 domain
U8-agatoxin-Ao1 ---------------------------MKSLLFVTIAVYFVAQAVTANLLSNFLGSSLID
GBJM01003319.1. LSSAQKSSDLSAVNALLVQYKMM----KQYLLLATLVTFLMVQTMAAVPYPPALDRNLSE
GBJM01112589.1. M-------------------KMMTCLPLKTLLLVALLLFLLAQFGAMGDAYAMRGFSLAD
GBJM01112590.1. -------------------------------MLIFLLYVCVCERDVLIVNVPSLGFSLAD
GBJM01054266.1. ------------------------------------------------------------
GBJM01019804.1. --------------------------------------------QVLIVS-PFMATSDAD
                             Toxin 9 domain
U8-agatoxin-Ao1 ---DDKGNMHK----LYKRSEDQCIGRSCTCDTSSTSCCPYAACRCNLWKTSCKCQRTG-
GBJM01003319.1. DYSD--NNLEN----IMRTDKRACIRRGGGCDGKPNDCCANSSCRCNLWGTNCRCERAGL
GBJM01112589.1. SSLDPEPNYENLARYLMSTRKRSCIRRGGSCDHRPSDCCYSSSCRCNLWGTNCRCQRVGI
GBJM01112590.1. SSLDPEPNYENLARYLMSTRKRSCIRRGGSCDHRPSDCCYSSSCRCNLWGTNCRCQRVGI
GBJM01054266.1. -----------VDVVLLPFDKRSCIRRGGSCDHRPNDCCFSSACRCNLWGTNCRCQRAGL
GBJM01019804.1. --REILADYPDVTRYLYYNRKRSCIKRGGSCDHRPNDCCYNSSCRCNLWGTNCRCQRMGL
                :    :  ** *.  **  ...**  ::****** *.*:*:* *

U8-agatoxin-Ao1 -RKWATPCKEIYSPN
GBJM01003319.1. FQQWGR--------K
GBJM01112589.1. FQKWGK---------
GBJM01112590.1. FQKWGK---------
GBJM01054266.1. FQRWGR--------K
GBJM01019804.1. FQKWGK---------
                ::*.
```

Figure S1. The sequence alignment of the agatotoxin family from *Latrodectus geometricus* venom gland. Alignment was performed with the Multiple alignment program for amino acid or nucleotide sequences (MAFFT) program, using U8-agatoxin-Ao1a from the spider *Agelena orientalis* (UniProt ID: Q5Y4U4.1) as a reference. The blue line marks the Toxin 9 domain.

```
Alpha-latrocrustotoxin VSIFIFHFSANILVRNSEMKGKRVISKREMSKADQCTFLSYQSVAYGTLGDVAGDVSSIE
GBJM01062411           ------------------------------------------------------------

Alpha-latrocrustotoxin GADLVATPIAAGGHLAKGATDAAMIAMDCSSIPFDEIKQQLNQRFNEVDKKLQKGAEALE
GBJM01062411           -----------GGQI---------------------------------------------
                                  **::
Alpha-latrocrustotoxin NVTELAEKTYSSVEKMRVEMREGFNHVIATIENANTKQIITGINQIIQYFNDERENINNR
GBJM01062411           ------------------------------------------------------------

Alpha-latrocrustotoxin QKEDYVAKLQEPASGNFLLYLRKSRTSEDGSLHSLLFKIINQELAIPNNAADNNAIRALF
GBJM01062411           ------------------------------------------------------------

Alpha-latrocrustotoxin ALFYGTQTFISIMFYLVKQYSYLADYHYQNGNLAEFNSNFDHMKTVFQDFKFTLIGINTS
GBJM01062411           ------------------------------------------------------------

Alpha-latrocrustotoxin NSKPLVNTVLSIIEDVKNKRFIRNLRSNLYQKIIKSTKSLLDLREKITKMDLPIIEDTPK
GBJM01062411           ------------------------------------------------------------

Alpha-latrocrustotoxin SSVLINFREKSSSVPRIETPILKWTPGTVVKYAIQYEQDGKYSKISKWSNPITVQRLANP
GBJM01062411           ------------------------------------------------------------

Alpha-latrocrustotoxin YITIDKDRRNRLVFRQFGNEKPELISILDSSQNEFRDIHRDLYNAAQMPYKETALGICRK
GBJM01062411           ---------DRL------------------------------------------------
                                :**
Alpha-latrocrustotoxin LIDSGAQVGASFEMGRKSIHASATAGNDDVARLLLAKNNGLLNVPDKNGYTPLHIASERK
GBJM01062411           ------------------------------------------------------------

Alpha-latrocrustotoxin NNDFVKFLLEKGADVNVRTFANELTPLHLAARQDFTIIVKTLMEKRGIDVNAKERAGFTP
GBJM01062411           ------------------------------------------------------------

Alpha-latrocrustotoxin LHLSITSNSRAARTLINETPAGINIKSNSGLTPLHLAVLQNNLSAAKVLVKSNKKVKLNE
GBJM01062411           ------------------------------------------------------------

Alpha-latrocrustotoxin MDNNGMTPLHYASMLGNLEFVKYFTSEQGIDVNAKTKVKNWTPLHLAILFKKFDVAQSLL
GBJM01062411           ----------------------------------------W-------------------
                                                               *
Alpha-latrocrustotoxin QVRNIDISTRADQAITPLHLAAATGNSQIVKTILNSGAVVDQETANGFTALHLAIMNPNT
GBJM01062411           ------------------------------------------------------------

Alpha-latrocrustotoxin ETPQFLIAKGANINAKTNDGSTPLHFAAALGKTNIFQLLMDKGANIKAENLINQMPIHEA
GBJM01062411           ------------------------------------------------------------

Alpha-latrocrustotoxin VVNGHLAIVKMLIEQDSSLMNAKNMRDEYPFYLAAEKRYKDVFNYLESKGADVNEKNNDG
GBJM01062411           ------------------------------------------------------------

Alpha-latrocrustotoxin NTLLHLFSINGEVEVVQFLIQNGADFRLRNKERKSFFDLAVEFGHAGIVGYAIEENKVDL
GBJM01062411           ------------------------------------------------------------

Alpha-latrocrustotoxin QEPYRGKTILYHAICDSVKYDRIEVVRYFVETLNEDQCSPLQEAAAYAHLDLVKYFVQER
GBJM01062411           ------------------------------------------------------------

Alpha-latrocrustotoxin GINPTAFNNDNQVSPLCIAIVGAPCGFVKSCDTPERLDVVEYLVDKTPDINKECDTQQST
GBJM01062411           --------------------------------------------------------RNVS
                                                  ANK domain                :: :
Alpha-latrocrustotoxin PVSSAVYGNKVSILNYLIRNGADPNKKV---RGDPPLFIAAMIGQYDIVKSLVEQHKIDV
GBJM01062411           PVSCAVYERAYDILEYLVKKGANVNIRTLDNKMEPPLFAACRLGHLEAVHILLQSSNLDI
```

Figure S2. The sequence alignment of the alpha-LCT-Lt1a family belonging to ANK superfamily from *Latrodectus geometricus* venom gland. Alignment was performed with the Multiple alignment program for amino acid or nucleotide sequences (MAFFT) program, using alpha-latrocrustatoxin-Lt1a from spider *Latrodectus tredecimguttatus* (UniProt ID: Q9XZC0.2) as a reference. This family presents only the matched sequences.

```
Alpha-latroinsectotoxin-Lt1a IEKLNE--SECN-PLHEAAAYAHLDLVKYFVQERGINPAEFNEENQASPFCITIHGA--P
GBJM01001888                 ------------------------------------------------------------
GBJM01066462                 -------------------TYAHLDLVKYFVQEKGMNPSEFNSKTPQSAFCQAILGKRTD
GBJM01062969                 ------------------------------------------------------------
GBJM01051925                 ------------------------------------------------------------
GBJM01109243                 ------------------------------------------------------------
GBJM01005762                 -------------------------------------------------------ES---
GBJM01059725                 ----------CE------------EILK--RSEADVNGV-FASHTAL----QAASQN---
GBJM01039245                 ---------------------------------------------------QSTNSE---
GBJM01090534                 ---------------------------------------------------QSTNSE---
GBJM01131353                 ----------C-----------LAELLK--HENTNINWLTHESETALLLTCKRRQGS---
GBJM01128973                 ------------------------------------------------------------
GBJM01023374                 DSKFNRSPSSCS----------RDGLLK--R-----------------------------
GBJM01058469                 ------------------------------------------------------------
GBJM01116720                 ----------CHIETYIASIPKGENILS-VQSADGYNLL------------QRAIGF---
GBJM01049106                 ----------CP------------------------------------------------

                                                                        ANK domain
Alpha-latroinsectotoxin-Lt1a CGYSLDCDTPDRLEVVEYLSDKIPDINGKCDVQENTPITVAI-FANKVSILNYLVGIGAD
GBJM01001888                 ----------------------------------------------KASILNYLVGIGAD
GBJM01066462                 CEQSIKCDTPERLEVVEYLVDKIPDINETCDDAKNTPISMAV-FSNKVSILNYLVRLGAD
GBJM01062969                 -------------------------------------LRMQV-DQSKMSILNYLVRLGDD
GBJM01051925                 -------------------------------------------DQSKMSILNYLVRLGDD
GBJM01109243                 -------------------------------------------KCGKVSILNYLVRLGAD
GBJM01005762                 ----------SSLKIAKYLVSKGADIKASPQF-----LYAAV-HAPKVSILNYLVRLGAN
GBJM01059725                 ----------GHMEVIKILLRHNADV-EIEDKDGDRAVHHAA-FGDKVSILNYLVRLGAD
GBJM01039245                 ----------KFFELIKLHPEFSSQMSEIANKHGDNIVHLSA-KCGKVSILNYLVRLGAD
GBJM01090534                 ----------KFFQLVKLHPEFSSQMSEIANKHGDNIVHLSA-KCGKVSILNYLVRLGAD
GBJM01131353                 ----------EANDFVKLLLDHGADP-NIPDNEADSPLLEAL-RNDKVSILNYLVRLGAD
GBJM01128973                 -------------------------------------FHHAV-KTNKVSILNYLVRLGAS
GBJM01023374                 -------------------------------------MHIAV-RNGKVSILNYLVRLGAE
GBJM01058469                 -------------------------------------MVKAI-YEKKVSILNYLVRLGAD
GBJM01116720                 ----------NCVELVKWILNRGCDV---NRGACSLPLHIAA-FHEKVSILNYLVRHGAR
GBJM01049106                 -----------------------------------TILHHALLRYWEKKVLNYLVRHGAC
                                                                           :.::******.**.
                                                 ANK domain
Alpha-latroinsectotoxin-Lt1a -------VDGDPPLYIAAR-QGRFEIVRCLIEVHKVDINTRNK----ERFTALH-AAARN
GBJM01001888                 -------VNGDPPLYIAAR-HGQIDIVRCLIEVHKIDINTRNK----EQFTALH-AAAVN
GBJM01066462                 -------ING--------------------------------------------------
GBJM01062969                 -------VSR--------------------------------------------------
GBJM01051925                 ------------------------------------------------------------
GBJM01109243                 -----------------------------------ASLN--------RGQTYLH-LAVLS
GBJM01005762                 -------FFNRPLVHYAAE-EC-FDCLEYAVERNLMDVNAKDD----RGNTACHKLVMYH
GBJM01059725                 -------KRRQTPLHIAVN-KGHVGVVRSILELG-CRPSLQDS----EGDTPLH-DAISK
GBJM01039245                 -------NDGKTPLHIAAH-VSQLECVEYLLSCN-VPVDALKR----SDWTPLM-LACTK
GBJM01090534                 -------NDGKTPLHIAAH-VSQLECVEYLLSCN-VPVDALKR----SDWTPLM-LACTK
GBJM01131353                 -------------------------------------VNVADC----SGWSPLH-EAATK
GBJM01128973                 -------NNGRPALLQATY-QGNERMVKLLLEYQ-ANPNAQNI----VGETALH-NAFSS
GBJM01023374                 -------VNN---LHKEVL-LNNRQPLSSF-----KPVSVLKKPFGNARITPLH-CAAIN
GBJM01058469                 -------MCHKCPIMICIE-ANSLKIGELLISEG-CSLSLKTT----MGNTPLH-IAVEL
GBJM01116720                 EDNWLPWQQKRPLLHLACE-RGAWNCVRYLVAERSDEIN-----QCYDEYYPIH-QAAIQ
GBJM01049106                 -------HVDMTPLQFALNYKHNAELIKILLNYG-ASVNVCSL----CGRTPLH-IAIVN
                                                 ANK domain
Alpha-latroinsectotoxin-Lt1a D-FMD--VVKYLVRQGADVNAKGI-DDLRPIDIAGEK------AKAYLQSS---------
GBJM01001888                 D-HLD--IVKYLVRNGADVNAKGT-DDLKPIDIAGEK------AKAYLQTSRM-------
GBJM01066462                 ------------------------------------------------------------
GBJM01062969                 ------------------------------------------------------------
GBJM01051925                 ----------VLIDANI----------------IGK-----------EEIF---------
GBJM01109243                 N-QLD--LVKLLIEKGIYIYVADN-EGHFPIWYASDK-----------EIYRYLNEKMSS
GBJM01005762                 N-PLGPRVIEYLCSHGVDKTIRNS-EGKTAYEIAQDL--------HTQSLYECLKPDASK
GBJM01059725                 K-RDD--MLTLLLDNGADICLTNN-NGFNALHHAALR--------GNPSAMRILLSKLPR
GBJM01039245                 S-NLD--IIKTLLKHGANVNYRNK-DGWTAFHIACRE--------GHINIIKLLLDHDPS
GBJM01090534                 S-NLD--IIKTLLKHGANVNYRNK-DGWTAFHIACRE--------GHINIIKLLLDHDPS
GBJM01131353                 K-NPV--FVNWLLRNGANIDVRDE-CGMTPLFSAAQH--------GNEQCLKALLSAAQQ
GBJM01128973                 K-TFSKKIVKALLERGANVNVRETLNGYTPVHIAAKMMSSKLSKSSQEDILETLTEMCER
GBJM01023374                 P-NPQ--YLTALLSSAPEYNIGDE-NGWRPIHYAAVC--------KSTGPLELLIARGIS
GBJM01058469                 S-NYD--FVKLLLNNRADVTSVDD-SGKTALHLAAQM--------NSLRMVEMLMEKATP
GBJM01116720                 D--LK--FLGLLIQCGADVLVRTATQQMTVLHVVLLSG--RKSAEETLNTIKMLLDHGCR
GBJM01049106                 NYNVD--VIEKLLKCGASVNAEDN-FGMNALNLALSS------KYCNMAIVEKLLQYGAE
                                                                ANK domain
Alpha-latroinsectotoxin-Lt1a HGISMSARTN----------DKL---TQQIS-----SKGTR------SDSNSTEGKMHSE
GBJM01001888                 NGISMSARTN----------SQLN-STQQIY-----SKRIR------SEFNSREDRMNSE
GBJM01066462                 -GISMSART-----------DKL---TQQIS-----SKGTR-------------------
GBJM01062969                 NGISMSARTN----------SQLN-STQQIY-----SKRIR------SEFNSREDRMNSE
GBJM01051925                 -GISMSARTNSFHEDVNKITIPQVSRSALATI----------------RLQETKSAVD--
GBJM01109243                 KDLNYDSTK---------------------------AKVIDQNYRVFARLKSNESSTKRS
GBJM01005762                 SEIKNRKMSSFHEDVNKITIPQVSRSALATI-----RDTPHFTF-PESRLHSNMN-----
GBJM01059725                 DGISMSARTNS----------ALHLAALNNH-----VEVAE------LLVQQGRANMNLQ
GBJM01039245                 -GISMSARTN-----------PLHTAALHGC-----IETVN------LILTTDH-DIDVS
GBJM01090534                 RGISMSART------------PLHTAALHGC-----IETVN------LILTTDH-DIDVS
GBJM01131353                 KGISMSART------------PLMIAAQQGF-----VNCVK------LLLAHGA-DPNLT
GBJM01128973                 NGISMSART------------ALMCAINKGH-----VRQAE------VLIKNGA-GVNIK
GBJM01023374                 EEPESEGNT------------PLHLAAMTGR-----SHNVD------LILKH--------
GBJM01058469                 NGISMSART-----------TPLLGALSNHNL---SLNTIN------MLLSAGA-NINQI
GBJM01116720                 PGISMSART------------ALACVLRHIRDWEFRYELLE------MLLQNGG-NPNSV
GBJM01049106                 SGISMSART------------PLILAVKLNL-----NKAIK------LLIKYNA-SARES
```

Figure S3. The sequence alignment of the alpha-LIT-Lt1a family belonging to ANK superfamily from *Latrodectus geometricus* venom gland. Alignment was performed with the Multiple alignment program for amino acid or nucleotide sequences (MAFFT) program, using alpha-latroinsectotoxin-Lt1a from the spider *Latrodectus tredecimguttatus* (UniProt ID: Q02989.1) as a reference. The green line marks the ankyrin repeat (ANK) domain.

```
Alpha-latrotoxin-Lh1a MFFLLEQYSFLANHYYEKGDLEKYDEYFNSLNNVFLDFKSSLVGTGTSNNEGLLDRVLQV
GBJM01062969          ----------------------------------------------------LLDKVIDT
GBJM01051925          --------------------------------------------------------VLID
GBJM01116664          ------------------------------------------------------------
GBJM01109243          ------------------------------------------------------------
GBJM01005762          ------------------------------------------------------------

Alpha-latrotoxin-Lh1a LVTVKNSEFLGLEKNGVNEMLNEKINLFNKIKVEIEGKQRMTLSETPENFAQISFDKDIT
GBJM01062969          LNTVKNLEFLRLQNDELIQLIKKNIDTFTENTVTILELILPIISETPRNYRQINFKKDVS
GBJM01051925          ANIIGKEEIFRLQ-----------------------------------------------
GBJM01116664          ----GKEEIFRLK-----------------------------------------------
GBJM01109243          ----PAEEIFRLE-----------------------------------------------
GBJM01005762          ----IPEEIFRLQ-----------------------------------------------
                                              ::******:
Alpha-latrotoxin-Lh1a TPIGDWRDGREVRYAVQYASETLFSKISHWSDPVGVREKACPTLRMPVDQTRRNILVFRK
GBJM01062969          TPLGRWIKGTPVRYAVQYENNDHYSRISSWSEPTIIQKKACPSLRMQVDQSKMNRLIFRK
GBJM01051925          ------------------------------------------------------------
GBJM01116664          ------------------------------------------------------------
GBJM01109243          ------------------------------------------------------------
GBJM01005762          ------------KYLV--------------------------------------------

Alpha-latrotoxin-Lh1a FDSSKPQLVGEITPYQSNFIDIDRDLYNTANNPDSAVGFKEFTKLNYDGANIRATFEQGR
GBJM01062969          IGDDKPQLVSR-------------------------------------------------
GBJM01051925          ------------------------------------------------------------
GBJM01116664          ------------------------------------------------------------
GBJM01109243          ------------------------------------------------------------
GBJM01005762          ------------------------------------------------------------

Alpha-latrotoxin-Lh1a VDFRRKNKLGATPFLTAVSENAFDIAEYLIRENRQDIDINEQNVDKETALH-LAVYYKNL
GBJM01062969          ------------------------------------------------------------
GBJM01051925          ------------------------------------------------------------
GBJM01116664          ------------------------------------------------------------
GBJM01109243          ---------------------------------------------GQTYLH-LAVLSNQL
GBJM01005762          ------------PLVHYAAEECFDCLEYAVERNL--MDVNAKDDRGNTACHKLVMYHNPL
                                                 ANK domain
Alpha-latrotoxin-Lh1a --QMIKLLVKYGIDMTIRNAYDKTALDIATDLKNSNIVEYLKTKSGKF-RREYKSS----
GBJM01062969          ------------------------------------------------------------
GBJM01051925          ------------------------------------------------------------
GBJM01116664          ------------------------------------------------------------
GBJM01109243          --DLVKLLIEKGIYIYVADNEGHFPIWYASD---KEIYRYLNEKMSSL--RHIREANFRR
GBJM01005762          GPRVIEYLCSHGVDKTIRNSEGKTAYEIAQDLHTQSLYECLKPDASKILKRNINKSFIRK
                                                 ANK domain
Alpha-latrotoxin-Lh1a --YGEHSLLQTNKISSFIDGKN---------------IEHDHPQFINA-DNESSQLFSDT
GBJM01062969          ------------------------------------------------------------
GBJM01051925          ----------------------------------------------------------ET
GBJM01116664          ------------------------------------------------------------
GBJM01109243          ---------ENSKL--FYKDLN-------YDSTKAKVIDQNYRVFARLKSNESSTKRSNV
GBJM01005762          KEYPRYSEIKNRKMSSFHEDVNKITIPQVSRSALATIRDTPHFTFPE----------SRL
                                                 ANK domain
Alpha-latrotoxin-Lh1a A-SNIDVIGPLLLIDVLIRYFSKQGYISKESDSASDGITQAAALSITEKFEDVLNSLHNE
GBJM01062969          ------------------------------------------------KFE---------
GBJM01051925          K-SAVDITGFLMLLDVIIRYLTNQKYIGEGNELALEAAIQATALKITENFENLLDI---L
GBJM01116664          -------------------------------------------LKAVEKLEELIALFSNL
GBJM01109243          GVQSAGVIDTLLLLNVIVRYFTNQKYIG-GSQSSSDADGLATALKITKKLERYLTSPTNF
GBJM01005762          H-SNMNFNHNLFLIDFFLKMLTGEKYKHVEKTDATEFEVKAEALKIIEKFESMLKSKSNA
                                                 ANK domain
                                                                       ***
Alpha-latrotoxin-Lh1a SAKEQVDLAEVHGKVYAALKSGRNSQIHPILCSSLKSISTLKPEDMEKLGSVIMNSHS--
GBJM01062969          ----QVDL----------------------------------------------------
GBJM01051925          PTFSGVDFCEVHRNVYKALKN------RPIICASMRTFSTLTSEKK--------------
GBJM01116664          PVDEEIDLAEVHANIYKALKSGKNSKLHEIVCSYVDTIMSLNPEQKEKLVTAFDNTFPSI
GBJM01109243          PV-EEIDFSEVHKEIHKKIIGDKGNQIHILLCSTVNFFSLLKPKEIEDLISEFVSVQ--L
GBJM01005762          PIEEYIDLPKVYSEIYKAIKSGNANEIPNILYSHGKYFSDLNSNEISKLFS---------
```

Figure S4. The sequence alignment of the alpha-LTX-Lh1a family belonging to ANK superfamily from *Latrodectus geometricus* venom gland. Alignment was performed with the Multiple alignment program for amino acid or nucleotide sequences (MAFFT) program, using alpha-latrotoxin-Lh1a from spider *Latrodectus hasseltii* (UniProt ID: G0LXV8.2) as a reference. The green line presents the several ankyrin repeat (ANK) domain in this family.

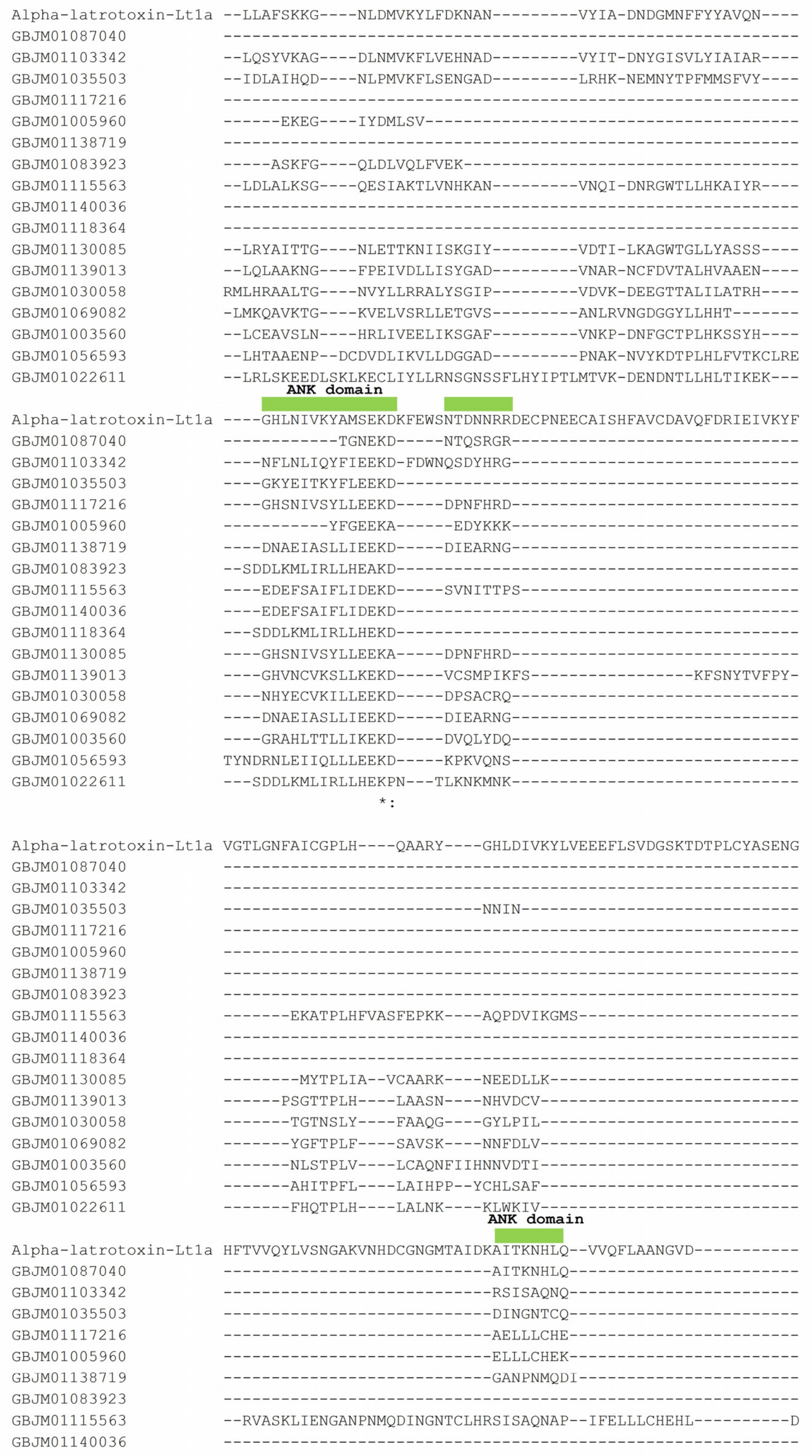

Figure S5. The sequence alignment of the alpha-LTX-Lt1a family 1 belonging to ANK superfamily from *Latrodectus geometricus* venom gland. Alignment was performed with the Multiple alignment program for amino acid or nucleotide sequences (MAFFT) program, using alpha-latrotoxin-Lt1a from spider *Latrodectus tredecimguttatus* (UniProt ID: P23631.2) as a reference. The green line presents the several ankyrin repeat (ANK) domain in this family.

```
                                                       ANK domain
GBJM01118364        ------------------------------------------------------------
GBJM01130085        ---CCNLLIKHGANVNAHERHLTTPLMFAAREGYYN--IAKILLDHNAN-----------
GBJM01139013        -----YELIQYGADYNAIDEYGRTSLFMAAKLGSEEC-----ILSHLKNAVGRNILSIPD
GBJM01030058        -----RLLLQYKADVRATAGDGATPYLMACQCNHLD--VIQELSSYDPE-----------
GBJM01069082        -----EVLLLKGANVHSIAPGGNNILHHAVEMKGCNKDIVSLLIKHGCS-----------
GBJM01003560        -----RVLIRAGTNVNAPNRYG--------------------------------------
GBJM01056593        -----VGLVKFGADFSVTKSNESLLEKPLIPSSSRSLDLVKIFLANEPTL----------
GBJM01022611        -----RILLLFGADPNIKDAEGNNCVHLAVKHNLPKC-LAEILTPSVSEMYKK---CSPC
                             ANK domain
Alpha-latrotoxin-Lt1a FRRKNSRGTTPFLTAVAENA-LHIAE------YLIREKRQDININEQ-NVDKDTALHLAV
GBJM01087040        ------------------------------------------------------------
GBJM01103342        ------------------------------------------------------------
GBJM01035503        INEKNGLGQTCLHLAVMFNE-WDL------------------------------------
GBJM01117216        ------------------------------------------------------------
GBJM01005960        -------------------------------------------------TNEDLNFYTKL
GBJM01138719        ------------------------------------------------------------
GBJM01083923        ------------------------------------------------------------
GBJM01115563        LEIKNAEGQTVLWFALDAG-----IGGYDDSSFAAKLVKRGSCLDAVCPLNGDTLLHLAC
GBJM01140036        ----------------------EV------------------------------------
GBJM01118364        -NAQNDEGDTILHKHVLIGN-TAV------------------------------------
GBJM01130085        PNLQDNRGKTAIAWAASSGH-GHI------------------------------------
GBJM01139013        FETSD----TPLHFAVSKGY-IEC------------------------------------
GBJM01030058        FEKKILEEPTPLYIAAENGH-LEV------------------------------------
GBJM01069082        LKFLNGLTRSPLHVALAND---EI------------------------------------
GBJM01003560        --------KIALHYAVLWGL-GEI------------------------------------
GBJM01056593        VKEKDIRQRTPLHIAVFGGASVDVIK---------ELINKGANVNET-DEEGNTPLH-QL
GBJM01022611        IDALNHDGLAPLHLSIMNKS--EIC-----------------------------------
                                                       ANK domain
Alpha-latrotoxin-Lt1a YYKNLQMIKLLIKY-GIDVT----------IRNAYDKTALDIAIDAK------FSNIVEY
GBJM01087040        ------------------------------------------------------------
GBJM01103342        ------------------------------------------------------------
GBJM01035503        -------VKLLVEK-GIYLF----------MLDYKTQLAVDYS---------KNQEMYDY
GBJM01117216        ------------------------------------------------------------
GBJM01005960        YFTSFEECLISWKQNGLDVSTLDQNNWKLCIQPWYDKSGLPIAFFTD-------DDFPSE
GBJM01138719        ------------------------------------------------------------
GBJM01083923        ---------------GIDTQ----------IRDMNGETALDLS---------GNKFIRDY
GBJM01115563        RVSN-EEAGIFLAVHGAKPN----------LTNNKGEVPLHIACLKG------LSNLTSV
GBJM01140036        -------INYLCDI-AADRT----------IKNNNNQKAYDIAKKAN------KAGKYSC
GBJM01118364        -------VNYLCSF-GADTT----------IKNNKKETAYDIAKKVN------KADMYSC
GBJM01130085        -------IRLLIEK-GGDPS----------ICDKMGQSPEDFAYENG------HTELVSM
GBJM01139013        -------VDLLLKC-GSDVS----------HKNFAGYSPLHLAVTPTVEVKNACFAITKL
GBJM01030058        -------VSYLVSR-GADIN----------RKIFDGSTPLLIACQMG------HGAVVQK
GBJM01069082        ---NWEAVKVLIEN-GVDIT----------RMDKTSCTPVDIALRNKS----CKSWMIKY
GBJM01003560        -------TDVLLKA-DSQVD----------YMDFKRKTPLFCCIRIFYD-----------
GBJM01056593        TLTTYSVIETLVKY-GADIN----------AKNVNKRTPLMECLKPRLNI--VNCTAITA
GBJM01022611        -------TKLLIKA-KANVNV---------TDGKSGKTPLQLACEQD------SKLAEYL
                                               ANK domain
Alpha-latrotoxin-Lt1a LKTK-------------------SGKFRREYK------SSYG-ERSLL-----QTNQIS-
GBJM01087040        ------------------------------------------------------------
GBJM01103342        ------------------------------------------------------------
GBJM01035503        LIEK------------------MHGRFIRSPK---IWKNNYNLNRNYLHYNSVKNNR---
GBJM01117216        ------------------------------------------------------------
GBJM01005960        LESS-------------------VKSYRRD------SESFY------L-----RDPQKY-
GBJM01138719        ------------------------------------------------------------
GBJM01083923        LLSK-------------------TYRLIRKIP---FPNKVH--NRDIV------------
GBJM01115563        LLQKGANANAQTFSPES----LAKLPFDEDEE-----EAVYQQTPLHLAILGQHEDTIQ-
GBJM01140036        L--------------------NQRKKFKRNNN---LLMGNNDDNNDLFPTKEFNRNEIR-
GBJM01118364        L--------------------KPKKYFKRSNS---LSMKNN--NNDWLSTKEFENNKDR-
GBJM01130085        FQRGNFSVENF------------EAKKSQNTE-LILANGTLGKSK---------------
GBJM01139013        LILQGYNTD-----------------------INQLDGSY-FSPLHFVCF----------
GBJM01030058        LIDLGADVNR---------------------------ERNDGCTPL--------------
GBJM01069082        LIDIGGYIENFSSMPDFESVLCIAVRNKFDVEIISLLSERFGVNPLTLSING-------G
GBJM01003560        ------------------------------------------------------------
GBJM01056593        LVELGADVD--------------------------------------LSLYDFYDNSV--
GBJM01022611        LLQPGIDVTNFSGCAPV----SKVTSFDENKE----------------------------
```

Figure S5. The sequence alignment of the alpha-LTX-Lt1a family 1 belonging to ANK superfamily from *Latrodectus geometricus* venom gland. Alignment was performed with the Multiple alignment program for amino acid or nucleotide sequences (MAFFT) program, using alpha-latrotoxin-Lt1a from spider *Latrodectus tredecimguttatus* (UniProt ID: P23631.2) as a reference. The green line presents the several ankyrin repeat (ANK) domain in this family. (cont.)

```
Alpha-latrotoxin-Lt1a GKIAGTIAAAAMTATHVASGRLDIEQTLLGCSDLPFDQIKEVLENRFNEIDRKLDSHSAA
GBJM01081561          ------------------------------------------------------------

Alpha-latrotoxin-Lt1a LEEITKLVEKSISVVEKTRKQMNKRFDEVMKSIQDAKVSPIISKINNFARYFDTEKERIR
GBJM01081561          ------------------------------------------------------------

Alpha-latrotoxin-Lt1a GLKLNDYILKLEEPNGILLHFKESRTPTDDSLQAPLFSIIEEGYAVPKSIDDELAFKVLY
GBJM01081561          ------------------------------------------------------------

Alpha-latrotoxin-Lt1a ALLYGTQTYVSVMFFLLEQYSFLANHYYEKGYLEKYDEYFNSLNNVFLDFKSSLVGTGTS
GBJM01081561          ------------------------------------------------------------

Alpha-latrotoxin-Lt1a NNEGLLDRVLQVLMTVKNSEFLGLEKNGVDEMLNEKINLFNKIKEEIEGKQKMTLSETPE
GBJM01081561          --------------------------------LNKKIDFFNKIKDDIRDMEPSISTRTPE
                                                      **:**::*****::*.. :    :.***

Alpha-latrotoxin-Lt1a NFAQISFDK------------DITTPIGDWRDGREVRYAVQYASETLFSKISHWSDPVSV
GBJM01081561          NYVHISFDTKIPVPIGEWEKHDITTPIGDWRDGREVRYR---------------------
                      *:.:****.            *****************

Alpha-latrotoxin-Lt1a REKACPTLRMPVDQTRRNVLVFRKFDSSKPQLVGEITPYLSNFIDIDRDLYNAASNPDSA
GBJM01081561          --------------------IFRKFQDSNSQLVGSVEPYQQEFVDINRDLYNAAENPEKE
                                          :****:.*:.****.: ** .:*:**:*******.**:.

Alpha-latrotoxin-Lt1a VGFKEFTKLNYDGANIRATFDHGRTVFHAAAKSGNDKIMFGLTFLAKSTELNQPDKKGYT
GBJM01081561          TGFKEANILLNKGADFTVGFEKGRKVLHAAAKSGNTEIALGI-LLKSSKEINVGDEDGYT
                      .**** . *  .**:: . *::**.*:******** :* :*: :* .*.*:*  *:.***

Alpha-latrotoxin-Lt1a PIHVAADSGNAGIVNLLIQRGVSINSKTYHFLQTPLHLAAQRGFVTTFQRLMESPEININ
GBJM01081561          PMHVAVDTGNSGFVKFLINHGGLVNTKTKREQLTPLHLAARRGFLRTFERLMQKADIKIN
                      *:***.*:**:*:*::**::*  :*:** :   *******:***: **:***:..:*:**

Alpha-latrotoxin-Lt1a ERDKDGFTPLHYAIRGGERILEAFLNQISIDVNAKSNTG-LTPFHLAIIKNDWPVASTLL
GBJM01081561          ERNKEGFTPFHYAVQGGNFMVKAFMQNEKFEMNPKSTKKELTPFHMAVINNDFNVTQTLM
                      **:*:****:***::**: :::**::: .:::*.**..  *****:*:*:**: *:.**:

Alpha-latrotoxin-Lt1a GSKKVDINAVDENNITALHYAAILGYLETTKQLINLKEINANVVSSPGLLSALHYAILYK
GBJM01081561          ESERIAINAGDKNNMTPLHYASMLGHSVIANYLLSSKKINPNILTNDNKWSPLHFAICFK
                      *::: *** *:**:*.****::**:    :: *:. *:**.*:::. .  *.**:** :*

Alpha-latrotoxin-Lt1a HDDVASFLMRSSNVNVNLKALGGITPLHLAVIQGRKQILSLMFDIGVNIEQKTDEKYTPL
GBJM01081561          HDEIALKLLNTPDINNDIKVDGGSTPLHFAVTFGRKTVVLELIKNKSNLEAKEKDGHTPL
                      **::*  *:.:.::* ::*. ** ****:**  *** ::  ::.   *:* * .: :***

Alpha-latrotoxin-Lt1a HLAAMSKYPELIQILLDQGSNFEAKTNSGATPLHLATFKGKSQAALILLNNEVNWRDTDE
GBJM01081561          HLSAMRNDPEILSILLNNGANIEAKSANDSTPLHLAIFNGKSKTALVLIDKGAKLTVTDK
                      **:** : **::.***::*:*:***: ..:****** *:***::**:*::: .:   **:

Alpha-latrotoxin-Lt1a NGQMPIHGAAMTGLLDVAQAIISIDATVVDIEDKNSDTPLNLAAQNSHIDVIKYFIDQGA
GBJM01081561          DGRAPIHGAVLNNQFEVVQKLLDRDPSVIDMKDDNSNTPLNIASQNFYTAIVKYLIKKHA
                      :*: *****.:.. ::*.* ::. *.:*:*::*.**:****:*:** :  ::**:*.: *
                                                 ANK domain
Alpha-latrotoxin-Lt1a DINTRNKKGLAPLLAFSKKGNLDMVKYLFDKNANVYIADNDGMNFFYYAVQNGHLNIVKY
GBJM01081561          HINTKNREGISPLIVFSKSGDLNMVEYLINNGADVYTADNSGNNFIYHAIKNGHFNIVQY
                      .***:*::*::**:.***.*:*:**:**:::.*:** ***.* **:*:*::***:***:*
                                                 ANK domain
Alpha-latrotoxin-Lt1a AMSEKDKFEWSNTDNNRRDECPNEECAISHFAVCDAVQFDRIEIVKYFVGTLGNFAICGP
GBJM01081561          ASKLKEKIDWKKRDYNREEYC-ESICTIEYFALCDSVKYGKSDIVEYFIGEL-NINLCHP
                      * . *:*::*.: * **.: * :. *:* :**:**:*::.: :**:**:* * *: :* *
                                                 ANK domain
Alpha-latrotoxin-Lt1a LHQAARYGHLDIVKYLVEEEFL----SVDGSKTDTPLCYASENGHFTVVQYLVSNGAKVN
GBJM01081561          LSNAVIFGWLNIVKYLVEQRGMNPNPTVEYPDIDQGADIAAKYGHLSIVQYLVVQKANLQ
                      * :*. :* *:*******:. :    :*:  .  *     *:: **::***** : *:::
                                                 ANK domain
Alpha-latrotoxin-Lt1a HDCGNGMTAIDKAITKNHLQVVQFLAANGVDFRRKNSRGTTPFLTAVAENALHIAEYLIR
GBJM01081561          AICGFEV-AIDLAIHYGHQLIVKFLAENGANLKRKNVYRMSPLGSAFFYKEFEIAKYFIE
                      **  : *** **  .*  :*:*** **.::.***    :*: :*.  : :.**:*:*.
                                                 ANK domain
Alpha-latrotoxin-Lt1a EKRQDININEQNVDKDTALHLAVYYKNLQMIKLLIKYGIDVTIRNAYDKTALDIAIDAKF
GBJM01081561          EKTDDIDINEKDVVNNTPLMVSVYLDRLDLVKSLVENGANVTARNNFDETALDIAKSQGK
                      ** :**:***::* ::*.* ::** ..*:::* *:: * :** ** :*:****** .

Alpha-latrotoxin-Lt1a SNIVEYLKTKSGKFRREYKSS----YGERSLLQTNQISNFIDRKNIEHDHPLFINADNES
GBJM01081561          KEIADYLQSKSLRFVQENIGSNEHNFYDQSQLNSNKGIKYMNHKYAIEKEELKQGYNNLN
                      .:*.:**::** :* :*   .*    : ::* *::*:  :::::*   ... *  . :* .

Alpha-latrotoxin-Lt1a SELFSKTASNIDVIGTLLLIDVLIRYFSKQGYISKESDSASDGITQAAALSITEKFEDVL
GBJM01081561          NQ-------NFDIVGTLMLLDVIIRYFTNQKYIGAGNKSLSEAEMQAIALNITEKFEALS
                      .:       *:*::***:*:**:****::* **.  ..* *:.  ** **.****** :

Alpha-latrotoxin-Lt1a NSLHNESAKEQVDLAEVHGKVYAALKSGRNSQIHQILCSSLNSISTLKPEDMEKLESVIM
GBJM01081561          DFFQNLPEEDQIDFSEVHKNIYKSLKSGNTNQIPKILCSSVKTFSAMTLDEIENFLPEFM
                      : ::* . ::*:*::*** ::* :****...** :*****:::*::. :::*:: . :*

Alpha-latrotoxin-Lt1a NSHSSVSLPEVTDSANEAYGETLHLFGESCLHSDGILTKKL
GBJM01081561          NTYS----PEVSTFTAAVAEKTLHSLSKYCL------AIET
                      *::*    ***:  :  .  :*** :.: **      : :
```

Figure S6. The sequence alignment of the alpha-LTX-Lt1a family 2 belonging to ANK superfamily from *Latrodectus geometricus* venom gland. Alignment was performed with the Multiple alignment program for amino acid or nucleotide sequences (MAFFT) program, using alpha-latrotoxin-Lt1a from spider *Latrodectus tredecimguttatus* (UniProt ID: P23631.2) as a reference. The green line presents the ankyrin repeat (ANK) domain in this family.

```
Delta-latroinsectotoxin-Lt1a GNACPTIKVRVDPKKRNRLIFRKFNSGKPQFAGTMTHSQTNFKDIHRD-------LYDAA
GBJM01109678                 -----------------RLIFRKFN------------------------------LYDAA
GBJM01091322                 -----------------RLIFRKFN------------------------------LYSKA
GBJM01015437                 -----------------RLIFRKFN------------------------------LYSQA
GBJM01044964                 ----------------HRLIFRKFNNKKPHLIGKLSASQKDFWDIDRD-------LYDAA
GBJM01082559                 ----------------HRLIFRKFNNKKPHLIGKLSASQKDFWDIDRD-------LYDAA
GBJM01090931                 -----------------RLIFRKFN-----------------------------------
GBJM01032236                 -----------------RLIFRKFN-----YLKSFLSKNITDMIDINSYSPLHYALYKDA
                                              ********                              **::*

Delta-latroinsectotoxin-Lt1a LNINKLKAVDEATTLIEKGADIEAKFDNDRSAMHAVAYRGNNKIALRFLLKNQSIDIELK
GBJM01109678                 ------------------------------------------------------------
GBJM01091322                 ------------------------------------------------------------
GBJM01015437                 ------------------------------------------------------------
GBJM01044964                 DGVHKQLSVAEFEKLFNAKA----------------------------------------
GBJM01082559                 DGVHKQLSVAEFEKLFNAKA----------------------------------------
GBJM01090931                 -------DVEIFEILIEKNS----------------------------------------
GBJM01032236                 DSALELLNVC-----------------------------GD-------------------
                                                                  ANK domain
Delta-latroinsectotoxin-Lt1a DKNGFTPLHIAAEAGQAGFVKLLINHGADVNAKTSKTNLTPLHLATRSGFSKTVRNLLES
GBJM01109678                 -------------------------------AKTSKTFSKTLHLATRS------------
GBJM01091322                 -------------------------------AKTSKTNLTPRNLLTRS------------
GBJM01015437                 -------------------------------AKTSKTFSKTLHLATRS------------
GBJM01044964                 -------------------------------ARNLKTNLTPLHLATRS------------
GBJM01082559                 -------------------------------AKTSKTNLTRNLLATRS------------
GBJM01090931                 -------------------------------AKTSKTNLTPLHLATRS------------
GBJM01032236                 -------------------------------AKTSKTNLTPLHLATRS------------
                                                            *:::**:...::*:***
                                                                       ANK domain
Delta-latroinsectotoxin-Lt1a PNIKVNEKEDDGFTPLHTAVMSTYMVVDALLNHPDIDKNAQSTSGLTPFHLAIINE----
GBJM01109678                 ------------------------------------------------------------
GBJM01091322                 ------------------AVIGTFQVVDAMLKHPDTDKNAQSVSGLTPFHLAIINE----
GBJM01015437                 ------------------------------------DIDAKTADGSTALHLAAAGR----
GBJM01044964                 ------------------------------------NIHAKFELDRTAMHAAAKSG----
GBJM01082559                 ------------------------------------NIHAKFELDRTAMHRNKIISELVE
GBJM01090931                 ------------------------------------DINSKAKDGKTILHLAASRG----
GBJM01032236                 ------------------------------------NKNLKATGKSSPLHFAVTFE----
                                                  ANK domain
Delta-latroinsectotoxin-Lt1a ------SQEVAESLVESNADLNIQDVNHMAPIHFAASMGSIKMLRYLISIKDKVSINSVT
GBJM01109678                 ------------------------------------------------------------
GBJM01091322                 ------NKDAAESLVESNADLNIQDVNHMAPIHFAATMGSIKMLKYLISIKDKVSINSVT
GBJM01015437                 ------KMKTVEALLNKGANLKEFDNNRYLPIHKAIKNEDIDMVR---------------
GBJM01044964                 ------NSRIALRFLLKRVNLDIKDKKGHTPLHEAAEAG---------------------
GBJM01082559                 QKWLIDETTDALRFLL---------KKGHTPPEEAAEAG---------------------
GBJM01090931                 ------AFSIVKRLLEMGATVIDYDKNDYTAAHYAVLNDD--------------------
GBJM01032236                 ------RNKIISELVEQKWLIDETTDDGYTALHLAAMRKD--------------------
```

Figure S7. The sequence alignment of the delta-LIT-Lt1a family belonging to ANK superfamily from *Latrodectus geometricus* venom gland. Alignment was performed with the Multiple alignment program for amino acid or nucleotide sequences (MAFFT) program, using delta-LIT-Lt1a family from spider *Latrodectus tredecimguttatus* (UniProt ID: Q25338.1) as a reference. The green line presents the several ankyrin repeat (ANK) domain in this family.

```
GBJM01057151    FN----------------------LFRILAQA----------------------------
GBJM01005424    MKMAL--------EFS----DYEN------------------------------------
GBJM01136397    MPI---EIKSNTAPFR----RAEQLKRW------EDSETNQVSATIPKDKKN--------
GBJM01115112    MS----------------------------------------------------------
GBJM01070559    MS-------------------LVDLGKRLLEA-ARLGETDEVRLLMTNGA-------PF-
GBJM01094360    ME-------------------------------------DQVPTDLNS------------
GBJM01133263    ME----------------------------------------------------------
GBJM01110061    MPSIAC---------------LQTLQRELADSIIRCAPLDDIRILLACGAKVN---EPVT
GBJM01073508    ------------------------------------------------------------
GBJM01064051    ME----------------------------------------------------------
GBJM01084503    MTGFG-SFHQGAGKFQVCEY----LVKSISPT--PDIAVNQLKIAVSQGQT---------
GBJM01040666    MKLF----------------RFSKYFRFLTPN--CQQKLRNLRCSLYTKSYACKYEEPF-
GBJM01072127    KK-----------------------------------QINEVNRKLDSHSMA--------

GBJM01057151    ------------------------------------------------------------
GBJM01005424    ------------------------------------------------------------
GBJM01136397    ------------------------------------------------------------
GBJM01115112    ------------------------------------------------------------
GBJM01070559    ------------------------------------------------------------
GBJM01094360    ------------------------------------------------------------
GBJM01133263    ------------------------------------------------------------
GBJM01110061    QGLRPIHYAAFQQYFEALNLLLVRGCDPNAMDEIGYTALHLCAERGYIDLVLLLLEHKAR
GBJM01073508    ------------------------------------------------------------
GBJM01064051    ------------------------------------------------------------
GBJM01084503    ---------------SVCRLLLSS------------------------------------
GBJM01040666    --LFPF---LFRKNNFKNSILIGR--------TFGLGVLYVC------------------
GBJM01072127    ------------------------------------------------------------

                                ANK domain
GBJM01057151    ---------------------------------------ILYLDATILDI----------
GBJM01005424    ----------------RDELFDEEILVRAASSGDFEKVSKLLKTGISPDAD---------
GBJM01136397    ----------------KIKFSDGCVFLAACAASDVEEVERLLKLGTDINT----------
GBJM01115112    ---------------------GRIPLHWAASGSQKNVVDYLLQQGSPVDE----------
GBJM01070559    ----------------TTDWLGTSPLHMAAQYGHTSTAEVLLRGGISRDA----------
GBJM01094360    ----------------VLQYYGPYPLHKTAFLGHKMALTFLLESGLSPNQ----------
GBJM01133263    ------------------------------------------------------------
GBJM01110061    VSFTGMDREDNALGNPPRATIADEPLRLAIKNNHFEVADILLRHGADPNARYFLGAEINL
GBJM01073508    ------------------------------------------------------------
GBJM01064051    ------------------VLSRNNLMKQAVKTGKVELVSRLLETGVSANLR---------
GBJM01084503    ------------------SLPDFDSVRLAAFCGHIPVLCALSDKEINWNH----------
GBJM01040666    ----------------CAEKRYDDQLFLAARLGNEKEVRRLVENGIDVNQ----------
GBJM01072127    ----------------------------------LETVSRMVNKTL--------------
                                          ANK domain
GBJM01057151    ---------------------EDKNLDTPLNLASQNSHIDM-VKYFIDLGAKVNTRNKKG
GBJM01005424    -------------EYNKALFYASKNYMTPLQLAAENGYSDI-VDILIAYGADVNAKNRFD
GBJM01136397    ---------------------ANVDGLTALHQACIDNNLEM-VQFLVEHGCDVNRGDNEG
GBJM01115112    ---------------------ADDSNWTPLMIAASVGCADI-VSTLIGRGANVNALNQTG
GBJM01070559    ---------------------RTKVDKTPLHIAAQEGHLPI-VELLISHGAEVDARDMLR
GBJM01094360    ---------------------TNYDCLTPLHEACIQNRADC-AEILINYGAKVNANSVDG
GBJM01133263    ------------------------------------------------------------
GBJM01110061    ISPLNTKFLQLLLKYGADPNSRDRTGLTPLMKACRHPQGLLSAQILIKYGANVNEMTPER
GBJM01073508    ------------------------------------------------------------
GBJM01064051    ---------------------VNGDGGYLLH---HTDNAEI-ASLLIEYQADIEARNGYG
GBJM01084503    ---------------------QDEHGRTALCWACINNQTES-VRFLLERGARVNLTDNQL
GBJM01040666    ---------------------RHKYGWTALHVAAINGKTKV-VEELLKLGANPNLGDNFS
GBJM01072127    ------------------------------------------------------------

                                                  ANK domain
GBJM01057151    Q------------------------------------APLLAFSKKGNLDMVKY------
GBJM01005424    V------------------------------------TALHMAAEKGHFNCLRSLLEANA
GBJM01136397    W------------------------------------TPLHATASCGFLSIAKYLIEQ--
GBJM01115112    Q------------------------------------SALHYAASKNRENIVKILLD---
GBJM01070559    M------------------------------------TPMHWAVERGHYLVVKVLLK---
GBJM01094360    G------------------------------------TPLCDACAAGSVTCVELLMR---
GBJM01133263    -------------------------------------LCLHEACKAGNAQIVNKILN---
GBJM01110061    HD---------------------------------NRTVLHYAVLSGNMDTVKLLLK---
GBJM01073508    ------------------------------------------------------------
GBJM01064051    F------------------------------------TPLFSAVSKNNFDLVEVLLL---
GBJM01084503    F------------------------------------SPIHLAAMYGNPDIVQMLLH---
GBJM01040666    NVYQVAKDKHVHSLEVWSTREEEFNSRLNLRASFRNCTALHYAVLANNIKLVEILLK---
GBJM01072127    -------------------------------------SAVEKSREEMNERFDEVLVS---
```

Figure S8. The sequence alignment of the ANK family belonging to ANK superfamily from *Latrodectus geometricus* venom gland. Alignment was performed with the Multiple alignment program for amino acid or nucleotide sequences (MAFFT) program with their sequences. This family was clustered only the domain prediction. The green line presents the ankyrin repeat (ANK) domain in this family.

```
GBJM01057151    ------------------------------------------------------------
GBJM01005424    ECSIPTKFSKSGSYTAVPHLGGTTPLHLAASNNHIDCVKELIQYGADYNAVDERGRTSLY
GBJM01136397    ----------GGAKVDAVNNDGDLPIDITEST-------EMEEYLSD--VLDEQG-----
GBJM01115112    ------------------------------------------------------------
GBJM01070559    ------------------------------------------------------------
GBJM01094360    ------------------------------------------------------------
GBJM01133263    ------------------------------------------------------------
GBJM01110061    ------------------------------------------------------------
GBJM01073508    ------------------------------------------------------------
GBJM01064051    ------------------------------------------------------------
GBJM01084503    ------------------------------------------------------------
GBJM01040666    ------------------------------------------------------------
GBJM01072127    ------------------------------------------------------------

                                           ANK domain
GBJM01057151    ------------------------------------------------------------
GBJM01005424    IAAKLGYSECILAHLKNA-----VGRDILSLPSFETSDTPLHFAVS-RG-LIDCVSSLL-
GBJM01136397    IDCDLARSVEQRLMLEDAKAILNSGADLNDRIHPRTGATPLHVAAA-KG-YIDVMKLLI-
GBJM01115112    -----------------------NHANINSADH--MGSTPLHRAAS-KG-NIKIMNIFLD
GBJM01070559    -----------------------SNADVNACSK--FDKTPLDIAKD-NM-YKDLVDLLSK
GBJM01094360    -----------------------MGAEVN---PPLALSTPLHEACL-RG-SLDCMTMLI-
GBJM01133263    -----------------------TNKYLLAVEDPFKNWTPIHWAAY-NG-HLECLRLVAS
GBJM01110061    -----------------------HGAVVKFSSD-YLKPSPLDFAIL-RG-NPEMVKLLI-
GBJM01073508    ------------------------------------MPCHQDSAVL-RG-NVKILEFLL-
GBJM01064051    -----------------------KGANVHSIAP--GGNNILHHAVEMKGCNKDIVSLLI-
GBJM01084503    -----------------------HGADPLLKTK--ENKTALDLAVI-VG-NFSTCISLI-
GBJM01040666    -----------------------NAADPFKSND--LGHQPIDYAAD-EE-MKRLINQYME
GBJM01072127    -----------------------------------IESTKIEPVV---------------

                                                       ANK domain
GBJM01057151    ------------------------------------------------------------
GBJM01005424    --------------------------------------------KFGSDVNHMNYAGC--
GBJM01136397    --------------------------------------------QGGADLNAQDNDGW--
GBJM01115112    QY------------------------------------------RNQLDINCKDCVGN--
GBJM01070559    HVVNPDAVKQISPAKVKLVQVTQPNNIPSSTINVPVLIKSPGIIPLSVTTNSKNQTNSVH
GBJM01094360    --------------------------------------------NAGARLNANDCHFG--
GBJM01133263    --------------------------------------------IEPTTINIQSSKTFQ-
GBJM01110061    --------------------------------------------DSGADVNLGSPIIG--
GBJM01073508    --------------------------------------------NSGADINAVSTVVG--
GBJM01064051    --------------------------------------------KHGCSLKFLNGLTR--
GBJM01084503    --------------------------------------------KKGVKTTEEAFTRAR-
GBJM01040666    KY---EEIKRERDAE-------ERRKFPLEQRLKQYIVG-----QEGAITNVAAAIRRKE
GBJM01072127    ------------------------------------------------------------

GBJM01057151    ------------------------------------------------------------
GBJM01005424    -------SPL------------------------------HLAVTPLND--------NEG
GBJM01136397    -------TPL------------------------------HAAAHWGQK-----------
GBJM01115112    -------TPL------------------------------HLACEEERI-----------
GBJM01070559    TA----ASPIS-----VSNVTSIKGMQYTSMNSPSIKKPVYLRTDQLSPEYL----TNLR
GBJM01094360    -------TPL------------------------------HAATTTNHP-----------
GBJM01133263    -------TPL------------------------------HCAAEGRHP-----------
GBJM01110061    -------CPL------------------------------HIALSEKVD-----------
GBJM01073508    -------SAL------------------------------HLSLVEKIP-----------
GBJM01064051    -------SPL------------------------------HVALANDEINW---------
GBJM01084503    -------DHP------------------------------HIYST---------------
GBJM01040666    NGWYDEDHPLVFLFMGSSGIGKTELAKQVAN---------YLHKDKKNA-FIRLDMSEYQ
GBJM01072127    -------SKI-------NNFVR------------------YFETEKERI-----------

GBJM01057151    ------------------------------------------------------------
GBJM01005424    SGLDILKLLILEGYNVDVNQLDGSD-----------LSPLHFVVFNGRNNCSRRRPEMAK
GBJM01136397    ---EACAVLADNLVNMDIQNLAGQT--CFDV-AEPDLVKLLEELKKKQTNMQKNRPDIQA
GBJM01115112    ---EAAKLLMEAGADADTLNKEKKNS----------------------------------
GBJM01070559    KGNSSSNAIPQPTYSSDSEESENNDPSCTNV-----LTTLAALAEATAPNASMSTSEAMK
GBJM01094360    ---DCVKLLLQAGAFVNAAKIHETA--------------LHIAARENYSN-------IAK
GBJM01133263    ---ECVLWLLQAGVNPE-----------------------------------------VK
GBJM01110061    NKEDIIKILLEYGADPNAVSVSSDGP------------LLKPPIGEYFNSCDHPTVDVVR
GBJM01073508    NQIQIVETLLKNGADPNAITTVEGKP------------VLKPPLGEYLQNCEHPRLEIVR
GBJM01064051    ---EAVKVLIENGVDIN-------------------------------------------
GBJM01084503    -------LLCKLLSDGHALYKEGQFP-------E------------------------AS
GBJM01040666    EKHEVAKLI---GSPPGYIGHDEGGQLTKQLKACPNAVVLFDEVDKAHTD-------VLT
GBJM01072127    -----------------------------------------------------------K
```

Figure S8. The sequence alignment of the ANK family belonging to ANK superfamily from *Latrodectus geometricus* venom gland. Alignment was performed with the Multiple alignment program for amino acid or nucleotide sequences (MAFFT) program with their sequences. This family was clustered only the domain prediction. The green line presents the ankyrin repeat (ANK) domain in this family. (Cont.)

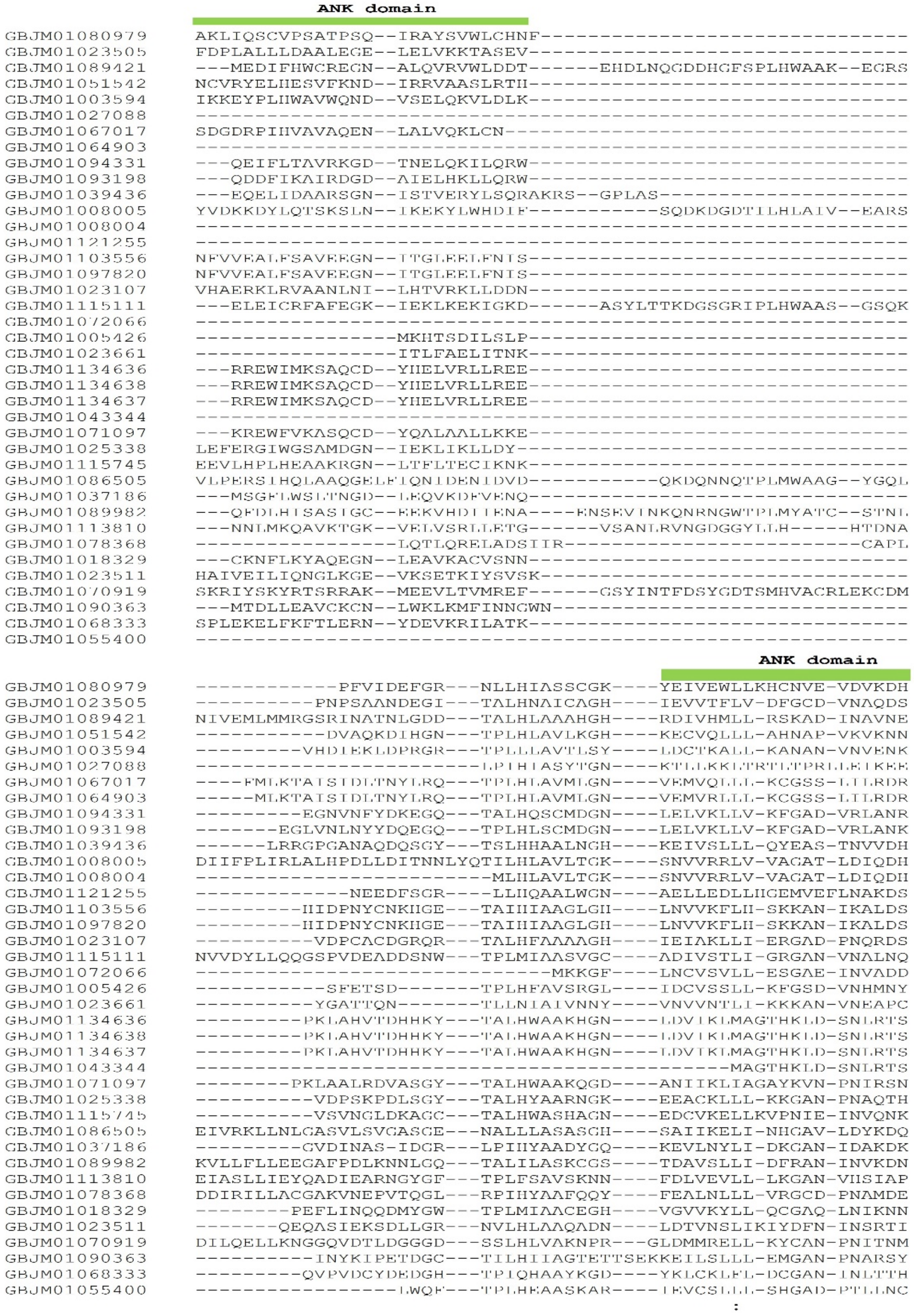

Figure S9. The sequence alignment of the ANK-like family belonging to ANK superfamily from *Latrodectus geometricus* venom gland. Alignment was performed with the Multiple alignment program for amino acid or nucleotide sequences (MAFFT) program with their sequences. This family was clustered only the domain prediction. The green line presents the ankyrin repeat (ANK) domain in this family. (Cont.)

```
Scoloptoxin SSD14    M----------------------------------------------------------G
GBJM01115433         MNREGSVGRISHGSYVQLNTFDEKENNFVQNHTTLPVTWPYQRCICSKRD-TVLIAVVLT
GBJM01137901         M-----VNIVNPFGAQKWNVGDKGESKI-------------------KRSLTIRFKVSLG
                     *

Scoloptoxin SSD14    TSYRKLGYVL-FLMLGMIVEEGIAYVRVKEKQSSPSFMGIYDESAVASDAVPCAEAGQSM
GBJM01115433         AAFILITTSL-VLGLPQIEEKKRLYLDTSFPVSS-SVLQTFDSAAIVTDGIPCAAIGKNT
GBJM01137901         IALVLITLTVAIVVVCFIIPSQESKRRWLPEYNSKSRLGKFENAAISTDAAPCAWIGKDI
                      :   :   : .: :  *  .            .* * :  ::.:*: :*. ***  *:.

Scoloptoxin SSD14    LRQKGSAVDAAIATLLCIGVYNPQSSGIGGGSFMVIYDRSTRTPEVIDAREEAPAAATQK
GBJM01115433         LQNGGSAVDAALSTLFCECVVNPQSMGLGGGFLMTIYIKSSKTSTVIDARETAPSKAHRD
GBJM01137901         LSKNGSAVDAAIAVLLCMGVVEPQSMGLGGGFFMLYYQKELGTTHFIDAREVAPRRASEN
                     * : *******::.*:*  * :*** *:*** :*  * :.  *. .***** **  * ..

Scoloptoxin SSD14    MFKGDKKLSSEGGLSIAVPGELRGMALAHERHGKLDWETLFQPAIRLAKEGFPVGSELAM
GBJM01115433         MFGGNESLALNGGLAIAVPGELRGYKLAHEKYGKLPWKSLIEPSIKLCNDGFKVSKHLAS
GBJM01137901         MFNGNATLAKVGGLAIAVPGELAGYVSAHDNYGVLPWEDLFPPTIKMCEEGIEVNHHLAR
                     ** *: .*:  ***:******* *   **:.:* * *: *: *:*::.::*: *. .**

Scoloptoxin SSD14    ALREQRSHILNSPTLKAVFTDPVSGDILKENDIVIRTKLGETLEKIAKNGADEFY-EGQV
GBJM01115433         KIEKLRQRIIADESMREEFFNNQTNDLYKENDILKRTTFAKTLQKIADQGADVLY-TGEL
GBJM01137901         ALQKYKEDILSREHIRNVFVNNETGDVYKEGDIYKRLDLARTLRIISQEKHSALYGQSNL
                     :.: :. *:    ::  * :  :.*: **.**  *  :..**. *:.:  . :*  .::

Scoloptoxin SSD14    AKDLINDIKSFGGIITAEDLKNYQPVLKNATVAHLSG--CLTLYSVPPPSSGYILSFILR
GBJM01115433         QKEFLKDISNCGGIINETDLANYQPAFKQAVEVPLSGSNNLTFHTVPPPGSGLILSLILN
GBJM01137901         TNEFLEDLQAAGSIINKKDMEYYIPEFRVATQIKIKD--NMTLYSASLPGSGPLLAFMLN
                      :::::*:.  *.**.  *:  * * :: *.   :..   :*:::.. *.** :*:::*.

Scoloptoxin SSD14    VLDKFH-FSKASVSDIENATLTYHRFLETLKYAYAYRTKLGDERIENVSSVIAELTSEDV
GBJM01115433         ILSHYG-MTAEDFKNIDNAALQYHRITEAFKFAFAHRTQLGDEAFVNISQIITNLMSESY
GBJM01137901         VLDGYEDFNPKVTESKESTILTLQRIIETFKFAYASRMDLEDSESKKVLELIDLLANRTY
                     :*. :  :.    .. :.: *  :*: *::*:*:* * .* *.   :: .:*  * ..

Scoloptoxin SSD14    IEKTKQKIEDGKTYEPLHYGAQLA-TNDHGTAHISVVSREGDAVVVTTTINYWFGSGLRS
GBJM01115433         AKTIFEQISDDTTNPPSYYKPDMAPSSDAGTAHVSVLAPSGDAVSVTSTVNTYFGSLCRS
GBJM01137901         ADEIRSKIDDKQTHDPDYYGVNVTIQENHGTAHLSIIAPNGDAVSVTSTINHYFGSQVMS
                      .   .:*.*  *  * :*  :::  .: ****:*::: .**** **:*:* :***   *

Scoloptoxin SSD14    PSTGVILNDEMDDFSAPDIQNIYGVPPSKANFIVPGKRPQSSTCPSIFVNKGGDVVMAIG
GBJM01115433         PSTGIILNNAMDDFSSPNITNYFGIPPSPANYIAPGKRPLSSMCPAIISDNSGDVKMVVG
GBJM01137901         PSTGILLNNEMDDFSSPNITNFFGVPPTGKNHIRPGKRPMSSMTPAIVVDSNKNARLVVG
                     ****::**: *****:*:* * :*:**:  *.* ***** **  *:*. :.. :. :.:*

Scoloptoxin SSD14    ASGGTRITSSVSLTSMRVLWLGRNIKEAIDEPRLHHQLLPDEIEYESKFPNEILEKLKAI
GBJM01115433         GAGGSRIITASAQGVIRTLWLSQDIKQATDAPRIHHQLFPNAIQYESSFPKVYLDKLQTY
GBJM01137901         GNGGTQITTSVAQVIIRNLWLGEDIKQAIDAPRFHHQLLPNYIEHEENYPEDILEELKAK
                     . **::* :: :   :* ***..:**:* * **:****:*: *::*..:*:  *::*::

Scoloptoxin SSD14    GHKTKPAGA-FGSLVVGIKRMKGGTLTANYDYRRGGSVDGSK
GBJM01115433         GHSLLPETQ--ASVFLGIVK-EGGKLKTNVDFRKGGSADGF-
GBJM01137901         GHELKKLGNNMLGIIMGVTRDEDGHLYANSDYRKGGEVDGF-
                     **.         .:.:*: : :.* * :* *:*:**..**
```

Figure S10. The sequence alignment of the centipede family from *Latrodectus geometricus* venom gland. Alignment was performed with the Multiple alignment program for amino acid or nucleotide sequences (MAFFT) program, using scoloptoxin SSD14 (UniProt ID: P0DPU3.1) from spider *Scolopendra dehaani* as a reference. This family presents only the matched sequences.

```
U-scoloptoxin(01)-Cw1a     --------------------LVRTEDEKPPNL--TRVPSGLSFSCDGKK--PGYYADQQM
GBJM01079633               ------------------SYICLIASQKYPTYWEDNLPQ-TSFSCENMVD-GGYYADIET
GBJM01031569               --------------------EVGVSFKKYPTYWEDNLPQ-TSFSCENMVD-GGYYADIET
GBJM01072122               ----------AFNLPDGADLLVGSI-------------R-TSFACPQGAE--GYYADIEN
GBJM01042369               ----------AFFLPDGVELIVGPI-------------N-TNFNCEGLRY--GYYADVDN
GBJM01100968               SEGGARGDEGALGFDGVLARHDGYVKGETP-----TIPQ-TSFSCSAQQYNPGLYADVET
GBJM01051153               DYPTEEIIAEEVEHPGFVGSVRGIPGIDYPAY--TEVPI-TSFSCSDKKYVPGFYADVET
GBJM01010476               ----------------------------------------CNFS-RDKKYVPGFYADVET
GBJM01089616               ----------------VNAKIRGIAGVDYPNY--STIPN-TSFKCSEQQFNPGFYADVET
GBJM01030198               NHDNHENNGGVANHGDLSENFRGTAGVDYPDF--KTIPQ-TSFSCDGQLYEGGMYADVET
GBJM01103095               -----------------------VHSQVLPVY--ETVPE-TSFSCEGRNY--GYYADPEV
GBJM01022671               ----------------------------------------TSFRCSDYDY-AGYFGDVEA
GBJM01136046               ------------------AKLYMEACAKQELF--ASVPP-LNFTCDNKIA-GGYYADIDT
GBJM01106443               ------------------AKLYMEACAKQELF--ASVPP-LNFTCDNKIA-GGYYADIDT
                                                                    .*         * :.* :

U-scoloptoxin(01)-Cw1a     ECQVYHVCTP--------DNEH-----AVLLCGPGTIFNQKHLVC-DFPSN-YACADAAK
GBJM01079633               DCQMYHVCY---------RDRKGIMRSNQFLCGNGTVFDQRHLVCNDYRKV-LKCQES-K
GBJM01031569               DCQMYHVCY---------RDRKGIMRSNQFLCGNGTVFDQRHLVCNDYRKV-LKCQES-Q
GBJM01072122               NCQIFHVCHTTVEEDGSSETTQ-----FSFLCGNQTVFNQLSFTC-SMPEDAVPCSDA-A
GBJM01042369               NCQIFHVCHPITHADGNQETLH-----YSFFCGNQTVFNQLTLTC-AFPEDAVPCQNA-R
GBJM01100968               QCQVYHVCF---------EDRQ-----ESFLCGPGTNFNQRILAC-DFWYN-FDCQNS-P
GBJM01051153               RCQVFHVCY---------EHRR-----ESFLCPVGTTFNQPILAC-DYWYS-SNCSLS-S
GBJM01010476               RCQVFHVCY---------EHRR-----ESFLCPVGTTFNQPILAC-DYWYS-SNCSLS-S
GBJM01089616               GCQVFHYCH---------NHRK-----ESFLCPPGTIYNQPIRSC-DYWYS-SECDLA-P
GBJM01030198               QCQVYHVCH---------DGRM-----DSFICGRGTVFNQEILAC-DYWYS-TDCSKA-P
GBJM01103095               NCQVFHICPGI------YGNRQ-----YSFLCPNQTVFNQALLVC-DYPYN-VDCASA-D
GBJM01022671               GCQAYHVCFP--------DGRN-----ASFLCVNGTVFHQRFFVC-DWWFH-FDCGQA-I
GBJM01136046               GCHIFHLCAP--------SNLQ-TLTDHPFCCHPELVFDQRFLVC-DRPEN-VDCPVS-Y
GBJM01106443               GCHIFHLCAP--------SNLQ-TLTDHPFCCHPELVFDQRFLVC-DRPEN-VDCPVS-Y
                            *: :* *                     : *     :.*    *         *  :
```

Figure S11. The sequence alignment of the centipede family from *Latrodectus geometricus* venom gland. Alignment was performed with the Multiple alignment program for amino acid or nucleotide sequences (MAFFT) program, using U-scoloptoxin(01)-Cw1a (UniProt ID: P0DPW5.1) from spider *Cormocephalus westwoodi* as a reference. This family presents only the matched sequences.

```
U-scoloptoxin(01)-Er1a LTRMPEGVNFACSGKK--PGFYADEGFDCQVYHMCSPEGQLTTYLCGPGTIFNQKKLVCD
GBJM01010476           ---------FS-RDKKYVPGFYADVETRCQVFHVCYE-HRRESFLCPVGTTFNQPILACD
GBJM01124420           YDDFPETS-FVCQNKKHIPGFYADQETKCQVFHVCWP-FRRESFLCPIGTTFNQAVLACD
GBJM01032789           YEEIPETI-FSCEDKKYIPGFYADMDTSCQVFHVCWP-HHREIFLCPTGSTFNQEILTCD
GBJM01055217           YEEIPETI-FSCEDKEYVPGFYADMDTSCQVFHVCWP-HHREIFLCPTGSTFNQEILTCD
GBJM01078726           YIKVPDDIVFDCEDRK--DGYYASVPHKCQLFHYCFA-GARYDFLCANYTLYDQTTFTCR
                                *   .::   *:**.    **::* *        :**   : ::*  :.*

U-scoloptoxin(01)-Er1a LPTNYNCADAAKDAEEANANVFKTQSSTSEP-----------------------------
GBJM01010476           YWYSSNCSLSS-HYYDVNAKKYEPAEV----------LDKTEEDI---------------
GBJM01124420           HWYKSNCSLSQ-AYIDVNLAFGESASTGTVGPMVDSLLTASADDRPIKTEVLSSMKDVSK
GBJM01032789           YWYNSNCDLSP-SFYDSNARYLPTITSESDSIFEES--DKK-------------------
GBJM01055217           YWYNSNCNLSP-SFYDSNERYLPTITSESDSSFEES--DKKSDERYVDTLLLSSSG----
GBJM01078726           FANNVDCESSA-------------------------------------------------
                          . :*  :
```

Figure S12. The sequence alignment of the centipede family from *Latrodectus geometricus* venom gland. Alignment was performed with the Multiple alignment program for amino acid or nucleotide sequences (MAFFT) program, using U-scoloptoxin (01)-Er1a (UniProt ID: P0DPU3.1) from spider *Ethmostigmus rubripes* as a reference. This family presents only the matched sequences.

```
U-scoloptoxin(16)-Er7a MTSTRKLSVSCLIVFMVSSLIAVSSGWLSIGKIAIKDGKCDPKNGNLYAIGEKWYNDEDC
GBJM01022996           MYRYWIL----LLCALLSIMVLNAQGYGVLNDVDTTDGFCDTPGFGRIPVGETRYRDGTC
GBJM01003609           MYRYWIL----LLCALLSIMVLNAQGYGVLNDVDTTDGFCDTPGFGRIPVGETRYRDGTC
                        *     *    *:  ::* ::  :.*:  :..:  .** **. .  .  .:**. *.*  *

U-scoloptoxin(16)-Er7a FEITCVQGDKGSVAQQVASCPVHAVKPGCELVFP-GGTYPKCCPYYECPNS-------
GBJM01022996           EKLTCSQG-----RLILEGCTV-LRNPDCEIVAGLGGQYPSCCPQVICGQNDADDNRI
GBJM01003609           EKLTCSQG-----RLILEGCTV-LRNPDCEIVAGLGGQYPSCCPQVICGQNEADDNRI
                        ::** **         : .*.*   :*.**:*   ** **.***   * :.
```

Figure S13. The sequence alignment of the centipede family from *Latrodectus geometricus* venom gland. Alignment was performed with the Multiple alignment program for amino acid or nucleotide sequences (MAFFT) program, using U-scoloptoxin (16)-Er7a (UniProt ID: P0DQD0.1) from spider *Ethmostigmus rubripes* as a reference. This family presents only the matched sequences.

```
U-scoloptoxin(16)Sm3a MALPSTLSSIVLRWVLFAIFLLLVEAGERQYRTESR-------DGKCVGEDGQVHAVTEI
GBJM01105861          MKV--GMETMLLRYRILYLMIIAFCSLISQISSDLKLQPMKMGFDDCTDEEFKQHPVGTR
                      * :   :.:::**: :: :::: . :   *  :: :        ..*..*: : *.*

U-scoloptoxin(16)Sm3a WYNDNDCSEHTCVNDHTGYYEIIRRCTLIVYPPECRLVNG-TGTRYPKCCCGVTCE----
GBJM01105861          WFSKTKCEEHSCLFIKGILYVMQSGCGKVGYSPECKLVPGKKGANYPECCPQVECQAVEY
                      *:....*.**:*:  :    * :    *  : *.***:** * .*:.**:**  * *:

U-scoloptoxin(16)Sm3a ---LN
GBJM01105861          DYYYD
                          :
```

Figure S14. The sequence alignment of the centipede family from *Latrodectus geometricus* venom gland. Alignment was performed with the Multiple alignment program for amino acid or nucleotide sequences (MAFFT) program, using U-scoloptoxin (16)-sm3a (UniProt ID: P0DQD9.1) from spider *Scolopendra morsitans* as a reference. This family presents only the matched sequences.

```
U-scoloptoxin(16)-Sm4a M-WALTVFVTILAAAIPITGVT--GVCEDMAGNGRAPGEVWTEDSCTLYECGEDDSGELN
GBJM01133952           MNCLLTVFLCIGLAALALSASTEKGFCDTRYGKISVGEETHTEHPCAKHICHSN-----G
                       *   ****: *  **:.::. *  *.*:    *:  .   *. **..*: : * .:     .

U-scoloptoxin(16)-Sm4a TLFVAGCPLSLEIPKGCHYEPRSGNFPYCCPLLVCPDYVDKKNVRRRI
GBJM01133952           TIFSIGCP-EFRAP-GCKIVRNEGKYPECCPKPVCPSTESKA------
                       *:*  *** .:. * **:   ..*::* ***  ***.  .*
```

Figure S15. The sequence alignment of the centipede family from *Latrodectus geometricus* venom gland. Alignment was performed with the Multiple alignment program for amino acid or nucleotide sequences (MAFFT) program, using U-scoloptoxin (16)-sm4a (UniProt ID: P0DQD0.1) from spider *Scolopendra morsitans* as a reference. This family presents only the matched sequences.

```
U19-ctenitoxin-Pn1a QW--------------IPGQSCTNAD-CGEGQCCTGGSYNRHCQSLSDDGKPCQRPNKYD
GBJM01063839        MRGLIALFLLACVVGVALGQSCGELESCGLGECCAGSFYHRTCRRLSEDGEPCERPNNAN
                                     **** : : ** *:**:*. *:* *: **:**:**:***: :

U19-ctenitoxin-Pn1a EYKFGCPCKEGLMCQVINYCQKK-
GBJM01063839        HYTVACPCREGLICSVINRCQTPY
                    .*...***:***:*.*** **.
```

Figure S16. The sequence alignment of the ctenitoxin family from *Latrodectus geometricus* venom gland. Alignment was performed with the Multiple alignment program for amino acid or nucleotide sequences (MAFFT) program, using U19-ctenitoxin-Pn1a (UniProt ID: P83997.1) from the spider *Phoneutria nigriventer* as a reference. The figure presents only the matched sequences.

```
U24-ctenitoxin-Pn1a ----------------------------ARPKSDCEKHRESTEKTG---TIMKLIPKCKE
GBJM01129008        ------MF---------------------------------------------------RE
GBJM01092149        ------MKLVFLFAVACVV----VSVALARPQSECEKHRENAIKHE---SIMKLIPKCKE
GBJM01116416        ------MKVFLIASIVV------CCAVGAFALTHCQEHRERELKND---ISVKLVPKCDA
GBJM01048313        ------------------------------------------------TKIKLIPKCDA
GBJM01076138        MLQLLTMKVFLIASIVV------CCAVGAFALTHCQEHRERELKND---ISVKLVPKCDA
GBJM01071729        MLQLLTMKVFLIASIVV------CCAVGAFALTHCQEHRERELKND---ISVKLVPKCDA
GBJM01101510        ------MK--YIASVLI---LSAVCLAGALAATDCQEHRERELKSN---SKVKLIPKCTE
GBJM01084005        ------MYKVFVFLTIL------VTLTSVKALTDCEEHRKRESNSNAP-LPMRLIPECDN
GBJM01104623        ------MYQFLIILVLT------AFVNGERAKTDCEEHREREKNSRAP-LPMRLIPECDE
GBJM01031395        ------MLKIVGILALCLFATAFAADDDEKVPSACEEDRARRLNATLTESILHLIPECEE
GBJM01041200        -------------------------MNHVSVPSACEEDRARRLNATLTESILHLIPECEE
GBJM01053665        -------------------------MNHVSVPSACEEDRARRLNATLTESILHLIPECEE
GBJM01112330        ------MKILGVTVIFCLTAGALCWEFPGYPGVDCPTARERMLHDP---DVRWMIPKCQE
GBJM01138178        ------MYRSIQLVVLV-AIFSCCLAVDTRRETTCQTHKRNSGGQR---ALMRWNIQCDS

U24-ctenitoxin-Pn1a NSDYEELQCYEDS--KFCVCYDKKGHAASPISTKVK--ECGCYLKQ------------K
GBJM01129008        NGDYEELQCYKDS--KFCVCYDKNGHAASPILSKLK--ECGCFLQR------------K
GBJM01092149        NGDYEELQCYKDS--KFCVCYDKNGHAASPILSKLK--ECGCFLQR------------K
GBJM01116416        NGDYEALQCFTGS--KFCMCWRPDGSHITDPSRKIK--TCKCHVHR------------D
GBJM01048313        NGDYEALQCFTGS--KFCMCWRPDGSHITDPSRKIK--TCKCHVHR------------D
GBJM01076138        NGDYEALQCFTGS--KFCMCWRPDGSHITDPSRKIK--TCKCHVHR------------D
GBJM01071729        NGDYEALQCFTGS--KFCMCWRPDGSHITDPSRKIK--TCKCHVHR------------D
GBJM01101510        EGDYEALQCFEGS--PFCMCWRPDGSHITDPSLKLK--TCSCIAHR------------D
GBJM01084005        NGEYKSLQCFQNS--KFCACWNKNGDPLTQPSGKIK--ACDCLVQK------------H
GBJM01104623        NGDYKPLQCFEGS--TFCACWDKTGQPVTQPSGKIK--ACDCIVQR------------E
GBJM01031395        NGDYAALQCFTAN--DWCVCYRRNGENINTPSKNIK--ACDCVRQK------------D
GBJM01041200        NGDYAALQCFTAN--DWCVCYRRNGENINTPSKNIK--ACDCVRQK------------D
GBJM01053665        NGDYAALQCFTAN--DWCVCYRRNGENINTPSKNIK--ACDCVRQKALWRQHLHSLWNAN
GBJM01112330        DGSFQDLQCYSGAVNDTCMCVAPDGSPLTLPGFGLNVVTCVCFNAQ-----------YK
GBJM01138178        EGNYMPLQCTENTP-KWCTCYNKE-QAITSPSRSTK--TCECHLAR------------D
                    :..:  ***        * *        .      :   * *   :             .

U24-ctenitoxin-Pn1a ERKDSG----RESAIIPQCEEDGKWAKKQLWEFNKSCWCVDEK-GEQVGKIHHDCDSLKC
GBJM01129008        RKIDSN----HDNAYIPQCENDGSWTTRQCWDFNNSCWCVDKN-GNQVGDIKANGEGLDC
GBJM01092149        RKIDSN----HDNAYIPQCENDGSWTTRQCWDFNNSCWCVDKN-GNQVGDIKANGEGLDC
GBJM01116416        TEVTKSNK-GMVGNFIPECNNEGTYAKKQCHASTGLCWCSDED-GNKTTEPARG--EINC
GBJM01048313        TEVTKSNK-GMVGNFIPECNNEGTYAKKQCHASTGLCWCSDED-GNK-------------
GBJM01076138        TEVT----------IIY-------------------------------------------
GBJM01071729        TEVTKSKSIPLIQNIIYS------------------------------------------
GBJM01101510        RVVQTH----LIGSYKPQCDQDGSYSRTQCHGGMGYCWCVDEH-GQKVNKSLND-----C
GBJM01084005        EVEKKG----LVGAFKPQCEEDGKFKKEQCHGSTGMCWCAHPVTGEKTTEPGRG--KPKC
GBJM01104623        TVNQRR----LIGAYSPQCEENGKYKKLQCHGSTGHCWCAEENSGEKTTAPTRG--QLNC
GBJM01031395        DAVTAG------DTYIPKCDKNGYFQAKQCQ--RDECWCVDKN-GKVLTDPQTG--DVNC
GBJM01041200        DAVTAG------DTYIPKCDKNGYFQAKQCQ--RDECWCVDKN-GKVLTDPQTG--DVNC
GBJM01053665        SSIS-----------IHSLEDN----LRYCENIA--------------------------
GBJM01112330        IFEHDH------EAEMPKCEKNGYYAPLQCSKASGKCWCVDPN-GNITVPPSTE--VHSC
GBJM01138178        LAETNS----ITGCDVLKCDRSGAFEKKQCCDTTRKCYCVDPITGQRKTQPSSD-RNIRC
```

Figure S17. The sequence alignment of the ctenitoxin family from *Latrodectus geometricus* venom gland. Alignment was performed with the Multiple alignment program for amino acid or nucleotide sequences (MAFFT) program, using U24-ctenitoxin-Pn1a (UniProt ID: P84032.1) from the spider *Phoneutria nigriventer* as a reference. The figure presents only the matched sequences.

```
U9-ctenitoxin-Pn1a ----------------------------EWCGSNADCGDGQCCTGGSFNRHCQSLADDGT
GBJM01121804       ----------------------------------------------FYHRTCRSLSDNGE
GBJM01100171       RFFFFFSWKMRGLIALFLLACVVGVALGQSCGELESCGMGECCAGSFYHRTCRSLSDNGE
GBJM01036734       ---------MKGFFALLLVVCIVGLSASDYCGSLESCGPNQCCSGGFYHRYCRTYGDKGE
                                                               ::* *:: .*.*

U9-ctenitoxin-Pn1a PCQKPNDYNEYKFGCPCKEGLICSPINYCQKK--
GBJM01121804       PCEKPNDGNYYTVACPCKEGLVCSPIRRCQEPW-
GBJM01100171       PCEKPNDGNYYTVACPCKEGLVCSPIRRCQEHW-
GBJM01036734       PCEQPNPSDSYTVACPCKEGLFCNVIRRCQEEMK
                   **::**  : *...*******.*. *. **:
```

Figure S18. The sequence alignment of the ctenitoxin family from *Latrodectus geometricus* venom gland. Alignment was performed with the Multiple alignment program for amino acid or nucleotide sequences (MAFFT) program, using U9-ctenitoxin-Pn1a (UniProt ID: P83893.1) from the spider *Phoneutria reidyi* as a reference. The figure presents only the matched sequences.

```
                                                        TY domain
GBJM01099190    VEKAKCQGHPVQFKYRGRC-----VDKARCEAQRALALE------QGSKKGIFVPECKED
GBJM01003199    -------------------------AVTKCELEAYEAKKL----FRQGVI-VHWPSCNED
GBJM01095503    -------------------------AETKCQAEVREGMEL----AAESVEDVDWPSCNAD
GBJM01021096    -------------------------EETKCQAAVRKAHEM----IAKGVQDMSIPACNED
GBJM01044701    -------------------------AETKCQKQVRIVQQK----IKLGIADFNLPSCNAD
GBJM01101299    -------------------------AETKCQKQVRIVQQK----IKLGIADFNLPSCNAD
GBJM01062807    -------------------------AETKCQLRAREARQW-----TKVIPGFYVPACKPD
GBJM01128112    ----------------CHCL-VIEPSTSACKVQQALVE-------KYGNLLSFYPRCDGD
GBJM01134247    -----------------------DPSMSACKIQQQIVE-------QQNDLTAFYPRCDGN
GBJM01050951    -------------------------GQSACKIQRRLVQ-------KSKDASAFLPRCTED
GBJM01080217    -------------------------GQSACELQRSMAS-----------GKDFKPTCDPN
GBJM01080218    -------------------------GQSACELQRSMAS-----------GKDFKPTCDPN
GBJM01020865    ---------------------TSLDSLSACNVQRTLVQ-------QFGDPGDFVPICSEN
GBJM01020865    ---------------------TSLDSLSACNVQRTLVQ-------QFGDPGDFVPICSEN
GBJM01036028    ---------------------AGMNDMKPCLLQRTVVS-------QYGSPNDFVPICDDN
GBJM01060745    -------------------------AETACQIRRRNEQDK----SKKGFVGNFIPKCDED
GBJM01121779    -------------------------AETACQIRRKNEQDK----SKKGFVGNFIPKCDED
GBJM01030095    -------------------------QETACQLQRKQEM-------AKNMVGNFVPKCDAD
GBJM01031597    -------------------------QETACQLQRKQEM-------AKNAIGNFIPKCDAD
GBJM01109069    -------------------------QQTDCQQKREEELEK----SRNGLIGNFVPTCDAD
GBJM01028547    VVRDQVLS---QYEW-CSCF---DKAQRPCAAALRR--------VPQGLIGGYKPVCDSE
GBJM01011174    ------------------------VVTSGCMRIRKFNE-------EIDFPGEYTPECKAN
GBJM01106598    -----------------KCSYEKDNSLTKCRMDRDLRRRRQTSEADKYAEDMWIPQCDEE
                                             *                        * *  :

                           TY domain
GBJM01099190    GSYAEVQCRL-STGYCWCVDEAGKPIRGSSIHNSRPNCAPKGGRRTNRRRSSRGKKN---
GBJM01003199    GSYKKVQCA---WTFCTCVDENGVAISYST--DDNEDSS---------------------
GBJM01095503    GSYKSLQCG---FVGCYCVDKDGEMVTDFA--NESEEID---------------------
GBJM01021096    GSYKSKQCGYFGFWGCYCVDENGKTISDVT--RPGKNVV---------------------
GBJM01044701    GSYKSKQCN---LMGCYCVDKEGKRISKIS--KLKPNSD---------------------
GBJM01101299    GSYKSKQCN---LMGCYCVDKEGKRISKIS--KLKPNSD---------------------
GBJM01062807    GSYKSMQCN---WHGCYCVDTDGNANTASV--PRNQ------------------------
GBJM01128112    GNYAAIQCR---QAFCWCANKEGVQLTVSK--PEYPDCSMFRR-----------------
GBJM01134247    GNYAAVQCR---QGFCWCANKQGIQQTKSK--PGYPDCSNVSR-----------------
GBJM01050951    GKYAQIQCR---DKFCWCAKSDGTQLTKSQ--KGKPDCSNQPY-----------------
GBJM01080217    GDYSQVQCR---DGWCWCAKKDGKQLTKSV--QGKPNCAGKPR-----------------
GBJM01080218    GDYSQVQCR---DGWCWCAKKDGKQLTKSV--QGKPDCTGKPR-----------------
GBJM01020865    GDYASIQCR---QGFCWCAAPDGTQLTPSK--AKIPDCSTASS-----------------
GBJM01020865    GDYASIQCR---QGFCWCAAPDGTQLTPSK--AKIPDCSTASS-----------------
GBJM01036028    GDYAYMQCR---EGFCWCAAKDGTQLTKSK--PYIPDCSGLTR-----------------
GBJM01060745    GSYSKMQCNH-STGYCWCADQDGTQISEGV--RGRPNC----------------------
GBJM01121779    GSYSKMQCNH-STGYCWCADQDGTQISEGV--RGRPNC----------------------
GBJM01030095    GSYSQVQCHG-STGFCWCADKDGNQLSKSV--RGQPNC----------------------
GBJM01031597    GSYSQVQCHG-STGFCWCADKDGNQLSESV--RGKPNC----------------------
GBJM01109069    GNYSQVQCYA-STGFCWCSDKNGNKVSESV--RGRPNC----------------------
GBJM01028547    GYYSPTQCHN-GSGTCWCVDRHGVEFTNTR-RRGRPDCEGLLKRGQYHHDDARTKKQ---
GBJM01011174    GYFEPKQCRE-SIHYCWCVDKHGRVTHQPE--PESQDLE---------------------
GBJM01106598    GKYSLKQVK---RKKSVCVDADGTRLFGQEELYASQNVT---------CRCSQYIKNLQI
                * :   *        . *    *
```

Figure S19. The sequence alignment of the ctenitoxin family from *Latrodectus geometricus* venom gland. Alignment was performed with the Multiple alignment program for amino acid or nucleotide sequences (MAFFT) program. These sequences were clustered only the domain prediction. Yellow line marks the TY domain.

```
ω-ctenitoxin-Cs1a M---KVLIISAVLFITIFSNISAEIEDDFLEDESFEAEDIIPFF--ENEQARSCIPKHEE
GBJM01087930      MNFVYIFLAAVVVFFRLISCM--EIGLDSEKDETPNS----PFFLEEARENKQCIKKHYE
                  *    ::: :.*:*: ::* :  **  *  :**: ::    ***  * .: :.** ** *

ω-ctenitoxin-Cs1a CTNDKHNCCRKGLFKLKCQCSTFDDESGQPTERCACGRPMGHQAIETGLNIFRGLFKGKK
GBJM01087930      CTYDKRNCCVGKVFQYSCKCYDFTNSAGFIEPRCKCIKSLLGTITDVGVNLWNRI-TGKS
                  ** **:***   :*: .*:*  * :.:*    ** * :.:     :.*:*::. : .**.

ω-ctenitoxin-Cs1a KNKKTKG
GBJM01087930      KSD----
                  *..
```

Figure S20. The sequence alignment of the lycotoxin family from *Latrodectus geometricus* venom gland. Alignment was performed with the Multiple alignment program for amino acid or nucleotide sequences (MAFFT) program, using ω-ctenitoxin-Cs1a (UniProt ID: P81694.2) from the spider *Cupiennius salei* as a reference. The figure presents only the matched sequences.

```
U12-lycotoxin-Ls1c MKFAVILLFSLVVLAVASESVEEVRREIDIEDLPEQQRG--CADLRQPCTEGDDCSCCGR
GBJM01106920       MKTFITLLLLFAFAYVMIEASPALGAD---SYLSEEARGGACAEIQQDCIAGDDCSCCGK
                   **  : **: :..  *  *:   :  :   . *.*: **  **:::* *  ********:

U12-lycotoxin-Ls1c EGVCNCS-HPHKKGCYCKTAGPLEKLA-----KKFKGCKNK
GBJM01106920       FGKCDCTWNEGIQDCKCK------KLVLVTDWKQNLNCP-E
                   * *:*: :   :.* **      **.     *:  .*  :
```

Figure S21. The sequence alignment of the lycotoxin family from *Latrodectus geometricus* venom gland. Alignment was performed with the Multiple alignment program for amino acid or nucleotide sequences (MAFFT) program, using U12-lycotoxin-Ls1c (UniProt ID: B6DD15.1) from the spider *Lycosa singoriensis* as a reference. The figure presents only the matched sequences.

```
U14-lycotoxin-Ls1b MN----------------SKVFAVLLLLALSTCVLSEKYCPTPRNTSCKKMNIRNNCCRD
GBJM01118330       CDFKHFCVPFKCILEFWKMRVLIIFLVSLGMMTVVNADYCPTRSTLQC--FRAINKCCSD
GBJM01024858       ------------------MRVLIIFLVSLVMMTVVNADYCPTRSTLQC--FRAINKCCSD
GBJM01097875       ------------------MRVLIIFLVSLVMMTVVNADYCPTRSTLQC--FRAINKCCSD
                                   :*: ::*:      *:. .****  . .*  :.  *:** *

U14-lycotoxin-Ls1b SDCTSNAFCCAEPCGNFCHKASD-KPGGRRVDPNASCKTGYVYW
GBJM01118330       ENCGEGQICCSEGCGNTCHEPSSVKTNGVKVKPSKTCRIDTS--
GBJM01024858       ENCGEGQICCSEGCGNTCHVPSLVKTNGVKVKPSRTCRIDTS--
GBJM01097875       ENCGEGQICCSEGCGNTCHVPSSVKTNGVKVKPSKTCRIDTS--
                   .:* .. :**:* *** ** .*  *..* :*.*. :*: .
```

Figure S22. The sequence alignment of the lycotoxin family from *Latrodectus geometricus* venom gland. Alignment was performed with the Multiple alignment program for amino acid or nucleotide sequences (MAFFT) program, using U14-lycotoxin-Ls1b (UniProt ID: B6DD37.1) from the spider *Lycosa singoriensis* as a reference. The figure presents only the matched sequences.

```
U15-lycotoxin-Ls1d MN--SKIFAVLLLLGLLSCVLSDQYCPKSSITACKKMNIRNDCCKDDDCTGGSWCCATPC
GBJM01109572       MERRAKVLFLICMLVIVHVISAQVYCPRRYTRVCFRSITR--CCSDQDCPEGRICCQENC
                   *:  :*:: :: :* ::  :  :: ***:    .* :   *  **.*:**. *  **   *

U15-lycotoxin-Ls1d GNFCKYPTDRP-GGKRAAGGKSCKTGYVY
GBJM01109572       GNRCNNPSSVPTDGSRVRPHDACRID-PF
                   ** *: *:. * .*.*.   .:*: .  :
```

Figure S23. The sequence alignment of the lycotoxin family from *Latrodectus geometricus* venom gland. Alignment was performed with the Multiple alignment program for amino acid or nucleotide sequences (MAFFT) program, using U15-lycotoxin-Ls1d (UniProt ID: B6DD37.1) from the spider *Lycosa singoriensis* as a reference. The figure presents only the matched sequences.

```
                                   Toxin 35 domain
GBJM01020778   MNFVYIFLAAVVVFFRLISAKEIGLDSEKDETPNSPFFLEEARENKQCIKKHYECTHDKR
GBJM01074903   MNFVYIFLAAVVVFFRLISCMEIGLDSEKDETPNSPFFLEEARSD-ECTPLTHECTNDRH
               ******************* . **********************.: :*    :***:*::
                                   Toxin 35 domain
GBJM01020778   NCCVGKVFQYTCKC-YDYTNNAGVVEPR--CKCTKSILGALTDFGVNLWNRVTG----
GBJM01074903   SCCRGPTFKYKCDCLYPFDNSTGTWDKKDLCFCIEPGLHHFLD---EIYDKTVGFFGG
               .** * .*:*.*.* * : *.:*. : :  * * :. *  : *   :::::..*
```

Figure S24. The sequence alignment of the lycotoxin family from *Latrodectus geometricus* venom gland. Alignment was performed with the Multiple alignment program for amino acid or nucleotide sequences (MAFFT) program. These sequences were clustered only the domain prediction. Brown line marks the Toxin 35 domain.

```
La1-like protein 13 MERILKPVFLAILIVLSFSSQCMGFGESCQAGKHIVPVGQQQIDSSTCTLYKCSNYNRKY
GBJM01064203        MARL--PFAFLCVVFVVYIMEIEAKNAFCQHGPHKIPTGEYHYSEKPCMKTKC---NEDG
                    * *:  *. :  ::.: :  :  . .  ** * * :*.*: : ....*   **   *..

La1-like protein 13 ALETTSCATLKMKSGCRMVPGAATAPFPNCCPMMMCKG
GBJM01064203        SMSGLGCATYQLQKGCKVIKG--SGPYPGCCDRPVCPK
                    ::.  .*** :::.**::: *  :.*:*.**   :*
```

Figure S25. The sequence alignment of the scorpion toxin like family from *Latrodectus geometricus* venom gland. Alignment was performed with the Multiple alignment program for amino acid or nucleotide sequences (MAFFT) program, using La1-like protein 13 (UniProt ID: L0GCJ1.1) from the spider *Urodacus yaschenkoi* as a reference. The figure presents only the matched sequences.

```
Toxin-like protein 14     ------MNTY-------------------------------NARLYIFSLALALVIL-KG
GBJM01078041              ------------------------------------------------------------
GBJM01035234              -----------------------------------------MSRYCLIAICFGLMSMIFN
GBJM01018960              -----------------------------------------MSRYCLIALCFGLMSMIFN
GBJM01040129              -----------------------------------------MHLCWSLILCMGITSMFLR
GBJM01042621              ------MRVYEKL---------------------DLSPLIKMHLCWSLILCMGITSMFLG
GBJM01040128              -----------------------------------------MHLCWSLVLCMGITFMFLG
GBJM01032588              M----YMRVYEKL---------------------DLSPLIKMHLCWSLVLCMGITFMFLG
GBJM01034780              -----------------------------------------MKVTFSIIL---LFSFVAA
GBJM01104094              -----------------------------------------MNAMLFASATLLIFSF-SV
GBJM01082588              ------MN---------------------------------RKKLLFVSLVIVMHLI-TQ
GBJM01094424              -------------------IY--------SVIFEIFKQRRKMSSLVTVFLCFGLIVV---
GBJM01020415              -----------------------------------------MSCLVTVFLCFGLIIV---
GBJM01020414              -----------------------------------------MSCLVTVFLCFGLIVV---
GBJM01026824              MLKDYLMDTHSEDSNPGTSLYLGFPYIRVHTLLHNLNKEGNMNRLVMTFICVGLVVL---
GBJM01098304              -----------------------------------------MARLTFALLCVAFAVC---
GBJM01005360              -----------------------------------------MARLTFALLCVAFAVC---
GBJM01089166              -----------------------------------------MARLTFALLCVAFAVC---
GBJM01002509              -----------------------------------------MARLTFALLCVAFAVC---

Toxin-like protein 14     TKCYMYVFLQEPGAAFCVDDSGVRYKPGDVWYDDEKCEKLRCSGAEASLKIIGAGCGII-
GBJM01078041              ---------------------------GDTIYDDENCVKITCRPG----IYYGEGCGSVS
GBJM01035234              TEAFVFRGEMNTTGGFCETTSYGKIPVGDTIYDDENCVKITCRPG----IYYGEGCGSVS
GBJM01018960              TEAFVFRGEMNTTGGFCETTSYGKIPIGDTIYDDEKCVKITCRPG----IYYGEGCGSVS
GBJM01040129              TDGFEFEERLNTSSGFCETEEYGRIPVGNTAYDDEQCVKVICQHY----RLITQGCGVGN
GBJM01042621              TDGFEFEERLNTSSGFCETEEYGRIPVGNTAYDDEQCVKVICQHY----RLITQGCGVGN
GBJM01040128              TEGFEFEERLNTSSGFCETEEYGRIPVGNTAYDDEQCVKVICQHY----RLITQGCGVGN
GBJM01032588              TEGFEFEERLNTSSGFCETEEYGRIPVGNTAYDDEQCVKVICQHY----RLITQGCGVGN
GBJM01034780              LRAYVYIVSMSTEDGYCEYAD-QRVPIGETFYDDVRCEKYVCSAG----KVDAYGCGKEM
GBJM01104094              VHGYRNTVPVDTSDGFCRYKD-QKAALGDVFYDDDACELYTCTEG----EVYVTGCSPFT
GBJM01082588              IYCYTYLEPQFVGEADCFDDDWVEHPLGTVWYDSTKCERLECVFHDGILFVEGFGCSAIG
GBJM01094424              ------GMTATTEEGYCETPE-GRVEVGHNFYSEFPCEKTTCDGHN---RFSSVSCPEF-
GBJM01020415              ------GMTATTEEGYCETPE-GRVEVGHNFYSEFPCEKTTCDGHN---RFSSVGCPEF-
GBJM01020414              ------GMTATTEEGYCETPE-GRVEVGHNFYSEFPCEKTTCDGHN---RFSSVGCPEF-
GBJM01026824              ------VSSESQEQEYCDTPDGGKLKVGEEYYTEFPCARHMCLKGG---GIGGLDCGVM-
GBJM01098304              ------ILGITAEDGFCDHKG-EKIPYGQYQYTENPCMKLRCNDDG---TMSGVGCPTF-
GBJM01005360              ------IMGITAEDGFCDHKG-EKIPYGQYQYTENPCMKLRCNDDG---TMSGVGCPTF-
GBJM01089166              ------IMGITAEDGFCDHKG-EKIPYGQYQYTENPCMKLRCNDDG---TMSGVGCPTF-
GBJM01002509              ------IMGITAEDGFCDHKG-EKIPYGQYQYTEHPCMKLRCNDDG---TMSGVGCPTF-
                                           *   * .  *    *            .*
Toxin-like protein 14     ---HVVGCETVRGSGH-YPNCCPRPKC---------
GBJM01078041              ID-ESTGCRLERGEGR-YPNCCLQIKCD--------
GBJM01035234              ID-ESTGCRLERGEGR-YPNCCLQIKCD--------
GBJM01018960              ID-ESTGCRLERGEGR-YPNCCLQIKCD--------
GBJM01040129              VD-QSSGCRLVRGEGR-HPNCCPRVDCS--------
GBJM01042621              VD-QSSGCRLVRGEGR-HPNCCPRVDCS--------
GBJM01040128              VD-QSSGCRLVRGEGR-HPNCCPRVDCS--------
GBJM01032588              VD-QSSGCRLVRGEGR-HPNCCPRVDCS--------
GBJM01034780              V--SGPGCRLEPGTGR-YPNCCDQPVCD--------
GBJM01104094              FTLDDPKCRLVRGEGS-YPQCCEQPKCD--------
GBJM01082588              ---HPKECRLVSGKGKNYPHCCPQVECPNGVIW---
GBJM01094424              ---HAPECETVQGTGK-WPACCPRPVCPSDES----
GBJM01020415              ---HAPECETVQGTGN-WPACCPRPVCPSDE-----
GBJM01020414              ---HAPECETVQGTGN-WPACCPRPVCPSDES----
GBJM01026824              ---YAPGCKEIQGKGK-YPDCCPRYECPSDNPSASK
GBJM01098304              ---QAPGCKVEKGTGP-YPACCEHPVCPK-------
GBJM01005360              ---QAPGCKVEKGTGP-YPACCEHPVCPK-------
GBJM01089166              ---QAPGCKVERGTGP-YPACCEHPVCPK-------
GBJM01002509              ---QAPGCKVEKGTGP-YPACCEHPVCPK-------
                                 *.   * *   * ** :  *
```

Figure S26. The sequence alignment of the scorpion toxin like family from *Latrodectus geometricus* venom gland. Alignment was performed with the Multiple alignment program for amino acid or nucleotide sequences (MAFFT) program, using Toxin-like protein 14 (UniProt ID: L0GCW8.1) from the spider *Urodacus yaschenkoi* as a reference. The figure presents only the matched sequences.

```
CRISP/Allergen/PR-1 ------------------------------------------MHFQVILMMMWLWL-EAE
GBJM01121080        ---------IGRR------FFFYLFSDIEK-----------MMHIIIAIVALSLPIYTIS
GBJM01137067        -----------------------------------------MMYFIIAIVALALPINIVT
GBJM01058555        --------------------RFCKLELIAN-----------MISVVIFVVALALPFCSNA
GBJM01005256        ------------------------------------------------------------
GBJM01005255        ------------------------------------------MSLFIALLVLTLPIYIIT
GBJM01026995        ------------------------------------------------------------
GBJM01140851        MSAFSHERTILRKPSDSFQTSFLTFHLVVNNKSSSYCKKMTNKFFFGVILFFILAFGEIY

CRISP/Allergen/PR-1 GGTCPTLYKRYSKHHTYCLHPNSTCKILTRGV-SSGDKE-IILREHNKLRSRVATGKET-
GBJM01121080        DKPCPMMYSRLTLEHTFCKAPNASCKIVSTGV-NEQEKA-EILNLHNELRNKVAMGQETR
GBJM01137067        EEPCPEIYRRLSRDHSFCKSPNPTCKFLSKGF-TEKEKA-EIIDLHNMYRNQVAMGQETR
GBJM01058555        EEVCPQKFLRFSKDHSFCKPTNATCKYVAKSFPTKEEQA-EIVKLHNMYRNQVALGQETL
GBJM01005256        ------------------------------------------------------------
GBJM01005255        QETCPKDYLRFTPEHSFCKPPSPTCQFIVKGM-VGKEKAKEIVDIHNRLRNKIAVGQETL
GBJM01026995        ------------------------------------------------------------
GBJM01140851        SDDCPEIYKRYSINHSYCKPKNSSCNILRSYL-TQGEID-QVLSLHNSFRSTIATQQEA-

CRISP/Allergen/PR-1 KYSLPKASDMMQLVWDDELASVAQKHADQCVFEHDCNDCRNVQNFGVGQNLHLRT-----
GBJM01121080        AGGLPVAANMLAFTWDEELATIAQKWAEQCIYNHDCKECRSTKGFSVGQNLGFKWYSCR-
GBJM01137067        AGGLPSASNMLGFTWDDELAAIAQKRVEQCIFEHDCRECRATKSFSVGQNIANNWYSCS-
GBJM01058555        GGGLDSASNMLAISWDDELAEIAQKWAEQCLFEHDCGECRASSSFSVGQNMGLMMYPCTP
GBJM01005256        ------------------------------------------------------------
GBJM01005255        AGGLPTASNMLGFSWDTELAQIAQKWIEQCIYDHDCSECRATRKFAVGQNIGFSWNACS-
GBJM01026995        GNSLP-------------------------------------------------------
GBJM01140851        SNKLPPAANMLQMEYDGELAAVAQNYADQCIYAHDCPSCRRVPNFGVGQNIAIQRLTG--

CRISP/Allergen/PR-1 SSAKFSDITWAGAVNGWYDEVKDFNKRQISGFIDGKGPPQTGHFTQAVWATSWRVGCGRS
GBJM01121080        GNCKIRNAEWTPKITAFYEEVKDYSKELIKK-HPRSYPKTIGHFTQVIWARTYKIGCGFA
GBJM01137067        GKCEAQKGDWAGRIKKFYDEVEVYDNSLIEK-HPQNYPNVIGHFTQVVWATTYKVGCGFA
GBJM01058555        GNCKPKQANWTGTVKMFYDEVGVFDKKLVKE-VDFSGP-MIGHFTQVIWAETYKVGCGYA
GBJM01005256        ------------------------------------------------------------
GBJM01005255        GSCDTVEPNWEDQITRFYDEVKDFRKEFVNS-VAYPGPGVTTHFTQIAWAKTYRVGCGFV
GBJM01026995        ------SPDWESTIQNLYDEVKDFYPKWISP-FRAPKYPITGHYSQALWATTWKVGCGYT
GBJM01140851        NNLSPPQPDWSFAVGDWFEEIVYFSPNLICDFQPPNGGGEYRHFTQLIWARTFRVGCGYT

CRISP/Allergen/PR-1 MFKD----GNTFKDLYTCNYGPGGNMKNAIIYTKGKPCSGCPLNSCCGKAC-G--GISYD
GBJM01121080        SFEGTDDGVPGIFQHYVCNYGPSGNLRNFPVYNEGSPCSRCPENSCCGTSC----NEHFP
GBJM01137067        SYESLYKGMQGVKQHYVCNYGPAANLPGASLYKKGAPCSGCPENSCCGSSC----NAKFP
GBJM01058555        SYEEPFFGTEGAKQLTVCNYGPAGNLGR--IYMEGPPCSKCPENSCCGESC----NKEFP
GBJM01005256        ----MEFGYDGIKQLYVCNYGPKGNMQKEPLYIEGPPCTKCPTNA---------------
GBJM01005255        AFKHMEFGYDGIKQLYVCNYGPKGNMQKEPLYIEGPPCTKCPTNACCGSSCKGKRQQKYP
GBJM01026995        VHSYSNDPRFKYVELYTCNYGPTGNLYMQDMYRIGPACSQCPENTCCGRQC----E----
GBJM01140851        MFQH----GNTFTRLYICNYGPAGNTYGSCVYDQGPACSACPENTCCGEYCPG--GPSSP
                                     ***** .*     :*  * .*: ** *:
```

Figure S27. The sequence alignment of the SCP family from *Latrodectus geometricus* venom gland. Alignment was performed with the Multiple alignment program for amino acid or nucleotide sequences (MAFFT) program, using CRISP/Allergen/PR-1 (UniProt ID: W4VS53.1) from the spider *Trittame loki* as a reference. The figure presents only the matched sequences.

```
GBJM01055086    ME-EINLLTPWFALWVTFVCCSLTFDIRVQGKDVPYDLEQDEMVSLLDNLEIIDSGPPVV
GBJM01057984    MKTKLHILNLFFIILGLCAGYPRLSRPKLHGTAIP-------------------------
GBJM01056476    MN---HVILLVLLVVTSCHAVPRLQRPRIYGNAIP-------------------------
GBJM01104565    MEGRIQAL-LTVVLLLTPLSTTKLLRPKLRGRAIP-------------------------
GBJM01104566    ------------------------------------------------------------
GBJM01057682    MDCLLQKL--------KGLRSSKQDSPKYRRQRTTTRSERTSHASTSDTSSYYSASSNFI
GBJM01038322    MQ-------------------------RIRRKYSNY---------LTTGEDNYLFNNSFL
GBJM01052569    MD----------------------------------------------NNDDWDNDSCFL
GBJM01002830    ME----------------------------------------------------------

GBJM01055086    DI-DEGMQDSIQKNRRRDVNGRGMDQEMKLLMVELHNLYRGNVTPPAADMSFLEWDQELG
GBJM01057984    ---------------PRDLDPT--NNATKRKILLAHNFFRSRVDPPASDMLEMTWHDEAA
GBJM01056476    ---------------QRDLDPS--NDATKKKIVLIHNFFRSRVQPPASDMLAMSWHDGAA
GBJM01104565    ---------------MRDLDTR--HVNTKKKIVLMHNFYRAKVEPPAGDMLQMTWHKGAE
GBJM01104566    ------------------------------------------------------------
GBJM01057682    DKVILGRHIYIRGKPEDPPDYG--ESEFVIDCIHWHNVFREKHDVPP-----LKLNNQLC
GBJM01038322    ---------------KQNLENE--EDDFAQECLRCHNHYRAQHGCPP-----LHLSLQIS
GBJM01052569    P-------------PSKSLSFD--VEDFRQRMIAAHNNYRRLHDSPD-----LTHLQDLE
GBJM01002830    ----------------NTGSSE--LWDFKRETFNAHNNYRTMHGCPP-----LVMCEELS

GBJM01055086    DLAQMWADGCK-FDHGFPEIRYSGKGGKYGQNLYMGT--------DPSGKHAIWMWYEEY
GBJM01057984    EDAQRWAEACQLLVHDNITGRWTEDFGSCGQNIFVANV-------QVPWFFATKVWFLEK
GBJM01056476    EDAQRWAESCMLLRHDNTTGRWTEDFGTCGQNIFVASV-------QVPWFFAAKVWFLER
GBJM01104565    EAAQMWAESCQFLIHDKPLSRWVEDFGSCGQNIFVSST-------QVDWMFVIKAWYMEH
GBJM01104566    -------------------------------------------------MFVIKAWYMEH
GBJM01057682    SMAQFWANHLA----HTNTFSHRNI-RDIGENLFSKWSYIPDF--DITAKQVTRYWYEEI
GBJM01038322    SVSQQWANELS----REDHMHHSHR-SQYGENLFVAYLSNNSTVPNIRGNQVVESWYSEH
GBJM01052569    DGAQIWAETIA----AKGYLQYCEHLQNIGESLYCINLIGS----PPSAEDIVRNWYREI
GBJM01002830    AIAQSWAEKLA----EKGFLQYSEN-PGLGENISLVDLEQP----TRKGEQIVKEWYKEI
                                       SCP domain                      *: *
GBJM01055086    LDYHF-HNMTCP--VGKKCGHYQTMAASKSQKIGCGWKKC-GF------RYYIVCHYFPP
GBJM01057984    FNFTYGEDLNDP----NIVGHYTQMVWYSTHRMGCGFNYCEANNVTRHPYFSYVCNYCPI
GBJM01056476    DNFTYGKNLNNP----DVVGHYTQMVWYSTHRVGCGFHYC-GPEVTKVPYYSYVCNYCPI
GBJM01104565    RNFTYGSRKNDL----KSVGHYTQMVWYNSHRIGCGFNFC-GKDVARRPFYNYVCNYCPI
GBJM01104566    RNFTYGSRKNDL----KSVGHYTQMVWYNSHRIGCGFNFC-GKDVARRPFYNYVCNYCPI
GBJM01057682    KSYNF---FQDPNLLHVKAGHFTQMVWRSSTDFGVGKAR------SRCGKVIVVANYKPA
GBJM01038322    IFYPFDGHITKE--IISKSGHFTQVIWSNSRELGVGMAT------SKTNRFYVVANYYPA
GBJM01052569    KNYSF---AEPK--WRKGSFHFSQMLWRSTYHIGVGVAPV-----PGEPKLFVVVRYYPG
GBJM01002830    NNYNY---SKPG--WKRGAYRFSQLLWKSTTEIGVGVAKI-----PGQKKAYVVVNYRPA
                  : :               ::  :   .:  .* *                 * .* *

GBJM01055086    -----------LYRKERLYIVGRPCSKCAESGSLCVQNLCITRDQCERNPQICSKAKCNL
GBJM01057984    GNH--------PERFDRPYRNGTACSSC--------------EDNCK--HKLCTN-----
GBJM01056476    GNH--------PDRFDFPYAKGEPCSAC--------------PGQCKF-KKLCTN-----
GBJM01104565    GND--------PRRFSRPYSVGKPCHKC--------------REHCKF-KKLCTN-----
GBJM01104566    GND--------PRRFSRPYSVGKPCHKC--------------REHCKF-KKLCTN-----
GBJM01057682    GNV--------IGEFQDNVFPPI--------------------ND-----MDILNE-----
GBJM01038322    GNILSKFANNVPNKLHSNYMYRQYTK------------------------RISSR-----
GBJM01052569    GNS------NFPGEFQKNVKPRVSTPSTGSITGL--------DETSFV-RTYSTR-----
GBJM01002830    GNN------NMPGEFERNVLPPQKKKIA--------------DDNANI-RKNMNR-----
                             .                                       ..

GBJM01055086    KCQNCGRLNRDECSCECADGWDLADCSSTCVDTNIHCGTKLNKYHCLQSESIKKGQCRKM
GBJM01057984    -------------GCNYADFW------TNCQELN----TTWHHWLCDDETTDRYQACRAT
GBJM01056476    -------------TCPHADSW------INCRELN----ETWHDWLCGNDQNEGHQACRAT
GBJM01104565    -------------SCPYADMW------VNCVDLN----ATFHSWLCGDGAA-GRRACAAT
GBJM01104566    -------------SCPYADMW------VNCVDLN----ATFHSWLCGDGAA-GRRACAAT
GBJM01057682    ------------------------------------------------------------
GBJM01038322    -------------TLHY-------------------------------------------
GBJM01052569    -------------LRSYADGY---------------------------------------
GBJM01002830    ------------------------------------------------------------
```

Figure S28. The sequence alignment of the lycotoxin family from *Latrodectus geometricus* venom gland. Alignment was performed with the Multiple alignment program for amino acid or nucleotide sequences (MAFFT) program. These sequences were clustered only the domain prediction. Orange line marks the SCP domain.

```
α-latrotoxin associated LMWP-2  MLKLICIVFLVTVLTFVVGEDTLDPAEYGCPSDVDMAELTEKNEVCLRC---EDFHKEGV
GBJM01047487                    MLKLICIVFLVTVLTFVVGEDTLDPAEYGCPSDVDMAELTEKNEVCLRC---EDFHKEGV
GBJM01047488                    MLKLICIVFLVTVLTFVVGEDTLDPAEYGCPSDVDMAELTEKNEVCLRC---EDFHKEGV
GBJM01056830                    MLKLICIAFLVTVLTLVAGQDSLDPAEYGCADDINQEDLLKKNDVCLQC---EDLHKEGV
GBJM01000181                    MNKLFFVVFLCLIISVFA----IGPADLGC-TDMPQAEFDEKNANCEKCGNEEGFGEEMV
GBJM01099609                    MSKLFFVAFLCLIISVFA----TTPDEIGC-TDISQAEFDEKNANC--------------
                                * **: :.**  :::...      * : **  *:   :: :**  *

α-latrotoxin associated LMWP-2  AFTLCKTNCFTTEYYKNCVKDLEEAGKE------TEE
GBJM01047487                    A------------------------------------
GBJM01047488                    AFTLCKTNCFTTEYYKNCVKDLEEAGKE------TEE
GBJM01056830                    VFSLCKTNCFTTQYFTNCVKDLEEAEKE------PPE
GBJM01000181                    --SRCRDKCFTDNFYQSCVDLLNKVYEEKDVPVPPEE
GBJM01099609                    -------------------------------------
```

Figure S29. The sequence alignment of the Theriditoxin family from *Latrodectus geometricus* venom gland. Alignment was performed with the Multiple alignment program for amino acid or nucleotide sequences (MAFFT) program, using Alpha-latrotoxin associated low molecular weight protein 2 (UniProt ID: V9QFG7.1) from the spider *Latrodectus geometricus* as a reference. The figure presents only the matched sequences.

Supplementary materials File S2 Shotgun proteomics of *Latrodectus geometricus*

Figure 1. False discovery rate (FDR) curve. X axis is the number of peptides being kept. Y axis is the corresponding FDR.

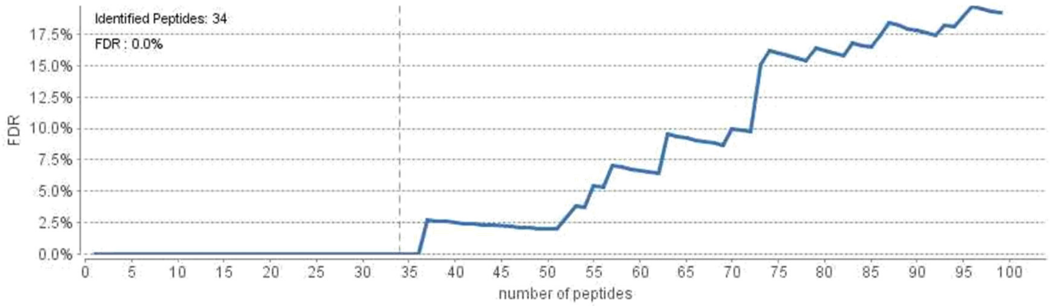

Figure 2. PSM score distribution. **(a)** Distribution of PEAKS peptide score; **(b)** Scatterplot of PEAKS peptide score versus precursor mass error.

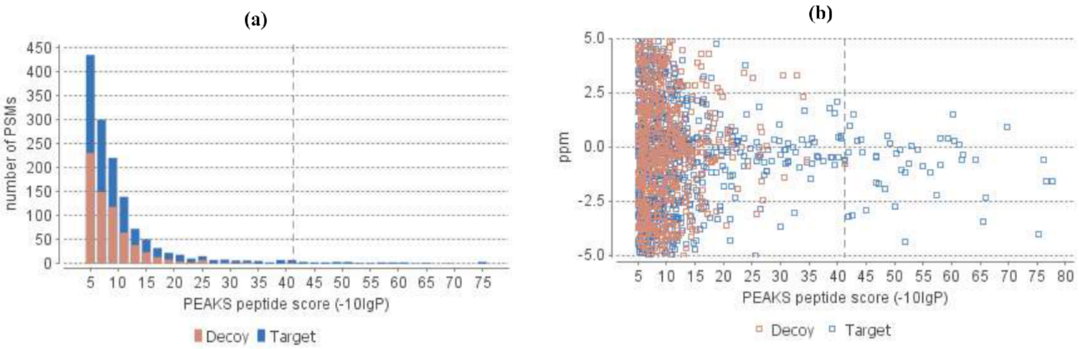

Table 1. Statistics of data.

	#Scans		#Features	Identified			#Peptides	#Sequences	#Proteins*		
	MS1	MS/MS		#PSMs	#Scans	#Features**			Groups	All	Top
Total	7240	4510	34757	44	49	41	34	33	14	22	17
FE2 3uL	7240	4510	34757	44	49	41	34	33	14	22	17

* proteins with significant peptides are used in counts.

** features are identified by DB search only.

Figure 3. Sample overlap for Proteins and Peptides (up to 8 samples). **(a)** All Proteins; **(b)** Top Proteins; **(c)** Peptides;

(a)
Not applicable to only one sample

(b)
Not applicable to only one sample

(c)
Not applicable to only one sample

Figure 4. Distribution of peptide feature detection. **(a)** Feature m/z distribution; **(b)** Feature RT distribution.

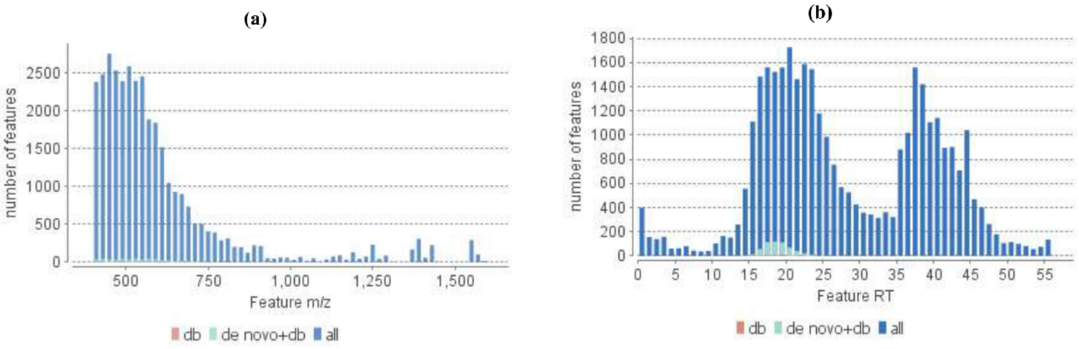

Figure 5. Distribution of identified peptide features. **(a)** Feature abundance distribution; **(b)** *De novo* sequencing validation.

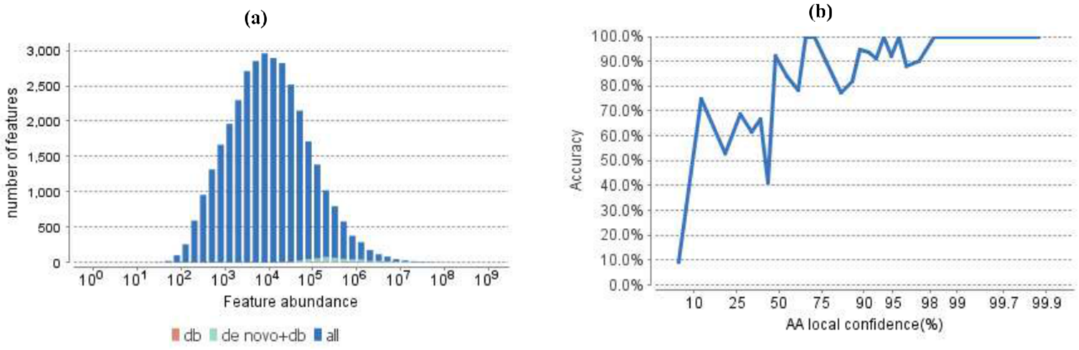

Table 2. Result filtration parameters.

Peptide -10lgP	≥41.3
PTM Ascore	≥0
Protein -10lgP	≥20
Proteins unique peptides	≥1
De novo score(%)	≥50%

Table 3. Statistics of filtered result.

FDR (Peptide-Spectrum Matches)	0.0%
FDR (Peptide Sequences)	0.0%
FDR (Protein Group)	0.0%
De Novo Only Spectra	575

Table 4. PTM profile.

Name	ΔMass	Position	#PSM	-10lgP	Abundance	AScore
Oxidation	15.99	M	7	64.26	1.11E6	1000.00
Carbamidomethyl	57.02	C	6	76.08	1.58E5	1000.00

3. Experiment Control

Figure 6. Precursor mass error of peptide-spectrum matches (PSM) in filtered result. **(a)** Distribution of precursor mass error in ppm; **(b)** Scatterplot of precursor m/z versus precursor mass error in ppm.

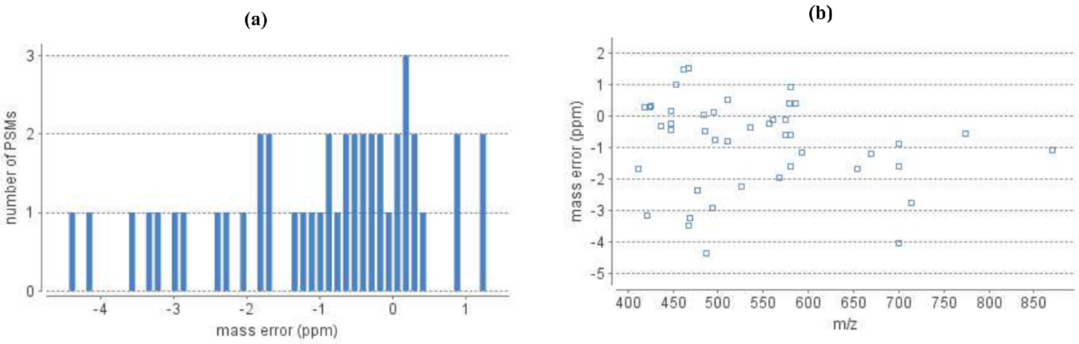

Table 5. Number of identified peptides in each sample by the number of missed cleavages.

Missed Cleavages	0	1	2	3	4+
FE2 3uL	25	6	3	0	0

4. Other Information

Table 6. Search parameters.

Search Engine Name: PEAKS
Parent Mass Error Tolerance: 5.0 ppm
Fragment Mass Error Tolerance: 0.015 Da
Precursor Mass Search Type: monoisotopic
Enzyme: Trypsin
Max Missed Cleavages: 3
Digest Mode: Semispecific
Fixed Modifications:
  Carbamidomethylation: 57.02
Variable Modifications:
  Oxidation (M): 15.99
Max Variable PTM Per Peptide: 3
Database: Uniprot_Theridiidae_Steve_oct21
Taxon: All
Searched Entry: 18128
FDR Estimation: Enabled
Merge Options: merged
Precursor Options: no correction

Table 7. Instrument parameters.

Fractions: 211015-MClass-FE2-3uL-LSM.raw
Ion Source: ESI(nano-spray)
Fragmentation Mode: high energy CID (y and b ions)
MS Scan Mode: FT-ICR/Orbitrap
MS/MS Scan Mode: Linear Ion Trap

Charge Options: no correction
Filter Options: no filter
Process: true
Associate chimera: no

Protein List

Protein Accession Contains:
Protein Description Contains:
Protein Sample Area >=
Protein PTM Contains:

Protein Group	Protein ID	Accession	-10lgP	Coverage (%)	Coverage (%) FE2 3uL	Area FE2 3uL	#Peptides	#Unique	#Spec FE2 3uL	PTM	Avg. Mass	Description
**1**	19930	tr|A0A2L2XZE1|A0A2L2XZE1_PARTP	159.93	9	9	1.5652E7	5	2	12	N	30585	Laminin subunit alpha-2 (Fragment) OS=Parasteatoda tepidariorum OX=114398 PE=2 SV=1
**2**	19424	tr|E7D1U1|E7D1U1_LATHE	149.40	11	11	3.2714E5	5	2	9	N	34753	Uncharacterized protein (Fragment) OS=Latrodectus hesperus OX=256737 PE=2 SV=1
**3**	19944	tr|A0A2L2YMR4|A0A2L2YMR4_PARTP	136.74	15	15	2.7715E5	4	1	8	N	17115	Laminin subunit alpha-2 (Fragment) OS=Parasteatoda tepidariorum OX=114398 PE=2 SV=1
**4**	19929	tr|E7D195|E7D195_LATHE	135.02	11	11	4.6689E6	6	5	7	Y	38031	Hemocyanin subunit G (Fragment) OS=Latrodectus hesperus OX=256737 PE=2 SV=1
**6**	19928	tr|A0A2L2Y2Y0|A0A2L2Y2Y0_PARTP	130.61	13	13	1.7424E6	5	5	5	Y	40057	Arginine kinase OS=Parasteatoda tepidariorum OX=114398 PE=2 SV=1
**5**	19931	tr|E7D1R3|E7D1R3_LATHE	119.54	6	6	6.729E7	4	4	6	Y	37977	Uncharacterized protein (Fragment) OS=Latrodectus hesperus OX=256737 PE=2 SV=1
**8**	19934	tr|A0A2L2YG81|A0A2L2YG81_PARTP	88.99	9	9	1.8532E5	2	1	2	N	24067	Hemocyanin subunit E OS=Parasteatoda tepidariorum OX=114398 PE=2 SV=1
**13**	19950	tr|A0A2L2XYG9|A0A2L2XYG9_PARTP	76.08	10	10	1.5774E5	1	1	1	Y	14239	U24-ctenitoxin-Pn1a (Fragment) OS=Parasteatoda tepidariorum OX=114398 PE=2 SV=1
**9**	19937	tr|E7D194|E7D194_LATHE	74.28	5	5	1.7663E6	2	2	2	N	38030	Hemocyanin subunit D (Fragment) OS=Latrodectus hesperus OX=256737 PE=2 SV=1
**10**	19954	tr|A0A2L2YAY8|A0A2L2YAY8_PARTP	61.26	12	12	3.7555E6	1	1	2	Y	7263	Hemocyanin subunit F (Fragment) OS=Parasteatoda tepidariorum OX=114398 PE=2 SV=1
**14**	19960	tr|A0A2L2Y372|A0A2L2Y372_PARTP	59.35	9	9	3.8482E5	1	1	1	Y	11764	Uncharacterized protein OS=Parasteatoda tepidariorum OX=114398 PE=2 SV=1
**14**	19961	tr|A0A2L2Y2N9|A0A2L2Y2N9_PARTP	59.35	9	9	3.8482E5	1	1	1	Y	11805	Uncharacterized protein OS=Parasteatoda tepidariorum OX=114398 PE=2 SV=1
**14**	19962	tr|A0A2L2Y2N1|A0A2L2Y2N1_PARTP	59.35	9	9	3.8482E5	1	1	1	Y	11763	Uncharacterized protein OS=Parasteatoda tepidariorum OX=114398 PE=2 SV=1
**15**	19955	tr|A0A2L2YL93|A0A2L2YL93_PARTP	52.42	7	7	4.5462E5	1	1	1	Y	10706	U24-ctenitoxin-Pn1a OS=Parasteatoda tepidariorum OX=114398 PE=2 SV=1
**11**	19953	tr|A0A2L2Z3D2|A0A2L2Z3D2_PARTP	44.99	15	15	7.4344E5	1	1	1	N	6421	Laminin subunit alpha-2 (Fragment) OS=Parasteatoda tepidariorum OX=114398 PE=2 SV=1
**16**	20028	tr|A0A2L2ZCD3|A0A2L2ZCD3_PARTP	41.99	11	11	3.9213E5	1	1	1	Y	8297	U24-ctenitoxin-Pn1a (Fragment) OS=Parasteatoda tepidariorum OX=114398 PE=2 SV=1
**16**	20029	tr|A0A2L2XZN5|A0A2L2XZN5_PARTP	41.99	6	6	3.9213E5	1	1	1	Y	16028	U24-ctenitoxin-Pn1a OS=Parasteatoda tepidariorum OX=114398 PE=2 SV=1
**total 17 proteins**												

tr|A0A2L2XZE1|A0A2L2XZE1_PARTP

back to list

| Protein Coverage | Supporting Peptides | Best Unique PSM |

Protein Coverage:

```
  1 YEADEDPKTELGKKLREHFDEILEKLKEALENGKGVKEDTIEKLKEIREQLRDLKVDIGKHAKELLEKLKDKVKEYWKKI
 81 LEKIKPDEASFADEDPKTELGKKLKEHFDEILEKLKEALENGKGVKEDTILKLKEIREKLKDLKIDLSKHAKELLEKLKD
161 KIKDYWQKILEKLKDKASYADDEGSDLQKRFQDLLEQIKDAWENRKTIATNVMTKLKDLYQEMKEKSIPLGDKDKELLEQ
241 LKEIAGGFWKRILVKFGGSN
```

Supporting Peptides:

Peptide	Uniq	-10lgP	Mass	Length	ppm	m/z	z	RT	Fraction	Scan	Source File	Area FE2 3uL	#Feature	#Feature FE2 3uL	Start	End	PTM	AScore	Found By
K.LKEHFDEILEK.L	Y	77.70	1399.7346	11	-1.6	700.8735	2	19.30	3	4454	211015-MClass-FE2-3uL-LSM.raw	1.5181E7	4	4	104	114			PEAKS DB
R.EHFDEILEK.L	N	76.48	1158.5557	9	-1.6	580.2842	2	19.50	3	4605	211015-MClass-FE2-3uL-LSM.raw	4.0773E7	2	2	17	25			PEAKS DB
K.LREHFDEILEK.L	N	66.00	1427.7408	11	-2.4	476.9198	3	18.95	3	4273	211015-MClass-FE2-3uL-LSM.raw	6.9047E6	2	2	15	25			PEAKS DB
H.FDEILEK.L	N	49.23	892.4542	7	0.2	447.2344	2	18.91	3	4244	211015-MClass-FE2-3uL-LSM.raw	9.1588E7	1	1	19	25			PEAKS DB
H.FDEILEKLK.E	Y	48.40	1133.6332	9	-2.0	567.8228	2	21.45	3	5393	211015-MClass-FE2-3uL-LSM.raw	4.703E5	1	1	19	27			PEAKS DB
**total 5 peptides**																			

Best Unique PSM (Scan 4454, m/z=700.8735, z=2, RT=19.30, ppm=-1.6):

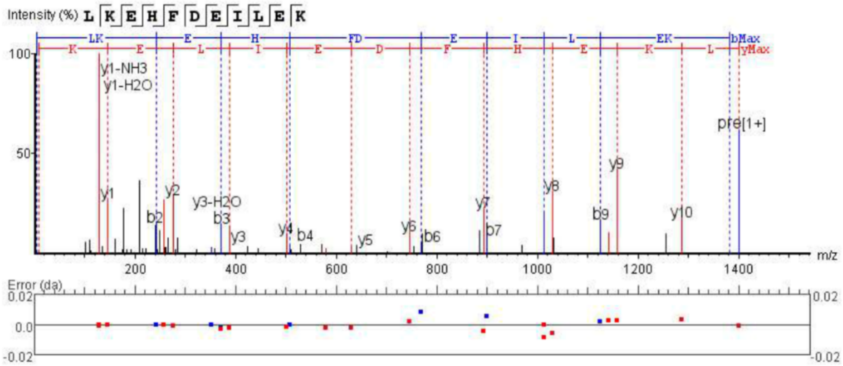

tr|E7D1U1|E7D1U1_LATHE

[back to list]

| Protein Coverage | Supporting Peptides | Best Unique PSM |

Protein Coverage:

1 HEAIHEKNLEKLKEIREQ**LKDLKVDLSNK**AKDLLNKLKEKAKDYWKNLLDKLKPEKRSVDYYFSDDEDPKTELGKK**LREH**

81 **FDEILEK**VKEAIENGKVVKEDYLQKLKEIREKLKDLKVDLSNKAKEALKKLKEKAKEYWQKILDRLQLKEKRSASHVDVM

161 DVLNILKKLKKIIQDKFDAEDLKEKVEKLFGK**GSEFTEQLFK**MLKEKGAQGKQKILDWIDRILDDKERRSISDIYEKVKN

241 FFKDLNIELKEKFTKFGQWVKTQYEKDCKGKGQARECQENCQEFLDDTRR

Supporting Peptides:

Peptide	Uniq	-10lgP	Mass	Length	ppm	m/z	z	RT	Fraction	Scan	Source File	Area FE2 3uL	#Feature	#Feature FE2 3uL	Start	End	PTM	AScore	Found By
R.EHFDEILEK.V	N	76.48	1158.5557	9	-1.6	580.2842	2	19.50	3	4605	211015-MClass-FE2-3uL-LSM.raw	4.0773E7	2	2	79	87			PEAKS DB
K.LREHFDEILEK.V	N	66.00	1427.7408	11	-2.4	476.9198	3	18.95	3	4273	211015-MClass-FE2-3uL-LSM.raw	6.9047E6	2	2	77	87			PEAKS DB
K.GSEFTEQLFK.M	Y	56.29	1184.5713	10	-1.2	593.2922	2	22.79	3	5838	211015-MClass-FE2-3uL-LSM.raw	1.3857E5	1	1	193	202			PEAKS DB
H.FDEILEK.V	N	49.23	892.4542	7	0.2	447.2344	2	18.91	3	4244	211015-MClass-FE2-3uL-LSM.raw	9.1588E7	1	1	81	87			PEAKS DB
Q.LKDLKVDLSNK.A	Y	44.21	1271.7449	11	0.3	424.9224	3	16.59	3	3038	211015-MClass-FE2-3uL-LSM.raw	1.8857E5	1	1	19	29			PEAKS DB
**total 5 peptides**																			

Best Unique PSM (Scan 5838, m/z=593.2922, z=2, RT=22.79, ppm=-1.2):

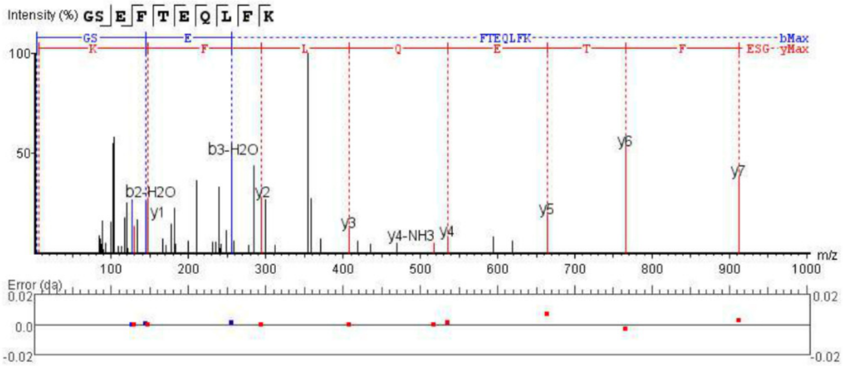

tr|A0A2L2YMR4|A0A2L2YMR4_PARTP

[back to list]

| Protein Coverage | Supporting Peptides | Best Unique PSM |

Protein Coverage:

1 DFLVDLTFEASDDTLEDINKVLKERYNEILEKIKEAIENGKGLREDYIDKAKDLAKKLNGLKVEVGERARDLFEQLKARA

81 KDYWKKLIDKLRDVKESADEYASYADEDPKSVLGKK**LREHFDEILEK**VKEALENGR**IGKDDLIEKLK**

Supporting Peptides:

Peptide	Uniq	-10lgP	Mass	Length	ppm	m/z	z	RT	Fraction	Scan	Source File	Area FE2 3uL	#Feature	#Feature FE2 3uL	Start	End	PTM	AScore	Found By
R.EHFDEILEK.V	N	76.48	1158.5557	9	-1.6	580.2842	2	19.50	3	4605	211015-MClass-FE2-3uL-LSM.raw	4.0773E7	2	2	119	127			PEAKS DB
K.LREHFDEILEK.V	N	66.00	1427.7408	11	-2.4	476.9198	3	18.95	3	4273	211015-MClass-FE2-3uL-LSM.raw	6.9047E6	2	2	117	127			PEAKS DB
H.FDEILEK.V	N	49.23	892.4542	7	0.2	447.2344	2	18.91	3	4244	211015-MClass-FE2-3uL-LSM.raw	9.1588E7	1	1	121	127			PEAKS DB
R.IGKDDLIEKLK	Y	43.39	1270.7496	11	0.3	424.5906	3	19.98	3	4797	211015-MClass-FE2-3uL-LSM.raw	2.7715E5	1	1	137	147			PEAKS DB
total 4 peptides																			

Best Unique PSM (Scan 4797, m/z=424.5906, z=3, RT=19.98, ppm=0.3):

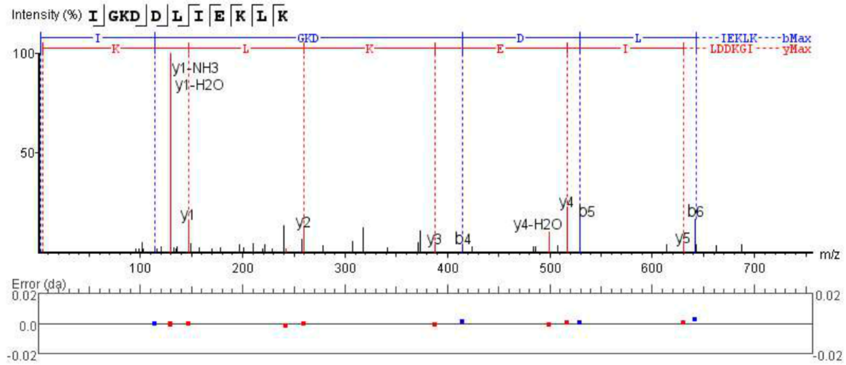

tr|E7D195|E7D195_LATHE

back to list

| Protein Coverage | Supporting Peptides | Best Unique PSM |

Protein Coverage:

```
  1  ARGFAFLSLDPLNQQAKMSIKEKLDRILPLFEKLTTLTRQQLPPDQRDPRLLGVGVLPRGTLFSCFHERHLKEATKLFEI
 81  LYTAADFDDFIKLATQARDVVNEGLFTYAFSVAVVHRDDCRGVTLPPIQEVFPDRFIPAETINLASKESKIKPTEDIVVE
161  IEDTGNILEPEYKLAYFREDIGINAHHWYFHVVYPANWSTELTGKVKDRKGELFYYMHQQMCARYDCERLSNGLNRMIPF
241  HNFEEKLEGYAPHLTSLVSGLHYASRPQGFSLQDLNDVDVQDMERWRERILEAIDLHKVHDAQNNEIPLDEANGANILGA
321  IIEASSDSPNKG
```

65

C Carbamidomethy

O Oxidation (M) (+

Supporting Peptides:

Peptide	Uniq	-10lgP	Mass	Length	ppm	m/z	z	RT	Fraction	Scan	Source File	Area FE2 3uL	#Feature	#Feature FE2 3uL	Start	End	PTM	ASc
R.GTLFSC(+57.02)FHER.H	Y	60.53	1252.5659	10	0.3	418.5294	3	20.71	3	5100	211015-MClass-FE2-3uL-LSM.raw	1.6503E5	1	1	60	69	Carbamidomethylation	C6: ban met atio 000
R.ILEAIDLHK.V	Y	57.31	1050.6073	9	-2.2	526.3098	2	19.54	3	4588	211015-MClass-FE2-3uL-LSM.raw	8.876E5	1	1	290	298		
R.GTLFSC(+57.02)FH.E	Y	53.03	967.4222	8	0.0	484.7184	2	22.90	3	5873	211015-MClass-FE2-3uL-LSM.raw	9.2897E4	1	1	60	67	Carbamidomethylation	C6: ban met atio 000
R.M(+15.99)IPFHNFEEK.L	Y	50.27	1306.6016	10	-0.3	436.5410	3	19.26	3	4444	211015-MClass-FE2-3uL-LSM.raw	4.167E5	2	2	237	246	Oxidation (M)	M1: dati (M) 00.(
R.LLGVGVLPR.G	Y	42.88	922.5964	9	1.5	462.3062	2	22.78	3	5817	211015-MClass-FE2-3uL-LSM.raw	3.1067E6	1	1	51	59		
R.MIPFHNF.E	N	42.05	904.4265	7	1.0	453.2210	2	23.56	3	6060	211015-MClass-FE2-3uL-LSM.raw	1.3967E5	1	1	237	243		
total 6 peptides																		

Best Unique PSM (Scan 5100, m/z=418.5294, z=3, RT=20.71, ppm=0.3):

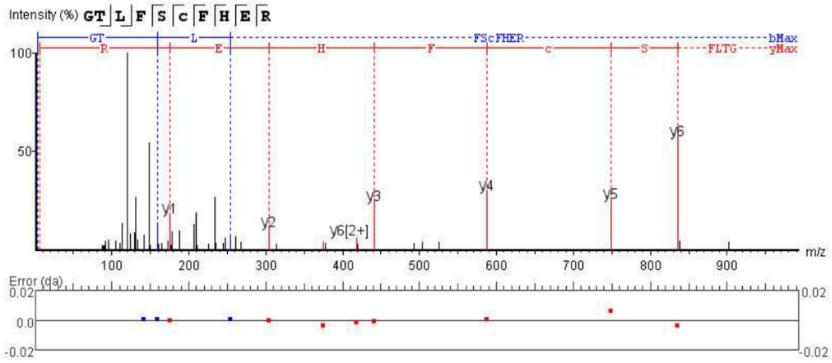

tr|A0A2L2Y2Y0|A0A2L2Y2Y0_PARTP

back to list

| Protein Coverage | Supporting Peptides | Best Unique PSM |

Protein Coverage:

```
  1 MVDQATKDKLEAGFQKLQAATDCKSLLKKYLTKEVFDACKDKKTALGATLLDCIQSGVENLDSGVGIYAPDAEAYTLFAP
 81 IFNPIIEDYHEGFKPTDKHPPTDFGDINTLVNVDPTGEYVVSTRVRCGRSLKGYAFNPCLTEANYKEMEDKVSAIFGSFE
161 GELKGKYHPLTGMDKATQQQLIDDHFLFKEGDRFLQHANACRYWPTGRGIYHNDAKTFLVWCNEEDHLRIISMQKGGDLK
241 TIFQRLVNAVNIIESKLPFSRDDRLGYLTFCPTNLGTTIRASVHIALPKLAKDKKVLEDIAAKFNLQVRGTRGEHTESEG
321 GVYDISNKRRMGLTEYQAVKEMQDGILEMIKMEKAAA
```

331 349

Oxidation (M) (+

Supporting Peptides:

Peptide	Uniq	-10lgP	Mass	Length	ppm	m/z	z	RT	Fraction	Scan	Source File	Area FE2 3uL	#Feature	#Feature FE2 3uL	Start	End	PTM	AScor
R.GEHTESEGGVYDISNK.R	Y	61.87	1720.7540	16	-0.6	574.5916	3	15.81	3	2635	211015-MClass-FE2-3uL-LSM.raw	4.7197E5	1	1	313	328		
R.M(+15.99)GLTEYQAVK.E	Y	58.08	1154.5641	10	0.4	578.2896	2	17.28	3	3389	211015-MClass-FE2-3uL-LSM.raw	3.0007E5	1	1	331	340	Oxidation (M)	M1:Oxdation (M):100.00
H.TESEGGVYDISNK.R	Y	55.04	1397.6310	13	-0.9	699.8221	2	16.79	3	3126	211015-MClass-FE2-3uL-LSM.raw	4.2046E5	1	1	316	328		
K.EM(+15.99)QDGILEM(+15.99)IK.M	Y	51.80	1337.6207	11	-1.2	669.8168	2	20.88	3	5165	211015-MClass-FE2-3uL-LSM.raw	4.0435E5	1	1	341	351	Oxidation (M)	M2:Oxdation (M):100.00 M9:Oxdation (M):100.00
R.ASVHIALPK.L	Y	41.94	934.5599	9	-3.2	468.2857	2	18.63	3	4118	211015-MClass-FE2-3uL-LSM.raw	1.4555E5	1	1	281	289		
**total 5 peptides**																		

Best Unique PSM (Scan 2635, m/z=574.5916, z=3, RT=15.81, ppm=-0.6):

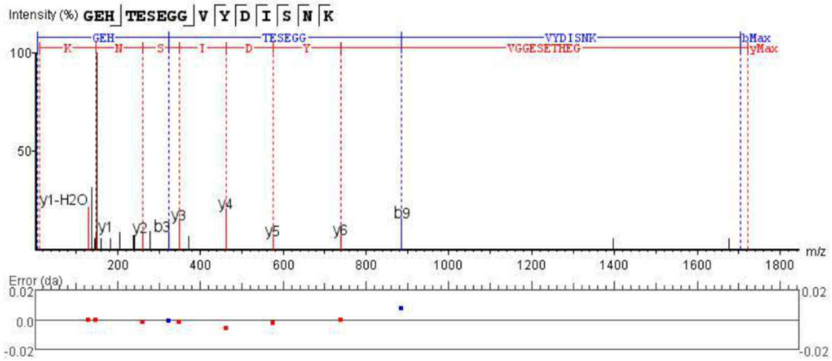

tr|E7D1R3|E7D1R3_LATHE

back to list

| Protein Coverage | Supporting Peptides | Best Unique PSM |

Protein Coverage:

1 HGPRSAKMKALVLLFLIGCASASLYDVLEDAAEEIYHDFMAGMEEAPSLKEALELEGDHFYADQTENDIKTELGKKLRDA

81 LDHILEKIKDAIDNGKTVKEDLQAKLKELKEKMKDLKVDMGNKAKELLEKIKEKSKEFLKELLDKLGLKDDLKRSAADDD

161 LAMLDFNLKDLFKRLKKYLLGKIDKEK**LKAKVEELFGKGSEMADALK**ALIDSKSENYKQKILDLIDRFLGKEDKEFYEQH

241 SISEYWQKIKDYFKDLHIDLKEKYFKFGEWVKTVINKGLDKSKDKLANIKEIAKEFIDHAKGVSKDVAVEALEFLRPYKE

321 DLGNLYDQV

O Oxidation (M) (+

Supporting Peptides:

Peptide	Uniq	-10lgP	Mass	Length	ppm	m/z	z	RT	Fraction	Scan	Source File	Area FE2 3uL	#Feature	#Feature FE2 3uL	Start	End	PTM	AScore
K.VEELFGKGSEM(+15.99)ADALK.A	Y	64.26	1738.8447	16	-0.6	580.6218	3	19.79	3	4697	211015-MClass-FE2-3uL-LSM.raw	1.3078E6	2	2	192	207	Oxidation (M)	M11:Oxidation (M):1000.00
K.AKVEELFGK.G	Y	57.98	1019.5651	9	-0.8	510.7894	2	18.53	3	4041	211015-MClass-FE2-3uL-LSM.raw	5.6766E7	1	1	190	198		
K.VEELFGK.G	Y	46.86	820.4330	7	-1.7	411.2231	2	18.92	3	4257	211015-MClass-FE2-3uL-LSM.raw	8.8546E6	1	1	192	198		
K.LKAKVEELFGK.G	Y	42.64	1260.7441	11	-3.2	421.2540	3	18.90	3	4274	211015-MClass-FE2-3uL-LSM.raw	3.6144E5	1	1	188	198		
**total 4 peptides**																		

Best Unique PSM (Scan 4697, m/z=580.6218, z=3, RT=19.79, ppm=-0.6):

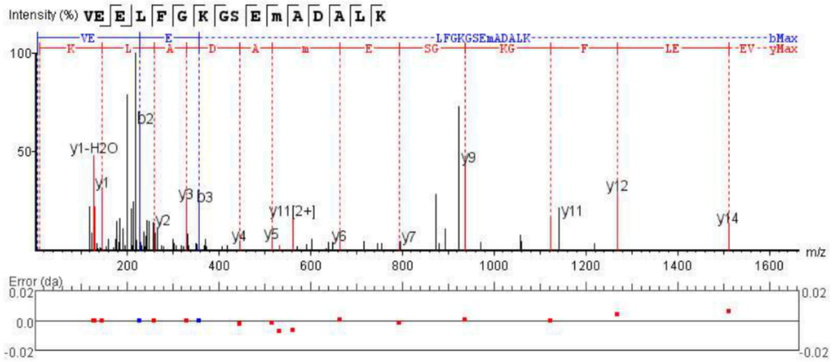

tr|A0A2L2YG81|A0A2L2YG81_PARTP

back to list

| Protein Coverage | Supporting Peptides | Best Unique PSM |

Protein Coverage:

1 MTVKEKQTRVCTLLTHLTSVSKTVVPVEDRDPRLNGIGKLPQGELFSCFHEKGLAEATK**LYETLYAAK**DFEDFMNLAKQA

81 RTFANEGLFVYAVSVAILHRADCRGVTVPPIQEIFPDRFVPTETISLAQKEVANHPDKDVKVEIET**TGNILDPEYK**MSYF

161 REDVGTNAHHWHWHIVYPATWKPEVMGKIKDRKGELFYYMHQQMCARYD

Supporting Peptides:

Peptide	Uniq	-10lgP	Mass	Length	ppm	m/z	z	RT	Fraction	Scan	Source File	Area FE2 3uL	#Feature	#Feature FE2 3uL	Start	End	PTM	AScore	Found By
K.LYETLYAAK.D	N	61.97	1070.5648	9	-0.4	536.2895	2	19.46	3	4565	211015-MClass-FE2-3uL-LSM.raw	7.0952E5	1	1	60	68			PEAKS DB
T.TGNILDPEYK.M	Y	54.04	1148.5713	10	-0.1	575.2928	2	19.52	3	4587	211015-MClass-FE2-3uL-LSM.raw	1.8532E5	1	1	147	156			PEAKS DB
**total 2 peptides**																			

Best Unique PSM (Scan 4587, m/z=575.2928, z=2, RT=19.52, ppm=-0.1):

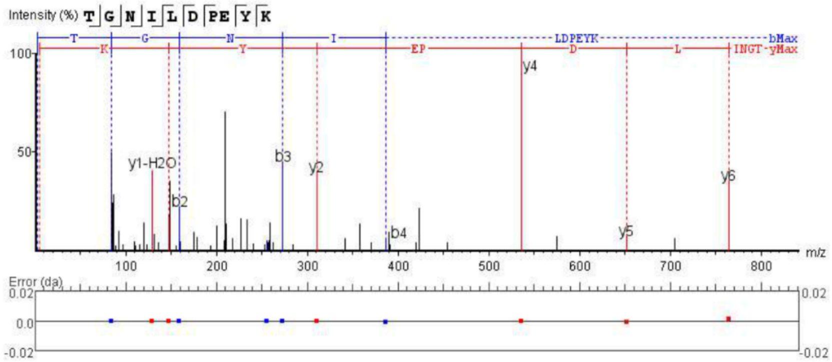

tr|A0A2L2XYG9|A0A2L2XYG9_PARTP

back to list

| Protein Coverage | Supporting Peptides | Best Unique PSM |

Protein Coverage:

33

1 ECEKRREEAQKHETIMKLIHKCK**ENGDYEELQCYK**DSTFCVCYTKDGRPASPNLSNLKECGCFLEKQRKIDSNHENPYLP Carbamidomethy

81 QCQDDGSWNPKQCYDYNNSCWCVDKNGKQVGESKHEGKCLDC

Supporting Peptides:

Peptide	Uniq	-10lgP	Mass	Length	ppm	m/z	z	RT	Fraction	Scan	Source File	Area FE2 3uL	#Feature	#Feature FE2 3uL	Start	End	PTM
K.ENGDYEELQC(+57.02)YK.D	Y	76.08	1546.6245	12	-0.6	774.3191	2	18.43	3	4011	211015-MClass-FE2-3uL-LSM.raw	1.5774E5	1	1	24	35	Carbamidomethylation
total 1 peptides																	

Best Unique PSM (Scan 4011, m/z=774.3191, z=2, RT=18.43, ppm=-0.6):

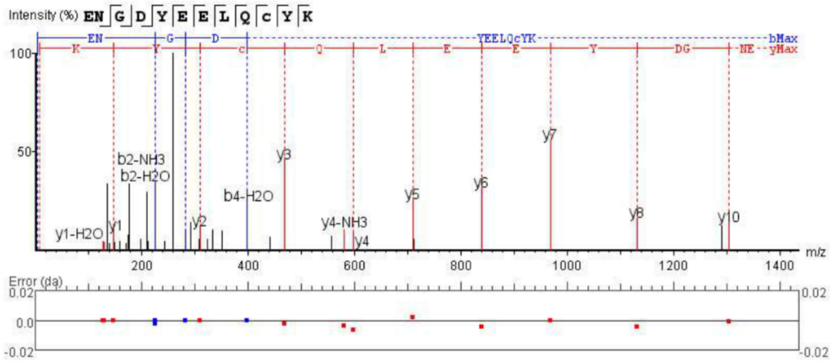

tr|E7D194|E7D194_LATHE

back to list

| Protein Coverage | Supporting Peptides | Best Unique PSM |

Protein Coverage:

1 GTRLRLSHPSGASIMTVKEKQAKILPLFKNLTALSPEPLPEAERDVRLKGVGVLPRGRLFSCFHEDHLGEAQALYETLYE

81 AKDFDDFINLAKQARDIVNEGLFAFALSVVVLHRDDCQGVVLPPIQEVFPDKFVPAETINRALKIDKQSTNEEKVISIQK

161 TGNILDPEYNLAYFREDIGINAHHWHWHLVYPATYRPDFFGKVKDRKGELFYYMHQQMCARYDCDRLSVGLRRMIPFQNF

241 EEKLEGYSAHLTSLISGLNYASRPAGMSLRDVR**EVDVQDMER**WRERILSAIHTGQVIDSNGK**EVPLDLER**GLDILGALIE

321 SSYESLNKGYYGT

Supporting Peptides:

Peptide	Uniq	-10lgP	Mass	Length	ppm	m/z	z	RT	Fraction	Scan	Source File	Area FE2 3uL	#Feature	#Feature FE2 3uL	Start	End	PTM	AScore	Found By
R.EVDVQDMER.W	Y	50.87	1119.4866	9	-0.1	560.7505	2	17.05	3	3259	211015-MClass-FE2-3uL-LSM.raw	2.3113E5	1	1	274	282			PEAKS DB
K.EVPLDLER.G	Y	46.81	969.5131	8	-0.5	485.7636	2	19.91	3	4754	211015-MClass-FE2-3uL-LSM.raw	1.5352E6	1	1	303	310			PEAKS DB
total 2 peptides																			

Best Unique PSM (Scan 3259, m/z=560.7505, z=2, RT=17.05, ppm=-0.1):

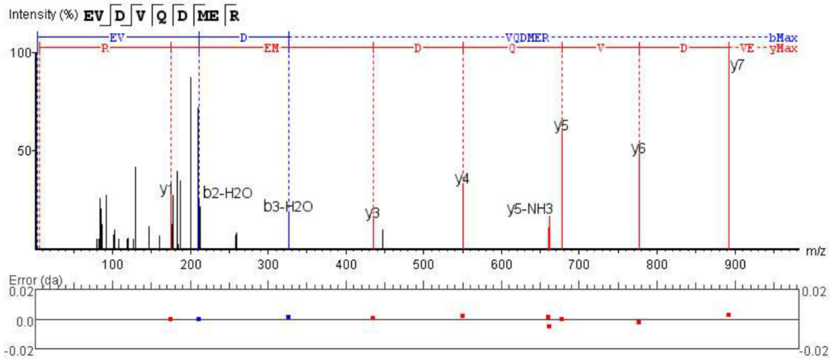

tr|A0A2L2YAY8|A0A2L2YAY8_PARTP

back to list

| Protein Coverage | Supporting Peptides | Best Unique PSM |

Protein Coverage:

1 DHVGTLNAQASCADAVSYCGAKDQKYPDKK**SMGYPFDR**IIRARTIAEFSTQNMNFQEVKIQFKE

Oxidation (M) (+

Supporting Peptides:

Peptide	Uniq	-10lgP	Mass	Length	ppm	m/z	z	RT	Fraction	Scan	Source File	Area FE2 3uL	#Feature	#Feature FE2 3uL	Start	End	PTM	AScore	Found By
K.SM(+15.99)GYPFDR.I	Y	61.26	987.4120	8	0.1	494.7133	2	17.86	3	3703	211015-MClass-FE2-3uL-LSM.raw	3.3522E6	1	1	31	38	Oxidation (M)	M2:Oxidation (M):1000.00	PEAKS DB
K.SMGYPFDR.I	Y	51.81	971.4171	8	-4.4	486.7137	2	20.27	3	4916	211015-MClass-FE2-3uL-LSM.raw	4.0329E5	1	1	31	38			PEAKS DB
total 2 peptides																			

Best Unique PSM (Scan 3703, m/z=494.7133, z=2, RT=17.86, ppm=0.1):

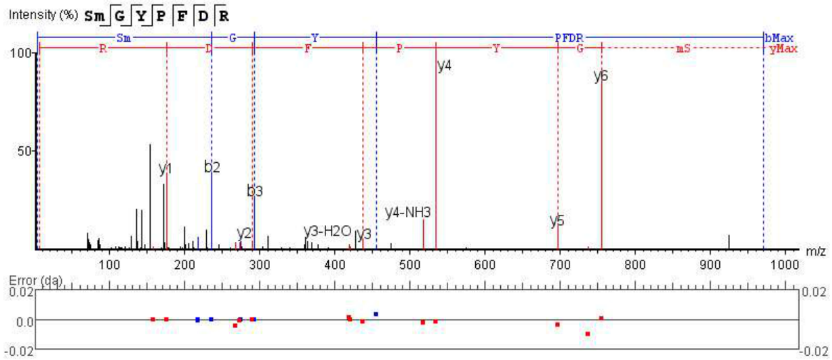

tr|A0A2L2Y372|A0A2L2Y372_PARTP

back to list

| Protein Coverage | Supporting Peptides | Best Unique PSM |

Protein Coverage:

1 MRVFLALFVITLCVLATNA**ALTCFGDEEK**CSAEECCIQVGNTPVGICKKKHEIDQICEMKPIKHLFKKHVFKVRCPCKDE

81 LKCVSSKGGILGKIAGKCQENSEEEE

Carbamidomethy

Supporting Peptides:

Peptide	Uniq	-10lgP	Mass	Length	ppm	m/z	z	RT	Fraction	Scan	Source File	Area FE2 3uL	#Feature	#Feature FE2 3uL	Start	End	PTM	ASc
A.ALTC(+57.02)FGDEEK.C	Y	59.35	1168.5070	10	0.4	585.2610	2	17.73	3	3639	211015-MClass-FE2-3uL-LSM.raw	3.8482E5	1	1	20	29	Carbamidomethylation	C4: bam met atio 000
total 1 peptides																		

Best Unique PSM (Scan 3639, m/z=585.2610, z=2, RT=17.73, ppm=0.4):

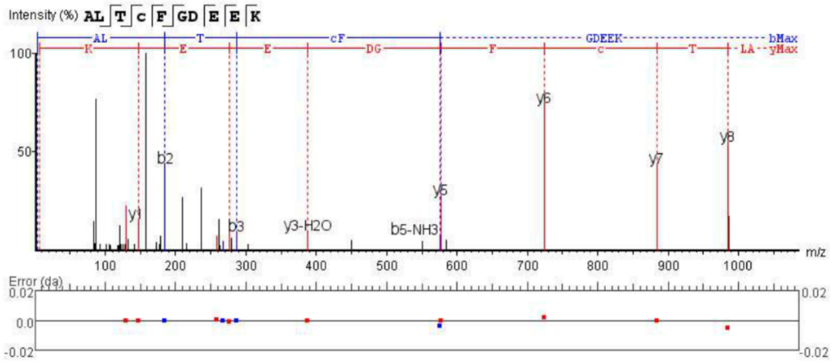

tr|A0A2L2Y2N9|A0A2L2Y2N9_PARTP

back to list

| Protein Coverage | Supporting Peptides | Best Unique PSM |

Protein Coverage:

1 MRVFLALLVITLCALAANA**ALTCFGDEEK**CSAEECCIQVGNTPVGICKKKHEIDQICEMKPIKHLFKKHVFKVRCPCKDE C Carbamidomethy

81 LKCVSSKGVIIGKFAGKCQENSGEEEE

Supporting Peptides:

Peptide	Uniq	-10lgP	Mass	Length	ppm	m/z	z	RT	Fraction	Scan	Source File	Area FE2 3uL	#Feature	#Feature FE2 3uL	Start	End	PTM	ASc
A.ALTC(+57.02)FGDEEK.C	Y	59.35	1168.5070	10	0.4	585.2610	2	17.73	3	3639	211015-MClass-FE2-3uL-LSM.raw	3.8482E5	1	1	20	29	Carbamidomethylation	C4: bam met atio 000
**total 1 peptides**																		

Best Unique PSM (Scan 3639, m/z=585.2610, z=2, RT=17.73, ppm=0.4):

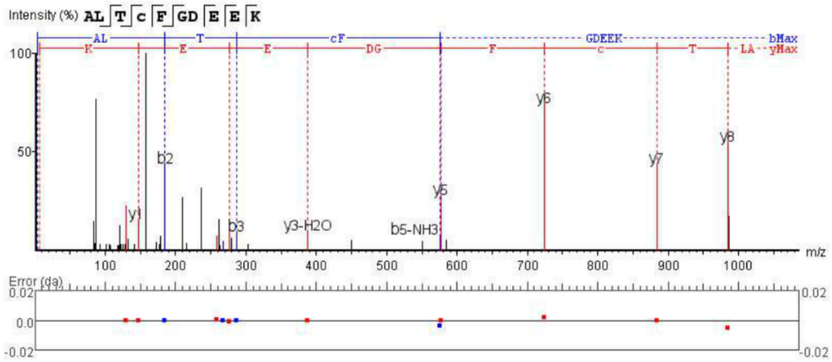

tr|A0A2L2Y2N1|A0A2L2Y2N1_PARTP

back to list

| Protein Coverage | Supporting Peptides | Best Unique PSM |

Protein Coverage:

1 MRVFLALLVITLCALAANA**ALTCFGDEEK**CSAEECCIQVGNTPVGICKKKHEIDQICEMKPIKHLFKKHVFKVRCPCKDE C Carbamidomethy

81 LKCVSSKGGIIGKFAGKCQENSGEEEE

Supporting Peptides:

Peptide	Uniq	-10lgP	Mass	Length	ppm	m/z	z	RT	Fraction	Scan	Source File	Area FE2 3uL	#Feature	#Feature FE2 3uL	Start	End	PTM	ASc
A.ALTC(+57.02)FGDEEK.C	Y	59.35	1168.5070	10	0.4	585.2610	2	17.73	3	3639	211015-MClass-FE2-3uL-LSM.raw	3.8482E5	1	1	20	29	Carbamidomethylation	C4: bam met atio 000
**total 1 peptides**																		

Best Unique PSM (Scan 3639, m/z=585.2610, z=2, RT=17.73, ppm=0.4):

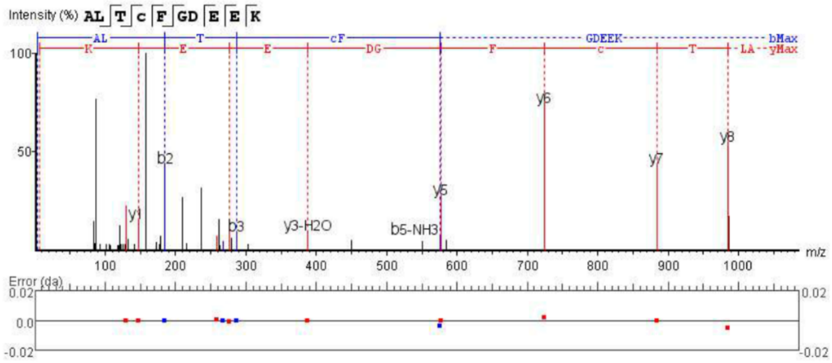

tr|A0A2L2YL93|A0A2L2YL93_PARTP back to list

| Protein Coverage | Supporting Peptides | Best Unique PSM |

Protein Coverage:

25 27

1 MKLIPKCKDKGDYEELQCYNDSK**FCVCYDK**KGHPASPILSKLTDCGCFLERKRKIDSNPGGAAAAAAAAAEAGSSPVYLSL   Carbamidomethy

81 GCSFVHVVFFFGVLVLCVY

Supporting Peptides:

Peptide	Uniq	-10lgP	Mass	Length	ppm	m/z	z	RT	Fraction	Scan	Source File	Area FE2 3uL	#Feature	#Feature FE2 3uL	Start	End	PTM
K.FC(+57.02)VC(+57.02)YDK.K	Y	52.42	990.3939	7	-0.8	496.2039	2	17.79	3	3681	211015-MClass-FE2-3uL-LSM.raw	4.5462E5	1	1	24	30	Carbamidomethylation
**total 1 peptides**																	

Best Unique PSM (Scan 3681, m/z=496.2039, z=2, RT=17.79, ppm=-0.8):

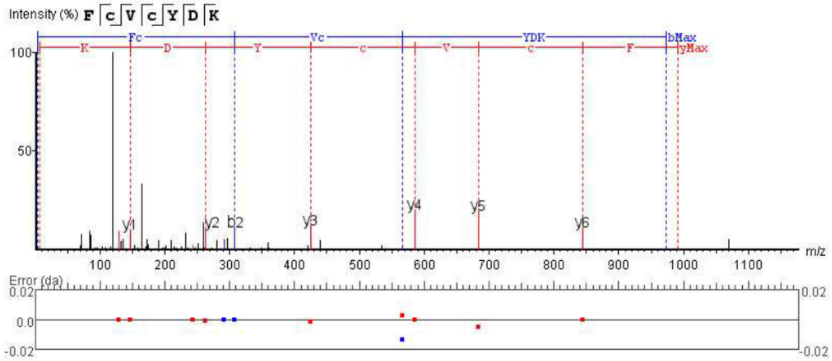

tr|A0A2L2Z3D2|A0A2L2Z3D2_PARTP back to list

| Protein Coverage | Supporting Peptides | Best Unique PSM |

Protein Coverage:

1 KAVTEILEKLKDARENGKVVK**EDILEKLK**EIRQMLKDLKQDISILAKELLDKLMD

Supporting Peptides:

Peptide	Uniq	-10lgP	Mass	Length	ppm	m/z	z	RT	Fraction	Scan	Source File	Area FE2 3uL	#Feature	#Feature FE2 3uL	Start	End	PTM	AScore	Found By
K.EDILEKLK.E	Y	44.99	986.5648	8	-2.9	494.2882	2	20.24	3	4908	211015-MClass-FE2-3uL-LSM.raw	7.4344E5	1	1	22	29			PEAKS DB
**total 1 peptides**																			

Best Unique PSM (Scan 4908, m/z=494.2882, z=2, RT=20.24, ppm=-2.9):

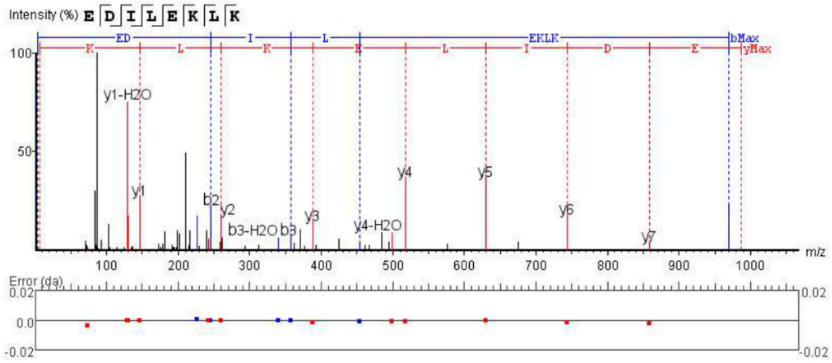

tr|A0A2L2ZCD3|A0A2L2ZCD3_PARTP

back to list

| Protein Coverage | Supporting Peptides | Best Unique PSM |

Protein Coverage:

37 39

1 CDCVRQKDADIPAGATYVPKCAKDGYFPSKQCSD**DECWCVDK**NRPCGGWSISLLLNAWCTPIGSYHWLNYTSML

Carbamidomethy

Supporting Peptides:

Peptide	Uniq	-10lgP	Mass	Length	ppm	m/z	z	RT	Fraction	Scan	Source File	Area FE2 3uL	#Feature	#Feature FE2 3uL	Start	End	PTM
D.DEC(+57.02)WC(+57.02)VDK.N	Y	41.99	1110.4110	8	-0.2	556.2126	2	17.43	3	3479	211015-MClass-FE2-3uL-LSM.raw	3.9213E5	1	1	35	42	Carbamidomethylat
total 1 peptides																	

Best Unique PSM (Scan 3479, m/z=556.2126, z=2, RT=17.43, ppm=-0.2):

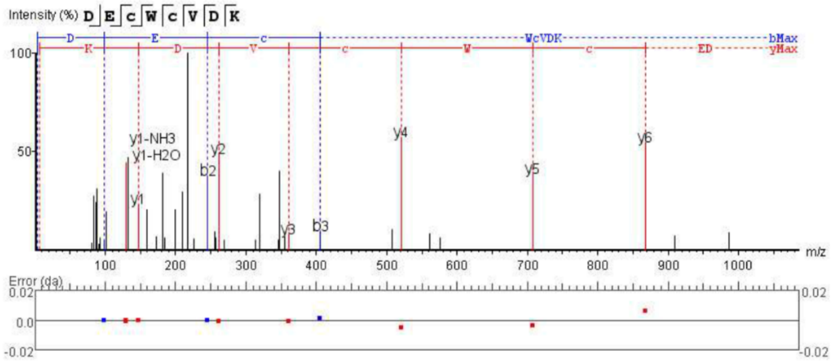

tr|A0A2L2XZN5|A0A2L2XZN5_PARTP

back to list

| Protein Coverage | Supporting Peptides | Best Unique PSM |

Protein Coverage:

1 MLKIVGILALCLFATAFAADDEKEPSACELDRARRLNATLTESILHLIPECEENGDYAALQCFTANDWCVCYRRNGDNIN

Carbamidomethy

125 127

81 TPSKNIKACDCVRQKDDAITAGDTYIPKCDKNGYFQSKQCSN**DECWCVDK**NGKVLTDPKTGGVDC

Supporting Peptides:

Peptide	Uniq	-10lgP	Mass	Length	ppm	m/z	z	RT	Fraction	Scan	Source File	Area FE2 3uL	#Feature	#Feature FE2 3uL	Start	End	PTM
N.DEC(+57.02)WC(+57.02)VDK.N	Y	41.99	1110.4110	8	-0.2	556.2126	2	17.43	3	3479	211015-MClass-FE2-3uL-LSM.raw	3.9213E5	1	1	123	130	Carbamidomethylat
total 1 peptides																	

Best Unique PSM (Scan 3479, m/z=556.2126, z=2, RT=17.43, ppm=-0.2):

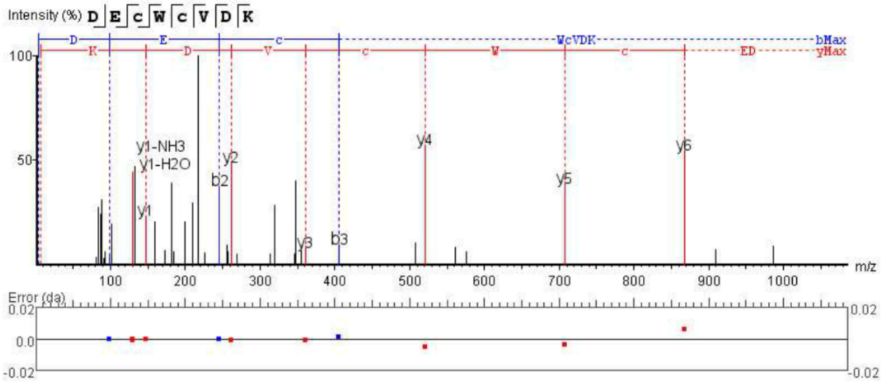

Peptide List

Prepared with PEAKS ™ (bioinfor.com)

Supplementary materials File S3 Shotgun proteomics of *Latrodectus geometricus*

Figure 1. False discovery rate (FDR) curve. X axis is the number of peptides being kept. Y axis is the corresponding FDR.

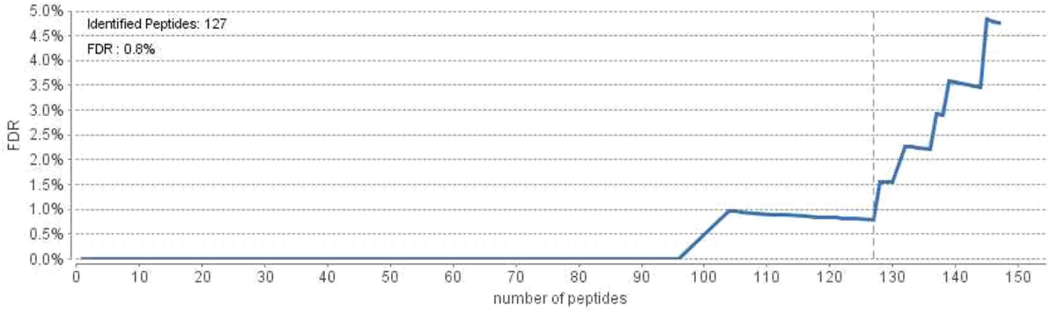

Figure 2. PSM score distribution. **(a)** Distribution of PEAKS peptide score; **(b)** Scatterplot of PEAKS peptide score versus precursor mass error.

(a) **(b)**

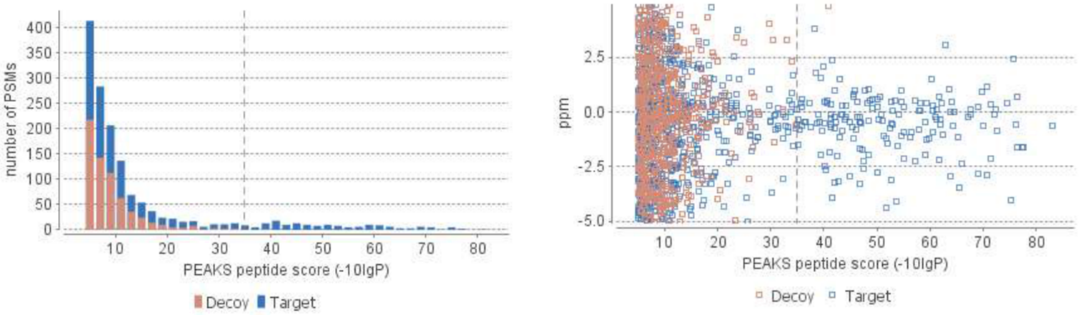

Table 1. Statistics of data.

	#Scans		#Features	Identified			#Peptides	#Sequences	#Proteins*		
	MS1	MS/MS		#PSMs	#Scans	#Features**			Groups	All	Top
Total	7240	4510	34757	167	236	143	128	102	30	41	35
FE2 3uL	7240	4510	34757	167	236	143	128	102	30	41	35

* proteins with significant peptides are used in counts.
** features are identified by DB search only.

Figure 3. Sample overlap for Proteins and Peptides (up to 8 samples). **(a)** All Proteins; **(b)** Top Proteins; **(c)** Peptides;

(a) Not applicable to only one sample

(b) Not applicable to only one sample

(c) Not applicable to only one sample

Figure 4. Distribution of peptide feature detection. **(a)** Feature m/z distribution; **(b)** Feature RT distribution.

(a) **(b)**

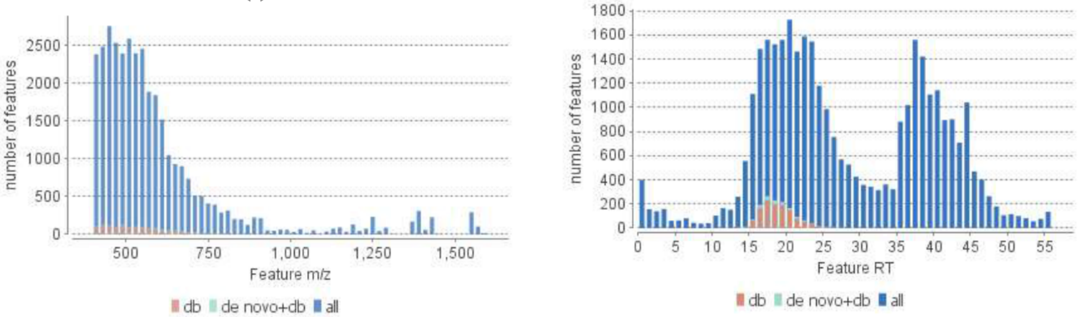

Figure 5. Distribution of identified peptide features. **(a)** Feature abundance distribution; **(b)** *De novo* sequencing validation.

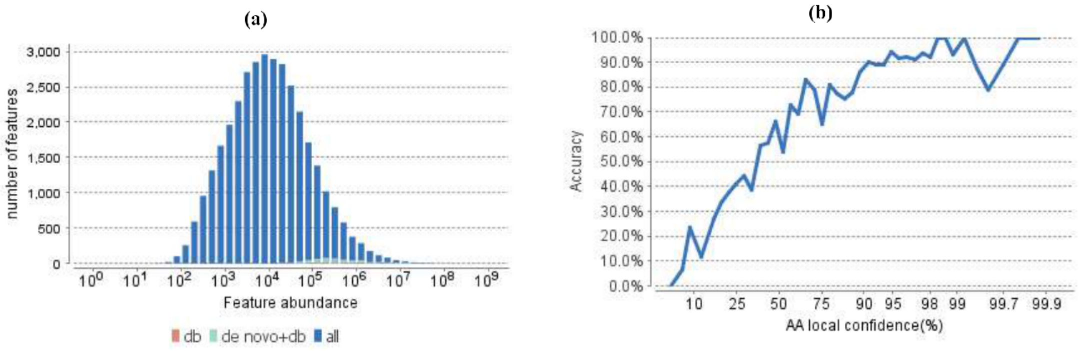

Table 2. Result filtration parameters.

Peptide -10lgP	≥34.9
PTM Ascore	≥0
Peptide mutation ion intensity	≥0%
Protein -10lgP	≥20
Proteins unique peptides	≥1
De novo score(%)	≥50%

Table 3. Statistics of filtered result.

FDR (Peptide-Spectrum Matches)	0.6%
FDR (Peptide Sequences)	0.8%
FDR (Protein Group)	0.0%
De Novo Only Spectra	484

Table 4. PTM profile.

Name	ΔMass	Position	#PSM	-10lgP	Abundance	AScore
Methylation(others)	14.02	DE	25	75.81	4.8E8	24.44
Deamidation	.98	N	17	83.07	1.21E6	1000.00
Oxidation	15.99	M	11	64.26	1.11E6	1000.00
Carbamidomethyl	57.02	C	11	76.08	1.58E5	1000.00
Carbamidomethyl	57.02	DK,N-term	8	74.42	2.07E5	12.28
Formylation	27.99	K,N-term	8	70.82	2.66E5	14.63
Methylation(KR)	14.02	KR	6	69.95	2.01E6	71.72
Pyro-glu from E	-18.01	N-term	5	62.25	5.37E5	1000.00
Acetylation	42.01	K	4	59.01	3.93E5	100.91
Acetylation	42.01	N-term	4	62.88	5.62E4	1000.00

3. Experiment Control

Figure 6. Precursor mass error of peptide-spectrum matches (PSM) in filtered result. **(a)** Distribution of precursor mass error in ppm; **(b)** Scatterplot of precursor m/z versus precursor mass error in ppm.

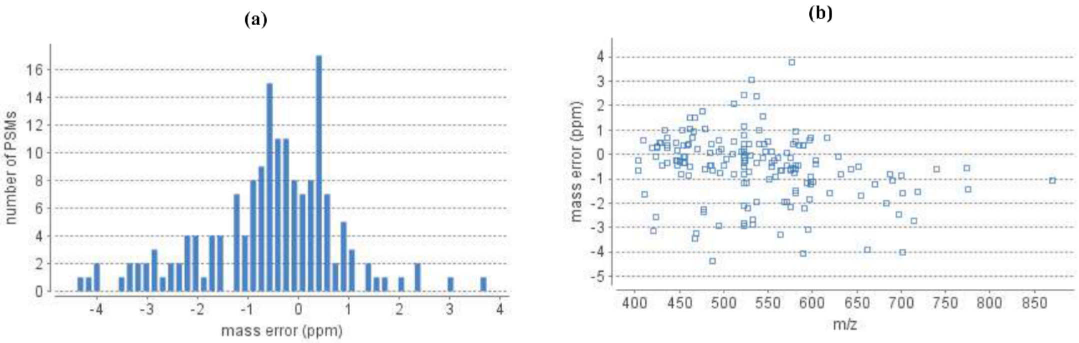

Table 5. Number of identified peptides in each sample by the number of missed cleavages.

Missed Cleavages	0	1	2	3	4+
FE2 3uL	58	55	15	0	0

4. Other Information

Table 6. Search parameters.

Query Type: Homology Match
Fixed Modifications:
Carbamidomethylation: 57.02
Variable Modifications:
Oxidation (M): 15.99
Fragment ion tolerance: 0.015
L equals I: true
Q equals K: true
Report number: 1
Maximum # of PTMs: 3
De novo score(%) threshold: 15
Peptide hit threshold (-10logP): 30.0
Peaks run ID: 82

Table 7. Instrument parameters.

Fractions: 211015-MClass-FE2-3uL-LSM.raw
Ion Source: ESI(nano-spray)
Fragmentation Mode: high energy CID (y and b ions)
MS Scan Mode: FT-ICR/Orbitrap
MS/MS Scan Mode: Linear Ion Trap

Merge Options: merged
Precursor Options: no correction
Charge Options: no correction
Filter Options: no filter
Process: true
Associate chimera: no

Protein List

Protein Accession Contains:
Protein Description Contains:
Protein Sample Area >=
Protein PTM Contains:
Protein Mutations: False

Protein Group	Protein ID	Accession	-10lgP	Coverage (%)	Coverage (%) FE2 3uL	Area FE2 3uL	#Peptides	#Unique	#Spec FE2 3uL	PTM	Avg. Mass	Description
**1**	19424	tr|E7D1U1|E7D1U1_LATHE	236.81	31	31	3.0189E7	19	13	53	Y	34753	Uncharacterized protein (Fragment) OS=Latrodectus hesperus OX=256737 PE=2 SV=1
**3**	19930	tr|A0A2L2XZE1|A0A2L2XZE1_PARTP	221.04	23	23	1.9101E7	16	3	47	Y	30585	Laminin subunit alpha-2 (Fragment) OS=Parasteatoda tepidariorum OX=114398 PE=2 SV=1
**2**	19944	tr|A0A2L2YMR4|A0A2L2YMR4_PARTP	220.19	31	31	6.2295E7	11	7	52	Y	17115	Laminin subunit alpha-2 (Fragment) OS=Parasteatoda tepidariorum OX=114398 PE=2 SV=1
**4**	19933	tr|A0A2L2XZF8|A0A2L2XZF8_PARTP	201.10	21	21	7.9455E4	12	1	41	Y	22436	Laminin subunit alpha-2 (Fragment) OS=Parasteatoda tepidariorum OX=114398 PE=2 SV=1
**6**	19931	tr|E7D1R3|E7D1R3_LATHE	177.90	22	22	7.0953E7	12	11	22	Y	37977	Uncharacterized protein (Fragment) OS=Latrodectus hesperus OX=256737 PE=2 SV=1
**5**	19949	tr|A0A2L2Z8N3|A0A2L2Z8N3_PARTP	160.68	39	39	4.2832E5	6	3	32	Y	10555	Laminin subunit alpha-2 (Fragment) OS=Parasteatoda tepidariorum OX=114398 PE=2 SV=1
**7**	19929	tr|E7D195|E7D195_LATHE	160.66	20	20	6.8383E6	10	8	12	Y	38031	Hemocyanin subunit G (Fragment) OS=Latrodectus hesperus OX=256737 PE=2 SV=1
**10**	19934	tr|A0A2L2YG81|A0A2L2YG81_PARTP	150.56	24	24	3.011E6	6	4	7	Y	24067	Hemocyanin subunit E OS=Parasteatoda tepidariorum OX=114398 PE=2 SV=1
**9**	19935	tr|A0A2L2Y2X9|A0A2L2Y2X9_PARTP	149.68	11	11	2.8956E6	8	5	9	Y	70981	Hemocyanin subunit E OS=Parasteatoda tepidariorum OX=114398 PE=2 SV=1
**9**	19936	tr|A0A2L2Y308|A0A2L2Y308_PARTP	149.68	11	11	2.8956E6	8	5	9	Y	71157	Hemocyanin subunit E OS=Parasteatoda tepidariorum OX=114398 PE=2 SV=1
**8**	19943	tr|A0A2L2Z761|A0A2L2Z761_PARTP	133.18	39	39	1.1103E7	6	4	10	Y	8218	Uncharacterized protein (Fragment) OS=Parasteatoda tepidariorum OX=114398 PE=2 SV=1
**11**	19928	tr|A0A2L2Y2Y0|A0A2L2Y2Y0_PARTP	130.61	13	13	5.4991E5	5	2	5	Y	40057	Arginine kinase OS=Parasteatoda tepidariorum OX=114398 PE=2 SV=1
**12**	19938	tr|A0A2L2Y7T9|A0A2L2Y7T9_PARTP	120.31	9	9	3.0521E5	4	1	4	Y	40287	Arginine kinase (Fragment) OS=Parasteatoda tepidariorum OX=114398 PE=2 SV=1
**15**	19937	tr|E7D194|E7D194_LATHE	87.65	7	7	1.7663E6	3	2	3	N	38030	Hemocyanin subunit D (Fragment) OS=Latrodectus hesperus OX=256737 PE=2 SV=1
**16**	19954	tr|A0A2L2YAY8|A0A2L2YAY8_PARTP	81.05	12	12	3.7555E6	2	2	3	Y	7263	Hemocyanin subunit F (Fragment) OS=Parasteatoda tepidariorum OX=114398 PE=2 SV=1
**17**	19950	tr|A0A2L2XYG9|A0A2L2XYG9_PARTP	76.08	10	10	3.0495E5	1	1	2	Y	14239	U24-ctenitoxin-Pn1a (Fragment) OS=Parasteatoda tepidariorum OX=114398 PE=2 SV=1
**14**	20006	tr|A0A2L2Y4H8|A0A2L2Y4H8_PARTP	76.05	3	3	1.1146E6	3	1	4	Y	72263	Hemocyanin C chain OS=Parasteatoda tepidariorum OX=114398 PE=2 SV=1
**13**	19952	tr|A0A2L2Z5H2|A0A2L2Z5H2_PARTP	69.53	11	11	9.4054E6	2	1	4	N	9938	Uncharacterized protein (Fragment) OS=Parasteatoda tepidariorum OX=114398 PE=2 SV=1
**18**	20029	tr|A0A2L2XZN5|A0A2L2XZN5_PARTP	63.63	14	14	7.0612E5	2	2	2	Y	16028	U24-ctenitoxin-Pn1a OS=Parasteatoda tepidariorum OX=114398 PE=2 SV=1
**28**	19960	tr|A0A2L2Y372|A0A2L2Y372_PARTP	59.35	9	9	3.8482E5	1	1	1	Y	11764	Uncharacterized protein OS=Parasteatoda tepidariorum OX=114398 PE=2 SV=1
**28**	19961	tr|A0A2L2Y2N9|A0A2L2Y2N9_PARTP	59.35	9	9	3.8482E5	1	1	1	Y	11805	Uncharacterized protein OS=Parasteatoda tepidariorum OX=114398 PE=2 SV=1
**28**	19962	tr|A0A2L2Y2N1|A0A2L2Y2N1_PARTP	59.35	9	9	3.8482E5	1	1	1	Y	11763	Uncharacterized protein OS=Parasteatoda tepidariorum OX=114398 PE=2 SV=1
**29**	19955	tr|A0A2L2YL93|A0A2L2YL93_PARTP	52.42	7	7	4.5462E5	1	1	1	Y	10706	U24-ctenitoxin-Pn1a OS=Parasteatoda tepidariorum OX=114398 PE=2 SV=1
**22**	19953	tr|A0A2L2Z3D2|A0A2L2Z3D2_PARTP	44.99	15	15	7.4344E5	1	1	1	N	6421	Laminin subunit alpha-2 (Fragment) OS=Parasteatoda tepidariorum OX=114398 PE=2 SV=1
**27**	23316	tr|E7D1N7|E7D1N7_LATHE	42.93	4	4	4.9227E4	1	1	1	Y	29705	Putative serine proteinase inhibitor (Fragment) OS=Latrodectus hesperus OX=256737 PE=2 SV=1
**23**	19957	tr|A0A2L2Z3P8|A0A2L2Z3P8_PARTP	41.26	17	17	5.0592E5	1	1	1	N	6379	Laminin subunit alpha-2 (Fragment) OS=Parasteatoda tepidariorum OX=114398 PE=2 SV=1
**24**	20062	tr|A0A2L2Y3F9|A0A2L2Y3F9_PARTP	40.62	4	4	5.9589E4	1	1	1	Y	45294	Metalloendopeptidase OS=Parasteatoda tepidariorum OX=114398 PE=2 SV=1

total 35 proteins

Protein Group	Protein ID	Accession	-10lgP	Coverage (%)	Coverage (%) FE2 3uL	Area FE2 3uL	#Peptides	#Unique	#Spec FE2 3uL	PTM	Avg. Mass	Description
**25**	19969	tr|A0A2L2YQH5|A0A2L2YQH5_PARTP	39.91	3	3	6.935E6	1	1	1	N	32289	DNA repair endonuclease XPF (Fragment) OS=Parasteatoda tepidariorum OX=114398 PE=2 SV=1
**26**	20018	tr|A0A2L2Y4C8|A0A2L2Y4C8_PARTP	39.43	4	4	2.3804E7	1	1	1	N	25654	MICOS complex subunit (Fragment) OS=Parasteatoda tepidariorum OX=114398 PE=2 SV=1
**30**	20083	tr|A0A2L2Z6L1|A0A2L2Z6L1_PARTP	36.64	6	6	1.2076E5	1	1	1	N	12960	Laminin subunit alpha-2 (Fragment) OS=Parasteatoda tepidariorum OX=114398 PE=2 SV=1
**21**	20081	tr|A0A2L2YL07|A0A2L2YL07_PARTP	35.44	12	12	3.7417E5	1	1	1	N	10391	Laminin subunit alpha-2 (Fragment) OS=Parasteatoda tepidariorum OX=114398 PE=2 SV=1
**20**	19981	tr|A0A2L2Y270|A0A2L2Y270_PARTP	34.98	1	1	3.8316E4	1	1	1	Y	200760	VWFD domain-containing protein (Fragment) OS=Parasteatoda tepidariorum OX=114398 PE=2 SV=1
**20**	19982	tr|A0A2L2Y1P7|A0A2L2Y1P7_PARTP	34.98	1	1	3.8316E4	1	1	1	Y	202874	VWFD domain-containing protein (Fragment) OS=Parasteatoda tepidariorum OX=114398 PE=2 SV=1
**20**	19983	tr|A0A2L2XZR6|A0A2L2XZR6_PARTP	34.98	1	1	3.8316E4	1	1	1	Y	204188	VWFD domain-containing protein (Fragment) OS=Parasteatoda tepidariorum OX=114398 PE=2 SV=1
**202**	19958	tr|A0A2L2Y9R4|A0A2L2Y9R4_PARTP	34.93	3	3	1.3002E5	1	1	1	N	36824	Putative serine protease (Fragment) OS=Parasteatoda tepidariorum OX=114398 PE=2 SV=1

total 35 proteins

tr|E7D1U1|E7D1U1_LATHE

back to list

| Protein Coverage | Supporting Peptides | Best Unique PSM |

Protein Coverage:

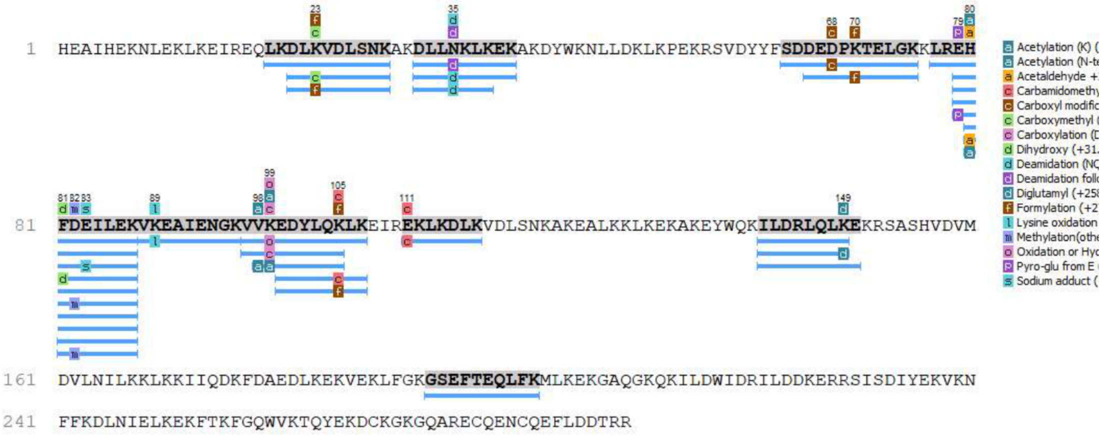

Supporting Peptides:

Peptide	Uniq	-10lgP	Mass	Length	ppm	m/z	z	RT	Fraction	Scan	Source File	Area FE2 3uL	#Feature	#Feature FE2 3uL	Start	End	PTM
R.EHFDEILEK.V	N	76.48	1158.5557	9	-1.6	580.2842	2	19.50	3	4605	211015-MClass-FE2-3uL-LSM.raw	4.0773E7	2	2	79	87	
E.HFD(+14.02)EILEK.V	N	75.81	1043.5287	8	2.4	522.7729	2	17.88	3	3717	211015-MClass-FE2-3uL-LSM.raw	4.8719E8	3	3	80	87	Methylation(others)
K.VVK(+15.99)EDYLQKLK.E	Y	72.69	1377.7867	11	-0.8	460.2691	3	17.48	3	3502	211015-MClass-FE2-3uL-LSM.raw	1.8679E6	2	2	97	107	Oxidation or Hydroxylation
K.EDYLQK(+57.02)LK.E	Y	72.36	1092.5815	8	0.1	547.2981	2	17.09	3	3311	211015-MClass-FE2-3uL-LSM.raw	8.7118E4	1	1	100	107	Carbamidomethylatio (DHKE, X@N-term)
K.EDYLQK(+27.99)LK.E	Y	70.82	1063.5549	8	-2.9	532.7832	2	20.26	3	4922	211015-MClass-FE2-3uL-LSM.raw	2.6601E5	1	1	100	107	Formylation
K.LREHFDEILEK.V	N	66.00	1427.7408	11	-2.4	476.9198	3	18.95	3	4273	211015-MClass-FE2-3uL-LSM.raw	6.9047E6	2	2	77	87	

total 29 peptides

Peptide	Uniq	-10lgP	Mass	Length	ppm	m/z	z	RT	Fraction	Scan	Source File	Area FE2 3uL	#Feature	#Feature FE2 3uL	Start	End	PTM
K.DLK(+27.99)VDLSNK.A	Y	63.88	1058.5608	9	0.0	530.2877	2	17.77	3	3680	211015-MClass-FE2-3uL-LSM.raw	2.4263E5	1	1	21	29	Formylation
K.DLLN(+.98)KLKEK.A	Y	62.53	1100.6440	9	-1.0	551.3287	2	19.29	3	4446	211015-MClass-FE2-3uL-LSM.raw	3.7608E5	1	1	32	40	Deamidation (NQ)
R.EHFDE(+21.98)ILEK.V	N	61.71	1180.5376	9	-2.2	591.2748	2	19.04	3	4329	211015-MClass-FE2-3uL-LSM.raw	7.6886E4	1	1	79	87	Sodium adduct
K.DLLN(+15.00)KLKEK.A	Y	60.50	1114.6598	9	-0.9	558.3367	2	18.80	3	4205	211015-MClass-FE2-3uL-LSM.raw	9.1849E5	1	1	32	40	Deamidation followed by a methylation
R.E(-18.01)HFDEILEK.V	N	60.48	1140.5450	9	0.1	571.2798	2	22.14	3	5629	211015-MClass-FE2-3uL-LSM.raw	3.104E5	2	2	79	87	Pyro-glu from E
V.V(+42.01)K(+42.01)EDYLQK.L	Y	58.28	1105.5656	8	0.1	553.7901	2	19.91	3	4759	211015-MClass-FE2-3uL-LSM.raw	3.0524E6	1	1	98	105	Acetylation (N-term); Acetylation (K)
D.DEDPK(+27.99)TELGK.K	N	57.16	1158.5404	10	0.5	580.2778	2	16.58	3	3024	211015-MClass-FE2-3uL-LSM.raw	5.1117E4	1	1	66	75	Formylation
K.GSEFTEQLFK.M	Y	56.29	1184.5713	10	-1.2	593.2922	2	22.79	3	5838	211015-MClass-FE2-3uL-LSM.raw	1.3857E5	1	1	193	202	
K.VK(-1.03)EAIENGK.V	Y	50.19	985.5080	9	0.6	493.7616	2	20.54	3	5024	211015-MClass-FE2-3uL-LSM.raw	1.3893E5	1	1	88	96	Lysine oxidation to aminoadipic semialdehyde
H.FDEILEK.V	N	49.23	892.4542	7	0.2	447.2344	2	18.91	3	4244	211015-MClass-FE2-3uL-LSM.raw	9.1588E7	1	1	81	87	
R.EHF(+31.99)DEILEK.V	N	49.18	1190.5455	9	0.6	596.2804	2	21.24	3	5317	211015-MClass-FE2-3uL-LSM.raw	1.0354E5	2	2	79	87	Dihydroxy
K.DLK(+58.01)VDLSNK.A	Y	48.26	1088.5713	9	0.4	545.2932	2	16.58	3	3019	211015-MClass-FE2-3uL-LSM.raw	2.6751E4	1	1	21	29	Carboxymethyl (KW, X@N-term)
R.E(+57.02)KLKDLK.V	N	47.62	929.5546	7	1.0	465.7850	2	17.79	3	3690	211015-MClass-FE2-3uL-LSM.raw	8.776E4	1	1	111	117	Carbamidomethylatio (DHKE, X@N-term)
K.DLLN(+.98)KLK.E	Y	47.40	843.5065	7	-2.6	422.7595	2	20.24	3	4892	211015-MClass-FE2-3uL-LSM.raw	4.0543E6	1	1	32	38	Deamidation (NQ)
E.H(+26.02)FDEILEK.V	N	45.26	1055.5287	8	-0.7	528.7712	2	19.47	3	4564	211015-MClass-FE2-3uL-LSM.raw	1.5334E6	1	1	80	87	Acetaldehyde +26
K.VVK(+43.99)EDYLQK.L	Y	44.94	1164.6027	9	-0.4	583.3083	2	18.01	3	3763	211015-MClass-FE2-3uL-LSM.raw	1.1078E6	1	1	97	105	Carboxylation (DKW)
Q.LKDLKVDLSNK.A	Y	44.21	1271.7449	11	0.3	424.9224	3	16.59	3	3038	211015-MClass-FE2-3uL-LSM.raw	1.8857E5	1	1	19	29	
E.H(+42.01)FDEILEK.V	N	41.55	1071.5237	8	2.4	536.7704	2	21.76	3	5524	211015-MClass-FE2-3uL-LSM.raw	6.3841E5	2	2	80	87	Acetylation (N-term)

total 29 peptides

Peptide	Uniq	-10lgP	Mass	Length	ppm	m/z	z	RT	Fraction	Scan	Source File	Area FE2 3uL	#Feature	#Feature FE2 3uL	Start	End	PTM
F.SDDED(+43.04)PKTELGK.K	Y	41.25	1375.6466	12	0.3	459.5563	3	16.23	3	2840	211015-MClass-FE2-3uL-LSM.raw	1.7682E5	1	1	64	75	Carboxyl modification with ethanolamine
K.ILDRLQLK.E	Y	39.11	997.6284	8	-0.5	499.8212	2	19.12	3	4367	211015-MClass-FE2-3uL-LSM.raw	4.2353E6	1	1	142	149	
K.ILDRLQLK(+258.09).E	Y	39.06	1255.7135	8	-0.1	628.8640	2	19.24	3	4432	211015-MClass-FE2-3uL-LSM.raw	1.6797E6	1	1	142	149	Diglutamyl
K.ILDRLQLKE.K	Y	37.53	1126.6710	9	-0.7	564.3424	2	19.37	3	4485	211015-MClass-FE2-3uL-LSM.raw	1.1632E7	1	1	142	150	
H.FD(+14.02)EILEK.V	N	35.07	906.4698	7	-0.4	454.2420	2	19.00	3	4295	211015-MClass-FE2-3uL-LSM.raw	9.9621E5	1	1	81	87	Methylation(others)

total 29 peptides

Best Unique PSM (Scan 3502, m/z=460.2691, z=3, RT=17.48, ppm=-0.8):

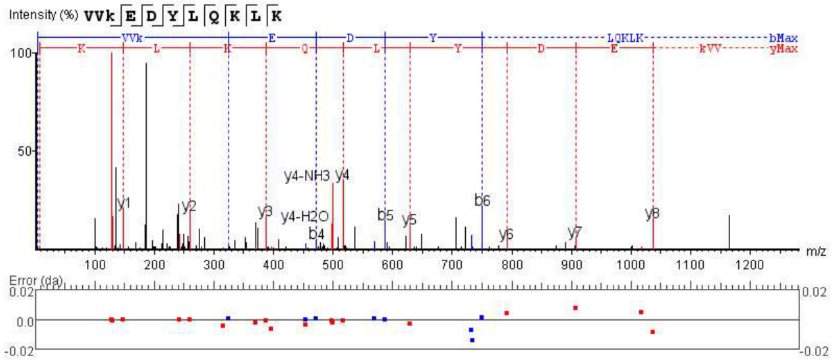

tr|A0A2L2XZE1|A0A2L2XZE1_PARTP

back to list

| Protein Coverage | Supporting Peptides | Best Unique PSM |

Protein Coverage:

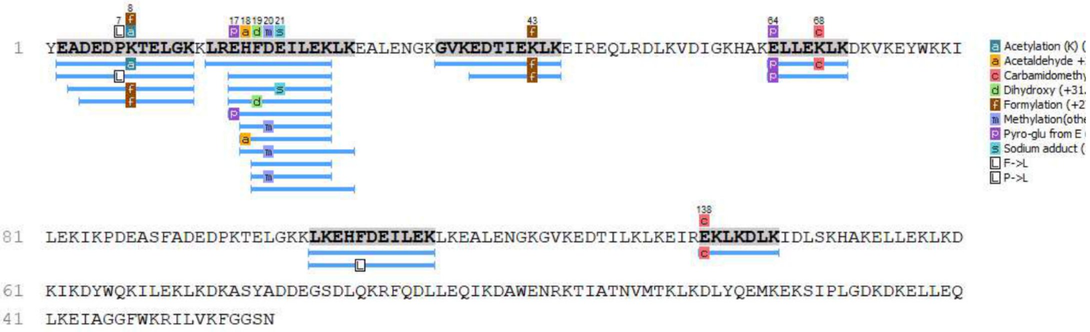

Supporting Peptides:

Peptide	Uniq	-10lgP	Mass	Length	ppm	m/z	z	RT	Fraction	Scan	Source File	Area FE2 3uL	#Feature	#Feature FE2 3uL	Start	End	PTM
K.LKEHFDEILEK.L	Y	77.70	1399.7346	11	-1.6	700.8735	2	19.30	3	4454	211015-MClass-FE2-3uL-LSM.raw	1.5181E7	4	4	104	114	
R.EHFDEILEK.L	N	76.48	1158.5557	9	-1.6	580.2842	2	19.50	3	4605	211015-MClass-FE2-3uL-LSM.raw	4.0773E7	2	2	17	25	
E.HFD(+14.02)EILEK.L	N	75.81	1043.5287	8	2.4	522.7729	2	17.88	3	3717	211015-MClass-FE2-3uL-LSM.raw	4.8719E8	3	3	18	25	Methylation(others)

total 22 peptides

Peptide	Uniq	-10lgP	Mass	Length	ppm	m/z	z	RT	Fraction	Scan	Source File	Area FE2 3uL	#Feature	#Feature FE2 3uL	Start	End	PTM
K.LREHFDEILEK.L	N	66.00	1427.7408	11	-2.4	476.9198	3	18.95	3	4273	211015-MClass-FE2-3uL-LSM.raw	6.9047E6	2	2	15	25	
R.EHFDE(+21.98)ILEK.L	N	61.71	1180.5376	9	-2.2	591.2748	2	19.04	3	4329	211015-MClass-FE2-3uL-LSM.raw	7.6886E4	1	1	17	25	Sodium adduct
R.E(-18.01)HFDEILEK.L	N	60.48	1140.5450	9	0.1	571.2798	2	22.14	3	5629	211015-MClass-FE2-3uL-LSM.raw	3.104E5	2	2	17	25	Pyro-glu from E
A.DEDPK(+27.99)TELGK.K	N	57.16	1158.5404	10	0.5	580.2778	2	16.58	3	3024	211015-MClass-FE2-3uL-LSM.raw	5.1117E4	1	1	4	13	Formylation
E.HFD(+14.02)EILEKLK.E	N	53.46	1284.7078	10	0.5	429.2434	3	21.27	3	5329	211015-MClass-FE2-3uL-LSM.raw	8.0079E4	1	1	18	27	Methylation(others)
H.FDEILEK.L	N	49.23	892.4542	7	0.2	447.2344	2	18.91	3	4244	211015-MClass-FE2-3uL-LSM.raw	9.1588E7	1	1	19	25	
R.EHF(+31.99)DEILEK.L	N	49.18	1190.5455	9	0.6	596.2804	2	21.24	3	5317	211015-MClass-FE2-3uL-LSM.raw	1.0354E5	2	2	17	25	Dihydroxy
H.FDEILEKLK.E	N	48.40	1133.6332	9	-2.0	567.8228	2	21.45	3	5393	211015-MClass-FE2-3uL-LSM.raw	4.703E5	1	1	19	27	
R.E(+57.02)KLKDLK.I	N	47.62	929.5546	7	1.0	465.7850	2	17.79	3	3690	211015-MClass-FE2-3uL-LSM.raw	8.776E4	1	1	138	144	Carbamidomethylation (DHKE, X@N-term)
K.EDTIEK(+27.99)LK.E	N	46.67	1002.5233	8	-0.2	502.2688	2	17.11	3	3313	211015-MClass-FE2-3uL-LSM.raw	0	0	0	38	45	Formylation
K.E(-18.01)LLEKLK.D	N	46.42	853.5273	7	0.5	427.7711	2	19.97	3	4780	211015-MClass-FE2-3uL-LSM.raw	2.9539E6	1	1	64	70	Pyro-glu from E
Y.EADEDPK(+42.01)TELGK.K	N	46.05	1372.6357	12	-0.8	687.3246	2	16.90	3	3203	211015-MClass-FE2-3uL-LSM.raw	1.2231E5	1	1	2	13	Acetylation (K)
E.ADEDPK(+27.99)TELGK.K	N	45.68	1229.5775	11	0.7	615.7964	2	16.52	3	2983	211015-MClass-FE2-3uL-LSM.raw	1.2941E5	1	1	3	13	Formylation
E.H(+26.02)FDEILEK.L	N	45.26	1055.5287	8	-0.7	528.7712	2	19.47	3	4564	211015-MClass-FE2-3uL-LSM.raw	1.5334E6	1	1	18	25	Acetaldehyde +26
K.E(-18.01)LLEK(+57.02)LK.D	N	43.96	910.5487	7	-0.4	456.2815	2	19.92	3	4767	211015-MClass-FE2-3uL-LSM.raw	2.0305E5	1	1	64	70	Pyro-glu from E; Carbamidomethylation (DHKE, X@N-term)
K.GVKEDTIEK(+27.99)LK.E	Y	42.39	1286.7081	11	-0.5	429.9098	3	17.02	3	3248	211015-MClass-FE2-3uL-LSM.raw	5.4998E4	1	1	35	45	Formylation
Y.EADEDL(sub P)KTELGK.K	N	41.38	1346.6565	12	-0.3	449.8926	3	16.23	3	2813	211015-MClass-FE2-3uL-LSM.raw	4.5787E6	1	1	2	13	
K.LKEHL(sub F)DEILEK.L	Y	41.25	1365.7504	11	-0.1	456.2574	3	18.57	3	4074	211015-MClass-FE2-3uL-LSM.raw	3.8646E6	1	1	104	114	
H.FD(+14.02)EILEK.L	N	35.07	906.4698	7	-0.4	454.2420	2	19.00	3	4295	211015-MClass-FE2-3uL-LSM.raw	9.9621E5	1	1	19	25	Methylation(others)
**total 22 peptides**																	

Best Unique PSM (Scan 4454, m/z=700.8735, z=2, RT=19.30, ppm=-1.6):

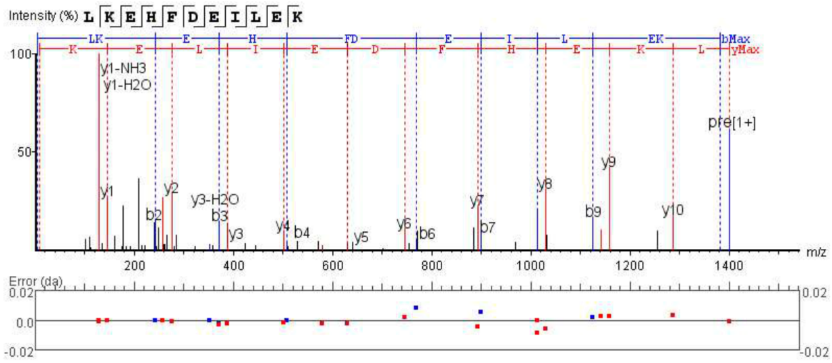

tr|A0A2L2YMR4|A0A2L2YMR4_PARTP back to list

| Protein Coverage | Supporting Peptides | Best Unique PSM |

Protein Coverage:

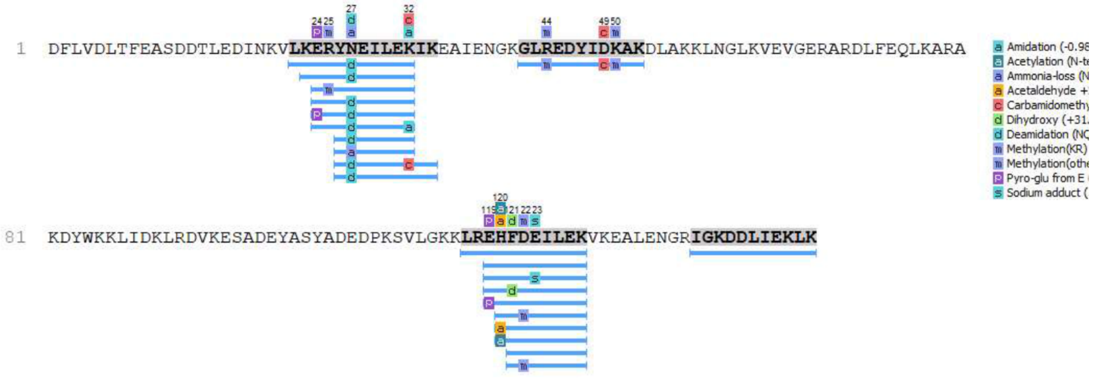

Supporting Peptides:

Peptide	Uniq	-10lgP	Mass	Length	ppm	m/z	z	RT	Fraction	Scan	Source File	Area FE2 3uL	#Feature	#Feature FE2 3uL	Start	End	PTM
R.YN(+.98)EILEKIK.E	Y	83.07	1149.6281	9	-0.7	575.8209	2	19.71	3	4665	211015-MClass-FE2-3uL-LSM.raw	1.3167E6	2	2	26	34	Deamid
R.EHFDEILEK.V	N	76.48	1158.5557	9	-1.6	580.2842	2	19.50	3	4605	211015-MClass-FE2-3uL-LSM.raw	4.0773E7	2	2	119	127	
K.ERYN(+.98)EILEK.I	Y	76.44	1193.5928	9	0.7	597.8041	2	17.99	3	3809	211015-MClass-FE2-3uL-LSM.raw	4.2705E7	2	2	24	32	Deamid
E.HFD(+14.02)EILEK.V	N	75.81	1043.5287	8	2.4	522.7729	2	17.88	3	3717	211015-MClass-FE2-3uL-LSM.raw	4.8719E8	3	3	120	127	Methyla
R.YN(+.98)EILEK.I	Y	71.48	908.4491	7	0.4	455.2320	2	17.26	3	3375	211015-MClass-FE2-3uL-LSM.raw	2.7715E6	2	2	26	32	Deamid
K.ER(+14.02)YNEILEK.I	Y	69.95	1206.6244	9	-0.6	403.2151	3	17.66	3	3623	211015-MClass-FE2-3uL-LSM.raw	4.2905E6	3	3	24	32	Methyla
K.LREHFDEILEK.V	N	66.00	1427.7408	11	-2.4	476.9198	3	18.95	3	4273	211015-MClass-FE2-3uL-LSM.raw	6.9047E6	2	2	117	127	
K.E(-18.01)RYN(+.98)EILEK.I	Y	62.25	1175.5822	9	0.4	588.7986	2	19.94	3	4782	211015-MClass-FE2-3uL-LSM.raw	5.3709E5	1	1	24	32	Pyro-glu Deamid

total 22 peptides

Peptide	Uniq	-10lgP	Mass	Length	ppm	m/z	z	RT	Fraction	Scan	Source File	Area FE2 3uL	#Feature	#Feature FE2 3uL	Start	End	PTM
K.GLR(+14.02)EDYID(+57.02)K(+14.02)AK.D	Y	61.77	1391.7408	11	0.7	464.9212	3	15.85	3	2662	211015-MClass-FE2-3uL-LSM.raw	2.6997E5	1	1	42	52	Methyla Carbam (DHKE,
R.EHFDE(+21.98)ILEK.V	N	61.71	1180.5376	9	-2.2	591.2748	2	19.04	3	4329	211015-MClass-FE2-3uL-LSM.raw	7.6886E4	1	1	119	127	Sodium
R.YN(+.98)EILEK(+57.02)IK.E	Y	60.90	1206.6495	9	-0.2	403.2237	3	19.66	3	4634	211015-MClass-FE2-3uL-LSM.raw	4.0258E5	1	1	26	34	Deamid Carbam (DHKE,
R.E(-18.01)HFDEILEK.V	N	60.48	1140.5450	9	0.1	571.2798	2	22.14	3	5629	211015-MClass-FE2-3uL-LSM.raw	3.104E5	2	2	119	127	Pyro-glu
K.ERYN(+.98)EILEK(-.98).I	Y	59.36	1192.6088	9	-1.2	597.3109	2	18.21	3	3921	211015-MClass-FE2-3uL-LSM.raw	0	0	0	24	32	Deamid Amidati
R.YN(-17.03)EILEK.I	Y	54.70	890.4385	7	0.5	446.2267	2	18.83	3	4233	211015-MClass-FE2-3uL-LSM.raw	1.3449E5	1	1	26	32	Ammon
V.LKERYN(+.98)EILEK.I	Y	53.87	1434.7717	11	-1.5	718.3920	2	17.63	3	3592	211015-MClass-FE2-3uL-LSM.raw	9.4607E6	2	2	22	32	Deamid
H.FDEILEK.V	N	49.23	892.4542	7	0.2	447.2344	2	18.91	3	4244	211015-MClass-FE2-3uL-LSM.raw	9.1588E7	1	1	121	127	
R.EHF(+31.99)DEILEK.V	N	49.18	1190.5455	9	0.6	596.2804	2	21.24	3	5317	211015-MClass-FE2-3uL-LSM.raw	1.0354E5	2	2	119	127	Dihydro
E.H(+26.02)FDEILEK.V	N	45.26	1055.5287	8	-0.7	528.7712	2	19.47	3	4564	211015-MClass-FE2-3uL-LSM.raw	1.5334E6	1	1	120	127	Acetald
R.IGKDDLIEKLK	Y	43.39	1270.7496	11	0.3	424.5906	3	19.98	3	4797	211015-MClass-FE2-3uL-LSM.raw	2.7715E5	1	1	137	147	
L.KERYN(+.98)EILEK.I	Y	41.57	1321.6877	10	-3.9	661.8486	2	17.89	3	3749	211015-MClass-FE2-3uL-LSM.raw	1.3003E5	1	1	23	32	Deamid
E.H(+42.01)FDEILEK.V	N	41.55	1071.5237	8	2.4	536.7704	2	21.76	3	5524	211015-MClass-FE2-3uL-LSM.raw	6.3841E5	2	2	120	127	Acetylat
H.FD(+14.02)EILEK.V	N	35.07	906.4698	7	-0.4	454.2420	2	19.00	3	4295	211015-MClass-FE2-3uL-LSM.raw	9.9621E5	1	1	121	127	Methyla
**total 22 peptides**																	

Best Unique PSM (Scan 4665, m/z=575.8209, z=2, RT=19.71, ppm=-0.7):

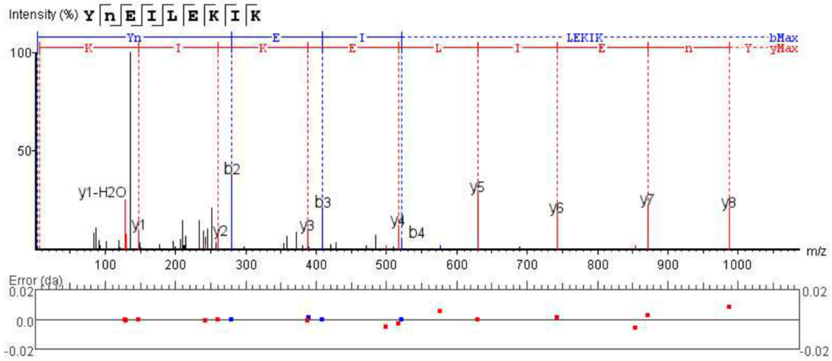

tr|A0A2L2XZF8|A0A2L2XZF8_PARTP back to list

| Protein Coverage | Supporting Peptides | Best Unique PSM |

Protein Coverage:

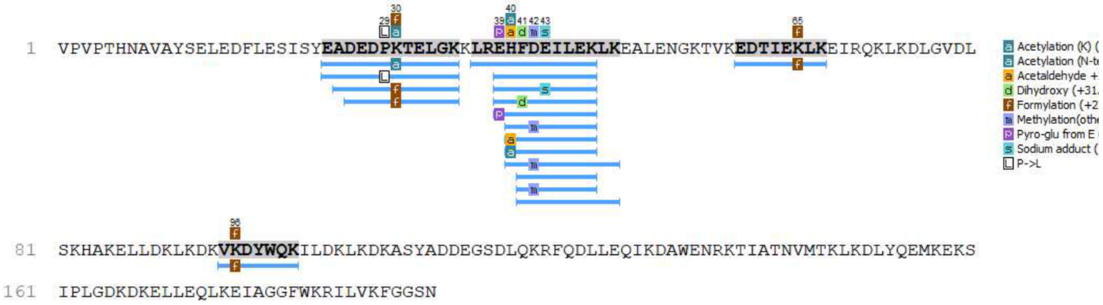

Supporting Peptides:

Peptide	Uniq	-10lgP	Mass	Length	ppm	m/z	z	RT	Fraction	Scan	Source File	Area FE2 3uL	#Feature	#Feature FE2 3uL	Start	End	PTM	ASc
R.EHFDEILEK.L	N	76.48	1158.5557	9	-1.6	580.2842	2	19.50	3	4605	211015-MClass-FE2-3uL-LSM.raw	4.0773E7	2	2	39	47		
E.HFD(+14.02)EILEK.L	N	75.81	1043.5287	8	2.4	522.7729	2	17.88	3	3717	211015-MClass-FE2-3uL-LSM.raw	4.8719E8	3	3	40	47	Methylation(others)	D3:I hyla (oth s):3 6
K.LREHFDEILEK.L	N	66.00	1427.7408	11	-2.4	476.9198	3	18.95	3	4273	211015-MClass-FE2-3uL-LSM.raw	6.9047E6	2	2	37	47		
R.EHFDE(+21.98)ILEK.L	N	61.71	1180.5376	9	-2.2	591.2748	2	19.04	3	4329	211015-MClass-FE2-3uL-LSM.raw	7.6886E4	1	1	39	47	Sodium adduct	E5:S um a uct: 06
R.E(-18.01)HFDEILEK.L	N	60.48	1140.5450	9	0.1	571.2798	2	22.14	3	5629	211015-MClass-FE2-3uL-LSM.raw	3.104E5	2	2	39	47	Pyro-glu from E	E1:F -glu m E 00.0
A.DEDPK(+27.99)TELGK.K	N	57.16	1158.5404	10	0.5	580.2778	2	16.58	3	3024	211015-MClass-FE2-3uL-LSM.raw	5.1117E4	1	1	26	35	Formylation	K5:F myla n:13
K.VK(+27.99)DYWQK.I	Y	55.17	993.4919	7	0.2	497.7534	2	17.68	3	3640	211015-MClass-FE2-3uL-LSM.raw	7.9455E4	1	1	95	101	Formylation	K2:F myla n:17
E.HFD(+14.02)EILEKLK.E	N	53.46	1284.7078	10	0.5	429.2434	3	21.27	3	5329	211015-MClass-FE2-3uL-LSM.raw	8.0079E4	1	1	40	49	Methylation(others)	D3:I hyla (oth s):1 6
H.FDEILEK.L	N	49.23	892.4542	7	0.2	447.2344	2	18.91	3	4244	211015-MClass-FE2-3uL-LSM.raw	9.1588E7	1	1	41	47		
R.EHF(+31.99)DEILEK.L	N	49.18	1190.5455	9	0.6	596.2804	2	21.24	3	5317	211015-MClass-FE2-3uL-LSM.raw	1.0354E5	2	2	39	47	Dihydroxy	F3:D drox 1.94

total 18 peptides

Peptide	Uniq	-10lgP	Mass	Length	ppm	m/z	z	RT	Fraction	Scan	Source File	Area FE2 3uL	#Feature	#Feature FE2 3uL	Start	End	PTM	ASc
H.FDEILEKLK.E	N	48.40	1133.6332	9	-2.0	567.8228	2	21.45	3	5393	211015-MClass-FE2-3uL-LSM.raw	4.703E5	1	1	41	49		
K.EDTIEK(+27.99)LK.E	N	46.67	1002.5233	8	-0.2	502.2688	2	17.11	3	3313	211015-MClass-FE2-3uL-LSM.raw	0	0	0	60	67	Formylation	K6:F myla n:18
Y.EADEDPK(+42.01)TELGK.K	N	46.05	1372.6357	12	-0.8	687.3246	2	16.90	3	3203	211015-MClass-FE2-3uL-LSM.raw	1.2231E5	1	1	24	35	Acetylation (K)	K7:A ylati (K): 58
E.ADEDPK(+27.99)TELGK.K	N	45.68	1229.5775	11	0.7	615.7964	2	16.52	3	2983	211015-MClass-FE2-3uL-LSM.raw	1.2941E5	1	1	25	35	Formylation	K6:F myla n:15
E.H(+26.02)FDEILEK.L	N	45.26	1055.5287	8	-0.7	528.7712	2	19.47	3	4564	211015-MClass-FE2-3uL-LSM.raw	1.5334E6	1	1	40	47	Acetaldehyde +26	H1:A talde de + 6:12 6
E.H(+42.01)FDEILEK.L	N	41.55	1071.5237	8	2.4	536.7704	2	21.76	3	5524	211015-MClass-FE2-3uL-LSM.raw	6.3841E5	2	2	40	47	Acetylation (N-term)	H1:A tylat (N-t m): 0.00
Y.EADEDL(sub P)KTELGK.K	N	41.38	1346.6565	12	-0.3	449.8926	3	16.23	3	2813	211015-MClass-FE2-3uL-LSM.raw	4.5787E6	1	1	24	35		
H.FD(+14.02)EILEK.L	N	35.07	906.4698	7	-0.4	454.2420	2	19.00	3	4295	211015-MClass-FE2-3uL-LSM.raw	9.9621E5	1	1	41	47	Methylation(others)	D2:I hyla (oth s):3 6
**total 18 peptides**																		

Best Unique PSM (Scan 3640, m/z=497.7534, z=2, RT=17.68, ppm=0.2):

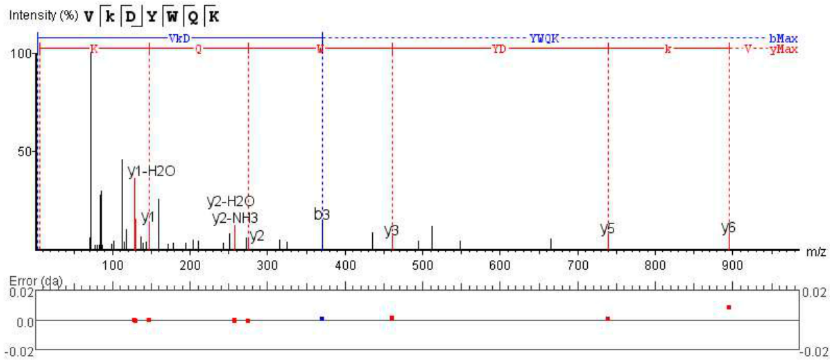

tr|E7D1R3|E7D1R3_LATHE

back to list

| Protein Coverage | Supporting Peptides | Best Unique PSM |

Protein Coverage:

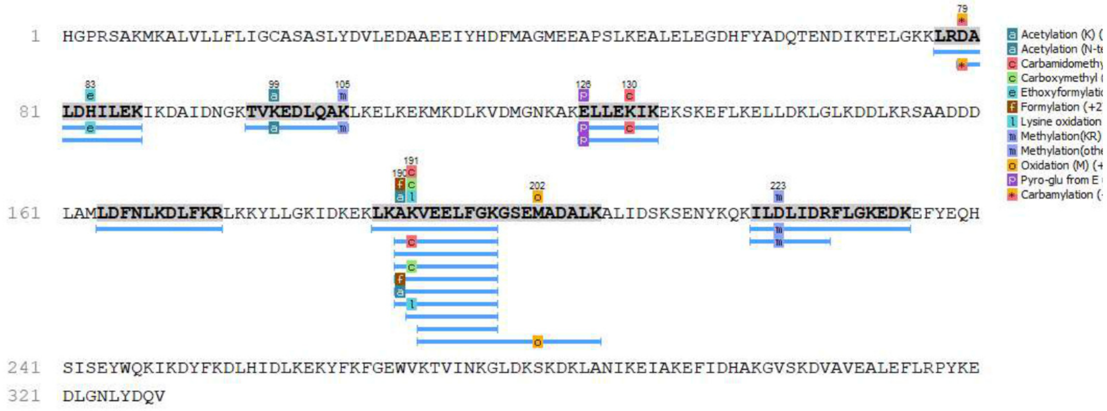

Supporting Peptides:

Peptide	Uniq	-10lgP	Mass	Length	ppm	m/z	z	RT	Fraction	Scan	Source File	Area FE2 3uL	#Feature	#Feature FE2 3uL	Start	End	PTM
K.AK(+57.02)VEELFGK.G	Y	74.42	1076.5865	9	0.4	539.3008	2	18.50	3	4062	211015-MClass-FE2-3uL-LSM.raw	2.0734E5	1	1	190	198	Carbamidomethylat (DHKE, X@N-term)
K.A(+27.99)KVEELFGK.G	Y	67.16	1047.5601	9	-0.3	524.7872	2	21.52	3	5420	211015-MClass-FE2-3uL-LSM.raw	7.6237E5	1	1	190	198	Formylation
K.VEELFGKGSEM(+15.99)ADALK.A	Y	64.26	1738.8447	16	-0.6	580.6218	3	19.79	3	4697	211015-MClass-FE2-3uL-LSM.raw	1.3078E6	2	2	192	207	Oxidation (M)
K.A(+42.01)KVEELFGK.G	Y	62.88	1061.5757	9	3.1	531.7968	2	21.76	3	5515	211015-MClass-FE2-3uL-LSM.raw	5.6151E4	1	1	190	198	Acetylation (N-term
K.AKVEELFGK.G	Y	57.98	1019.5651	9	-0.8	510.7894	2	18.53	3	4041	211015-MClass-FE2-3uL-LSM.raw	5.6766E7	1	1	190	198	
K.AK(-1.03)VEELFGK.G	Y	57.87	1018.5334	9	0.0	510.2740	2	23.32	3	5973	211015-MClass-FE2-3uL-LSM.raw	3.8538E5	1	1	190	198	Lysine oxidation to aminoadipic semialdehyde
K.AK(+58.01)VEELFGK.G	Y	54.68	1077.5706	9	1.0	539.7931	2	19.96	3	4796	211015-MClass-FE2-3uL-LSM.raw	1.6014E5	1	1	190	198	Carboxymethyl (KW X@N-term)
K.ILD(+14.02)LIDR.F	Y	50.69	870.5175	7	0.7	436.2663	2	22.07	3	5620	211015-MClass-FE2-3uL-LSM.raw	0	0	0	221	227	Methylation(others)
K.VEELFGK.G	Y	46.86	820.4330	7	-1.7	411.2231	2	18.92	3	4257	211015-MClass-FE2-3uL-LSM.raw	8.8546E6	1	1	192	198	
K.E(-18.01)LLEKIK.E	N	45.85	853.5273	7	0.5	427.7711	2	19.97	3	4780	211015-MClass-FE2-3uL-LSM.raw	2.9539E6	1	1	126	132	Pyro-glu from E
K.TVK(+42.01)EDLQAK(+14.02).L	Y	43.45	1086.5920	9	-0.1	544.3032	2	19.21	3	4413	211015-MClass-FE2-3uL-LSM.raw	4.4172E5	1	1	97	105	Acetylation (K); Methylation(KR)
K.E(-18.01)LLEK(+57.02)IK.E	N	43.41	910.5487	7	-0.4	456.2815	2	19.92	3	4767	211015-MClass-FE2-3uL-LSM.raw	2.0305E5	1	1	126	132	Pyro-glu from E; Carbamidomethylat (DHKE, X@N-term)
K.ILD(+14.02)LIDRFLGKEDK.E	Y	43.29	1687.9508	14	-0.1	422.9949	4	26.75	3	6758	211015-MClass-FE2-3uL-LSM.raw	2.5825E5	1	1	221	234	Methylation(others)
K.LKAKVEELFGK.G	Y	42.64	1260.7441	11	-3.2	421.2540	3	18.90	3	4274	211015-MClass-FE2-3uL-LSM.raw	3.6144E5	1	1	188	198	
K.LRDALDH(+72.02)ILEK.I	Y	40.22	1393.7565	11	-2.5	697.8838	2	18.64	3	4121	211015-MClass-FE2-3uL-LSM.raw	1.9126E5	1	1	77	87	Ethoxyformylation
A.KVEELFGK.G	Y	38.51	948.5280	8	1.8	475.2721	2	18.52	3	4063	211015-MClass-FE2-3uL-LSM.raw	3.2137E5	1	1	191	198	

total 18 peptides

Peptide	Uniq	-10lgP	Mass	Length	ppm	m/z	z	RT	Fraction	Scan	Source File	Area FE2 3uL	#Feature	#Feature FE2 3uL	Start	End	PTM
R.D(+43.01)(+57.02)ALDHILEK.I	Y	38.35	1152.5775	9	3.8	577.2982	2	20.62	3	5104	211015-MClass-FE2-3uL-LSM.raw	5.7152E5	1	1	79	87	Carbamylation; Carbamidomethylat (DHKE, X@N-term)
M.LDFNLKDLFKR.L	Y	35.86	1407.7874	11	0.2	470.2698	3	26.26	3	6660	211015-MClass-FE2-3uL-LSM.raw	3.0752E5	1	1	164	174	
**total 18 peptides**																	

Best Unique PSM (Scan 4062, m/z=539.3008, z=2, RT=18.50, ppm=0.4):

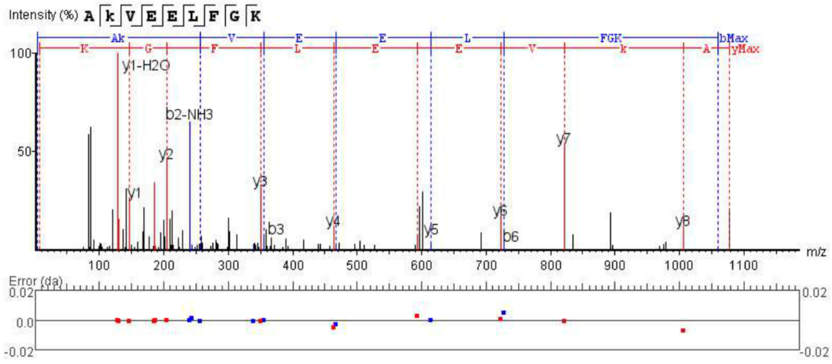

tr|A0A2L2Z8N3|A0A2L2Z8N3_PARTP back to list

| Protein Coverage | Supporting Peptides | Best Unique PSM |

Protein Coverage:

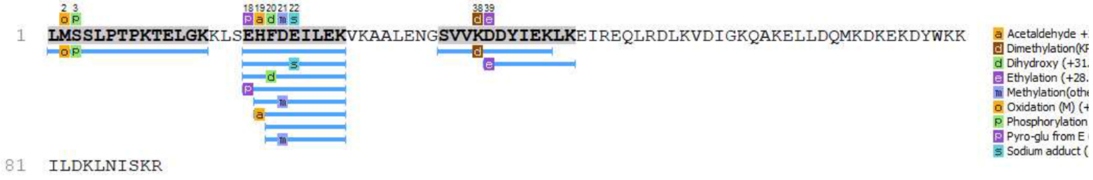

Supporting Peptides:

Peptide	Uniq	-10lgP	Mass	Length	ppm	m/z	z	RT	Fraction	Scan	Source File	Area FE2 3uL	#Feature	#Feature FE2 3uL	Start	End	PTM
S.EHFDEILEK.V	N	76.48	1158.5557	9	-1.6	580.2842	2	19.50	3	4605	211015-MClass-FE2-3uL-LSM.raw	4.0773E7	2	2	18	26	
E.HFD(+14.02)EILEK.V	N	75.81	1043.5287	8	2.4	522.7729	2	17.88	3	3717	211015-MClass-FE2-3uL-LSM.raw	4.8719E8	3	3	19	26	Methylation(oth
S.EHFDE(+21.98)ILEK.V	N	61.71	1180.5376	9	-2.2	591.2748	2	19.04	3	4329	211015-MClass-FE2-3uL-LSM.raw	7.6886E4	1	1	18	26	Sodium adduct
S.E(-18.01)HFDEILEK.V	N	60.48	1140.5450	9	0.1	571.2798	2	22.14	3	5629	211015-MClass-FE2-3uL-LSM.raw	3.104E5	2	2	18	26	Pyro-glu from E
K.D(+28.03)DYIEKLK.E	Y	59.76	1050.5597	8	-1.2	526.2865	2	17.41	3	3471	211015-MClass-FE2-3uL-LSM.raw	1.1456E5	1	1	39	46	Ethylation
H.FDEILEK.V	N	49.23	892.4542	7	0.2	447.2344	2	18.91	3	4244	211015-MClass-FE2-3uL-LSM.raw	9.1588E7	1	1	20	26	
S.EHF(+31.99)DEILEK.V	N	49.18	1190.5455	9	0.6	596.2804	2	21.24	3	5317	211015-MClass-FE2-3uL-LSM.raw	1.0354E5	2	2	18	26	Dihydroxy

**total 11 peptides**

Peptide	Uniq	-10lgP	Mass	Length	ppm	m/z	z	RT	Fraction	Scan	Source File	Area FE2 3uL	#Feature	#Feature FE2 3uL	Start	End	PTM
E.H(+26.02)FDEILEK.V	N	45.26	1055.5287	8	-0.7	528.7712	2	19.47	3	4564	211015-MClass-FE2-3uL-LSM.raw	1.5334E6	1	1	19	26	Acetaldehyde +
LM(+15.99)S(+79.97)SLPTPKTELGK.K	Y	40.36	1596.7833	14	-2.7	533.2670	3	16.25	3	2860	211015-MClass-FE2-3uL-LSM.raw	1.9213E4	1	1	1	14	Oxidation (M); Phosphorylation (STY)
G.SVVK(+28.03)DDYIEK.L	Y	35.91	1222.6445	10	0.6	408.5557	3	17.28	3	3399	211015-MClass-FE2-3uL-LSM.raw	2.9455E5	1	1	35	44	Dimethylation(k
H.FD(+14.02)EILEK.V	N	35.07	906.4698	7	-0.4	454.2420	2	19.00	3	4295	211015-MClass-FE2-3uL-LSM.raw	9.9621E5	1	1	20	26	Methylation(oth
**total 11 peptides**																	

Best Unique PSM (Scan 3471, m/z=526.2865, z=2, RT=17.41, ppm=-1.2):

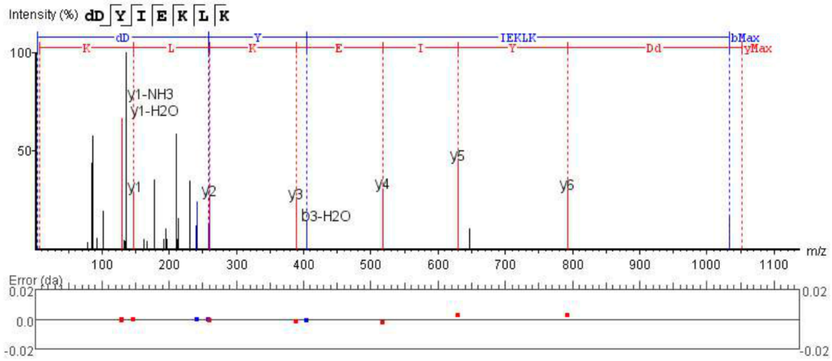

tr|E7D195|E7D195_LATHE

back to list

| Protein Coverage | Supporting Peptides | Best Unique PSM |

Protein Coverage:

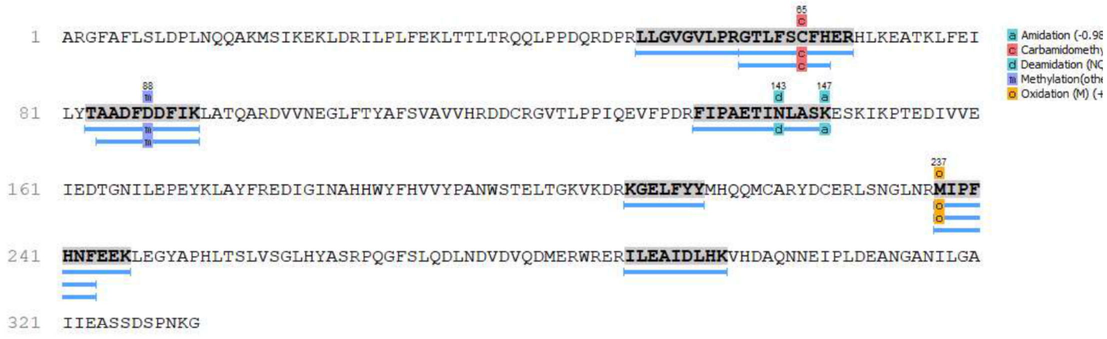

Supporting Peptides:

Peptide	Uniq	-10lgP	Mass	Length	ppm	m/z	z	RT	Fraction	Scan	Source File	Area FE2 3uL	#Feature	#Feature FE2 3uL	Start	End	PTM
R.GTLFSC(+57.02)FHER.H	Y	60.53	1252.5659	10	0.3	418.5294	3	20.71	3	5100	211015-MClass-FE2-3uL-LSM.raw	1.6503E5	1	1	60	69	Carbamidomethylation
R.ILEAIDLHK.V	Y	57.31	1050.6073	9	-2.2	526.3098	2	19.54	3	4588	211015-MClass-FE2-3uL-LSM.raw	8.876E5	1	1	290	298	
R.GTLFSC(+57.02)FH.E	Y	53.03	967.4222	8	0.0	484.7184	2	22.90	3	5873	211015-MClass-FE2-3uL-LSM.raw	9.2897E4	1	1	60	67	Carbamidomethylation
**total 11 peptides**																	

Peptide	Uniq	-10lgP	Mass	Length	ppm	m/z	z	RT	Fraction	Scan	Source File	Area FE2 3uL	#Feature	#Feature FE2 3uL	Start	End	PTM
Y.TAADFD(+14.02)DFIK.L	Y	52.95	1155.5448	10	-0.5	578.7794	2	23.91	3	6138	211015-MClass-FE2-3uL-LSM.raw	6.1365E5	1	1	83	92	Methylation(others)
R.FIPAETIN(+.98)LASK(-.98).E	Y	52.81	1302.7183	12	-0.5	652.3661	2	22.32	3	5681	211015-MClass-FE2-3uL-LSM.raw	1.2274E5	1	1	136	147	Deamidation (NQ); Amidation
T.AADFD(+14.02)DFIK.L	Y	52.60	1054.4971	9	0.4	528.2560	2	23.70	3	6083	211015-MClass-FE2-3uL-LSM.raw	1.433E6	1	1	84	92	Methylation(others)
R.M(+15.99)IPFHNFEEK.L	Y	50.27	1306.6016	10	-0.3	436.5410	3	19.26	3	4444	211015-MClass-FE2-3uL-LSM.raw	4.167E5	2	2	237	246	Oxidation (M)
R.LLGVGVLPR.G	Y	42.88	922.5964	9	1.5	462.3062	2	22.78	3	5817	211015-MClass-FE2-3uL-LSM.raw	3.1067E6	1	1	51	59	
R.MIPFHNF.E	N	42.05	904.4265	7	1.0	453.2210	2	23.56	3	6060	211015-MClass-FE2-3uL-LSM.raw	1.3967E5	1	1	237	243	
R.M(+15.99)IPFHNF.E	N	40.14	920.4214	7	-0.5	461.2178	2	21.88	3	5542	211015-MClass-FE2-3uL-LSM.raw	3.7742E6	1	1	237	243	Oxidation (M)
R.KGELFYY.M	N	40.10	918.4487	7	0.4	460.2318	2	22.70	3	5800	211015-MClass-FE2-3uL-LSM.raw	5.3772E5	1	1	210	216	

total 11 peptides

Best Unique PSM (Scan 5100, m/z=418.5294, z=3, RT=20.71, ppm=0.3):

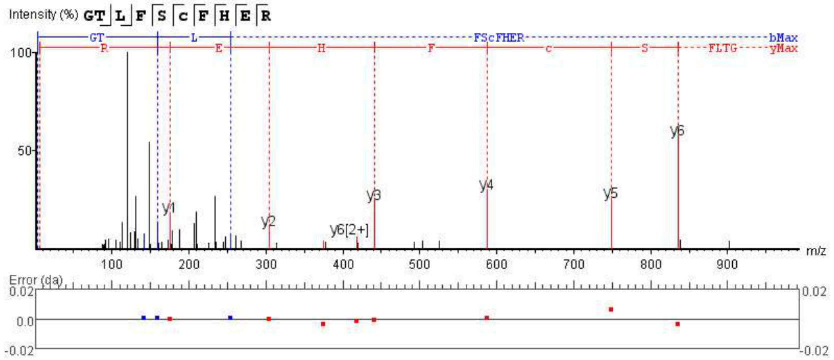

tr|A0A2L2YG81|A0A2L2YG81_PARTP

back to list

| Protein Coverage | Supporting Peptides | Best Unique PSM |

Protein Coverage:

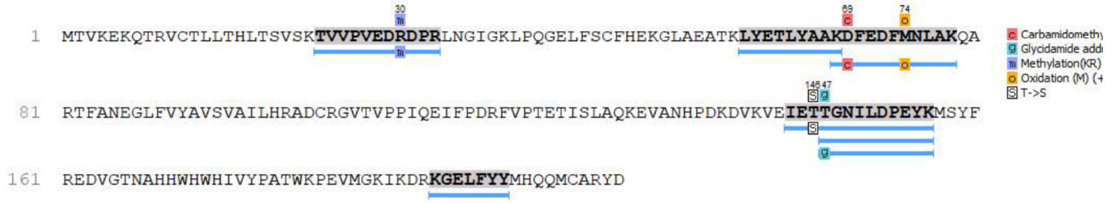

Supporting Peptides:

Peptide	Uniq	-10lgP	Mass	Length	ppm	m/z	z	RT	Fraction	Scan	Source File	Area FE2 3uL	#Feature	#Feature FE2 3uL	Start	End	PTM
T.T(+87.03)GNILDPEYK.M	Y	77.30	1235.6034	10	-1.6	618.8080	2	19.63	3	4628	211015-MClass-FE2-3uL-LSM.raw	1.4534E6	1	1	147	156	Glycidamide add

total 7 peptides

Peptide	Uniq	-10lgP	Mass	Length	ppm	m/z	z	RT	Fraction	Scan	Source File	Area FE2 3uL	#Feature	#Feature FE2 3uL	Start	End	PTM
K.LYETLYAAK.D	N	61.97	1070.5648	9	-0.4	536.2895	2	19.46	3	4565	211015-MClass-FE2-3uL-LSM.raw	7.0952E5	1	1	60	68	
T.TGNILDPEYK.M	Y	54.04	1148.5713	10	-0.1	575.2928	2	19.52	3	4587	211015-MClass-FE2-3uL-LSM.raw	1.8532E5	1	1	147	156	
A.KD(+57.02)FEDFM(+15.99)NLAK.Q	Y	47.55	1429.6548	11	-2.2	477.5578	3	20.15	3	4891	211015-MClass-FE2-3uL-LSM.raw	2.7364E5	1	1	68	78	Carbamidomethy (DHKE, X@N-ter Oxidation (M)
K.TVVPVEDR(+14.02)DPR.L	Y	45.90	1295.6833	11	1.0	432.9022	3	16.69	3	3105	211015-MClass-FE2-3uL-LSM.raw	1.9444E4	1	1	23	33	Methylation(KR)
E.IES(sub T)TGNILDPEYK.M	Y	41.28	1477.7300	13	-0.6	739.8718	2	20.92	3	5180	211015-MClass-FE2-3uL-LSM.raw	1.0792E6	1	1	144	156	
R.KGELFYY.M	N	40.10	918.4487	7	0.4	460.2318	2	22.70	3	5800	211015-MClass-FE2-3uL-LSM.raw	5.3772E5	1	1	193	199	
**total 7 peptides**																	

Best Unique PSM (Scan 4628, m/z=618.8080, z=2, RT=19.63, ppm=-1.6):

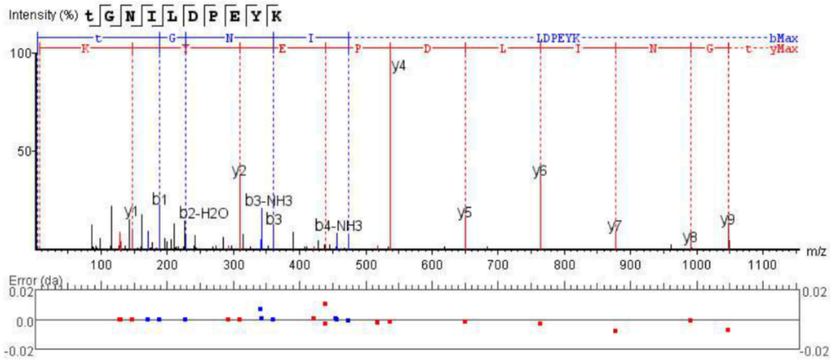

tr|A0A2L2Y2X9|A0A2L2Y2X9_PARTP

back to list

| Protein Coverage | Supporting Peptides | Best Unique PSM |

Protein Coverage:

```
1   MTVKEKQTRVCSLFTKLTSVSKTIVPVDQRDPRLHGIGKLAQGELFSCFHEKGLAEATKLYETLYAAKDFEDFMILAKQA
81  RTFANEGLFVYAASVAILHRADCRGVTVPPIQEIFPDRFVPTETISLAQKEVANHPDKDIKVEIESTGNILDPEYRMSYF
161 REDVGTNAHHWHWHIVYPATWRPEVMGSIKDRKGELFYYMHQQMCARYDCERLSNGMRRMIPFHNFEENLEGYAPHLTSL
241 VSGLQYSSRPQGFSLHDLKDVDVQDMTRWRERIIEAIHIGYVENENHQQVKLTPENGIDILGALLESSYESTNKLFYGSL
321 HNWGHVMMANITDPDGRFNENPGVMSDTSTSLRDPIFYRYHRFIDNIFQEYKSTLTPYTKADLDFPGVQIVNVTVNAKVP
401 NLVTTHMKTDELELTHGIDFGTTHSVKVLYQHLDHEPFTYTINLDNGSGATKATVRIFLGPKYDELGNRLDPEHQRTLCI
481 ELDKFQVDLTAGKNTITRDHKHSSVTVSESHSFAKLLAGEGVSEATTEFCSCGWPEHMLIPRGSHKGTEFDLFVMLTDYT
561 KDAVDGESSGICQDAISYCGAKDQKYPDKKPMGFPFDRVIQAGTVHEFLTPNMSVTDVKIKHQV
```

Modification sites marked: 220 (O), 265 (e), 340 (m), 572 (C), 574 (O), 579 (C)

C Carbamidomethy
e Ethylation (+28.
m Methylation(othe
O Oxidation (M) (+
O Oxidation or Hyc

Supporting Peptides:

Peptide	Uniq	-10lgP	Mass	Length	ppm	m/z	z	RT	Fraction	Scan	Source File	Area FE2 3uL	#Feature	#Feature FE2 3uL	Start	End	PTM
**total 9 peptides**																	

Peptide	Uniq	-10lgP	Mass	Length	ppm	m/z	z	RT	Fraction	Scan	Source File	Area FE2 3uL	#Feature	#Feature FE2 3uL	Start	End	PTM
R.FNE(+14.02)NPGVM.S	Y	63.69	920.4062	8	1.0	461.2108	2	19.30	3	4473	211015-MClass-FE2-3uL-LSM.raw	3.859E5	1	1	338	345	Meth
K.LYETLYAAK.D	N	61.97	1070.5648	9	-0.4	536.2895	2	19.46	3	4565	211015-MClass-FE2-3uL-LSM.raw	7.0952E5	1	1	60	68	
K.DVDVQD(+28.03)MTR.W	Y	61.30	1105.5073	9	-0.4	553.7607	2	17.14	3	3296	211015-MClass-FE2-3uL-LSM.raw	2.6702E5	1	1	260	268	Ethyl
R.MIPFHNF.E	N	42.05	904.4265	7	1.0	453.2210	2	23.56	3	6060	211015-MClass-FE2-3uL-LSM.raw	1.3967E5	1	1	220	226	
R.M(+15.99)IPFHNF.E	N	40.14	920.4214	7	-0.5	461.2178	2	21.88	3	5542	211015-MClass-FE2-3uL-LSM.raw	3.7742E6	1	1	220	226	Oxida
R.KGELFYY.M	N	40.10	918.4487	7	0.4	460.2318	2	22.70	3	5800	211015-MClass-FE2-3uL-LSM.raw	5.3772E5	1	1	193	199	
M.SDTSTSLRDPIFYR.Y	Y	38.83	1656.8107	14	-0.5	553.2772	3	21.12	3	5265	211015-MClass-FE2-3uL-LSM.raw	1.1554E6	1	1	346	359	
S.GIC(+57.02)QD(+15.99)AISYC(+57.02)GAK.D	Y	35.77	1457.6279	13	-0.7	486.8829	3	16.61	3	3028	211015-MClass-FE2-3uL-LSM.raw	9.4226E5	1	1	570	582	Carba<br>Oxida<br>Hydr
D.TSTSLRDPIFYR.Y	Y	35.56	1454.7517	12	0.4	485.9247	3	20.68	3	5090	211015-MClass-FE2-3uL-LSM.raw	1.4506E5	1	1	348	359	
**total 9 peptides**																	

Best Unique PSM (Scan 4473, m/z=461.2108, z=2, RT=19.30, ppm=1.0):

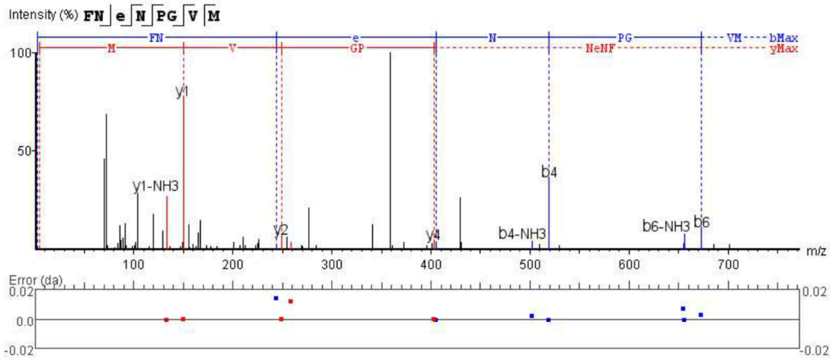

tr|A0A2L2Y308|A0A2L2Y308_PARTP

back to list

| Protein Coverage | Supporting Peptides | Best Unique PSM |

Protein Coverage:

1 MTVKEKQTRVCSLFTKLTSVSKTIVPVDQRDPRLHGIGKLAQGELFSCFHEKGLAEATK**LYETLYAAK**DFEDFMILAKQA

81 RTFANEGLFVYAASVAILHRADCRGVTVPPIQEIFPDRFVPTETISLAQKEVANHPDKDIKVEIESTGNILDPEYRMSYF

161 REDVGTNAHHWHWHIVYPATWRPEVMGSIKDR**KGELFYY**MHQQMCARYDCERLSNGMRR**MIPFHNF**EENLEGYAPHLTSL

241 VSGLQYSSRPQGFSLHDLK**DVDVQDMTR**WRERIIEAIHLRYVEDEKHQRITLTPENGIDILGSLLESSYESKNKLFYGSL

321 HNWGHVMMANITDPDGR**FNENPGVMSDTSTSLRDPIFYR**YHRFIDNIFQEYKSTLTPYTKADLEFPGVQVVSVTVNAKVP

401 NFITTHMKTDELELTHGIDLGTTHSVKVLYQHLDHEPFTYTINVDNGSGGAKQATVRIFLGPKYDELGNRLDPEHQRTLC

481 IELDKFQVELAAGKSSITRDHKLSSVTVSETHNFAKLLAGEGVSEATTEFCSCGWPEHMLIPRGSHKGTEFDLFVMLTDY

561 SKDAVDGESS**GICQDAISYCGAK**DQKYPDKKPMGFPFDRVIQAGTVHEFLTPNMSVTDVKIKHQV

C Carbamidomethy
e Ethylation (+28.
m Methylation(othe
o Oxidation (M) (+
o Oxidation or Hyc

Supporting Peptides:

Peptide	Uniq	-10lgP	Mass	Length	ppm	m/z	z	RT	Fraction	Scan	Source File	Area FE2 3uL	#Feature	#Feature FE2 3uL	Start	End	PTM
R.FNE(+14.02)NPGVM.S	Y	63.69	920.4062	8	1.0	461.2108	2	19.30	3	4473	211015-MClass-FE2-3uL-LSM.raw	3.859E5	1	1	338	345	Meth
K.LYETLYAAK.D	N	61.97	1070.5648	9	-0.4	536.2895	2	19.46	3	4565	211015-MClass-FE2-3uL-LSM.raw	7.0952E5	1	1	60	68	
K.DVDVQD(+28.03)MTR.W	Y	61.30	1105.5073	9	-0.4	553.7607	2	17.14	3	3296	211015-MClass-FE2-3uL-LSM.raw	2.6702E5	1	1	260	268	Ethyl
R.MIPFHNF.E	N	42.05	904.4265	7	1.0	453.2210	2	23.56	3	6060	211015-MClass-FE2-3uL-LSM.raw	1.3967E5	1	1	220	226	
R.M(+15.99)IPFHNF.E	N	40.14	920.4214	7	-0.5	461.2178	2	21.88	3	5542	211015-MClass-FE2-3uL-LSM.raw	3.7742E6	1	1	220	226	Oxida
R.KGELFYY.M	N	40.10	918.4487	7	0.4	460.2318	2	22.70	3	5800	211015-MClass-FE2-3uL-LSM.raw	5.3772E5	1	1	193	199	
M.SDTSTSLRDPIFYR.Y	Y	38.83	1656.8107	14	-0.5	553.2772	3	21.12	3	5265	211015-MClass-FE2-3uL-LSM.raw	1.1554E6	1	1	346	359	
S.GIC(+57.02)QD(+15.99)AISYC(+57.02)GAK.D	Y	35.77	1457.6279	13	-0.7	486.8829	3	16.61	3	3028	211015-MClass-FE2-3uL-LSM.raw	9.4226E5	1	1	571	583	Carba Oxida Hydro
D.TSTSLRDPIFYR.Y	Y	35.56	1454.7517	12	0.4	485.9247	3	20.68	3	5090	211015-MClass-FE2-3uL-LSM.raw	1.4506E5	1	1	348	359	
total 9 peptides																	

Best Unique PSM (Scan 4473, m/z=461.2108, z=2, RT=19.30, ppm=1.0):

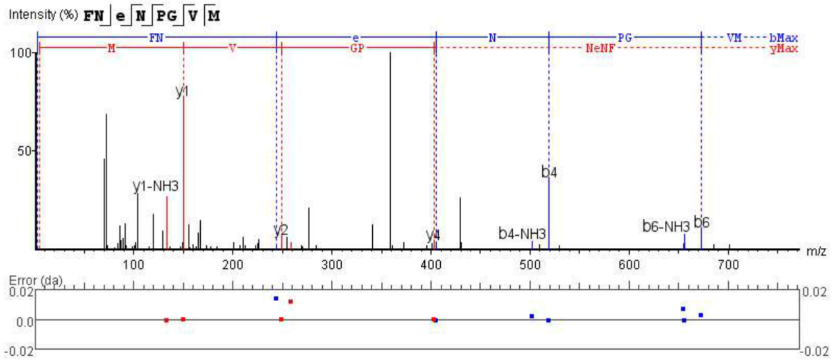

tr|A0A2L2Z761|A0A2L2Z761_PARTP

back to list

| Protein Coverage | Supporting Peptides | Best Unique PSM |

Protein Coverage:

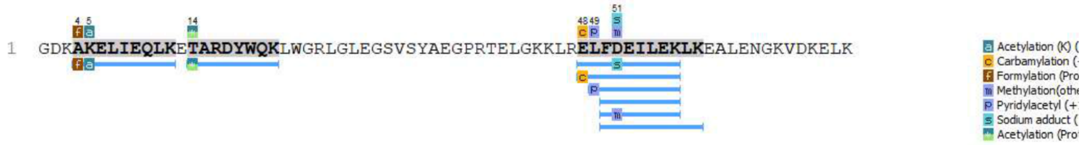

Supporting Peptides:

Peptide	Uniq	-10lgP	Mass	Length	ppm	m/z	z	RT	Fraction	Scan	Source File	Area FE2 3uL	#Feature	#Feature FE2 3uL	Start	End	PTM
K.A(+27.99)K(+42.01)ELIEQLK.E	Y	59.01	1140.6390	9	-0.5	571.3265	2	20.98	3	5226	211015-MClass-FE2-3uL-LSM.raw	3.9289E5	1	1	4	12	Formylation (Protein N-term); Acetylation (K)
R.E(+43.01)LFDEILEK.L	Y	53.57	1177.5867	9	-4.1	589.7982	2	20.24	3	4937	211015-MClass-FE2-3uL-LSM.raw	3.0859E5	1	1	48	56	Carbamylation
E.T(+42.01)(+79.97)ARDYWQK.L	Y	52.75	1188.4965	8	-3.1	595.2537	2	17.52	3	3542	211015-MClass-FE2-3uL-LSM.raw	1.2594E5	1	1	14	21	Acetylation (Protein N-term); Phosphorylation (STY)
L.FDEILEK.L	N	49.23	892.4542	7	0.2	447.2344	2	18.91	3	4244	211015-MClass-FE2-3uL-LSM.raw	9.1588E7	1	1	50	56	
L.FDEILEKLK.E	N	48.40	1133.6332	9	-2.0	567.8228	2	21.45	3	5393	211015-MClass-FE2-3uL-LSM.raw	4.703E5	1	1	50	58	
E.L(+119.04)FDEILEK.L	Y	46.81	1124.5753	8	-3.3	563.2931	2	18.57	3	4072	211015-MClass-FE2-3uL-LSM.raw	7.0599E6	1	1	49	56	Pyridylacetyl
R.ELFD(+21.98)EILEK.L	Y	42.08	1156.5627	9	-0.8	579.2882	2	17.65	3	3609	211015-MClass-FE2-3uL-LSM.raw	3.2157E6	1	1	48	56	Sodium adduct
L.FD(+14.02)EILEK.L	N	35.07	906.4698	7	-0.4	454.2420	2	19.00	3	4295	211015-MClass-FE2-3uL-LSM.raw	9.9621E5	1	1	50	56	Methylation(others)
**total 8 peptides**																	

Best Unique PSM (Scan 5226, m/z=571.3265, z=2, RT=20.98, ppm=-0.5):

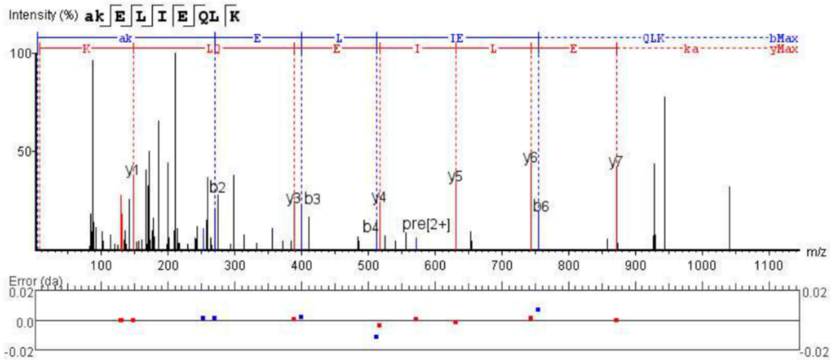

tr|A0A2L2Y2Y0|A0A2L2Y2Y0_PARTP

back to list

| Protein Coverage | Supporting Peptides | Best Unique PSM |

Protein Coverage:

Oxidation (M) (+

```
  1 MVDQATKDKLEAGFQKLQAATDCKSLLKKYLTKEVFDACKDKKTALGATLLDCIQSGVENLDSGVGIYAPDAEAYTLFAP
 81 IFNPIIEDYHEGFKPTDKHPPTDFGDINTLVNVDPTGEYVVSTRVRCGRSLKGYAFNPCLTEANYKEMEDKVSAIFGSFE
161 GELKGKYHPLTGMDKATQQQLIDDHFLFKEGDRFLQHANACRYWPTGRGIYHNDAKTFLVWCNEEDHLRIISMQKGGDLK
241 TIFQRLVNAVNIIESKLPFSRDDRLGYLTFCPTNLGTTIRASVHIALPKLAKDKKVLEDIAAKFNLQVRGTRGEHTESEG
                                                   331        342    349
321 GVYDISNKRRMGLTEYQAVKEMQDGILEMIKMEKAAA
```

Supporting Peptides:

Peptide	Uniq	-10lgP	Mass	Length	ppm	m/z	z	RT	Fraction	Scan	Source File	Area FE2 3uL	#Feature	#Feature FE2 3uL	Start	End	PTM	AScor
R.GEHTESEGGVYDISNK.R	N	61.87	1720.7540	16	-0.6	574.5916	3	15.81	3	2635	211015-MClass-FE2-3uL-LSM.raw	4.7197E5	1	1	313	328		
R.M(+15.99)GLTEYQAVK.E	N	58.08	1154.5641	10	0.4	578.2896	2	17.28	3	3389	211015-MClass-FE2-3uL-LSM.raw	3.0007E5	1	1	331	340	Oxidation (M)	M1:Oxdation (M):100.00
H.TESEGGVYDISNK.R	N	55.04	1397.6310	13	-0.9	699.8221	2	16.79	3	3126	211015-MClass-FE2-3uL-LSM.raw	4.2046E5	1	1	316	328		
K.EM(+15.99)QDGILEM(+15.99)IK.M	Y	51.80	1337.6207	11	-1.2	669.8168	2	20.88	3	5165	211015-MClass-FE2-3uL-LSM.raw	4.0435E5	1	1	341	351	Oxidation (M)	M2:Oxdation (M):100.00 M9:Oxdation (M):100.00
R.ASVHIALPK.L	Y	41.94	934.5599	9	-3.2	468.2857	2	18.63	3	4118	211015-MClass-FE2-3uL-LSM.raw	1.4555E5	1	1	281	289		
**total 5 peptides**																		

Best Unique PSM (Scan 5165, m/z=669.8168, z=2, RT=20.88, ppm=-1.2):

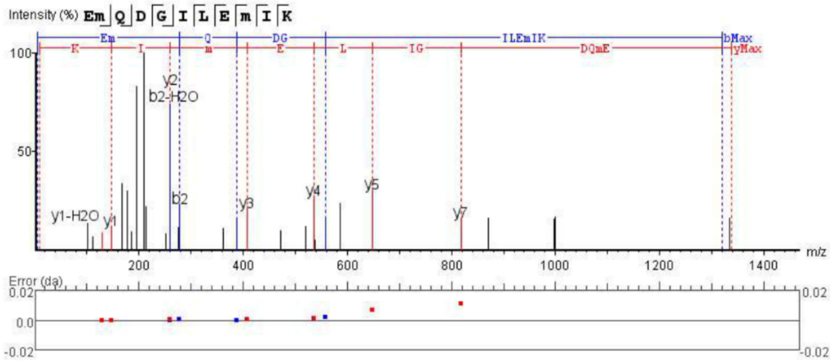

tr|A0A2L2Y7T9|A0A2L2Y7T9_PARTP

back to list

| Protein Coverage | Supporting Peptides | Best Unique PSM |

Protein Coverage:

1 SKMVDQAVLDKLEAGFRKLQEAKTCKSLLKKHLTK**EVFDKLK**TKQTAMGATLLDVIQSGVENLDSGVGVYAPDAESYTLF

81 GDLFNPIIEEYHMGFGSSDKHPAKDFGDVNTLVNVDPKGEFVISTRVRCGRSLEGYPFNPCLTEPQYKEMEQKVSGILSD

161 MSGELKGTYYPLTGMDKATQQKLIDDHFLFKEGDRFLQAANACRFWPSGRGIYHNDNKTFLVWSNEEDHLRIISMQKGGD

241 LKQIYKRLVDAVNIIESRLPFSHDDRLGFLTFCPTNLGTTIRASVHIKLPKLAKNKQQLEAVAAKFNLQVRGTR**GEHTES**

321 **EGGVYDISNK**RR**MGLTEYQAVK**EMQDGILELIKMEKSA

m Methylation(othe
o Oxidation (M) (+

Supporting Peptides:

Peptide	Uniq	-10lgP	Mass	Length	ppm	m/z	z	RT	Fraction	Scan	Source File	Area FE2 3uL	#Feature	#Feature FE2 3uL	Start	End	PTM	AScor
R.GEHTESEGGVYDISNK.R	N	61.87	1720.7540	16	-0.6	574.5916	3	15.81	3	2635	211015-MClass-FE2-3uL-LSM.raw	4.7197E5	1	1	315	330		
R.M(+15.99)GLTEYQAVK.E	N	58.08	1154.5641	10	0.4	578.2896	2	17.28	3	3389	211015-MClass-FE2-3uL-LSM.raw	3.0007E5	1	1	333	342	Oxidation (M)	M1:Oxdation (M):1(00.00
H.TESEGGVYDISNK.R	N	55.04	1397.6310	13	-0.9	699.8221	2	16.79	3	3126	211015-MClass-FE2-3uL-LSM.raw	4.2046E5	1	1	318	330		
K.EVFD(+14.02)KLK.T	Y	44.19	891.5065	7	-0.2	446.7604	2	20.24	3	4921	211015-MClass-FE2-3uL-LSM.raw	3.0521E5	1	1	36	42	Methylation(others)	D4:Mehylatic(others):26.2
**total 4 peptides**																		

Best Unique PSM (Scan 4921, m/z=446.7604, z=2, RT=20.24, ppm=-0.2):

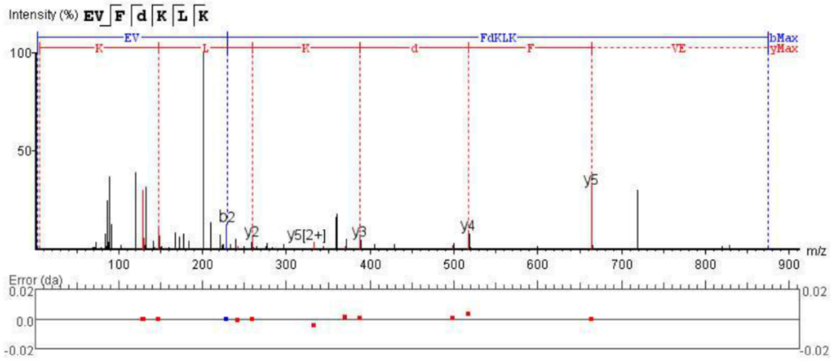

tr|E7D194|E7D194_LATHE

back to list

| Protein Coverage | Supporting Peptides | Best Unique PSM |

Protein Coverage:

1 GTRLRLSHPSGASIMTVKEKQAKILPLFKNLTALSPEPLPEAERDVRLKGVGVLPRGRLFSCFHEDHLGEAQALYETLYE

81 AKDFDDFINLAKQARDIVNEGLFAFALSVVVLHRDDCQGVVLPPIQEVFPDKFVPAETINRALKIDKQSTNEEKVISIQK

161 TGNILDPEYNLAYFREDIGINAHHWHWHLVYPATYRPDFFGKVKDR**KGELFYY**MHQQMCARYDCDRLSVGLRRMIPFQNF

241 EEKLEGYSAHLTSLISGLNYASRPAGMSLRDVR**EVDVQDMER**WRERILSAIHTGQVIDSNGK**EVPLDLER**GLDILGALIE

321 SSYESLNKGYYGT

Supporting Peptides:

Peptide	Uniq	-10lgP	Mass	Length	ppm	m/z	z	RT	Fraction	Scan	Source File	Area FE2 3uL	#Feature	#Feature FE2 3uL	Start	End	PTM	AScore	Found By
R.EVDVQDMER.W	Y	50.87	1119.4866	9	-0.1	560.7505	2	17.05	3	3259	211015-MClass-FE2-3uL-LSM.raw	2.3113E5	1	1	274	282			PEAKS DB
K.EVPLDLER.G	Y	46.81	969.5131	8	-0.5	485.7636	2	19.91	3	4754	211015-MClass-FE2-3uL-LSM.raw	1.5352E6	1	1	303	310			PEAKS DB
R.KGELFYY.M	N	40.10	918.4487	7	0.4	460.2318	2	22.70	3	5800	211015-MClass-FE2-3uL-LSM.raw	5.3772E5	1	1	207	213			PEAKS DB
**total 3 peptides**																			

Best Unique PSM (Scan 3259, m/z=560.7505, z=2, RT=17.05, ppm=-0.1):

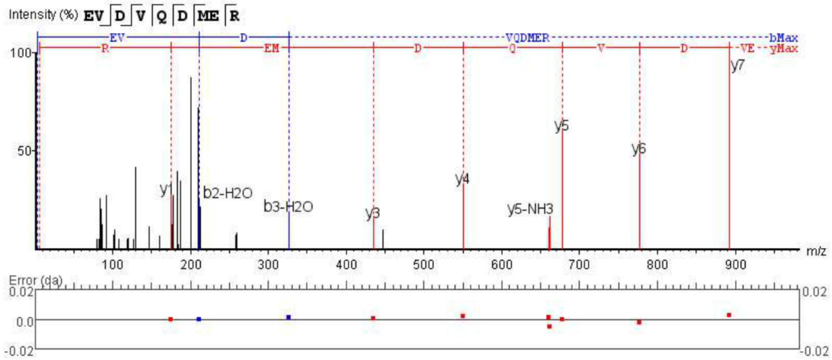

tr|A0A2L2YAY8|A0A2L2YAY8_PARTP back to list

| Protein Coverage | Supporting Peptides | Best Unique PSM |

Protein Coverage:

1 DHVGTLNAQASCADAVSYCGAKDQKYPDKKSMGYPFDRIIRARTIAEFSTQNMNFQEVKIQFKE

o Oxidation (M) (+

Supporting Peptides:

Peptide	Uniq	-10lgP	Mass	Length	ppm	m/z	z	RT	Fraction	Scan	Source File	Area FE2 3uL	#Feature	#Feature FE2 3uL	Start	End	PTM	AScore	Found By
K.SM(+15.99)GYPFDR.I	Y	61.26	987.4120	8	0.1	494.7133	2	17.86	3	3703	211015-MClass-FE2-3uL-LSM.raw	3.3522E6	1	1	31	38	Oxidation (M)	M2:Oxidation (M):1000.00	PEAKS DB
K.SMGYPFDR.I	Y	51.81	971.4171	8	-4.4	486.7137	2	20.27	3	4916	211015-MClass-FE2-3uL-LSM.raw	4.0329E5	1	1	31	38			PEAKS DB
S.M(+15.99)GYPFDR.I	Y	39.56	900.3799	7	-0.8	451.1969	2	17.91	3	3757	211015-MClass-FE2-3uL-LSM.raw	0	0	0	32	38	Oxidation (M)	M1:Oxidation (M):1000.00	PEAKS DB
**total 3 peptides**																			

Best Unique PSM (Scan 3703, m/z=494.7133, z=2, RT=17.86, ppm=0.1):

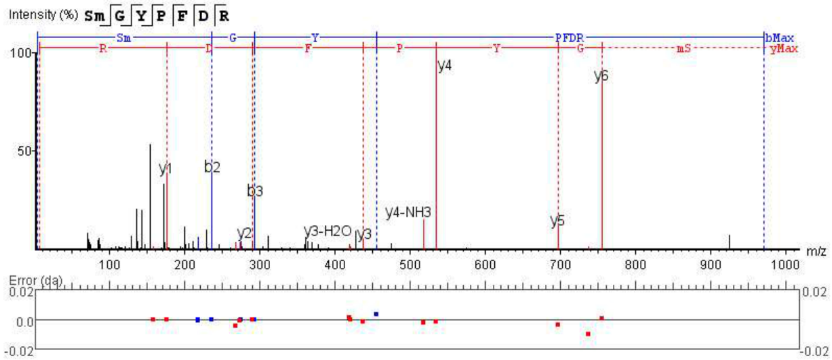

tr|A0A2L2XYG9|A0A2L2XYG9_PARTP back to list

| Protein Coverage | Supporting Peptides | Best Unique PSM |

Protein Coverage:

1 ECEKRREEAQKHETIMKLIHKCKENGDYEELQCYKDSTFCVCYTKDGRPASPNLSNLKECGCFLEKQRKIDSNHENPYLP

81 QCQDDGSWNPKQCYDYNNSCWCVDKNGKQVGESKHEGKCLDC

c Carbamidomethy
d Deamidation (NQ

Supporting Peptides:

Peptide	Uniq	-10lgP	Mass	Length	ppm	m/z	z	RT	Fraction	Scan	Source File	Area FE2 3uL	#Feature	#Feature FE2 3uL	Start	End	PTM
K.ENGDYEELQC(+57.02)YK.D	Y	76.08	1546.6245	12	-0.6	774.3191	2	18.43	3	4011	211015-MClass-FE2-3uL-LSM.raw	1.5774E5	1	1	24	35	Carbamidomethyl
**total 2 peptides**																	

Peptide	Uniq	-10lgP	Mass	Length	ppm	m/z	z	RT	Fraction	Scan	Source File	Area FE2 3uL	#Feature	#Feature FE2 3uL	Start	End	PTM
K.EN(+.98)GDYEELQC(+57.02)YK.D	Y	51.90	1547.6085	12	-1.4	774.8104	2	18.75	3	4167	211015-MClass-FE2-3uL-LSM.raw	1.4721E5	1	1	24	35	Deamidation (NQ) Carbamidomethyl
total 2 peptides																	

Best Unique PSM (Scan 4011, m/z=774.3191, z=2, RT=18.43, ppm=-0.6):

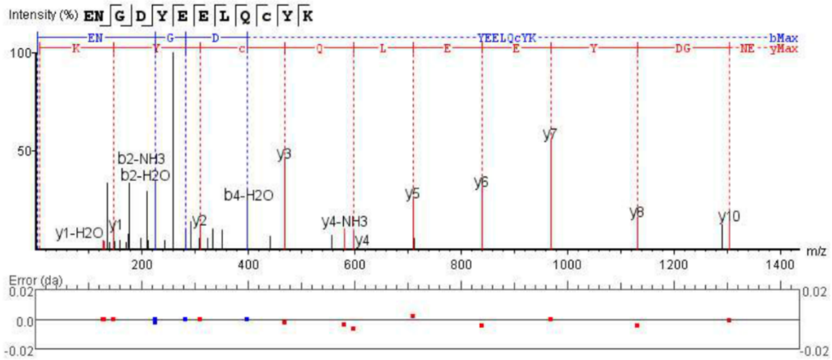

tr|A0A2L2Y4H8|A0A2L2Y4H8_PARTP

back to list

| Protein Coverage | Supporting Peptides | Best Unique PSM |

Protein Coverage:

Oxidation (M) (+

1 MPADAAEKQRRVLPLFEYCTLPTSTKFGLKVERDAKLQGLGVLGRGRLFSSFQQDHLIEANRLAEVLLGAETFDEFVDLC
81 HQARDFVNEGLFVYAVSVAVLHRNDCRGISLPPVQEVFPDKFFPVETIYKAIKVANAHPDKDDEIIVDVEATGNILDPEY
161 NLAYYREDVGINAHHWHWHLVYPSTWNAAVTGKAKDR**KGEIFYY**MHQQMCARYDCERLSNGMPR**MIPFHNF**HEGLEGYSA (225)
241 HLSSIINGLPYASRPSGMSLQDYQGVGVQNLERWRERILDAINLGYVTDADGKETILDETHGIDILGDIIESSHESKNPE
321 FYGSLHNWGHVLISNVLDPDHRFLNNPGVMDDATTSLRDPIFYRWHRFIDDMFNEYKKKLEPYDPDELGFPSVEIESVRV
401 AAHQDNIVTTTFEEDELDLSNAFNFGRNGSVKIRYQHLDHEPFAYDIKIQNKSTKVCHGTIRIFLGPVFDELGNKIKIDD
481 IRRLMIELDRFSAVLNPGVNDIHRTSRESSVTINKERKFEQLLRGEGTNEHSSEFCSCGWPDHLLVPKGNDKGMKFHLFV
561 IVTDHLKDLVGQLTDKNICADAVSYCGVKDDLYPDRK**SMGFPFDR**SIKKDSKEWLLPNMKATEVTIVHSARH

Supporting Peptides:

Peptide	Uniq	-10lgP	Mass	Length	ppm	m/z	z	RT	Fraction	Scan	Source File	Area FE2 3uL	#Feature	#Feature FE2 3uL	Start	End	PTM	AScore	Found By
R.MIPFHNF.H	N	42.05	904.4265	7	1.0	453.2210	2	23.56	3	6060	211015-MClass-FE2-3uL-LSM.raw	1.3967E5	1	1	225	231			PEAKS DB
K.SMGFPFDR.S	Y	41.25	955.4222	8	-0.6	478.7181	2	20.81	3	5164	211015-MClass-FE2-3uL-LSM.raw	1.1146E6	1	1	598	605			PEAKS DB
R.M(+15.99)IPFHNF.H	N	40.14	920.4214	7	-0.5	461.2178	2	21.88	3	5542	211015-MClass-FE2-3uL-LSM.raw	3.7742E6	1	1	225	231	Oxidation (M)	M1:Oxidation (M):1000.00	PEAKS DB
R.KGEIFYY.M	N	40.10	918.4487	7	0.4	460.2318	2	22.70	3	5800	211015-MClass-FE2-3uL-LSM.raw	5.3772E5	1	1	198	204			PEAKS DB
total 4 peptides																			

Best Unique PSM (Scan 5164, m/z=478.7181, z=2, RT=20.81, ppm=-0.6):

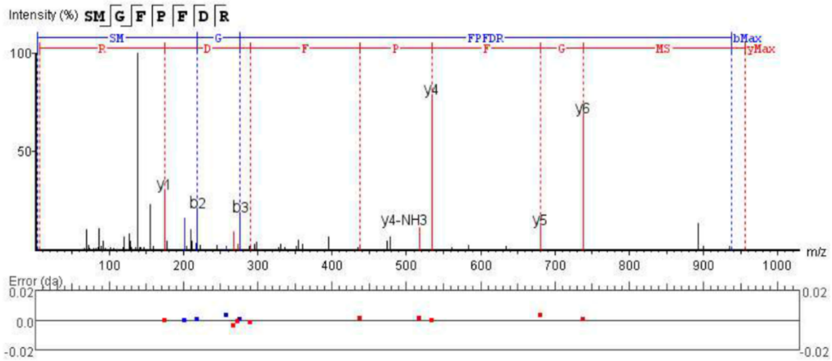

tr|A0A2L2Z5H2|A0A2L2Z5H2_PARTP

back to list

| Protein Coverage | Supporting Peptides | Best Unique PSM |

Protein Coverage:

1 FADDDVKEELKKKLK**ERFDEILEK**VKEALENGKVLSEENLKKLKDIRQKMKDLHVDLGNQAKELLEKLKDKDKAYWQKIL

81 DRL

Supporting Peptides:

Peptide	Uniq	-10lgP	Mass	Length	ppm	m/z	z	RT	Fraction	Scan	Source File	Area FE2 3uL	#Feature	#Feature FE2 3uL	Start	End	PTM	AScore	Found By
R.FDEILEK.V	N	49.23	892.4542	7	0.2	447.2344	2	18.91	3	4244	211015-MClass-FE2-3uL-LSM.raw	9.1588E7	1	1	18	24			PEAKS DB
K.ERFDEILEK.V	Y	40.61	1177.5979	9	0.4	589.8065	2	19.83	3	4743	211015-MClass-FE2-3uL-LSM.raw	9.4054E6	1	1	16	24			PEAKS DB
total 2 peptides																			

Best Unique PSM (Scan 4743, m/z=589.8065, z=2, RT=19.83, ppm=0.4):

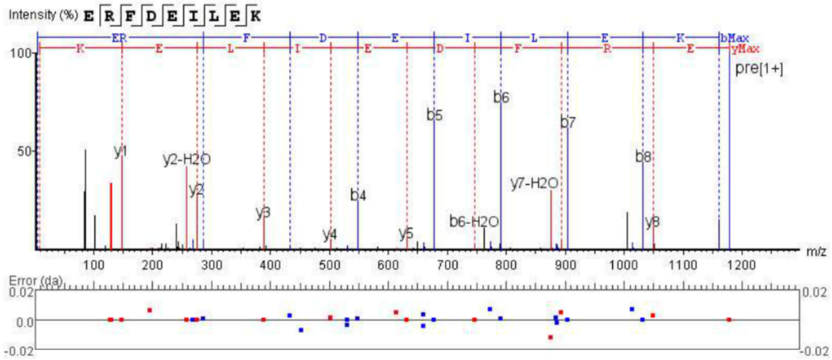

tr|A0A2L2XZN5|A0A2L2XZN5_PARTP

back to list

| Protein Coverage | Supporting Peptides | Best Unique PSM |

Protein Coverage:

1 MLKIVGILALCLFATAFAADDEKEPSACELDRARRLNATLTESILHLIPECEENGDYAALQCFTANDWCVCYRRNGDNIN

C Carbamidomethy
V I->V

81 TPSKNIKACDCVRQK**DDAITAGDTYIPK**CDKNGYFQSKQCSN**DECWCVDK**NGKVLTDPKTGGVDC

Supporting Peptides:

Peptide	Uniq	-10lgP	Mass	Length	ppm	m/z	z	RT	Fraction	Scan	Source File	Area FE2 3uL	#Feature	#Feature FE2 3uL	Start	End	PTM
K.DDAV(sub I)TAGDTYIPK.C	Y	42.63	1364.6460	13	-2.0	683.3289	2	19.37	3	4517	211015-MClass-FE2-3uL-LSM.raw	3.1399E5	1	1	96	108	
N.DEC(+57.02)WC(+57.02)VDK.N	Y	41.99	1110.4110	8	-0.2	556.2126	2	17.43	3	3479	211015-MClass-FE2-3uL-LSM.raw	3.9213E5	1	1	123	130	Carbamidomethylat
total 2 peptides																	

Best Unique PSM (Scan 4517, m/z=683.3289, z=2, RT=19.37, ppm=-2.0):

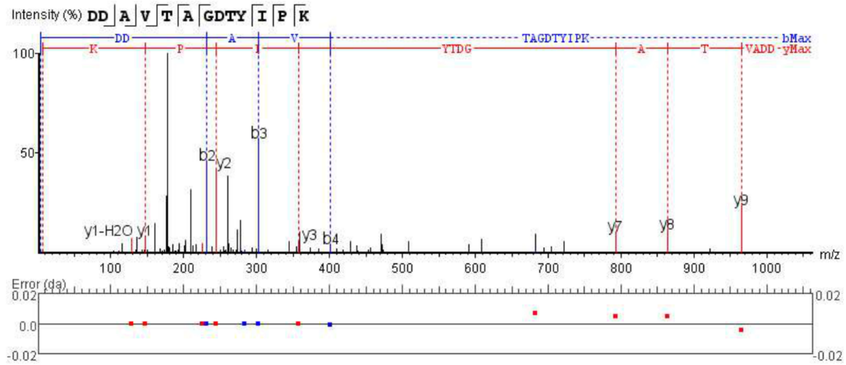

tr|A0A2L2Y372|A0A2L2Y372_PARTP back to list

| Protein Coverage | Supporting Peptides | Best Unique PSM |

Protein Coverage:

23

1 MRVFLALFVITLCVLATNA**ALTCFGDEEK**CSAEECCIQVGNTPVGICKKKHEIDQICEMKPIKHLFKKHVFKVRCPCKDE Carbamidomethy

81 LKCVSSKGGILGKIAGKCQENSEEEE

Supporting Peptides:

Peptide	Uniq	-10lgP	Mass	Length	ppm	m/z	z	RT	Fraction	Scan	Source File	Area FE2 3uL	#Feature	#Feature FE2 3uL	Start	End	PTM	ASc
A.ALTC(+57.02)FGDEEK.C	Y	59.35	1168.5070	10	0.4	585.2610	2	17.73	3	3639	211015-MClass-FE2-3uL-LSM.raw	3.8482E5	1	1	20	29	Carbamidomethylation	C4:bam metatio 000
total 1 peptides																		

Best Unique PSM (Scan 3639, m/z=585.2610, z=2, RT=17.73, ppm=0.4):

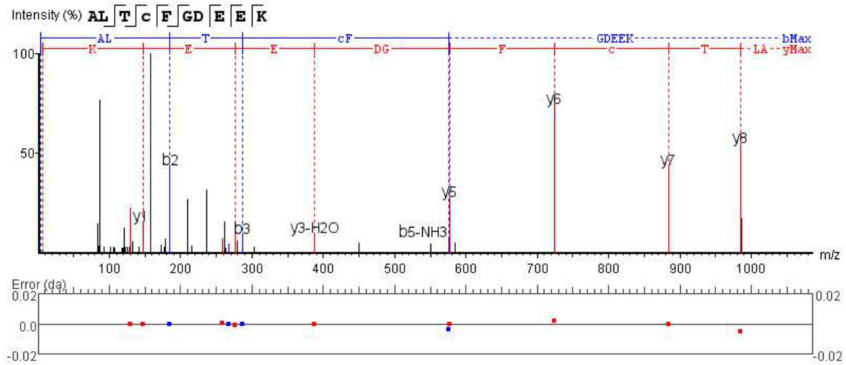

tr|A0A2L2Y2N9|A0A2L2Y2N9_PARTP back to list

| Protein Coverage | Supporting Peptides | Best Unique PSM |

Protein Coverage:

23

1 MRVFLALLVITLCALAANA**ALTCFGDEEK**CSAEECCIQVGNTPVGICKKKHEIDQICEMKPIKHLFKKHVFKVRCPCKDE Carbamidomethy

81 LKCVSSKGVIIGKFAGKCQENSGEEEE

Supporting Peptides:

Peptide	Uniq	-10lgP	Mass	Length	ppm	m/z	z	RT	Fraction	Scan	Source File	Area FE2 3uL	#Feature	#Feature FE2 3uL	Start	End	PTM	ASc
A.ALTC(+57.02)FGDEEK.C	Y	59.35	1168.5070	10	0.4	585.2610	2	17.73	3	3639	211015-MClass-FE2-3uL-LSM.raw	3.8482E5	1	1	20	29	Carbamidomethylation	C4:bam metatio 000
total 1 peptides																		

Best Unique PSM (Scan 3639, m/z=585.2610, z=2, RT=17.73, ppm=0.4):

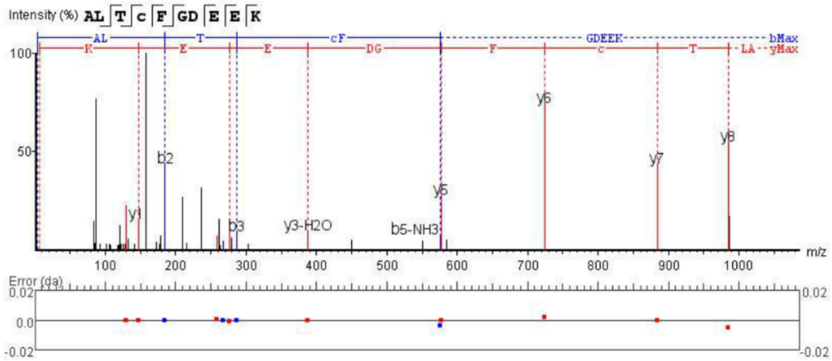

tr|A0A2L2Y2N1|A0A2L2Y2N1_PARTP

back to list

| Protein Coverage | Supporting Peptides | Best Unique PSM |

Protein Coverage:

23

1 MRVFLALLVITLCALAANA**ALTCFGDEEK**CSAEECCIQVGNTPVGICKKKHEIDQICEMKPIKHLFKKHVFKVRCPCKDE C Carbamidomethy

81 LKCVSSKGGIIGKFAGKCQENSGEEEE

Supporting Peptides:

Peptide	Uniq	-10lgP	Mass	Length	ppm	m/z	z	RT	Fraction	Scan	Source File	Area FE2 3uL	#Feature	#Feature FE2 3uL	Start	End	PTM	ASc
A.ALTC(+57.02)FGDEEK.C	Y	59.35	1168.5070	10	0.4	585.2610	2	17.73	3	3639	211015-MClass-FE2-3uL-LSM.raw	3.8482E5	1	1	20	29	Carbamidomethylation	C4:(bam met atio 000
**total 1 peptides**																		

Best Unique PSM (Scan 3639, m/z=585.2610, z=2, RT=17.73, ppm=0.4):

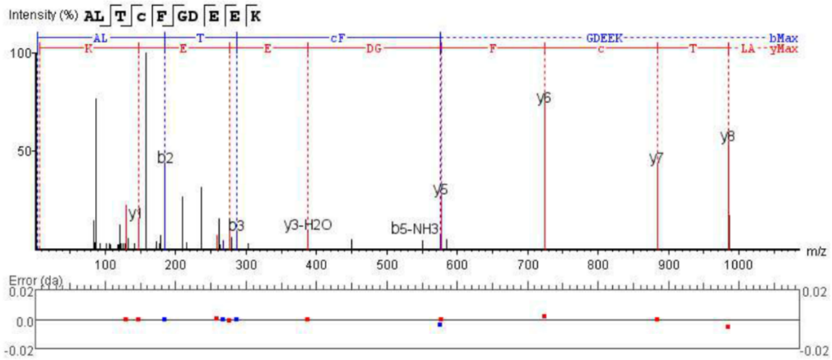

tr|A0A2L2YL93|A0A2L2YL93_PARTP

back to list

| Protein Coverage | Supporting Peptides | Best Unique PSM |

Protein Coverage:

25 27

1 MKLIPKCKDKGDYEELQCYNDSK**FCVCYDK**KGHPASPILSKLTDCGCFLERKRKIDSNPGGAAAAAAAAEAGSSPVYLSL C Carbamidomethy

81 GCSFVHVVFFFGVLVLCVY

Supporting Peptides:

Peptide	Uniq	-10lgP	Mass	Length	ppm	m/z	z	RT	Fraction	Scan	Source File	Area FE2 3uL	#Feature	#Feature FE2 3uL	Start	End	PTM
K.FC(+57.02)VC(+57.02)YDK.K	Y	52.42	990.3939	7	-0.8	496.2039	2	17.79	3	3681	211015-MClass-FE2-3uL-LSM.raw	4.5462E5	1	1	24	30	Carbamidomethylation
**total 1 peptides**																	

Best Unique PSM (Scan 3681, m/z=496.2039, z=2, RT=17.79, ppm=-0.8):

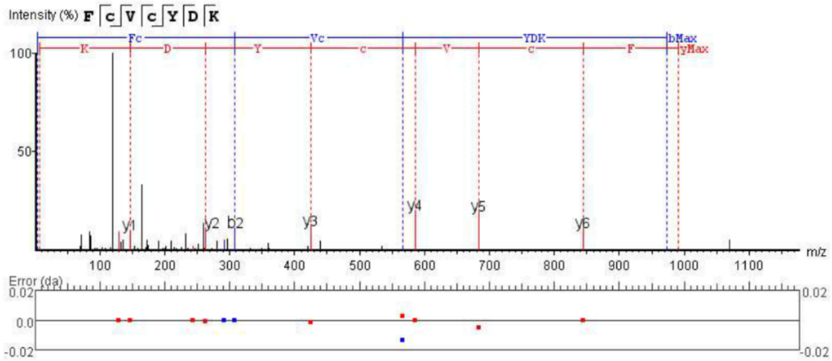

tr|A0A2L2Z3D2|A0A2L2Z3D2_PARTP

back to list

| Protein Coverage | Supporting Peptides | Best Unique PSM |

Protein Coverage:

1 KAVTEILEKLKDARENGKVVK**EDILEKLK**EIRQMLKDLKQDISILAKELLDKLMD

Supporting Peptides:

Peptide	Uniq	-10lgP	Mass	Length	ppm	m/z	z	RT	Fraction	Scan	Source File	Area FE2 3uL	#Feature	#Feature FE2 3uL	Start	End	PTM	AScore	Found By
K.EDILEKLK.E	Y	44.99	986.5648	8	-2.9	494.2882	2	20.24	3	4908	211015-MClass-FE2-3uL-LSM.raw	7.4344E5	1	1	22	29			PEAKS DB
total 1 peptides																			

Best Unique PSM (Scan 4908, m/z=494.2882, z=2, RT=20.24, ppm=-2.9):

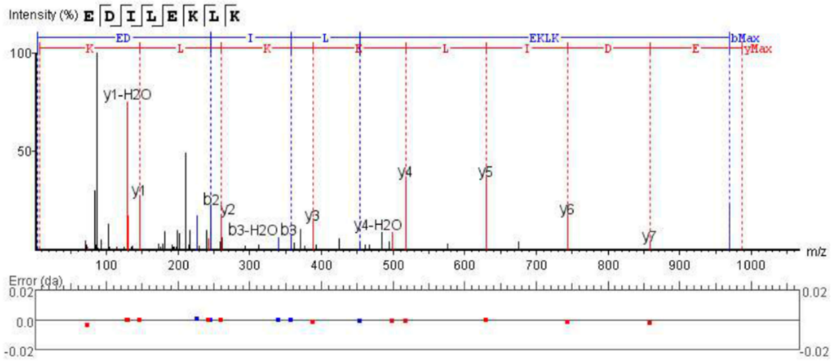

tr|E7D1N7|E7D1N7_LATHE

back to list

| Protein Coverage | Supporting Peptides | Best Unique PSM |

Protein Coverage:

1 GTRQRFTETEKMQFLILLCAVVGALAANECPANSHFEECGTACPLTCENYKNPPKFCVLMCKIGCQCDAGYVKAQDGSCV

81 LPEQCPAQASVEKTCLGEPETGFCRGYFPKYYYDVQSGTCKEFIYGGCGGNGNRYETEEECLEQCGDVKLKQSEAEVCDL

161 PAETGLCRGYFKRYAFDKASGQCK**QFIYGGCGGNK**NNFRTVQDCEKTCKAVASVSEVCEQEKVVGPCRAAFRRYFFNKAT

241 GQCELFIYGGCRGNDNNFGTKEECESVCLA

191 C Carbamidomethy
E Q->E

Supporting Peptides:

Peptide	Uniq	-10lgP	Mass	Length	ppm	m/z	z	RT	Fraction	Scan	Source File	Area FE2 3uL	#Feature	#Feature FE2 3uL	Start	End	PTM	AS
K.E(sub Q)FIYGGC(+57.02)GGNK.N	Y	42.93	1200.5233	11	-1.1	601.2682	2	17.70	3	3655	211015-MClass-FE2-3uL-LSM.raw	4.9227E4	1	1	185	195	Carbamidomethylation	C7 ba me ati 00
total 1 peptides																		

Best Unique PSM (Scan 3655, m/z=601.2682, z=2, RT=17.70, ppm=-1.1):

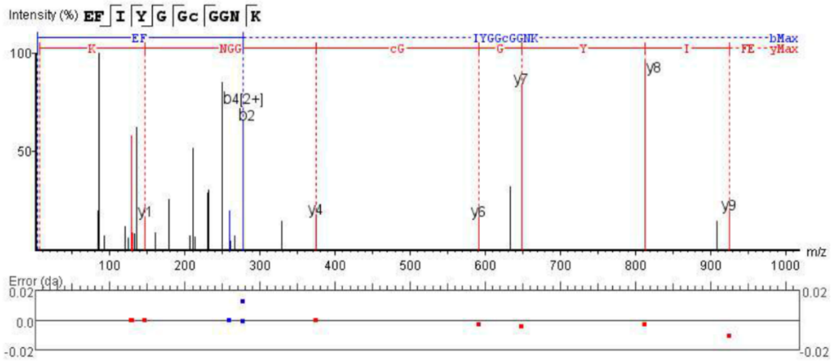

tr|A0A2L2Z3P8|A0A2L2Z3P8_PARTP

back to list

| Protein Coverage | Supporting Peptides | Best Unique PSM |

Protein Coverage:

1 RSSLALSPR**EIIEQLKEK**VGGFWKRILDKLKDTAEYAADDESRDLRKRFEDLLE

Supporting Peptides:

Peptide	Uniq	-10lgP	Mass	Length	ppm	m/z	z	RT	Fraction	Scan	Source File	Area FE2 3uL	#Feature	#Feature FE2 3uL	Start	End	PTM	AScore	Found By
R.EIIEQLKEK.V	Y	41.26	1128.6390	9	-0.7	565.3264	2	17.52	3	3528	211015-MClass-FE2-3uL-LSM.raw	5.0592E5	1	1	10	18			PEAKS DB
total 1 peptides																			

Best Unique PSM (Scan 3528, m/z=565.3264, z=2, RT=17.52, ppm=-0.7):

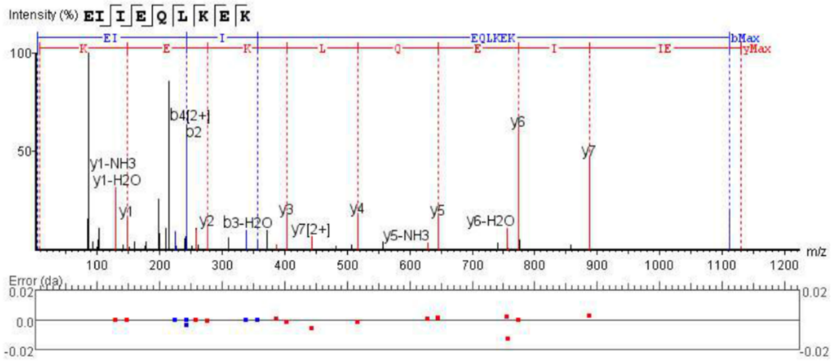

tr|A0A2L2Y3F9|A0A2L2Y3F9_PARTP

back to list

| Protein Coverage | Supporting Peptides | Best Unique PSM |

Protein Coverage:

1 MAYCWAFMFVGILYQNAWANPMENEGLFEGDIAGIDPYALTDRNAVVDPAELWPDGVVYYEIDWKLRDVKDIIQEAIDEY

81 ESKTCLRFKKRTATTKDYIKMTIVTGCWSSVGRK**GGEQELSLSEGCHDK**VSAVHELGHAIGLWHEHSRSDRDDYLEILWG

161 NIKPGSEHNFLKLKPWENNLLGEKLDYKSIMLYGEYAFAKDRKSMTMRPREEGAVIGLINNKPGLSDSDVRRINRLYECN

241 GEKRPPPPEVPDFKCDFETDMCDMVNAENNGKTEWRIEKGTLGGRDGSYVYVKASEASFRKVRLITPFFGAFGRKKGCFK

321 FDVYFNGGGVVSLDVSVHNINTSNLVVKHVDKKDEWQSVQINVNLEGDVKFSLDAKTRKSDGEGIIALDNIVYQLREC

126 C Carbamidomethy

Supporting Peptides:

Peptide	Uniq	-10lgP	Mass	Length	ppm	m/z	z	RT	Fraction	Scan	Source File	Area FE2 3uL	#Feature	#Feature FE2 3uL	Start	End	PTM
K.GGEQELSLSEGC(+57.02)HDK.V	Y	40.62	1644.7050	15	0.5	549.2426	3	15.75	3	2625	211015-MClass-FE2-3uL-LSM.raw	5.9589E4	1	1	115	129	Carbamidomethylati
total 1 peptides																	

Best Unique PSM (Scan 2625, m/z=549.2426, z=3, RT=15.75, ppm=0.5):

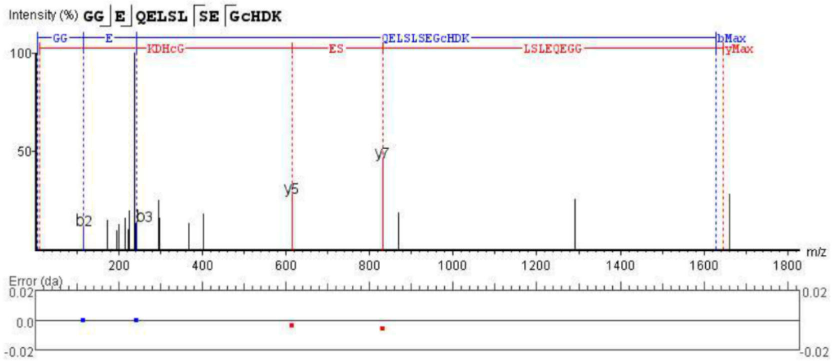

tr|A0A2L2YQH5|A0A2L2YQH5_PARTP

back to list

| Protein Coverage | Supporting Peptides | Best Unique PSM |

Protein Coverage:

1 MALLDYENQMYLESFHDDALVVLAKGIGAERLAVNLIKTYNDPGNLVLVIGSTTV**DEEFIIEK**LKSLGETLLPKVITSDV
81 GTKDRRNVYMEGGVLFVSSRILVVDMLVDRVPIDLITGIIVLRAHKILESCQEAFIVRLYRMKNNKGFIKAISSNPSAFT
161 RGFAQVQRVMRNLFVKHLFIWPRFHAVVASSLERAKPDVMEMHVPMTLAMTSIQISLLDLMNVCVKELKHINPSLDTDEL
241 TVENAIAKSFDKIIKYHLYPIWHQLIAMTMRLIAYLKFLRSVLL

Supporting Peptides:

Peptide	Uniq	-10lgP	Mass	Length	ppm	m/z	z	RT	Fraction	Scan	Source File	Area FE2 3uL	#Feature	#Feature FE2 3uL	Start	End	PTM	AScore	Found By
V.DEEFIIEK.L	Y	39.91	1021.4968	8	2.1	511.7567	2	20.74	3	5092	211015-MClass-FE2-3uL-LSM.raw	6.935E6	1	1	56	63			PEAKS DB
total 1 peptides																			

Best Unique PSM (Scan 5092, m/z=511.7567, z=2, RT=20.74, ppm=2.1):

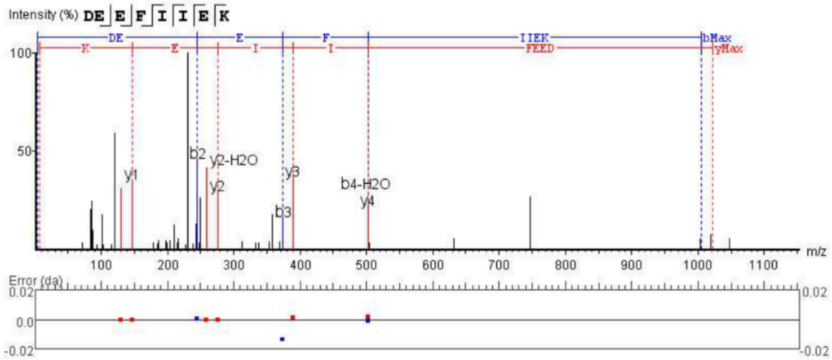

tr|A0A2L2Y4C8|A0A2L2Y4C8_PARTP

back to list

| Protein Coverage | Supporting Peptides | Best Unique PSM |

Protein Coverage:

1 SVALYSFNSLQAKEMIDCHDCRMKRLPKAHELPLYDKNEDMCLEYDGKTKLPLISEISGVRQQLSGLASYLNVFKDQVVH
81 VYETGIAHSQSSYDYLTSEENKTTRILAICSGGLLGLLLARKRGIFKKIIYTSTGTGLVFSAFYPSLAMDYASTGWEHTK
161 **TYTFDALEK**YGGYDTQKLSNDYNEKVEAIKSTLKLEGVMDQLKEWFDKNKEKVATTLNMKTESKEK

Supporting Peptides:

Peptide	Uniq	-10lgP	Mass	Length	ppm	m/z	z	RT	Fraction	Scan	Source File	Area FE2 3uL	#Feature	#Feature FE2 3uL	Start	End	PTM	AScore	Found By
K.TYTFDALEK.Y	Y	39.43	1086.5233	9	1.5	544.2698	2	21.47	3	5387	211015-MClass-FE2-3uL-LSM.raw	2.3804E7	1	1	161	169			PEAKS DB
total 1 peptides																			

Best Unique PSM (Scan 5387, m/z=544.2698, z=2, RT=21.47, ppm=1.5):

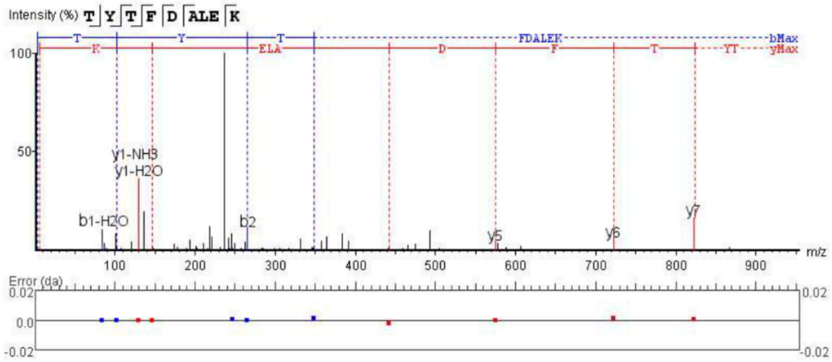

tr|A0A2L2Z6L1|A0A2L2Z6L1_PARTP

back to list

| Protein Coverage | Supporting Peptides | Best Unique PSM |

Protein Coverage:

1 RDRTGDLLRKILENFGFHKRSAVDHAEDIMAEDEHADLSDILKKKEDLEELHKKAIDALDNGSTATEYLERIQHIRDALK
81 LLNIYLVEKAKEYLLVLKEKVK**DYWNKLK**E

Supporting Peptides:

Peptide	Uniq	-10lgP	Mass	Length	ppm	m/z	z	RT	Fraction	Scan	Source File	Area FE2 3uL	#Feature	#Feature FE2 3uL	Start	End	PTM	AScore	Found By
K.DYWNKLK.E	Y	36.64	965.4971	7	-0.5	483.7556	2	18.37	3	3984	211015-MClass-FE2-3uL-LSM.raw	1.2076E5	1	1	103	109			PEAKS DB
total 1 peptides																			

Best Unique PSM (Scan 3984, m/z=483.7556, z=2, RT=18.37, ppm=-0.5):

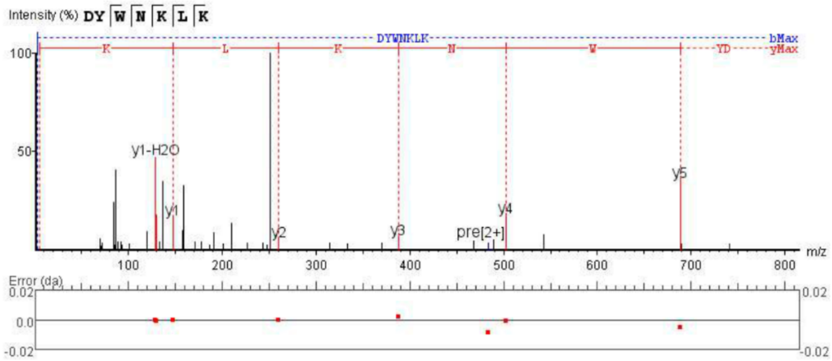

tr|A0A2L2YL07|A0A2L2YL07_PARTP

back to list

| Protein Coverage | Supporting Peptides | Best Unique PSM |

Protein Coverage:

1 FWIRILDIFGGSNRSVVDIDDFHLLF**ADDDVKDDLKK**KLKERLAEILEKVKEALENGKGLREETIKKLKEIREKLRVLKV
81 DLGDKAKEL

Supporting Peptides:

Peptide	Uniq	-10lgP	Mass	Length	ppm	m/z	z	RT	Fraction	Scan	Source File	Area FE2 3uL	#Feature	#Feature FE2 3uL	Start	End	PTM	AScore	Found By
F.ADDDVKDDLKK.K	Y	35.44	1260.6198	11	-0.8	631.3167	2	16.09	3	2761	211015-MClass-FE2-3uL-LSM.raw	3.7417E5	1	1	27	37			PEAKS DB
total 1 peptides																			

Best Unique PSM (Scan 2761, m/z=631.3167, z=2, RT=16.09, ppm=-0.8):

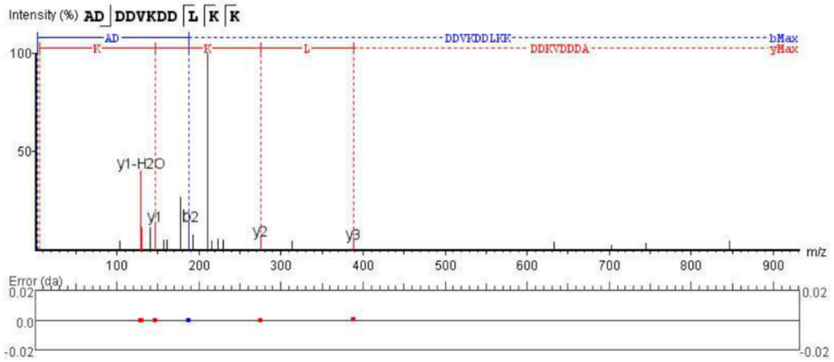

tr|A0A2L2Y270|A0A2L2Y270_PARTP **back to list**

| **Protein Coverage** | **Supporting Peptides** | **Best Unique PSM** |

Protein Coverage:

C Carbamidomethy
T A->T

```
   1 FSISSPSHYGTGSPHTLDFKWKRGVKAEFSIDTSYMEGQFAVENQSPVRSIKAEFNLKKVLNKKISVIGSVTPGDPNLLN
  81 VEVEWEGDGSQKKVVIDAKSNKGTEDGIEKYMITATVNYVGAISVDITGKVSTDLLRGPHYFRADYTAKEPIAIEFTHEI
 161 KNGKLVSFGRYLNNNAERLRMDIKGKMELTGYKFELDGGIFMTSPYKSLDGKEISFRIMADSTDAVRTFISEYRIMPVSF
 241 INYAGKLEYTRKRGWPGQIKSIALVSVHNRPSIEATTTIDYGNGKYSLKSSLTPLSRRKINLVSTFEHSGRFAAFHHSLA
 321 TSFEYLQTVDLKAVADLRNMEDAKIHSNFDINKHKLYDVNSTLKFRTLIDFDGQLTIYSKITPSLKLFYKTQASGQISKY
 401 EMNLDVDSQSLMTGSGEIKKRKKGFNGDLIFKYREKEFLALTISQEAKSQRERNYVIKSKTPWRNHIGSIKVMKDKKGSS
 481 KYETKFCREKEEDKCISIDLAHKEIPDGNEWNVCYKRQEVDFCIERIRADTKEVSRYHTVIYKGDTRYGYDVRFGKEGNG
 561 LMTSAGLILPSREIVNKLIVEFSLSKPKLKWELKLDAAKNPDRKLNVDLRFDNHLTDNQASKLDITITHPIYEKPIEIKL
 641 VGDLELSQNKIFTGEMSLDYSNDPEDKLIGKLAVEKGPVPTAILNIYHQDEQHLDFVVKLNGTVNENEEYFGLIWNWKDD
 721 SNVDQEAFIVFNWLFKERKISFAYQRPKFFYRLDADIVASQTLLDESQVTDVTSNLNGEISKYKIINDYATGCNKLYVLN
 801 DDGENKRSYELCHNEDGRKILSVLVEGLDDENQWTFQYSLEIIRKNWWTIGIINNFNPEFLGQTLFRLSALGDELTAAGV
 881 GTLPISDTARYKKIKSSFTSKIVQPSLRFFVPEATKFISGMIRDIQTLRQKLTFYYHTLPSFEEIRHCGKKVLAISQAIA
 961 KEIWNVVGSYYKNYLETTLKTVHKFFELVKKACDDNVECKAFVKAYHDDGFKGLVKQLRDYLLRLPDRIQHFLSQEHPEL
1041 RNFMGKAVTLLKEFLEPLTKYRCGEIVVKVVKLSYEKLEPIAQRFLRDFPSYYRAVRGKIVSNPYVIQAAKLAKETYLNI
1121 RETLLKIDYKKLYKLAIEKLEECLLNVNPPSLKKSFKVLALNTEKGIAEFEVYLATSHPLPRKIIDYTVSTTINIVKRLA
1201 DIQPSWKTVLVKVKRVADLNFFPPYKAQAMILGNQHYVTFDKEFFDFAGECTYLLTRDFEDGNFTFALTPKEGEKSPSIL
1281 AWINDASIELNPADKSVLVGDKAVELPYISKDFVVSRSGNAITINDRHGVEVKCNLVHELCTFSITGWYFGKTAGLLGTY
1361 NYEPKDDFKRPKGQIANSATVHAKSWELKKGCKSNNLVSEPRINERSDYYKLCRQHFVDDASPLSACYSEVSPADYMQLC
                           1465
                              C
1441 LRHLASASDSTKALCRVSAAYVAECERNYVELSLPNKCLVCTGPDGSTLEHSDKKKYENLSKKSSDVVFIIEDHECNKAV
                           T  C
1521 IGELSNIARSIDRELQTEGFKDNMFGVIGYGGDVGSPEIFTVGGKTFFNSRDINSIVGRISLQSNKVESSRAFEAMKLAV
1601 TYLSRSDTSKNFVLFSCSSCKYDFKSLQYPVVQQILLERGISLHVVHSAEISIRKSKEKDIIGVDPDTVYHSKDVTQREL
1681 VGEPALRSQVSIPKDLCIALSQDVGGSFFSTYPLSKGTSDAKNWRSVFSRKFIKAVQSFKCQWCDCSSTRDHIPNTVCQP
1761 CETK
```

Supporting Peptides:

Peptide	Uniq	-10lgP	Mass	Length	ppm	m/z	z	RT	Fraction	Scan	Source File	Area FE2 3uL	#Feature	#Feature FE2 3uL	Start	End	PTM	AScore	F
R.VSAAYVT(sub A)EC(+57.02)ER.N	Y	34.98	1283.5815	11	-0.6	642.7977	2	16.44	3	2952	211015-MClass-FE2-3uL-LSM.raw	3.8316E4	1	1	1457	1467	Carbamidomethylation	C9:Carbamidomethylation:1000.00	S
total 1 peptides																			

Best Unique PSM (Scan 2952, m/z=642.7977, z=2, RT=16.44, ppm=-0.6):

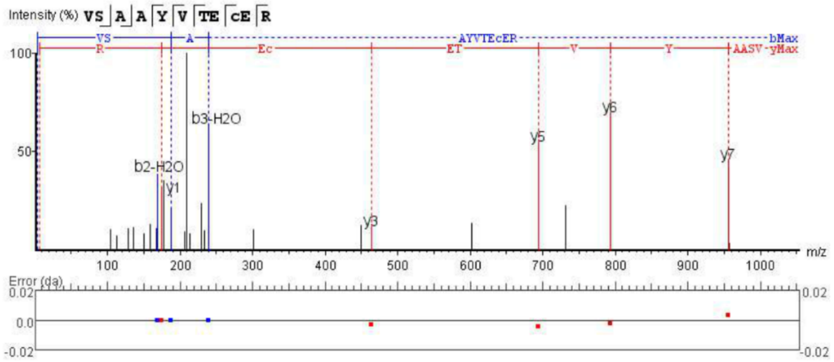

tr|A0A2L2Y1P7|A0A2L2Y1P7_PARTP back to list

| Protein Coverage | Supporting Peptides | Best Unique PSM |

Protein Coverage:

C Carbamidomethy
T A->T

```
   1 FSISSPSHYGTGSPHTLDFKWKRGVKAEFSIDTSYMEGQFAVENQSPVRSIKAEFNLKKVLNKKISVIGSVTPGDPNLLN
  81 VEVEWEGDGSQKKVVIDAKSNKGTEDGIEKYMITATVNYVGAISVDITGKVSTDLLRGPHYFRADYTAKEPIAIEFTHEI
 161 KNGKLVSFGRYLNNNAERLRMDIKGKMELTGYKFELDGGIFMTSPYKSLDGKEISFRIMADSTDAVRTFISEYRIMPVSF
 241 INYAGKLEYTRKRGWPGQIKSIALVSVHNRPSIEATTTIDYGNGKYSLKSSLTPLSRRKINLVSTFEHSGRFAAFHHSLA
 321 TSFEYLQTVDLKAVADLRNMEDAKIHSNFDINKHKLYDVNSTLKFRTLIDFDGQLTIYSKITPSLKLFYKTQASGQISKY
 401 EMNLDVDSQSLMTGSGEIKKRKKGFNGDLIFKYREKEFLALTISQEAKSQRERNYVIKSKTPWRNHIGSIKVMKDKKGSS
 481 KYETKFCREKEEDKCISIDLAHKEIPDGNEWNVCYKRQEVDFCIERIRADTKEVSRYHTVIYKGDTRYGYDVRFGKEGNG
 561 LMTSAGLILPSREIVNKLIVEFSLSKPKLKWELKLDAAKNPDRKLNVDLRFDNHLTDNQASKLDITITHPIYEKPIEIKL
 641 VGDLELSQNKIFTGEMSLDYSNDPEDKLIGKLAVEKGPVPTAILNIYHQDEQHLDFVVKLNGTVNENEEYFGLIWNWKDD
 721 SNVDQEAFIVFNWLFKERKISFAYQRPKFFYRLDADIVASQTLLDESQVTDVTSNLNGEISKYKIINDYATGCNKLYVLN
 801 DDGENKRSYELCHNEDGRKILSVLVEGLDDENQWTFQYSLEIIRKNWWTIGIINNFNPEFLGQTLFRLSALGDELTAAGV
 881 GTLPISDTARYKKIKSSFTSKIVQPSLRFFVPEATKFISGMIRDIQTLRQKLTFYYHTLPSFEEIRHCGKKVLAISQAIA
 961 KEIWNVVGSYYKNYLETTLKTVHKFFELVKKACDDNVECKAFVKAYHDDGFKGLVKQLRDYLLRLPDRIQHFLSQEHPEL
1041 RNFMGKAVTLLKEFLEPLTKYRCGEIVVKVVKLSYEKLEPIAQRFLRDFPSYYRAVRGKIVSNPYVIQAAKLAKETYLNI
1121 RETLLKIDYKKLYKLAIEKLEECLLNVNPPSLKKSFKVLALNTEKGIAEFEVYLATSHPLPRKIIDYTVSTTINIVKRLA
1201 DIQPSWKTVLVKVKRVADLNFFPPYKAQAMILGNQHYVTFDKEFFDFAGECTYLLTRDFEDGNFTFALTPKEGEKSPSIL
1281 AWINDASIELNPADKSVLVGDKAVELPYISKDFVVSRSGNAITINDRHGVEVKCNLVHELCTFSITGWYFGKTAGLLGTY
1361 NYEPKDDFKRPKGQIANSATVHAKSWELKKGCKSNNLVSEPRINERSDYYKLCRQHFVDDASPLSACYSEVSPADYMQLC
                                   1465
                                     C
1441 LRHLASASDSTKALCRVSAAYVAECERNYVELSLPNKCLVCTGPDGSTLEHSDKKKYENLSKKSSDVVFIIEDHECNKAV
                                 T   C
1521 IGELSNIARSIDRELQTEGFKDNMFGVIGYGGDVGSPEIFTVGGKTFFNSRDINSIVGRISLQSNKVESSRAFEAMKLAV
1601 TYLSRSDTSKNFVLFSCSSCKYDFKSLQYPVVQQILLERGISLHVVHSAEISIRKSKEKDIIGVDPDTVYHSKDVTQREL
1681 VGEPALRSQVSIPKDLCIALSQDVGGSFFSTYPLSKGTSDAKNWRSVFSRKFIKAVQSFKCQWCDCSSTRDHIPNTVCQP
1761 CETKRPRLPYSLYLTTDVKYF
```

Supporting Peptides:

Peptide	Uniq	-10lgP	Mass	Length	ppm	m/z	z	RT	Fraction	Scan	Source File	Area FE2 3uL	#Feature	#Feature FE2 3uL	Start	End	PTM	AScore	F
R.VSAAYVT(sub A)EC(+57.02)ER.N	Y	34.98	1283.5815	11	-0.6	642.7977	2	16.44	3	2952	211015-MClass-FE2-3uL-LSM.raw	3.8316E4	1	1	1457	1467	Carbamidomethylation	C9:Carbamidomethylation:1000.00	S
total 1 peptides																			

Best Unique PSM (Scan 2952, m/z=642.7977, z=2, RT=16.44, ppm=-0.6):

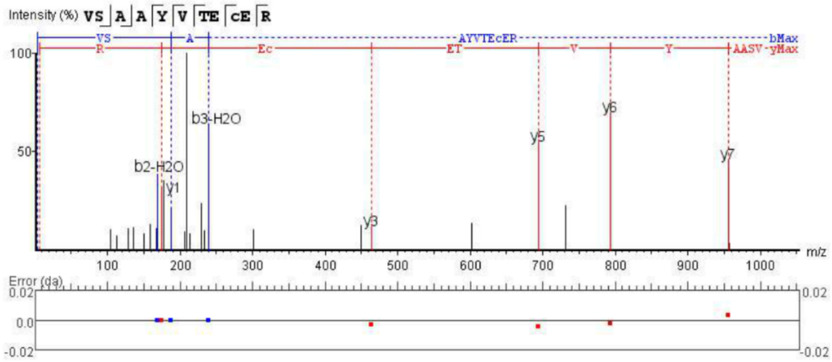

tr|A0A2L2XZR6|A0A2L2XZR6_PARTP back to list

| Protein Coverage | Supporting Peptides | Best Unique PSM |

Protein Coverage:

C Carbamidomethy
T A->T

```
   1 FSISSPSHYGTGSPHTLDFKWKRGVKAEFSIDTSYMEGQFAVENQSPVRSIKAEFNLKKVLNKKISVIGSVTPGDPNLLN
  81 VEVEWEGDGSQKKVVIDAKSNKGTEDGIEKYMITATVNYVGAISVDITGKVSTDLLRGPHYFRADYTAKEPIAIEFTHEI
 161 KNGKLVSFGRYLNNNAERLRMDIKGKMELTGYKFELDGGIFMTSPYKSLDGKEISFRIMADSTDAVRTFISEYRIMPVSF
 241 INYAGKLEYTRKRGWPGQIKSIALVSVHNRPSIEATTTIDYGNGKYSLKSSLTPLSRRKINLVSTFEHSGRFAAFHHSLA
 321 TSFEYLQTVDLKAVADLRNMEDAKIHSNFDINKHKLYDVNSTLKFRTLIDFDGQLTIYSKITPSLKLFYKTQASGQISKY
 401 EMNLDVDSQSLMTGSGEIKKRKKGFNGDLIFKYREKEFLALTISQEAKSQRERNYVIKSKTPWRNHIGSIKVMKDKKGSS
 481 KYETKFCREKEEDKCISIDLAHKEIPDGNEWNVCYKRQEVDFCIERIRADTKEVSRYHTVIYKGDTRYGYDVRFGKEGNG
 561 LMTSAGLILPSREIVNKLIVEFSLSKPKLKWELKLDAAKNPDRKLNVDLRFDNHLTDNQASKLDITITHPIYEKPIEIKL
 641 VGDLELSQNKIFTGEMSLDYSNDPEDKLIGKLAVEKGPVPTAILNIYHQDEQHLDFVVKLNGTVNENEEYFGLIWNWKDD
 721 SNVDQEAFIVFNWLFKERKISFAYQRPKFFYRLDADIVASQTLLDESQVTDVTSNLNGEISKYKIINDYATGCNKLYVLN
 801 DDGENKRSYELCHNEDGRKILSVLVEGLDDENQWTFQYSLEIIRKNWWTIGIINNFNPEFLGQTLFRLSALGDELTAAGV
 881 GTLPISDTARYKKIKSSFTSKIVQPSLRFFVPEATKFISGMIRDIQTLRQKLTFYYHTLPSFEEIRHCGKKVLAISQAIA
 961 KEIWNVVGSYYKNYLETTLKTVHKFFELVKKACDDNVECKAFVKAYHDDGFKGLVKQLRDYLLRLPDRIQHFLSQEHPEL
1041 RNFMGKAVTLLKEFLEPLTKYRCGEIVVKVVKLSYEKLEPIAQRFLRDFPSYYRAVRGKIVSNPYVIQAAKLAKETYLNI
1121 RETLLKIDYKKLYKLAIEKLEECLLNVNPPSLKKSFKVLALNTEKGIAEFEVYLATSHPLPRKIIDYTVSTTINIVKRLA
1201 DIQPSWKTVLVKVKRVADLNFFPPYKAQAMILGNQHYVTFDKEFFDFAGECTYLLTRDFEDGNFTFALTPKEGEKSPSIL
1281 AWINDASIELNPADKSVLVGDKAVELPYISKDFVVSRSGNAITINDRHGVEVKCNLVHELCTFSITGWYFGKTAGLLGTY
1361 NYEPKDDFKRPKGQIANSATVHAKSWELKKGCKSNNLVSEPRINERSDYYKLCRQHFVDDASPLSACYSEVSPADYMQLC
                                        1465
                                         C
1441 LRHLASASDSTKALCRVSAAYVAECERNYVELSLPNKCLVCTGPDGSTLEHSDKKKYENLSKKSSDVVFIIEDHECNKAV
                      T  C
1521 IGELSNIARSIDRELQTEGFKDNMFGVIGYGGDVGSPEIFTVGGKTFFNSRDINSIVGRISLQSNKVESSRAFEAMKLAV
1601 TYLSRSDTSKNFVLFSCSSCKYDFKSLQYPVVQQILLERGISLHVVHSAEISIRKSKEKDIIGVDPDTVYHSKDVTQREL
1681 VGEPALRSQVSIPKDLCIALSQDVGGSFFSTYPLSKGTSDAKNWRSVFSRKFIKAVQSFKCQWCDCSSTRDHIPNTVCQP
1761 CETKRPRLPYSVCRSCALPRTVEVFLSYIRHLI
```

Supporting Peptides:

Peptide	Uniq	-10lgP	Mass	Length	ppm	m/z	z	RT	Fraction	Scan	Source File	Area FE2 3uL	#Feature	#Feature FE2 3uL	Start	End	PTM	AScore	F
R.VSAAYVT(sub A)EC(+57.02)ER.N	Y	34.98	1283.5815	11	-0.6	642.7977	2	16.44	3	2952	211015-MClass-FE2-3uL-LSM.raw	3.8316E4	1	1	1457	1467	Carbamidomethylation	C9:Carbamidomethylation:1000.00	S
total 1 peptides																			

Best Unique PSM (Scan 2952, m/z=642.7977, z=2, RT=16.44, ppm=-0.6):

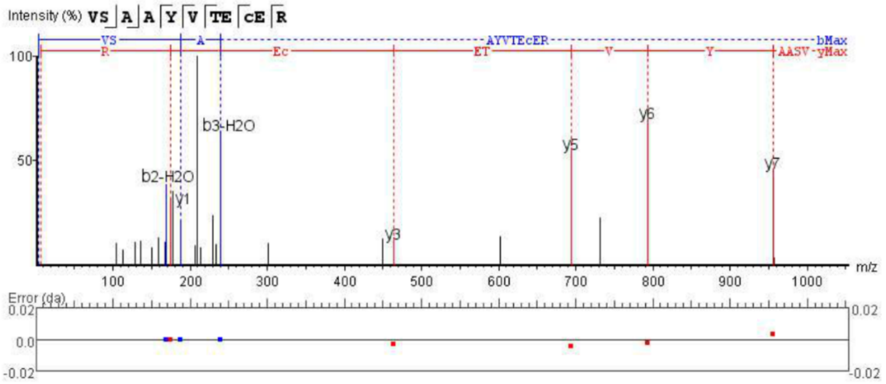

tr|A0A2L2Y9R4|A0A2L2Y9R4_PARTP

back to list

| Protein Coverage | Supporting Peptides | Best Unique PSM |

Protein Coverage:

```
  1 NFQCHELYEHDATNIGEVKSPFYDEGSYPDKLWCEYRIRAPAGYRIKITFKDLDIEPTGSCGHDKLVLYGKDKESVLGEF
 81 CGDLIPRPILSNSGESEIRLLFLSDEMVAGRGFRLQYESSPNIELCSSKEGSCRNRKCYPTEKKCDGVDDCGDGTDEEKC
161 GKPVSMPSDICGEPPIQPKTIYGSADRQVGGAEVVTNSWPWQVSLQHTYKEPNAHFCGGSLISPQWVVTAAHCVTGKPDP
241 QDLRIVLGSHNKYNKTKYEVTRIAEKIISYPDLEGEKLRQMSITHDIALIKLNAPVIYTDGIQPVCLPSLGWEAQPDWVC
321 YSTGWGETRG
```

Supporting Peptides:

Peptide	Uniq	-10lgP	Mass	Length	ppm	m/z	z	RT	Fraction	Scan	Source File	Area FE2 3uL	#Feature	#Feature FE2 3uL	Start	End	PTM	AScore	Found By
I.SYPDLEGEK.L	Y	34.93	1036.4713	9	0.1	519.2430	2	16.84	3	3160	211015-MClass-FE2-3uL-LSM.raw	1.3002E5	1	1	269	277			PEAKS DB
total 1 peptides																			

Best Unique PSM (Scan 3160, m/z=519.2430, z=2, RT=16.84, ppm=0.1):

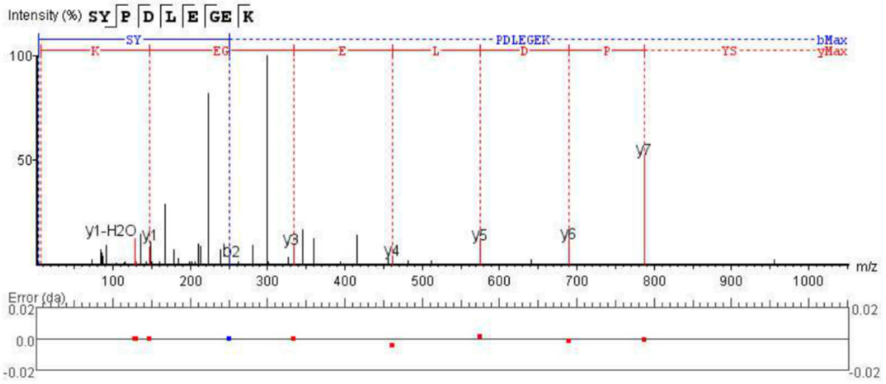

Peptide List

Prepared with PEAKS ™ (bioinfor.com)

Supplementary materials Table S1 The statistic of annotation databases of *Latrodectus geometricus* venom gland.

Annotation databases	NR	Anotation databases	InterPro
NR	3,364	NR	566
InterPro	566	InterPro	78
SwissProt	139	SwissProt	1
KEGG	655	KEGG	3
KOG	59	KOG	0
NR.SwissProt.KEGG	180	InterPro.SwissProt.KEGG	0
NR.InterPro.SwissProt	323	InterPro.SwissProt.NR	323
NR.KOG.InterPro	101	InterPro.KOG.NR	101
NR.KOG.SWissProt	750	InterPro.KOG.SWissProt	0
NR.SwissProt.InterPro.KEGG	578	InterPro.SwissProt.NR.KEGG	750
NR.SwissProt.InterPro.KEGG.KOG	10,773	InterPro.SwissProt.NR.KEGG.KOG	10,773
NR.InterPro.KEGG	371	InterPro.InterPro.KEGG	371
NR.KOG.KEGG.InterPro	156	InterPro.KEGG.SwissProt.NR	578
NR.SwissProt.KEGG.KOG	1,024	InterPro.SwissProt.KEGG.KOG	2
NR.KOG.KEGG	84	InterPro.KOG.KEGG	0
NR.SwissProt.KOG	119	InterPro.KOG.NR.KEGG	156
**Total (unigenes)**	**19,242**	**Total (unigenes)**	**13,702**
**Annotation databases**	**KEGG**	**Annotation databases**	**KOG**
NR	655	NR	59
InterPro	3	InterPro	0
SwissProt	2	SwissProt	7
KEGG	5	KEGG	1
KOG	1	KOG	1
KEGG.SwissProt.NR	180	KOG.NR.InterPro.KEGG	156
KEGG.NR.InterPro	371	KOG.InterPro.NR	101
KEGG.KOG.InterPro	0	KOG.KEGG.InterPro	0
KEGG.KOG.SwissProt	5	KOG.SwissProt.InterPro	0
KEGG.InterPro.SwissProt	0	KOG.InterPro.SwissProt.NR	750
KEGG.SwissProt.NR.InterPro.KOG	10,773	KOG.SwissProt.NR.KEGG.InterPro	10,773
KEGG.NR.KOG	84	KOG.KEGG.NR	84
KEGG.NR.InterPro.KOG	156	KOG.KEGG.SwissProt	5
KEGG.NR.KOG.SwissProt	1,024	KOG.NR.KEGG.SwissProt	1,024
KEGG.InterPro.SwissProt.NR	578	KOG.KEGG.InterPro.SwissProt	2
KEGG.KOG.InterPro.SwissProt	2	KOG.NR.SwissProt	119
**Total (unigenes)**	**13,839**	**Total (unigenes)**	**13,082**

Supplementary materials Table S1 The statistic of annotation databases of *Latrodectus geometricus* venom gland. (cont.)

Annotation databases	SwissProt
NR	139
InterPro	1
SwissProt	3
KEGG	2
KOG	7
SwissProt.KEGG.NR	180
SwissProt.InterPro.NR	323
SwissProt.KOG.KEGG	5
SwissProt.KEGG.InterPro	0
SwissProt.KOG.InterPro.NR	750
SwissProt.InterPro.NR.KEGG.KOG	10,773
SwissProt.InterPro.KEGG.KOG	2
SwissProt.InterPro.KOG	0
SwissProt.KOG.NR.KEGG	1,024
SwissProt.KEGG.InterPro.NR	578
SwissProt.KOG.NR	119
**Total (unigenes)**	**13,906**

Abbreviations:
NR: Non-redundant
KEGG: Kyoto Encyclopedia of Genes and Genomes
KOG: EuKaryotic Orthologous Groups

Supplementary materials Table S2 FPKM statistics of ion channel in *Latrodectus geometricus* venom gland.

Class name	Ion channels	FPKM
Cation channels	ATP-sensitive inward rectifier potassium channel	4.37
	Calcium load-activated calcium channel	13.115
	Calcium permeable stress-gated cation channel 1	0.9
	Calcium release-activated calcium channel protein 1	2.08
	Cyclic nucleotide-gated cation channel	1.36
	Mitochondrial potassium channel	24.3
	Open rectifier potassium channel protein 1	0.06
	Potassium channel subfamily K	12.685
	Potassium channel subfamily T	0.15
	Potassium voltage-gated channel protein Shab	0.41
	Potassium voltage-gated channel protein Shaker	0.315
	Potassium voltage-gated channel subfamily KQT	17.71
	Proton channel OtopLc	28.235
	Ryanodine receptor	20.415
	Transient receptor potential cation channel subfamily A	1.71
	Transient receptor potential cation channel subfamily M	2.13
	Transient receptor potential cation channel gamma	0.45
	Trimeric intracellular cation channel	0.45
	TWiK family of potassium channels	8.78
	Voltage-dependent calcium channel protein	4.77
	Voltage-dependent calcium channel subunit alpha-2/delta-1	1.865
	Voltage-dependent calcium channel subunit alpha-2/delta-3	17.665
	Voltage-dependent calcium channel type D subunit alpha-1	2.555
	Voltage-dependent T-type calcium channel subunit alpha-1H	0.06
	Voltage-gated hydrogen channel 1	1.1
	Chloride channel protein 2	0.47
	Chloride intracellular channel	29.425
	FMRFamide-activated amiloride-sensitive sodium channel	1.22

Supplementary materials Table S2 FPKM statistics of ion channel in *Latrodectus geometricus* venom gland. (cont.)

Class name	Ion channels	FPKM
Anion channels	Sodium channel protein 1 brain	0.175
	Sodium channel protein 60E	10.055
	Sodium channel protein type 11 subunit alpha	0.52
	Sodium channel protein type 4 subunit alpha A	0.43
	Sodium leak channel non-selective protein	2.06
	Volume-regulated anion channel subunit LRRC8A	6.295
Other channels	Acetylcholine receptor subunit alpha	0.15
	Acid-sensing ion channel	0.46
	Gamma-aminobutyric acid receptor (GABA receptor)	14.745
	Glutamate receptor	8.135
	Glutamate receptor ionotropic, kainate 2	10.56
	Glutamate receptor ionotropic, kainate 5	7.865
	Glutamate-gated chloride channel	8.28
	Glutamate-gated chloride channel alpha	0.09
	Glycine receptor subunit alpha-1	15.58
	Glycine receptor subunit alpha-2	0.18
	Glycine receptor subunit alpha-3	10.265
	Glycine receptor subunit beta-type 4	2.73
	Ionotropic receptor	1.13
	Ligand-gated ion channel	0.055
	Mucolipin-3 (Transient receptor potential channel mucolipin 3)	1.775
	Neuronal acetylcholine receptor subunit alpha	128.48
	Short transient receptor potential channel 5	6.365
	Transient receptor potential protein	0.28

Supplementary materials Table S3 Blastn/x of 20 most expressed unigenes of *Latrodectus geometricus* venom gland.

No.	FPKM	UniProt.BlastP	Organism	Uniprot ID	E-value
1	60435	α-latrotoxin associated low molecular weight protein 2	*Latrodectus geometricus*	V9QFG7	1.60E-58
2	19855	α-latrotoxin associated low molecular weight protein 2	*Latrodectus geometricus*	V9QFG7	6.11E-36
3	16884.6	Neprilysin-2	*Drosophila melanogaster*	A0A0B4K692	8.94E-88
4	15068.8	Cytochrome c oxidase subunit 3 (EC 7.1.1.9)	*Allomyces macrogynus*	P80439	5.35E-21
5	14841.7	α-latrotoxin associated low molecular weight protein 2	*Latrodectus geometricus*	V9QFG7	6.11E-36
6	13108	U4-lycotoxin-Ls1a	*Lycosa singoriensis*	B6DCT8	8.57E-17
7	12095.7	Actin, cytoplasmic 1 (Beta-actin-1)	*Danio rerio*	Q7ZVI7	4.65E-67
8	9714.59	CRISP/Allergen/PR-1	*Trittame loki*	W4VS53	4.22E-77
9	9472.3	Muscle LIM protein Mlp84B	*Drosophila melanogaster*	Q24400	3.47E-36
10	7188.99	Myophilin	*Echinococcus granulosus*	Q24799	2.98E-73
11	6268.11	CRISP/Allergen/PR-1	*Trittame loki*	W4VS53	7.06E-86
12	6188.7	Myosin regulatory light chain 2	*Bombyx mori*	Q1HPS0	1.76E-60
13	5666.96	Troponin C (Allergen Tyr p 24)	*Tyrophagus putrescentiae*	D2DGW3	1.90E-87
14	4977.5	ATP synthase subunit a	*Rhipicephalus sanguineus*	O99821	1.11E-17
15	4684.58	Pre-mRNA-splicing factor CWC25 homolog	*Mus musculus*	Q9DBF7	4.38E-56
16	4348.68	Tropomyosin	*Rhipicephalus microplus*	O97162	1.22E-164
17	4214.34	ω-ctenitoxin-Cs1a	*Cupiennius salei*	P81694	6.51E-11
18	3446.56	CRISP/Allergen/PR-1	*Trittame loki*	W4VS53	3.24E-88
19	2865.83	U-scoloptoxin (01)-Cw1a	*Cormocephalus westwoodi*	P0DPW5	6.29E-13
20	2844.12	CRISP/Allergen/PR-1 (Cap-1)	*Trittame loki*	W4VS53	8.36E-07

Supplementary materials Table S4 Clustering classification of *Latrodectus geometricus* venom gland.

No.	Unigenes	Cluster	Strategy	Domain	Homologous description	FPKM	Number of cysteine
**Agatoxin family**							
1	GBJM01003319	Agatoxin family	Homology	-	U8-agatoxin-Ao1a	0.26	8
2	GBJM01112589	Agatoxin family	Homology	-	U8-agatoxin-Ao1a	0	9
3	GBJM01112590	Agatoxin family	Domain	Toxin 9	-	0	10
4	GBJM01019804	Agatoxin family	Domain	Toxin 9	-	0	8
5	GBJM01054266	Agatoxin family	Domain	Toxin 9	-	0	8
**ANK family**							
6	GBJM01062411	Alpha-LCT-Lt1a family	Homology	-	α-latrocrustotoxin-Lt1a	4.42	10
7	GBJM01001888	Alpha-LIT-Lt1a family	Homology	-	α-latroinsectotoxin-Lt1a	31.555	4
8	GBJM01059725	Alpha-LIT-Lt1a family	Domain	ANK	-	1.23	44
9	GBJM01066462	Alpha-LIT-Lt1a family	Homology	-	α-latroinsectotoxin-Lt1a	0.52	5
10	GBJM01116720	Alpha-LIT-Lt1a family	Domain	ANK	-	4.74	16
11	GBJM01131353	Alpha-LIT-Lt1a family	Domain	ANK	-	2.88	17
12	GBJM01049106	Alpha-LIT-Lt1a family	Domain	ANK	-	0	11
13	GBJM01058469	Alpha-LIT-Lt1a family	Domain	ANK	-	0.06	16
14	GBJM01023374	Alpha-LIT-Lt1a family	Domain	ANK	-	0	5
15	GBJM01128973	Alpha-LIT-Lt1a family	Domain	ANK	-	1.555	8
16	GBJM01039245	Alpha-LIT-Lt1a family	Domain	ANK	-	0.06	11
17	GBJM01090534	Alpha-LIT-Lt1a family	Domain	ANK	-	10.43	11
18	GBJM01005762	Alpha-LTX-Lh1a family	Homology	-	α-latrotoxin-Lh1a	5.805	5

Supplementary materials Table S4 Clustering classification of *Latrodectus geometricus* venom gland. (cont.)

No.	Unigenes	Cluster	Strategy	Domain	Homologous description	FPKM	Number of cysteine
19	GBJM01062969	Alpha-LTX-Lh1a family	Homology	-	α-latrotoxin-Lh1a	0.66	1
20	GBJM01051925	Alpha-LTX-Lh1a family	Homology	-	α-latrotoxin-Lh1a	2.005	4
21	GBJM01109243	Alpha-LTX-Lh1a family	Homology	-	α-latrotoxin-Lh1a	11.92	3
22	GBJM01116664	Alpha-LTX-Lh1a family	Homology	-	α-latrotoxin-Lh1a	7.025	2
23	GBJM01022611	Alpha-LTX-Lt1a family 1	Domain	ANK	-	0.915	28
24	GBJM01115563	Alpha-LTX-Lt1a family 1	Domain	ANK	-	0.02	16
25	GBJM01056593	Alpha-LTX-Lt1a family 1	Domain	ANK	-	0.355	15
26	GBJM01130085	Alpha-LTX-Lt1a family 1	Domain	ANK	-	4.895	9
27	GBJM01139013	Alpha-LTX-Lt1a family 1	Domain	ANK	-	4.065	11
28	GBJM01069082	Alpha-LTX-Lt1a family 1	Domain	ANK	-	0	9
29	GBJM01030058	Alpha-LTX-Lt1a family 1	Domain	ANK	-	5.69	8
30	GBJM01003560	Alpha-LTX-Lt1a family 1	Domain	ANK	-	0	10
31	GBJM01005960	Alpha-LTX-Lt1a family 1	Homology	-	α-latrotoxin-Lt1a	1.735	4
32	GBJM01140036	Alpha-LTX-Lt1a family 1	Homology	-	α-latrotoxin-Lt1a	6.165	2
33	GBJM01103342	Alpha-LTX-Lt1a family 1	Homology	-	α-latrotoxin-Lt1a	0.62	0
34	GBJM01035503	Alpha-LTX-Lt1a family 1	Homology	-	α-latrotoxin-Lt1a	712.85	1
35	GBJM01117216	Alpha-LTX-Lt1a family 1	Homology	-	α-latrotoxin-Lt1a	447.14	2
36	GBJM01087040	Alpha-LTX-Lt1a family 1	Homology	-	α-latrotoxin-Lt1a	0	0
37	GBJM01118364	Alpha-LTX-Lt1a family 1	Homology	-	α-latrotoxin-Lt1a	28.81	2
38	GBJM01138719	Alpha-LTX-Lt1a family 1	Homology	-	α-latrotoxin-Lt1a	5.16	2

Supplementary materials Table S4 Clustering classification of *Latrodectus geometricus* venom gland. (cont.)

No.	Unigenes	Cluster	Strategy	Domain	Homologous description	FPKM	Number of cysteine
39	GBJM01083923	Alpha-LTX-Lt1a family 1	Homology	-	α-latrotoxin-Lt1a	21.62	2
40	GBJM01081561	Alpha-LTX-Lt1a family 2	Domain	ANK	-	776.18	10
41	GBJM01091322	Delta-LIT-Lt1a family	Homology	-	δ-latroinsectotoxin-Lt1a	0	0
42	GBJM01015437	Delta-LIT-Lt1a family	Homology	-	δ-latroinsectotoxin-Lt1a	0	1
43	GBJM01032236	Delta-LIT-Lt1a family	Homology	-	δ-latroinsectotoxin-Lt1a	0.685	2
44	GBJM01090931	Delta-LIT-Lt1a family	Homology	-	δ-latroinsectotoxin-Lt1a	0	0
45	GBJM01044964	Delta-LIT-Lt1a family	Homology	-	δ-latroinsectotoxin-Lt1a	0.95	0
46	GBJM01082559	Delta-LIT-Lt1a family	Homology	-	δ-latroinsectotoxin-Lt1a	1.9	0
47	GBJM01109678	Delta-LIT-Lt1a family	Homology	-	δ-latroinsectotoxin-Lt1a	0	0
48	GBJM01057151	ANK family	Domain	ANK	-	0	0
49	GBJM01072127	ANK family	Domain	ANK	-	0	0
50	GBJM01136397	ANK family	Domain	ANK	-	7.12	9
51	GBJM01070559	ANK family	Domain	ANK	-	3.77	3
52	GBJM01005424	ANK family	Domain	ANK	-	0.115	9
53	GBJM01110061	ANK family	Domain	ANK	-	0.155	9
54	GBJM01084503	ANK family	Domain	ANK	-	22.665	11
55	GBJM01094360	ANK family	Domain	ANK	-	3.62	11
56	GBJM01073508	ANK family	Domain	ANK	-	2.965	4
57	GBJM01115112	ANK family	Domain	ANK	-	0.665	4
58	GBJM01064051	ANK family	Domain	ANK	-	0	2

Supplementary materials Table S4 Clustering classification of *Latrodectus geometricus* venom gland. (cont.)

No.	Unigenes	Cluster	Strategy	Domain	Homologous description	FPKM	Number of cysteine
59	GBJM01133263	ANK family	Domain	ANK	-	30.825	7
60	GBJM01040666	ANK family	Domain	ANK	-	29.44	9
61	GBJM01080979	ANK-like family	Domain	ANK	-	3.145	32
62	GBJM01023505	ANK-like family	Domain	ANK	-	2.425	15
63	GBJM01089421	ANK-like family	Domain	ANK	-	0.985	9
64	GBJM01051542	ANK-like family	Domain	ANK	-	4.4	7
65	GBJM01134636	ANK-like family	Domain	ANK	-	0.035	5
66	GBJM01068333	ANK-like family	Domain	ANK	-	0.185	15
67	GBJM01089982	ANK-like family	Domain	ANK	-	0.55	8
68	GBJM01018329	ANK-like family	Domain	ANK	-	7.38	9
69	GBJM01067017	ANK-like family	Domain	ANK	-	1.42	5
70	GBJM01023107	ANK-like family	Domain	ANK	-	5.19	6
71	GBJM01071097	ANK-like family	Domain	ANK	-	2.775	1
72	GBJM01134638	ANK-like family	Domain	ANK	-	0	3
73	GBJM01039436	ANK-like family	Domain	ANK	-	1.72	1
74	GBJM01086505	ANK-like family	Domain	ANK	-	0	3
75	GBJM01134637	ANK-like family	Domain	ANK	-	0.06	3
76	GBJM01115111	ANK-like family	Domain	ANK	-	0	5
77	GBJM01008005	ANK-like family	Domain	ANK	-	0	5
78	GBJM01115745	ANK-like family	Domain	ANK	-	9.1	6

Supplementary materials Table S4 Clustering classification of *Latrodectus geometricus* venom gland. (cont.)

No.	Unigenes	Cluster	Strategy	Domain	Homologous description	FPKM	Number of cysteine
79	GBJM01070919	ANK-like family	Domain	ANK	-	0	4
80	GBJM01025338	ANK-like family	Domain	ANK	-	52.225	2
81	GBJM01078368	ANK-like family	Domain	ANK	-	0.025	5
82	GBJM01090363	ANK-like family	Domain	ANK	-	0	4
83	GBJM01113810	ANK-like family	Domain	ANK	-	0	2
84	GBJM01023511	ANK-like family	Domain	ANK	-	1.765	3
85	GBJM01055400	ANK-like family	Domain	ANK	-	0	6
86	GBJM01103556	ANK-like family	Domain	ANK	-	0	1
87	GBJM01037186	ANK-like family	Domain	ANK	-	63.695	3
88	GBJM01094331	ANK-like family	Domain	ANK	-	1.815	1
89	GBJM01093198	ANK-like family	Domain	ANK	-	1.375	2
90	GBJM01097820	ANK-like family	Domain	ANK	-	0	1
91	GBJM01003594	ANK-like family	Domain	ANK	-	0.785	1
92	GBJM01005426	ANK-like family	Domain	ANK	-	0.145	2
93	GBJM01043344	ANK-like family	Domain	ANK	-	0	1
94	GBJM01064903	ANK-like family	Domain	ANK	-	0	1
95	GBJM01023661	ANK-like family	Domain	ANK	-	0	2
96	GBJM01008004	ANK-like family	Domain	ANK	-	5.24	3
97	GBJM01027088	ANK-like family	Domain	ANK	-	0	2
98	GBJM01121255	ANK-like family	Domain	ANK	-	0	1

Supplementary materials Table S4 Clustering classification of *Latrodectus geometricus* venom gland. (cont.)

No.	Unigenes	Cluster	Strategy	Domain	Homologous description	FPKM	Number of cysteine
99	GBJM01072066	ANK-like family	Domain	ANK	-	0	3
**Centipede toxin family**							
100	GBJM01115433	Centipede toxin family	Homology	-	Scoloptoxin SSD14	7.99	9
101	GBJM01137901	Centipede toxin family	Homology	-	Scoloptoxin SSD14	11.53	4
102	GBJM01097142	Centipede toxin family	Homology	-	Scoloptoxin SSD20	0	0
103	GBJM01079633	Centipede toxin family	Homology	-	U-scoloptoxin(01)-Cw1a	1.655	7
104	GBJM01072122	Centipede toxin family	Homology	-	U-scoloptoxin(01)-Cw1a	30.315	7
105	GBJM01136046	Centipede toxin family	Homology	-	U-scoloptoxin(01)-Cw1a	3.375	8
106	GBJM01106443	Centipede toxin family	Homology	-	U-scoloptoxin(01)-Cw1a	0	8
107	GBJM01100968	Centipede toxin family	Homology	-	U-scoloptoxin(01)-Cw1a	0	7
108	GBJM01030198	Centipede toxin family	Homology	-	U-scoloptoxin(01)-Cw1a	55.735	10
109	GBJM01031569	Centipede toxin family	Homology	-	U-scoloptoxin(01)-Cw1a	1.725	6
110	GBJM01051153	Centipede toxin family	Homology	-	U-scoloptoxin(01)-Cw1a	0.04	9
111	GBJM01103095	Centipede toxin family	Homology	-	U-scoloptoxin(01)-Cw1a	2865.83	6
112	GBJM01042369	Centipede toxin family	Homology	-	U-scoloptoxin(01)-Cw1a	70.13	7
113	GBJM01022671	Centipede toxin family	Homology	-	U-scoloptoxin(01)-Cw1a	0.725	9
114	GBJM01089616	Centipede toxin family	Homology	-	U-scoloptoxin(01)-Cw1a	0	6
115	GBJM01010476	Centipede toxin family	Homology	-	U-scoloptoxin(01)-Er1a	0	6
116	GBJM01055217	Centipede toxin family	Homology	-	U-scoloptoxin(01)-Er1a	0.105	6
117	GBJM01078726	Centipede toxin family	Homology	-	U-scoloptoxin(01)-Er1a	35.955	7

Supplementary materials Table S4 Clustering classification of *Latrodectus geometricus* venom gland. (cont.)

No.	Unigenes	Cluster	Strategy	Domain	Homologous description	FPKM	Number of cysteine
118	GBJM01124420	Centipede toxin family	Homology	-	U-scoloptoxin(01)-Er1a	0	7
119	GBJM01032789	Centipede toxin family	Homology	-	U-scoloptoxin(01)-Er1a	0	6
120	GBJM01022996	Centipede toxin family	Homology	-	U-scoloptoxin(16)-Er7a	5.69	9
121	GBJM01003609	Centipede toxin family	Homology	-	U-scoloptoxin(16)-Er7a	0	9
122	GBJM01105861	Centipede toxin family	Homology	-	U-scoloptoxin(16)-Sm3a	0	9
123	GBJM01133952	Centipede toxin family	Homology	-	U-scoloptoxin(16)-Sm4a	0.21	10
**Ctenitoxin family**							
124	GBJM01063839	Ctenitoxin family	Homology	-	U19-ctenitoxin-Pn1a	12.695	11
125	GBJM01129008	Ctenitoxin family	Homology	-	U24-ctenitoxin-Pn1a	0	10
126	GBJM01112330	Ctenitoxin family	Homology	-	U24-ctenitoxin-Pn1a	1.775	14
127	GBJM01092149	Ctenitoxin family	Homology	-	U24-ctenitoxin-Pn1a	3.53	13
128	GBJM01116416	Ctenitoxin family	Homology	-	U24-ctenitoxin-Pn1a	0.275	14
129	GBJM01101510	Ctenitoxin family	Homology	-	U24-ctenitoxin-Pn1a	67.79	13
130	GBJM01031395	Ctenitoxin family	Homology	-	U24-ctenitoxin-Pn1a	1.39	13
131	GBJM01076138	Ctenitoxin family	Homology	-	U24-ctenitoxin-Pn1a	0	9
132	GBJM01071729	Ctenitoxin family	Homology	-	U24-ctenitoxin-Pn1a	0	9
133	GBJM01053665	Ctenitoxin family	Homology	-	U24-ctenitoxin-Pn1a	2.455	8
134	GBJM01041200	Ctenitoxin family	Homology	-	U24-ctenitoxin-Pn1a	0.75	12
135	GBJM01084005	Ctenitoxin family	Homology	-	U24-ctenitoxin-Pn1a	105.845	12
136	GBJM01104623	Ctenitoxin family	Homology	-	U24-ctenitoxin-Pn1a	0	11

Supplementary materials Table S4 Clustering classification of *Latrodectus geometricus* venom gland. (cont.)

No.	Unigenes	Cluster	Strategy	Domain	Homologous description	FPKM	Number of cysteine
137	GBJM01138178	Ctenitoxin family	Homology	-	U24-ctenitoxin-Pn1a	0.605	16
138	GBJM01048313	Ctenitoxin family	Homology	-	U24-ctenitoxin-Pn1a	0.06	10
139	GBJM01121804	Ctenitoxin family	Homology	-	U9-ctenitoxin-Pr1a	0	6
140	GBJM01036734	Ctenitoxin family	Homology	-	U9-ctenitoxin-Pr1a	21.71	11
141	GBJM01100171	Ctenitoxin family	Homology	-	U9-ctenitoxin-Pr1a	0.14	11
142	GBJM01099190	Ctenitoxin family	Domain	TY	-	10.245	28
143	GBJM01106598	Ctenitoxin family	Domain	TY	-	0.07	31
144	GBJM01028547	Ctenitoxin family	Domain	TY	-	0	20
145	GBJM01003199	Ctenitoxin family	Domain	TY	-	0	9
146	GBJM01095503	Ctenitoxin family	Domain	TY	--	0	9
147	GBJM01021096	Ctenitoxin family	Domain	TY	-	9.95	10
148	GBJM01044701	Ctenitoxin family	Domain	TY	-	0	10
149	GBJM01062807	Ctenitoxin family	Domain	TY	-	0.24	7
150	GBJM01011174	Ctenitoxin family	Domain	TY	-	0.05	8
151	GBJM01128112	Ctenitoxin family	Domain	TY	-	0	10
152	GBJM01101299	Ctenitoxin family	Domain	TY	-	0	10
153	GBJM01020865	Ctenitoxin family	Domain	TY	-	0.425	9
154	GBJM01134247	Ctenitoxin family	Domain	TY	-	0.1	9
155	GBJM01036028	Ctenitoxin family	Domain	TY	-	0.095	7
156	GBJM01060745	Ctenitoxin family	Domain	TY	-	0.895	8

Supplementary materials Table S4 Clustering classification of *Latrodectus geometricus* venom gland. (cont.)

No.	Unigenes	Cluster	Strategy	Domain	Homologous description	FPKM	Number of cysteine
157	GBJM01109069	Ctenitoxin family	Domain	TY	-	0.19	8
158	GBJM01121779	Ctenitoxin family	Domain	TY	-	2.19	8
159	GBJM01050951	Ctenitoxin family	Domain	TY	-	0.72	8
160	GBJM01020865	Ctenitoxin family	Domain	TY	-	0.425	5
161	GBJM01030095	Ctenitoxin family	Domain	TY	-	1.46	8
162	GBJM01031597	Ctenitoxin family	Domain	TY	-	0.845	8
163	GBJM01080217	Ctenitoxin family	Domain	TY	-	12.95	8
164	GBJM01080218	Ctenitoxin family	Domain	TY	-	0.51	8
**Lycotoxin family**							
165	GBJM01087930	Lycotoxin family	Homology	-	ω-CNTX-Cs1a	4214.34	9
166	GBJM01106920	Lycotoxin family	Homology	-	U12-lycotoxin-Ls1c	4654.58	10
167	GBJM01118330	Lycotoxin family	Homology	-	U14-lycotoxin-Ls1b	2.235	13
168	GBJM01024858	Lycotoxin family	Homology	-	U14-lycotoxin-Ls1b	2.56	10
169	GBJM01097875	Lycotoxin family	Homology	-	U14-lycotoxin-Ls1b	2.05	10
170	GBJM01109572	Lycotoxin family	Homology	-	U15-lycotoxin-Ls1d	0	11
171	GBJM01020778	Lycotoxin family	Domain	Toxin 35	-	2.47	8
172	GBJM01074903	Lycotoxin family	Domain	Toxin 35	-	13108	9
**Scorpion toxin like family**							
173	GBJM01064203	Scorpion toxin like family	Homology	-	La1-like protein 13	1.16	9
174	GBJM01078041	Scorpion toxin like family	Homology	-	Toxin-like protein 14	0	7

Supplementary materials Table S4 Clustering classification of *Latrodectus geometricus* venom gland. (cont.)

No.	Unigenes	Cluster	Strategy	Domain	Homologous description	FPKM	Number of cysteine
175	GBJM01094424	Scorpion toxin like family	Homology	-	Toxin-like protein 14	0.29	9
176	GBJM01098304	Scorpion toxin like family	Homology	-	Toxin-like protein 14	0	10
177	GBJM01002509	Scorpion toxin like family	Homology	-	Toxin-like protein 14	0.125	10
178	GBJM01005360	Scorpion toxin like family	Homology	-	Toxin-like protein 14	0.405	10
179	GBJM01089166	Scorpion toxin like family	Homology	-	Toxin-like protein 14	0	10
180	GBJM01040129	Scorpion toxin like family	Homology	-	Toxin-like protein 14	2.065	10
181	GBJM01040128	Scorpion toxin like family	Homology	-	Toxin-like protein 14	3.64	10
182	GBJM01034780	Scorpion toxin like family	Homology	-	Toxin-like protein 14	0	8
183	GBJM01026824	Scorpion toxin like family	Homology	-	Toxin-like protein 14	0.75	9
184	GBJM01104094	Scorpion toxin like family	Homology	-	Toxin-like protein 14	0	8
185	GBJM01082588	Scorpion toxin like family	Homology	-	Toxin-like protein 14	15.795	9
186	GBJM01042621	Scorpion toxin like family	Homology	-	Toxin-like protein 14	3.86	10
187	GBJM01020415	Scorpion toxin like family	Homology	-	Toxin-like protein 14	0	10
188	GBJM01020414	Scorpion toxin like family	Homology	-	Toxin-like protein 14	0.72	10
189	GBJM01035234	Scorpion toxin like family	Homology	-	Toxin-like protein 14	11.975	10
190	GBJM01018960	Scorpion toxin like family	Homology	-	Toxin-like protein 14	1.27	10
191	GBJM01032588	Scorpion toxin like family	Homology	-	Toxin-like protein 14	51.565	10
**SCP family**							
192	GBJM01026995	SCP family	Homology	-	CRISP/Allergen/PR-1	0	7
193	GBJM01121080	SCP family	Homology	-	CRISP/Allergen/PR-1	6268.11	20

Supplementary materials Table S4 Clustering classification of *Latrodectus geometricus* venom gland. (cont.)

No.	Unigenes	Cluster	Strategy	Domain	Homologous description	FPKM	Number of cysteine
194	GBJM01005256	SCP family	Homology	-	CRISP/Allergen/PR-1	2844.12	3
195	GBJM01137067	SCP family	Homology	-	CRISP/Allergen/PR-1	3446.56	20
196	GBJM01140851	SCP family	Homology	-	CRISP/Allergen/PR-1	0.26	21
197	GBJM01005255	SCP family	Homology	-	CRISP/Allergen/PR-1	9714.59	20
198	GBJM01058555	SCP family	Homology	-	CRISP/Allergen/PR-1	1734.95	35
199	GBJM01055086	SCP family	Domain	SCP	-	17.775	17
200	GBJM01057984	SCP family	Domain	SCP	-	2.23	16
201	GBJM01104565	SCP family	Domain	SCP	-	8.61	17
202	GBJM01056476	SCP family	Domain	SCP	-	0	4
203	GBJM01057682	SCP family	Domain	SCP	-	1.755	3
204	GBJM01038322	SCP family	Domain	SCP	-	5.195	3
205	GBJM01052569	SCP family	Domain	SCP	-	11.64	2
206	GBJM01002830	SCP family	Domain	SCP	-	48.05	2
207	GBJM01104566	SCP family	Domain	SCP	-	5.435	14
**Theriditoxin family**							
208	GBJM01000181	Theriditoxin family	Homology	-	α-latrotoxin-associated LMWP	3714.28	7
209	GBJM01099609	Theriditoxin family	Homology	-	α-latrotoxin-associated LMWP	0	3
210	GBJM01056830	Theriditoxin family	Homology	-	α-latrotoxin-associated LMWP-2	0	7

Supplementary materials Table S4 Clustering classification of *Latrodectus geometricus* venom gland. (cont.)

No.	Unigenes	Cluster	Strategy	Domain	Homologous description	FPKM	Number of cysteine
211	GBJM01047487	Theriditoxin family	Homology	-	α-latrotoxin-associated LMWP-2	14841.68	4
212	GBJM01047488	Theriditoxin family	Homology	-	α-latrotoxin-associated LMWP-2	60435.04	7
